# Photoredox/Nickel-Catalyzed Diastereoselective Allylation of Aldehydes with Morita–Baylis–Hillman Adducts

**DOI:** 10.1021/acscatal.5c00891

**Published:** 2025-05-02

**Authors:** Francesco Calogero, Emanuele Pinosa, Andrea Gualandi, Luca Sensoli, Sayan Dutta, Bholanath Maity, Luigi Cavallo, Andrea Fermi, Paola Ceroni, Pier Giorgio Cozzi

**Affiliations:** † Alma Mater Studiorum-Dipartimento di Chimica “G. Ciamician”, Università di Bologna, Via Gobetti 83, Bologna 40129, Italy; ‡ Center for Chemical Catalysis - C3, Alma Mater Studiorum, Università di Bologna, Via Gobetti 83, Bologna 40129, Italy; § Physical Science and Engineering Division, King Abdullah University of Science and Technology (KAUST), Thuwal 23955-6900, Saudi Arabia

**Keywords:** photoredox catalysis, nickel catalysis, Morita−Baylis−Hillman acetates, aldehydes, allylation reaction, diastereoselective reaction

## Abstract

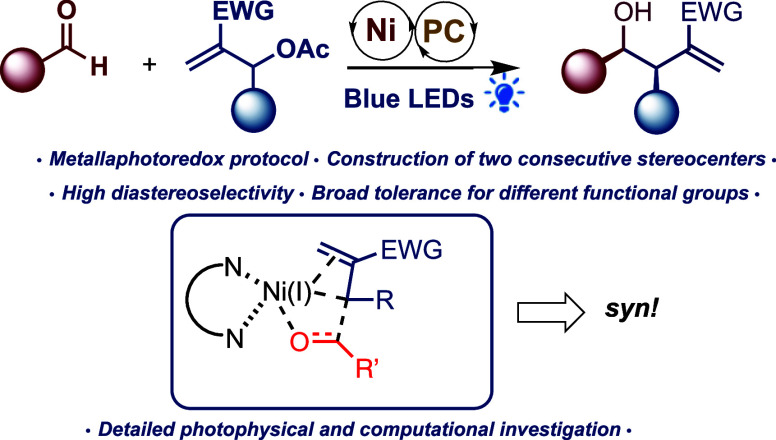

A practical diastereoselective photoredox nickel-mediated allylation of aliphatic and aromatic aldehydes with Morita–Baylis–Hillman (MBH) acetates is reported here. The reaction proceeds under visible-light irradiation using MBH derivatives, which are easily prepared from inexpensive and available starting materials, affording functionalized alcohols as a single diastereoisomer (>20:1 for the *syn* diastereoisomer). The popular organic dye 3DPAFIPN is used as a photocatalyst in combination with Hantzsch ester in the presence of a catalytic amount of stable nickel complexes and *o*-phenanthroline as a ligand. The reaction shows a broad scope, avoiding the use of Ni(0), as well as the stoichiometric metal reductants such as Zn and Mn. The relevance of the reaction was confirmed by using the prepared products for rapid access to α-methylene-β-lactones, which are useful intermediates for the synthesis of natural or biologically active relevant products. DFT calculations suggest that the allyl acetate-coordinated Ni(0) species plays the role of active catalyst and the nickel catalytic cycle operates through the Ni(0)–Ni(II)–Ni(I)–Ni(0) pathway.

## Introduction

The Morita–Baylis–Hillman (MBH) reaction^1^ is a well-known and established reaction used in organic synthesis to produce a variety of functionalized starting materials.^2^ Stereoselective variants of MBH promoted by Lewis bases,^3^ Lewis acids,^4^ and metal complexes have been reported.^5^ What makes the reaction unique and interesting is the possibility to further transform the obtained intermediates and increase the molecular diversity and complexity.^6^ The electrophilicity of the MBH adducts has been exploited in a variety of transformations and reactions,^7^ using organocatalytic methodologies,^8^ promoted by chiral amines,^9^ phosphines,^10^ or electrosynthesis.^11^ On the other hand, organometallic methodologies that can activate the MBH adducts via the formation of electrophilic metal intermediates have also been explored.^12^ In this regard, the formation of palladium,^13^ iridium,^14^ or nickel^15^ allyl electrophiles from MBH compounds has been used as a key strategy to add a range of nucleophiles, even in a stereoselective manner.^16^ In addition, cross-coupling reactions^17^ between MBH adducts and various reagents^18^ have been reported as a possible route to nucleophilic allylation of compounds.^19^ However, organocatalytic and organometallic methods have also been used to activate the MBH adducts and were also employed to form reactive nucleophilic species.^20^ Recently, the concept of dual metal and photoredox catalysis^21^ has become a rather unique topic with wide applications in organic chemistry, both in academia and in industry.^22^ In addition, the use of inexpensive, easily prepared, and efficient organic dyes represents another advantage in this research.^23,24^ The use of photocatalysts (organometallic complexes or organic dyes) in the framework of electron transfer events^25^ promotes the production of radicals under mild and controlled conditions.^26^ Radical chemistry and radical disconnections^27^ can now be easily considered in the synthetic design of complex molecules. Taking advantage of the easy and effective generation of radicals under photoredox conditions, MBH derivatives have also been used in photoredox catalysis^28^ in some examples of radical Michael reactions. However, MBH adducts can also be used under photoredox conditions in the presence of transition metals. We and others have recently explored the generation of nucleophilic organometallic reagents^29^ in photoredox conditions by using cobalt,^30^ chromium,^31^ titanium,^32^ and nickel^33^ in their low oxidation states.

**Figure 1:**
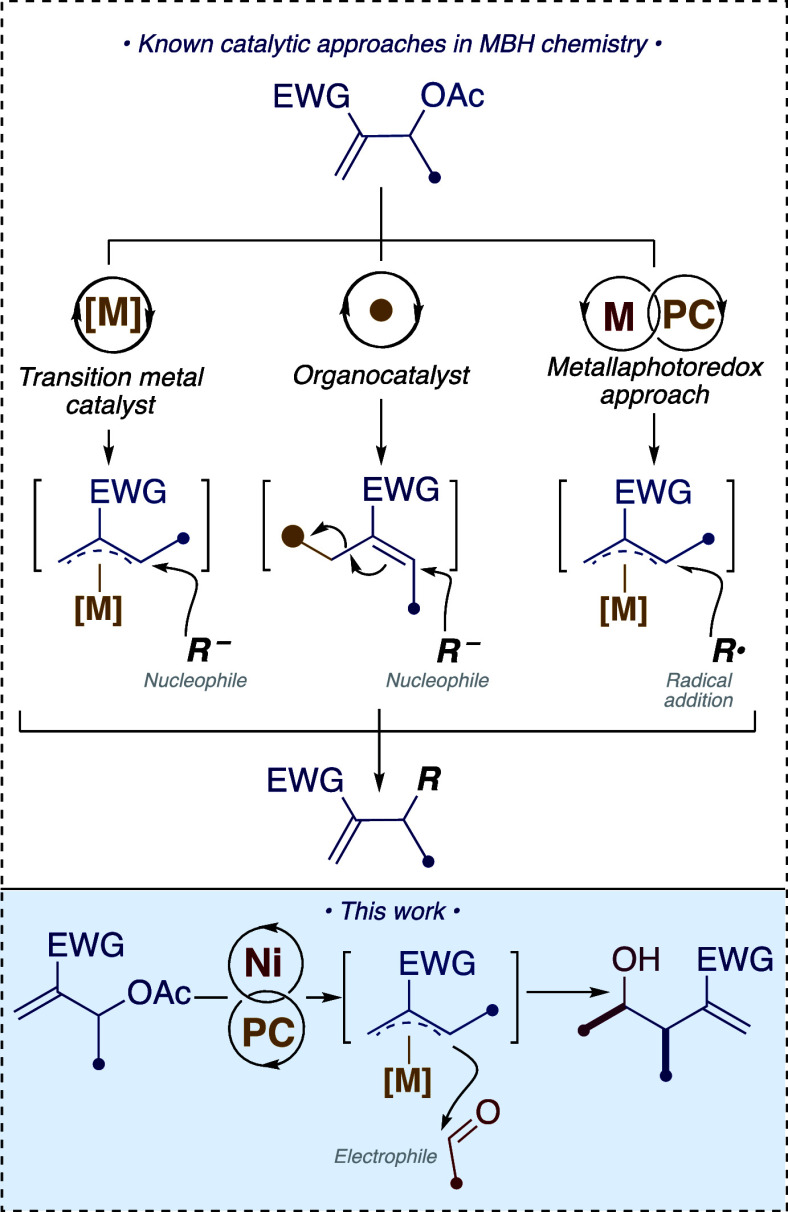
Electrophilic behavior of MBH acetates compared with our nucleophilic nickel photoredox reactions.

In light of these studies and the potential for preparing π-allyl MBH adducts with various metals, we hypothesized that MBH acetates could serve as useful starting materials for the preparation of nucleophilic organometallic nickel reagents^29b,33^ by applying photoredox conditions in combination with nickel catalysis to produce highly functionalized and densely decorated compounds (Figure 1). In our study, we found that it was indeed possible to generate nucleophilic organometallic π-allylic nickel reagents using various functionalized MBH adducts. These reagents were successfully employed as nucleophiles under photoredox conditions with aliphatic and aromatic aldehydes, resulting in the formation of various diversified homoallylic alcohols in excellent to good yields in favor of the *syn* diastereoisomer (dr > 20:1).

## Methods

The nickel-promoted photoredox reaction was carefully optimized by varying several reaction parameters using *p*-chlorobenzaldehyde **1a** as the model substrate (Table 1) and the simply prepared MBH adduct **2a** (see Supporting Information for the general preparation and all of the MBH adducts used in this study). 3DPAFIPN, NiCl_2_(glyme) and *o*-phenanthroline form the metallaphotoredox catalytic system in THF as the solvent (Table 1, entry 1). The sacrificial reductant for the reaction is the Hantzsch ester,^34^ which is also required by the catalytic turnover of the reaction with nickel, as demonstrated by DFT calculations recently performed by our group.^33b^ The reduction to 1.1 or 1.5 equiv of this reagent leads to a decrease in the reaction yield (Table 1, entries 3 and 4). The organic dyes 4CzIPN^35^ and 3DPAFIPN^36^ were both found to be suitable photocatalysts for the reaction (Table 1, entry 5). We chose 3DPAFIPN because of the slightly better yields obtained (>99% compared to 95%). The presence of the photocatalyst, irradiation with visible light (blue LED, 456 nm, 40 W from a Kessil Lamp), and nickel was essential to observe the desired reaction (Table 1, entries 6–8). We chose THF as the optimal reaction solvent, but it is possible to carry out the reaction in other solvents (Table 1, entries 9 and 10) with a minimal reduction in yield. Phenanthroline as a ligand for nickel was also advantageous for the transformation (Table 1, entry 11), controlling both the yield and the diastereoselectivity of the reaction. The amount of nickel catalyst can be reduced to 5 mol % (in combination with 7.5 mol % L1) while maintaining a high conversion in a reaction time of 16 h (Table 1, entry 12). The use of DPPE and DPPP as ligands completely inhibited the reaction (Table 1, entries 13 and 14). Low yields were observed with a more rigid BINAP ligand (Table 1, entry 15).^37^ Reactivity is restored with the commercially available nickel phosphine complex NiCl_2_(PBu_3_)_2_ (Table 1, entry 16). Various substituted phenanthrolines have been tested in reactions, with results that are either lower or comparable to those obtained with unsubstituted phenanthroline (Table 1, entries 17–20). Ni(acac)_2_ and Ni(OAc)_2_ can serve as effective precursors for the formation of nickel complexes with phenanthroline (Table 1, entries 21–22). Other metals tested in the reaction such as CrCl_3_,^38^ Cp_2_TiCl_2_,^32a^ and CoBr_2_^30a,30b^ were found to be completely unreactive with MBH acetate (Table 1, entries 23–25). For the MBH adducts, it was possible to use an unprotected alcohol for the reaction, but the yields were significantly reduced, while carbonates of the MBH adduct gave similar reactivities (Table 1, entries 26–28).

**Table 1:**
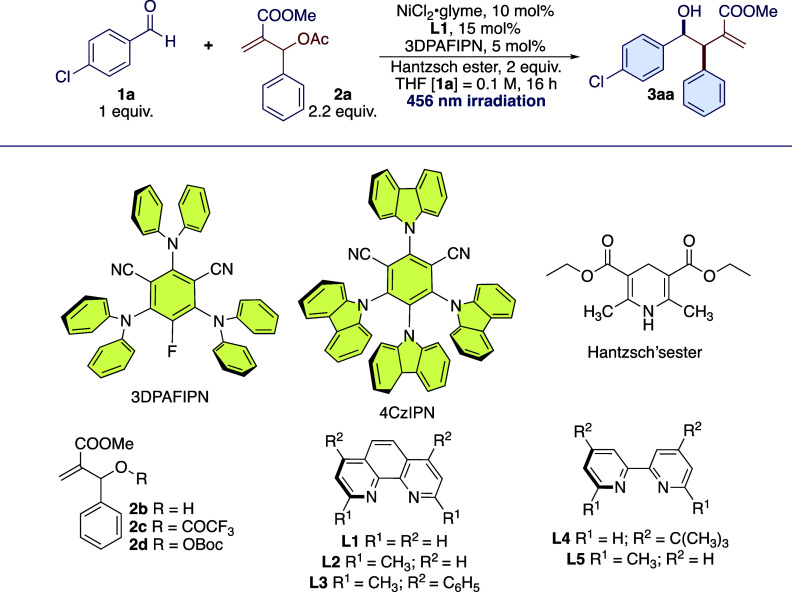
Optimization of the Nickel-Promoted Photoredox Reaction with the MBH Adducts

Entry^a^	Deviations from standard conditions	Yield (%)^b^	*dr*^c^
1	none	>99(87)^d^	>95:5
2^e^	none	>99(80)^d^	>95:5
3	1.1 equiv of Hantzsch ester	86	>95:5
4	1.5 equiv of Hantzsch ester	86	>95:5
5	4CzIPN instead of 3DPAFIPN	95	>95:5
6	no photocatalyst	no reaction	–
7	no light	no reaction	–
8	no NiCl_2_·glyme	no reaction	–
9	DMF instead of THF	97	90:10
10	toluene instead of THF	90	93:7
11^f^	No **L1**	36	94:6
12^g^	5 mol % of NiCl_2_·glyme	>99	95:5
13^f^	DPPE instead of **L1**	traces	–
14^f^	DPPP instead of **L1**	traces	–
15^f^	(±)–BINAP instead of **L1**	28	66:34
16	NiCl_2_(PBu_3_)_2_ instead of **L1**+ NiCl_2_·glyme	82	>95:5
17	**L2** instead of **L1**	73	80:20
18	**L3** instead of **L1**	50	94:6
19	**L4** instead of **L1**	95	>95:5
20	**L5** instead of **L1**	56	80:20
21^h^	Ni(acac)_2_ instead of NiCl_2_·glyme	95	86:14
22^h^	Ni(OAc)_2_ instead of NiCl_2_·glyme	>99	>95:5
23^f^	Cp_2_TiCl_2_ instead of **L1**+ NiCl_2_·glyme	no reaction	–
24^f^	CrCl_3_ instead of **L1**+ NiCl_2_·glyme	no reaction	
25	CoBr_2_ instead of NiCl_2_·glyme	no reaction	
26^f^	**2b** instead of **2a**	45%	66:34
27^h^	**2c** instead of **2a**	18%	83:17
28^h^	**2d** instead of **2a**	95%	–

a Reactions performed on 0.1 or 0.2 mmol scale.

b Determined by ^1^H NMR analysis.

c Determined by the integration of two benzylic CH ^1^H NMR signal on the reaction crude.

d Isolated yield after chromatographic purification.

e Reaction performed on 2 mmol of **1a**. Reaction conditions: NiCl_2_·glyme 5 mol %, **L1** 7.5 mol %, **3DPAFIPN** 3 mol %, **HE** 200 mol %, THF [**1a**] = 0.1 M, and irradiation time 60 h.

f Pinacolization of **1a** detected as main byproduct *dl*/*meso* 1:1.

g 7.5 mol % of **L1** employed.

h 5 h irradiation time. DPPP = 1,3-bis(diphenylphosphino)propane; DPPE = 1,2-bis(diphenylphosphino)ethane; (±)-BINAP = (±)-2,2′-bis(diphenylphosphino)-1,1′-binaphthalene.

**Scheme 1:**
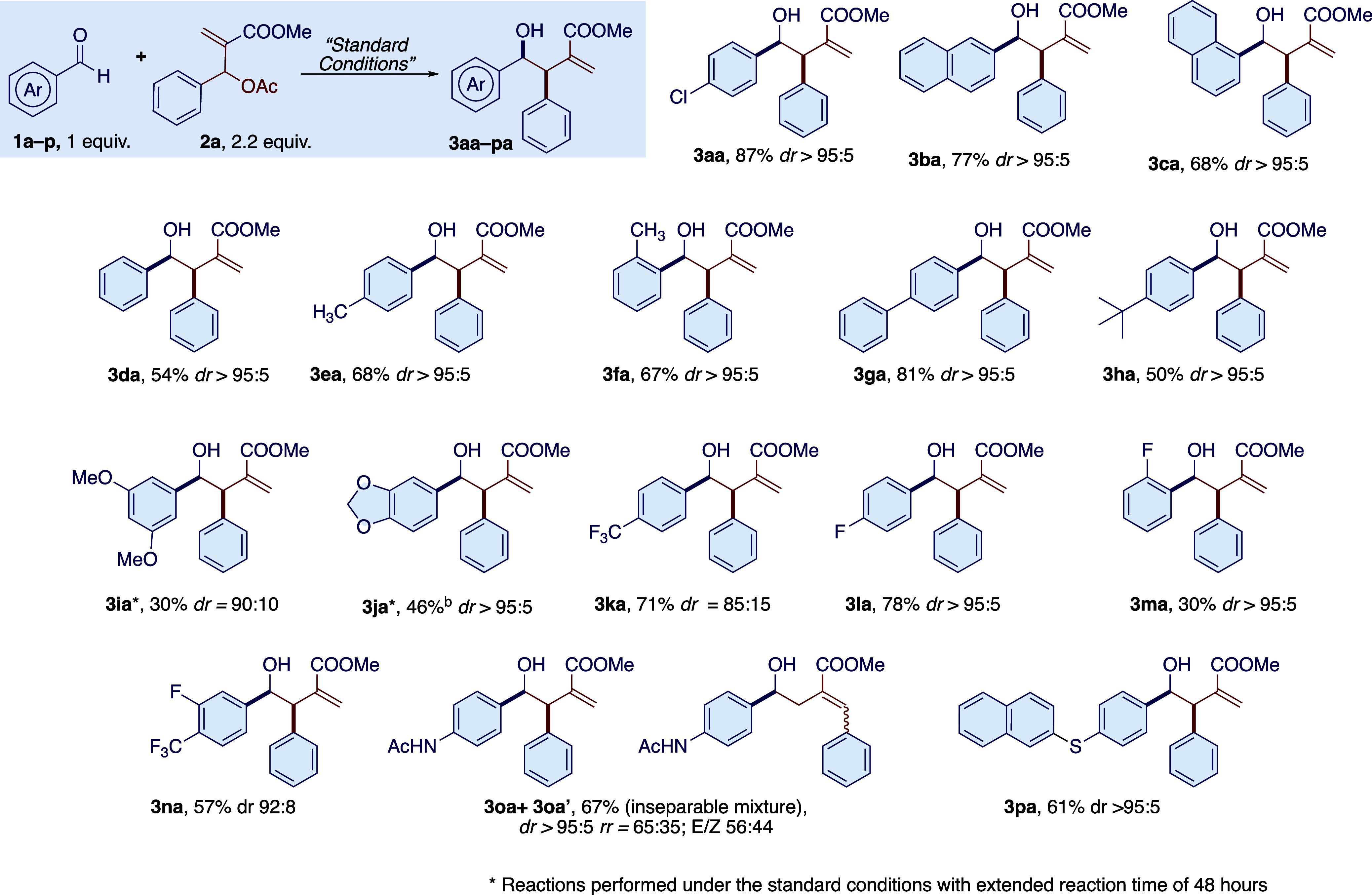
Photoredox Nickel-Promoted Reaction of MBH Adduct **2a** with Aromatic Aldehydes

**Scheme 2:**
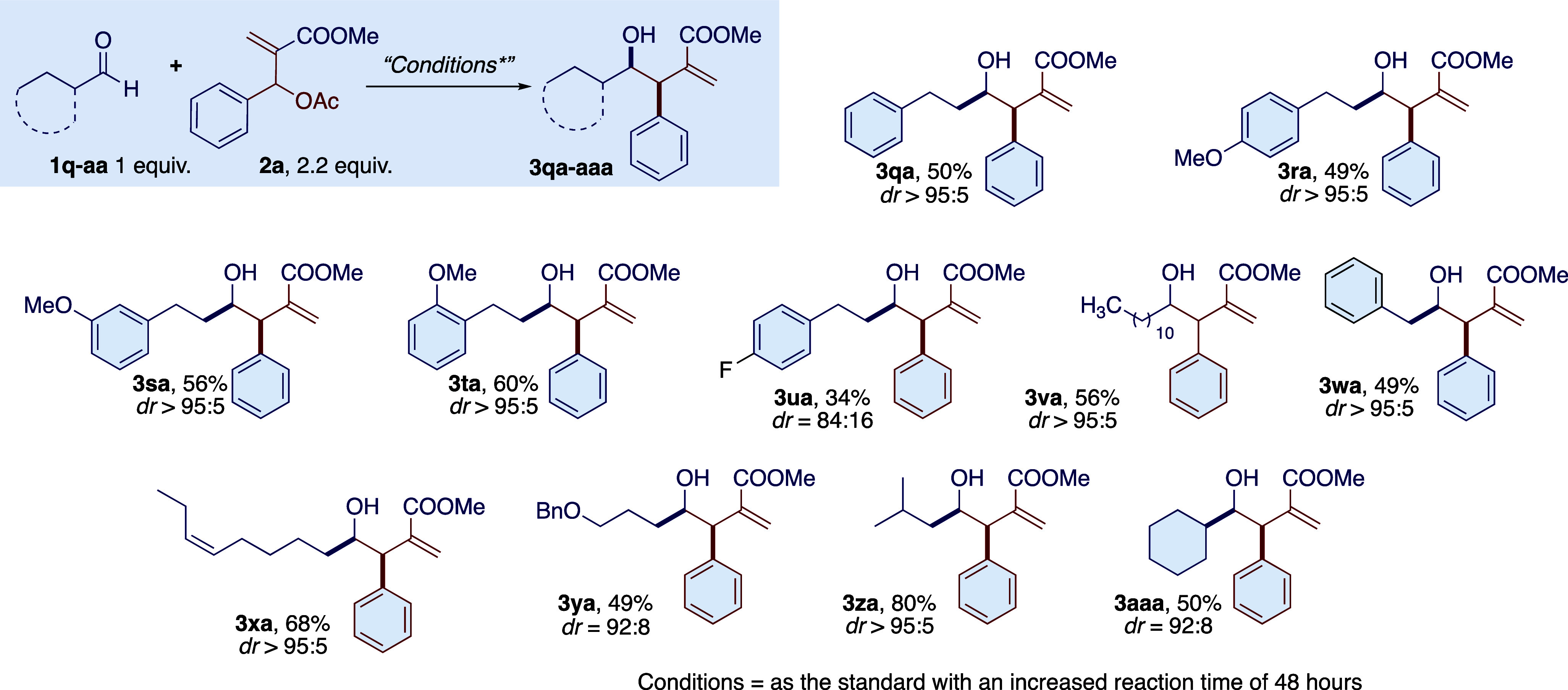
Photoredox Nickel-Promoted Reaction of MBH Adduct **2a** with Aliphatic Aldehydes

**Scheme 3:**
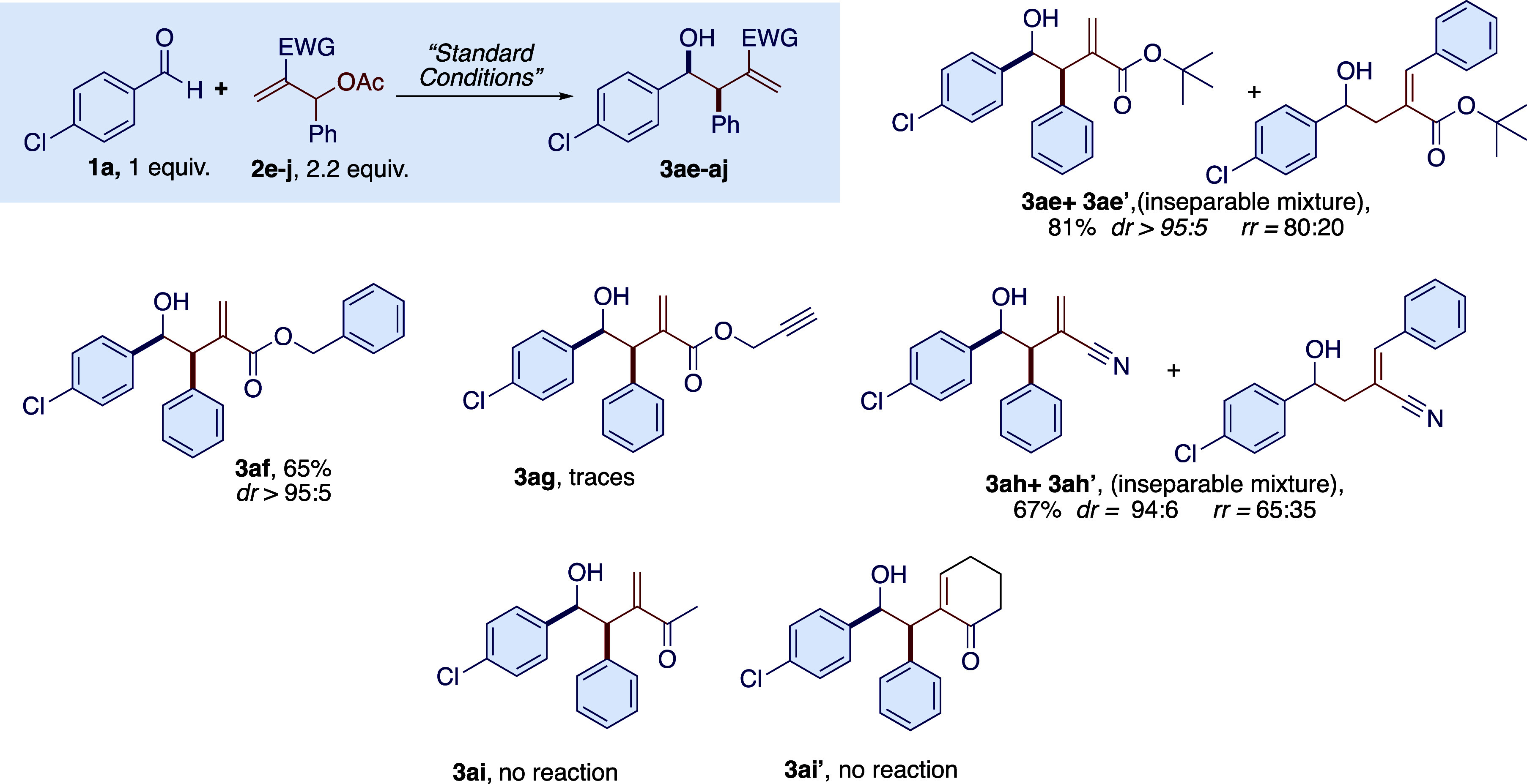
Photoredox Nickel-Promoted Reaction of MBH Adducts Carrying Different EWG Groups in the Reaction with 4-Chloroaldehyde **1a**

**Scheme 4:**
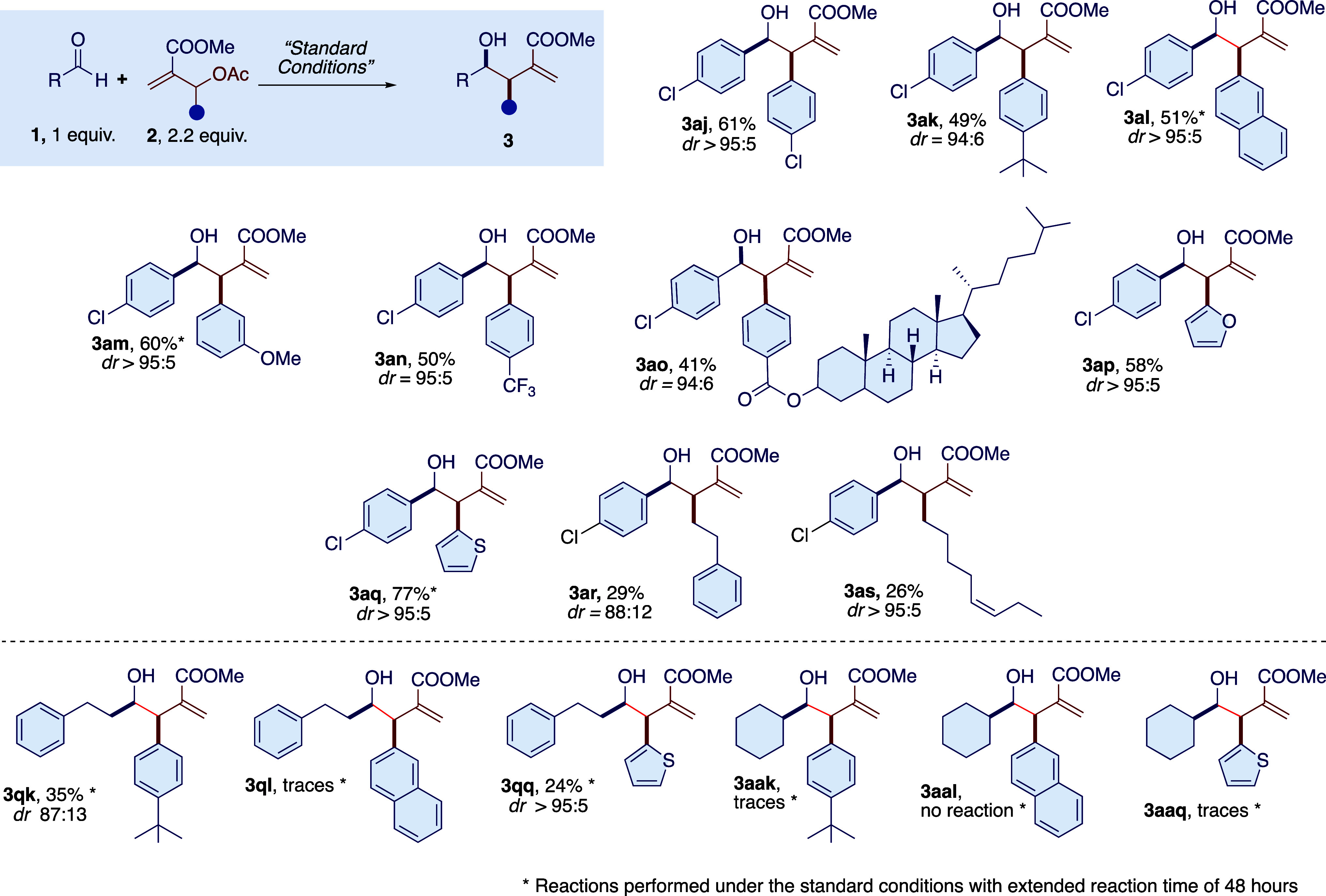
Photoredox Nickel-Promoted Reaction of Various MBH Adducts with Aromatic Aldehydes and Aliphatic Aldehydes

With the optimized reaction conditions in hand, we have investigated the reaction by first varying the aldehyde partner and then exploring a wide range of substituted MBH adducts. The main results are shown in Schemes 1–4. Various substituted aromatic aldehydes were found to be reactive under the optimized reaction conditions (Scheme 1), and in all the cases, the relative configuration of the detected major diastereoisomer was assigned to be *syn* by analogy with the reported data^38^ for **3da**. Aromatic aldehydes substituted with electron-withdrawing groups showed improved reactivity compared to electron-rich aldehydes. The diastereoselectivity was quite high, with a majority of 90:10 in most of the cases in favor of the *syn* diastereoisomer. The predominant formation of this diastereoisomer was explained by performing detailed DFT studies on the reaction profile (see below). Furthermore, the reaction was selective for the formation of the branched isomer with complete regioselectivity in almost all the cases studied. In a few cases, a low amount (<15%, compared to the product obtained) of the linear product was detected in the crude reaction mixture, but after chromatographic purification, the linear product was never detected. The purification of the product is sometimes complicated by the formation of byproducts due to the decomposition of the MBH adduct (e.g., reduction of the double bond, deacetylation, etc.) and of the unreacted MBH. The isolated yields are lower compared to the aldehyde conversion due to the unavoidable pinacol coupling that accompanies the photoredox catalysis in the case of aromatic aldehydes. The photocatalyst, in this case 3DPAFIPN, can promote the nonselective pinacol coupling, in the presence of HEH^+^ or HE^•+^ (HE = Hantzsch’s ester), which act as a Brønsted acid and activate the aromatic aldehydes through their reduction to ketyl radicals.^39^ Other functionalized aromatic aldehydes tested, (4-(phenylethynyl)benzaldehyde, 4-pyridinecarboxaldehyde, 3-indolecarbaldehyde), were found to be unreactive or to give a complex mixture of uncharacterized products. In the case of methyl 4-formylbenzoate, the aldehyde conversion was complete but toward the uncharacterized products and pinacol coupling (dr = 1:1). In the case of unprotected 4-hydroxybenzaldehyde, ^1^H NMR of the crude reaction mixture showed the presence of many byproducts like those observed for the aldehydes mentioned above. In contrast with the previous examples, for this aldehyde, we observed the presence of the desired product in an estimated amount of 30%, which, however, could not be isolated by column chromatography. However, the reaction was tolerant of acetamido and naphythyl thioether functional groups. In the case of 4-acetamidobenzaldehyde **3o**, the product was isolated in 75% yield as an inseparable mixture of branched and linear regioisomers (65:35). For the branched product **3oa**, the high diastereoselection typical for this reaction was observed (*dr* > 95:5). In the linear stereoisomer **3oa’**, we observed, by ^1^H NMR, the presence of *E* and *Z* (*E/Z* = 56:44) configurations at the substituted double bond. The presence of an increased amount of linear product was attributed to the presence of the AcNH in the para position, which is likely to reduce the difference in selectivity-determining energy barriers, favoring the formation of the thermodynamic linear product. The reaction with 4-(naphthalen-2-ylthio)benzaldehyde **3p** gave an isolated yield of 61% with a dr of >20:1 for the product **3pa**. In this case, no traces of the linear isomer were detected.

For aliphatic aldehydes, we also found positive results, briefly illustrated in Scheme 2. We also observed a high diastereoselectivity with aliphatic aldehydes, exceeding 90:10 in most of the cases studied. Branched and linear aliphatic aldehydes were found to be reactive in the standard reaction conditions, but it was necessary to increase the reaction time from 16 to 48 h. In these cases, reduced yields were only due to the decomposition of the MBH adduct, as pinacol coupling of the aldehydes was never detected. Instead, aliphatic aldehydes gave aldol byproducts (5–15%) in the presence of the strong Brønsted acid formed upon the oxidation of the HE ester (HEH^+^ pyridine). The presence of some functional groups such as double bonds and protected alcohols was tolerated.

Finally, we also varied the MBH adducts, taking advantage of their simple and inexpensive preparation reported in the literature (Schemes 3 and 4, see Supporting Information for details). The effect of the EWG group of the MBH adducts on the reaction outcome was evaluated. Changing the ester substituent from methyl to *tert*-butyl (**2e**) or benzyl (**2f**) does not inhibit the reaction, and the desired product is obtained with good diastereomeric ratios; however, increasing the steric hindrance of the ester moiety resulted in a significant amount of the linear isomer (**3ae’**). However, the MBH adduct derived from propargyl acrylate (**2g**, Scheme 3) was found to be incompatible with the reaction conditions, and only traces of products were observed in the crude reaction mixture. On the other hand, the transition from the ester group to other EWG groups resulted in reduced reactivity. The MBH adduct derived from acrylonitrile (**2h**) allows the reaction, but the presence of a significant amount of linear isomer was detected (**3ah’**). On the other hand, MBH acetates derived from methyl vinyl ketone (**2i**) and cyclohexanone (**2i’**) were completely unreactive under the optimized conditions and for prolonged reaction times. In contrast, various MBH adducts bearing substituted aromatic, heteroaromatic, and aliphatic groups (Scheme 4, **2j**–**s**) were well tolerated with the model *p*-chlorobenzaldehyde (**1a**). In the case of MBH adducts (**2k**), (**2l**) and (**2q**) we also tested linear (**1q**) and branched (**1aa**) aliphatic aldehydes and found a modest reactivity only in favor of aliphatic aldehydes (35% yield for **3qk** and 24% yield for **3qq**), while the reaction with cyclohexanecarbaldehyde **3aa** did not give the desired product. The 2-naphthylaldehyde MBH derivative (**2l**), on the other hand, was found to be quite unreactive even with a linear aliphatic aldehyde after 48 h of irradiation. In all cases, the diastereoselection was again quite high, favoring the *syn*-diastereoisomer, with a dr of >95:5 in the case of **3qq** and 87:13 for **3qk**. In the case of the Morita–Baylis–Hillman adduct substituted with an aliphatic chain (**2r** and **2s**), we also found positive results. However, the introduction of an aliphatic chain into the MBH adduct significantly reduced its reactivity with the model aldehyde **1a**, and although the diastereoselection was good to high, the reaction yields were modest. Unfortunately, the reactivity of the allylic nickel intermediate obtained under photoredox conditions was low with other aromatic and aliphatic hindered aldehydes, as illustrated and commented on in the Supporting Information, and a direct comparison between the results obtained by inserting different aromatic and aliphatic moieties into the MBH adducts showed quite a difference in reactivity.

**Scheme 5:**
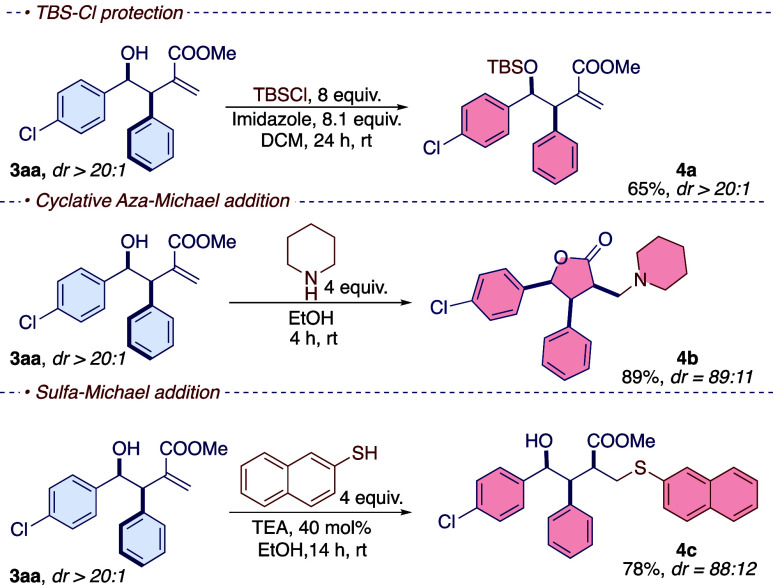
Selective Chemical Transformations Performed on the Product **3aa**

With the final isolated adducts, the possibility of further functionalization of the prepared molecules was tested, as shown in Scheme 5. The free alcohol moiety was easily protected as *tert*-butyl-dimethylsilyl ether under standard conditions, allowing the isolation of the product **4a** as a single *syn*-diastereoisomer (dr > 20:1). The protection of the alcohol does not change the diastereoisomeric ratio, but it allows for the separation of the two diastereoisomers. The isolated adducts are compelling substrates for Michael reactions.^1,2^ However, in Michael reactions, the presence of basic conditions promotes the formation of enolate, resulting in a slight equilibration of the two distereoisomers, and in two cases studied, an erosion of the diastereoisomeric ratio was observed. In both cases, the MBH adduct reacted to give the desired product in high yields due to the good reactivity of the MBH adduct in Michael-type reactions.^40^

## Mechanistic Details

After the potential reported methodology in terms of applicability and tolerance to different functional groups was demonstrated, a combination of experimental studies, photophysical studies, and DFT calculations was performed to fully examine the reaction mechanism.

**Scheme 6:**
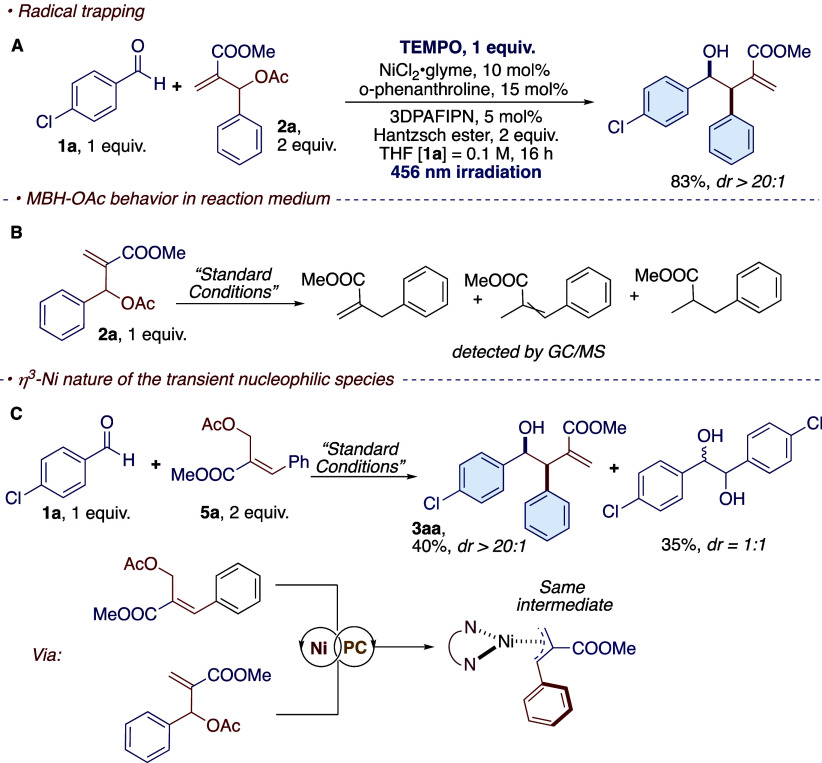
Experimental Mechanistic Investigations: (A) Radical Trapping with TEMPO, (B) Reactivity of MBH Adduct in Absence of Aldehydes, and (C) Reaction with an Isomer of the MBH Adduct Forming the Same Nickel Intermediate

The model reaction proceeds unchanged in the presence of 1 equiv. of TEMPO as a radical trap (Scheme 6A). As discussed, the isolation of the desired target materials is complicated by the presence of byproducts derived from the MBH adduct. When the MBH acetate **2a** was subjected to the reaction conditions in the absence of aldehyde, we were able to confirm the formation of the regio- and stereoisomer reduced products (due to L_*n*_NiH formation in the absence of aldehyde) and the fully hydrogenated reduced product (Scheme 6B).

Finally, the origin of the regio- and diastereoselectivity was investigated. According to our hypothesis, the key intermediate leading to the observed diastereo- and regioselectivity in all cases depends on the formation of an η^3^ intermediate of the nickel complex after the oxidative addition in the presence of the substrate **2a**. Based on this premise, the structural isomer of substrate **2a** (**5a**) was synthesized, which would generate the same η^3^ intermediate of **2a** and would lead to the same product obtained under standard conditions. Based on this premise, the structural isomer of substrate **2a** (**5a**) was synthesized, which would generate the same η^3^-intermediate of **2a** and lead to the same product obtained under standard conditions. The use of **5a** (refer to the Supporting Information for its synthesis) as the substrate resulted in the formation of the desired product **3aa** with the same diastereo- and regioselective outcome (Scheme 6C). However, the yield is significantly influenced by the presence of the pinacol product, as a 1:1 mixture of diastereoisomers is derived from the reductive dimerization of the aldehyde. The reason for this discrepancy may be that in substrate **2a**, the oxidative addition occurs at the benzylic position, which contributes positively to the stabilization of the intermediate (see below), whereas in the case of **5a**, the oxidative addition occurs at an sp^3^ carbon, which does not provide sufficient stabilization and thus leads to a higher energy barrier.

### Photophysical Investigation

**Figure 2:**
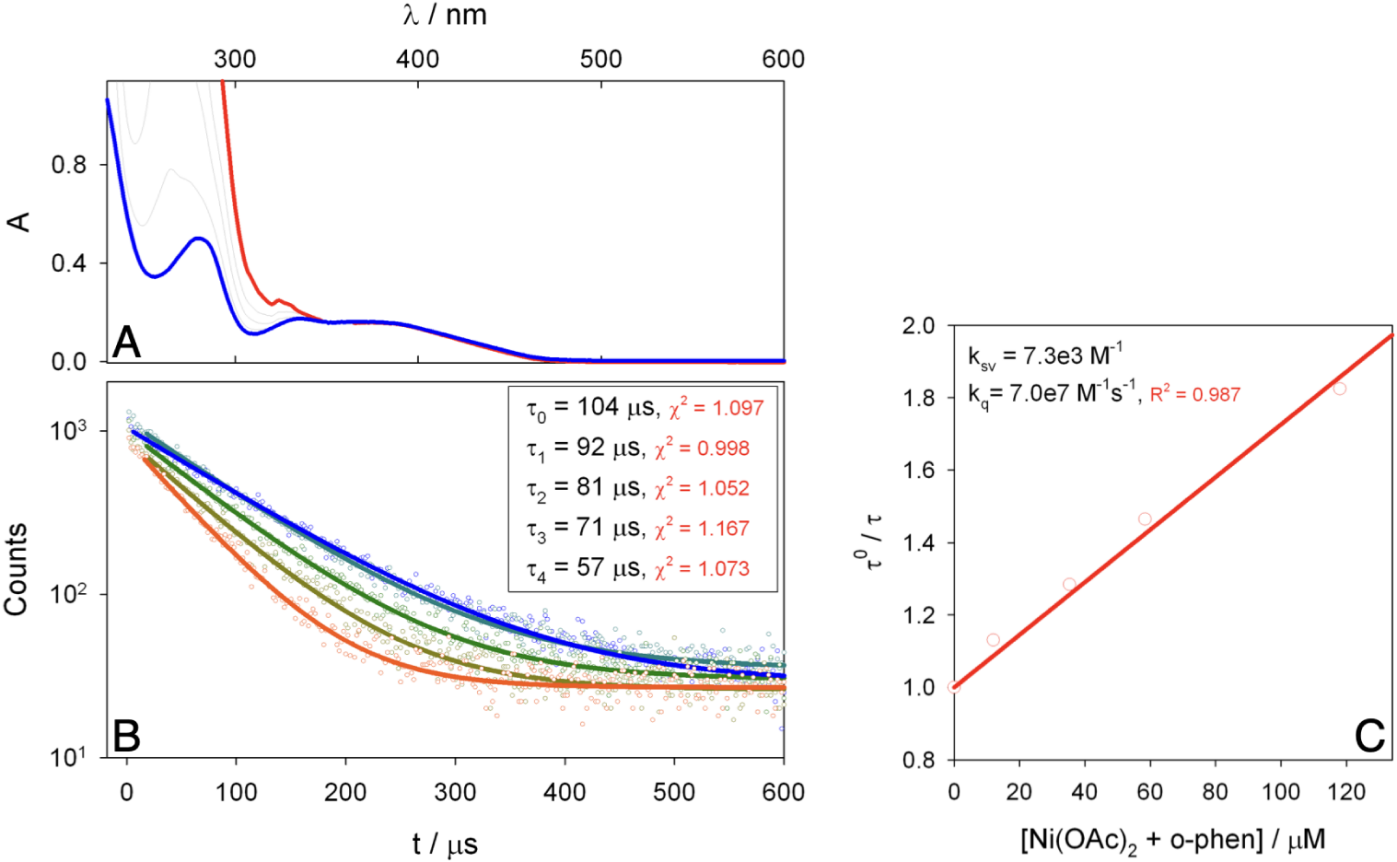
(A) Absorption spectra of solutions of **3DPAFIPN** in degassed THF at r.t. (ca. 11 μM, blue line) obtained upon the addition of increasing amounts of the complex between *o*-phenathroline and Ni(OAc)_2_ (1.5:1.0 molar ratio in THF; up to ca. 0.12 mM of Ni(OAc)_2_). (B) Delayed fluorescence decays of **3DPAFIPN** obtained from the same solutions at λ_em_= 530 nm (λ_ex_ = 475 nm) and the corresponding monoexponential fitting functions. (C) Stern–Volmer diagram relative to the determined decay lifetimes.

To elucidate the mechanism of the presented dual nickel-photoredox methodology and clarify the role of visible light irradiation as the trigger for the reported transformation, a comprehensive photophysical characterization of the photocatalyst’s behavior in the presence of various reaction partners has been performed. Although the optimization study revealed that 4CzIPN leads to nearly identical results, the protocol was developed using 3DPAFIPN as the photocatalyst responsible for driving the process. 3DPAFIPN was selected as the photocatalyst due to its favorable photophysical and redox characteristics.^32d,33b^ As previously reported, in a deaerated THF solution, it exhibits an absorption band in the visible range (ε = 14,900 M^–1^ cm^–1^ at λ = 370 nm, with an absorption onset at 470 nm in THF, Figure 2A) and an emission band centered at 520 nm, attributed to prompt fluorescence (τ = 3.6 ns) and delayed fluorescence (τ = 110 μs).^32d,33b^ Additionally, it is worth noting that 3DPAFIPN has been previously reported to be quenched by the Hantzsch’s ester (HE) via a static quenching mechanism, i.e., the formation of a ground state complex referred to as 3DPAFIPN·HE. Under the present experimental conditions, in which the HE concentration is about 0.2 M, close to its solubility limit in THF, 3DPAFIPN forms the 3DPAFIPN·HE complex in near-quantitative amounts. This interaction and the photophysical behavior of 3DPAFIPN·HE have been previously demonstrated through a study combining photophysical experiments with computational modeling.^33b^ In addition, it was demonstrated that 3DPAFIPN is not significantly quenched by aldehyde **1a** (*k*_q_ < 1.0 × 10^6^ M^–1^ s^–1^).^32d,33b^ To fully trace the catalytic role of the photosensitizer, quenching analyses of the luminescence were carried out, considering the other reaction partners in the presented protocol. In line with our previous reports, the pronucleophilic species **2a** and the model ligand **L1** alone do not quench the fluorescent excited state of the photocatalyst, even when working at concentrations comparable to or exceeding those used under the reaction conditions (see Supporting Information for details). The *in situ*-formed complex between Ni(OAc)_2_ and phenanthroline was then considered as a potential quencher (see Supporting Information for details and Figure 2A,B). It is important to highlight that, in contrast to standard conditions, the precatalyst Ni(OAc)_2_ was employed in the analysis, as it ensures the same outcome (see Table 1, entry 22) and enhances the solubility of the resulting complex. In this context, a quenching constant *k*_q_ = 7.0 × 10^7^ M^–1^ s^–1^ was measured (Figure 2C). Given the higher concentration of HE (0.2 M) under the reaction conditions compared to that of the Ni(II) complex (0.01 M), the quenching efficiency is higher for the former compared to the latter (98% and 2%, respectively; see Supporting Information for details). Therefore, we can conclude that the quenching observed with HE is the most significant quenching process of the luminescent excited state of 3DPAFIPN.

**Scheme 7:**
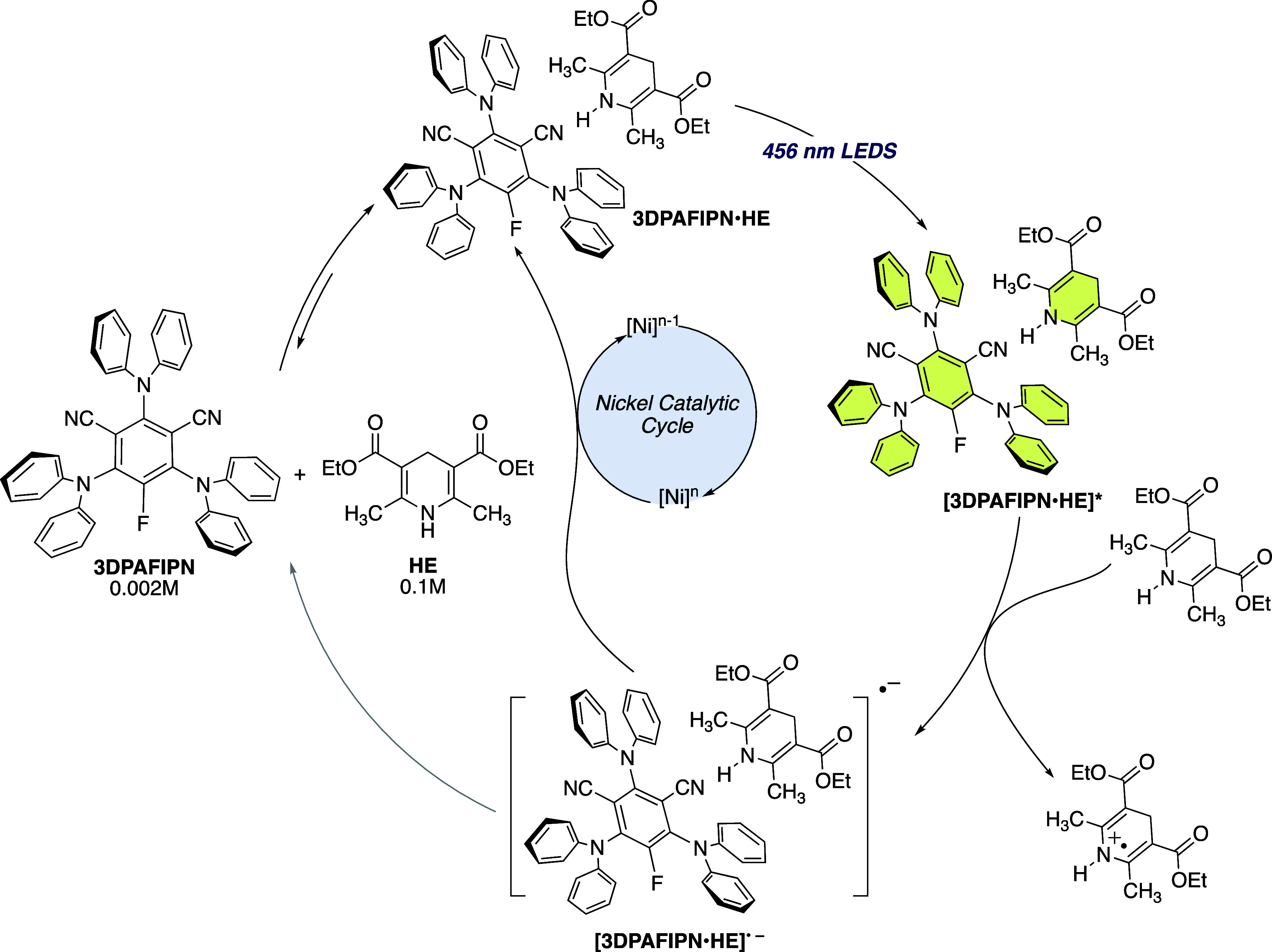
Proposed Photocatalytic Cycle

Considering the data obtained, we propose a photocatalytic cycle, as shown in Scheme 7. The static quenching between 3DPAFIPN and HE leads to the formation of the corresponding [3DPAFIPN·HE]^•–^. This species is a powerful reductant (*E*_red_ = −1.63 V vs SCE)^33b^ and can reduce the nickel complex, thereby triggering the desired transformation while simultaneously regenerating the pristine photoactive species (see below).^29b,38^

### DFT Calculations

**Figure 3:**
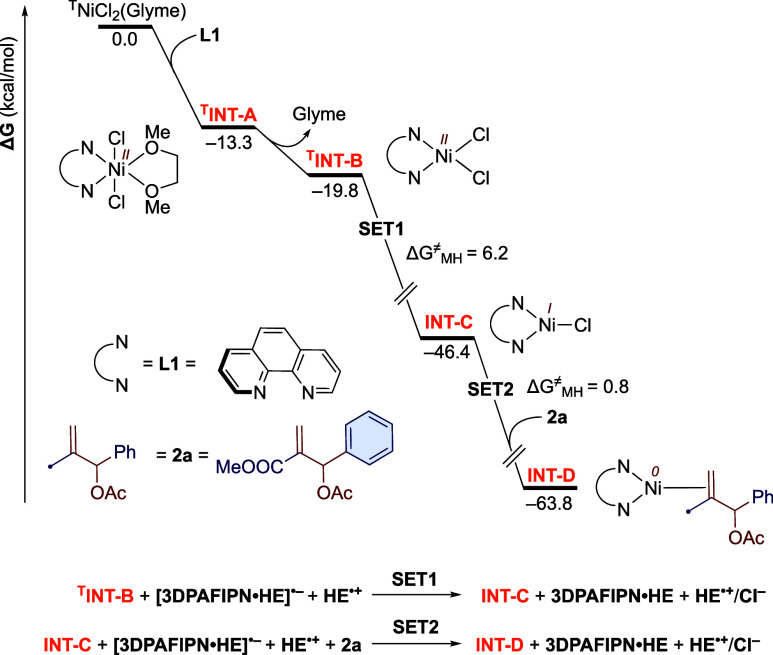
Free energy profile for the formation of Ni(0) species **INT-D** at the M06(SMD,THF)/def2-TZVPP//PBE-D3(SMD,THF)/def2-TZVP(Ni)/def2-SVP level of theory.

Density functional theory (DFT) calculations were performed to unfold the reaction mechanism of the photoredox- and nickel-catalyzed allylation of aldehydes with MBH adducts. Hereafter, the letter as the apex before the species denotes the spin multiplicity: for example, T stands for the triplet state. The reaction starts with the coordination of **L1** to the nickel precatalyst ^**T**^**NiCl**_**2**_**(Glyme)**, resulting in the appreciably stable octahedral Ni(II) species ^**T**^**INT-A**, from which the exergonic liberation of glyme yields ^**T**^**INT-B** (Figure 3). Then, ^**T**^**INT-B** gets reduced to **INT-C** by **[3DPAFIPN·HE]**^**•–**^ (SET1) following reductive quenching within the photoredox catalytic cycle. This step is exergonic by 26.6 kcal/mol with an energy barrier of 6.2 kcal/mol, estimated using the Marcus–Hush theory (Table S1).^41^ This is in line with our previous finding, which showed that the associated species **3DPAFIPN·HE** plays the role of photocatalyst in the reaction.^33b^ Moreover, a 20-fold higher concentration of **HE** compared to ^**T**^**NiCl**_**2**_**(Glyme)** under the experimental conditions triggers a preference for the reductive quenching of the photoexcited ^**T**^**3DPAFIPN·HE** over oxidative quenching.^33b,42^ The oxidative addition of allyl acetate (**2a**) to the Ni(I) intermediate **INT-C**, leading to a significantly less stable Ni(III) intermediate, demands an unattainable energy barrier of 35.0 kcal/mol and therefore can be ruled out (Figure S9). Rather, further reduction of **INT-C** by single-electron transfer (SET2) from **3DPAFIPN·HE**^**•–**^ with concurrent coordination of **2a** results in an appreciably stable Ni(0) species **INT-D**, which acts as the active catalyst in the nickel catalytic cycle (Figure 3). Moreover, generation of **INT-D** from **INT-C** via the disproportionation route is thermodynamically unfavorable by 9.2 kcal/mol and hence can be discarded (Figure S1-).

**Figure 4:**
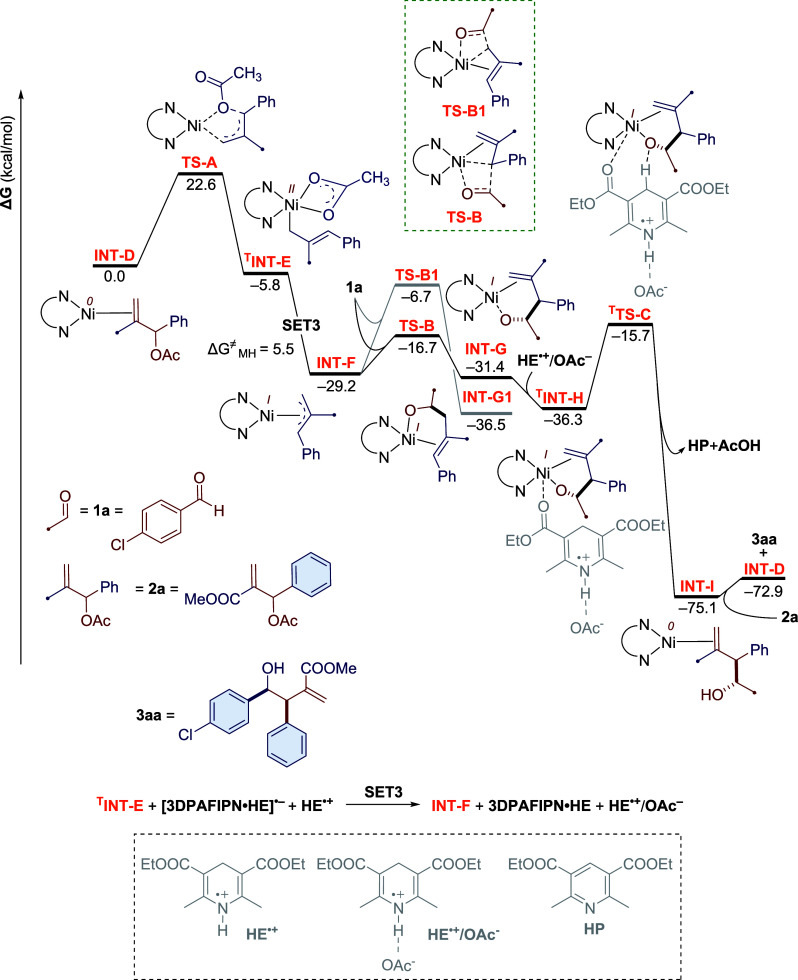
Free energy profile for the nickel catalytic cycle of allylation of aldehydes. For energy conventions, refer Figure 3.

The nickel catalytic cycle begins with the oxidative addition of coordinated allyl acetate in **INT-D** via the transition state **TS-A** (Figure 4). This step needs to surmount an energy barrier of 22.6 kcal/mol. The facile single-electron reduction of the resulting intermediate ^**T**^**INT-E** by **3DPAFIPN·HE**^**•–**^ (SET3) and an estimated energy barrier of 5.5 kcal/mol furnish the Ni(I) species **INT-F**. The subsequent insertion of *p*-chlorobenzaldehyde (**1a**) into the Ni-allyl bond in **INT-F** from its more hindered site leads to a slightly more stable intermediate **INT-G** via the transition state **TS-B**. The insertion step needs to overcome an energy barrier of 12.5 kcal/mol. Besides, similar carbonyl insertion from the less hindered site of **INT-F** via transition state **TS-B1** requires a noticeably higher energy barrier of 22.5 kcal/mol, explaining the regioselectivity and diastereoselectivity of the reaction. The reaction further continues with the coordination of **HE**^**•+**^**/AcO**^**–**^ to **INT-G**, leading to a notably stable intermediate ^**T**^**INT-H** that undergoes intramolecular hydrogen atom transfer (HAT) involving the transition state ^**T**^**TS-C** to deliver a remarkably stable intermediate **INT-I** with the release of Hantzsch pyridine (**HP**) and acetic acid.^30b^ Finally, another molecule of **2a** replaces the desired product (**3aa**) already formed in **INT-I** to regenerate **INT-D** for the next catalytic cycle. In conclusion, the oxidative addition step turns out to be the rate-limiting step of the reaction, while the carbonyl insertion step is responsible for the regioselectivity and diastereoselectivity.

**Figure 5:**
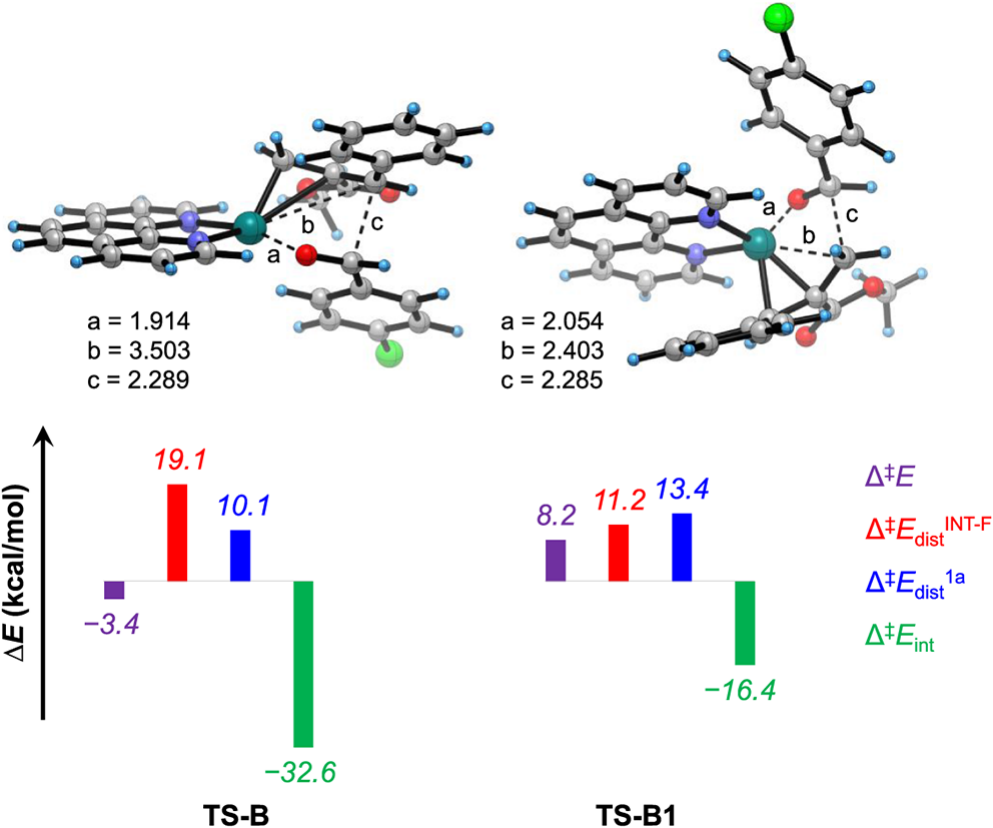
Distortion–interaction analysis of **TS-B** and **TS-B1**. Optimized geometries of the transition states are determined with selected bond distances in angstroms (Å).

To cast light on the origin of activation barriers associated with the regio- and diastereoselectivity-determining steps, distortion–interaction analysis^43^ was performed on the competitive transition states **TS-B** and **TS-B1**, considering **INT-F** and **1a** as the interacting fragments (Figure 5). The remarkably lower activation barrier for **TS-B** than **TS-B1** is attributed to the appreciably higher interaction energy (in absolute values) between the two distorted fragments in **TS-B**, which counterbalances the higher distortion energy of the Ni fragment of **TS-B**, reflected in the stretching of the Ni-allyl bond (3.503/2.403 Å in **TS-B**/**TS-B1**) participating in aldehyde insertion. Besides, the distortion energy of the aldehyde fragment is slightly lower in **TS-B** in comparison with **TS-B1**.

**Scheme 8:**
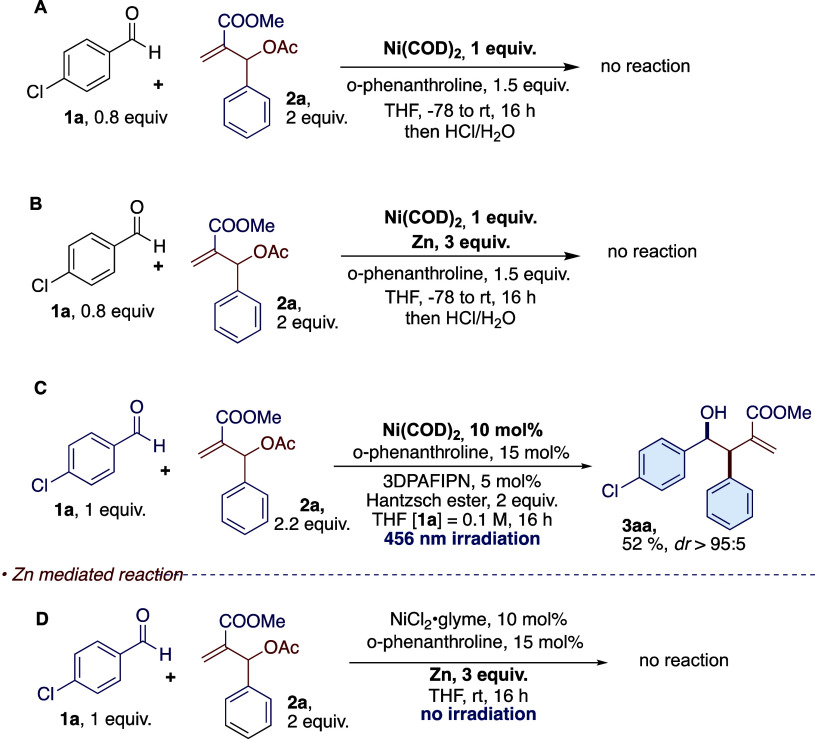
Mechanistic Investigations with Ni(COD)_2_: (A) Use of Ni(COD)_2_ in the Absence of Light Irradiation and Hantzsch Ester; (B) Use of Ni(COD)_2_ in the Presence of Zn Metal as the Reductant; (C) Employment of Ni(COD)_2_ in Catalytic Amount under Photoredox Conditions; and (D) Use of NiCl_2_Glyme in Catalytic Amount, in the Presence of Phenanthroline as the Ligand and using Zn Metal as the Reductant without Irradiation with Visible Light

Recently, a highly enantioselective photoredox acyl C–H allylation of aldehydes and formamides, which proceeded under the cooperative catalysis of a chiral phosphinooxazolines (PHOX) nickel complex and tetrabutylammonium decatungstate (TBADT), was reported.^44^ It is remarkable that Morita–Baylis–Hillman adducts behaved as the electrophilic allylating agent. However, for the proposed mechanism, the authors reported the oxidative addition of the MBH adduct to Ni(0) to form an allyl Ni(II) complex. It has been reported that the cationic η^3^ allylnickel(II) complex, prepared in situ from [Ni(COD)_2_] and allyl chloride in the presence of bipyridine and ammonium hexafluorophosphate, was able to allylate aromatic aldehydes. The reactivity was enhanced when the reaction was carried out in the presence of zinc. The zinc is responsible for the reduction of the allylnickel(II) to form the allylnickel(I) complex.^45^ We prepared Ni(COD)_2_ according to the procedure described by Percec^46^ (see Supporting Information for details) and used Ni(COD)_2_ for a reaction with 1 equiv of **1a**, 1 equiv of **2a**, and 1 equiv of phenanthroline as a model stoichiometric reaction in the absence and in the presence of Zn^45^ (Scheme 8A,B). After 16 h at room temperature, no formation of the product was observed.

We also used Ni(COD)_2_ as a precatalyst under photoredox conditions (Scheme 8C) and the reaction gave the products with reduced yield compared to the catalytic reaction with NiCl_2_-glyme (52% vs 87%) and the same diastereoselectivity. This result showed that under photoredox conditions, Ni(COD)_2_ was able to promote the formation of the allylating species. We checked if nickel catalysis with Zn and no irradiation could promote the reaction (Scheme 8D), but we observed no reactivity, indicating the peculiar conditions of photoredox catalysis. Furthermore, to confirm that the oxidative addition is the rate-limiting, we performed three experiments over 3 h, varying the concentration of **2a** (0.5, 1, and 2 equiv). The conversion of the product was evaluated by ^1^H NMR with an internal standard. The yields of **3aa** evaluated by internal standard were 2%, 8%, and 16%, respectively, confirming the increased rate with higher concentrations of Baylis–Hillman adduct.

## Conclusions

In conclusion, we have used MBH adducts under photoredox conditions to access nucleophilic allylating reagents. These reagents were reactive with aromatic and aliphatic aldehydes, affording the corresponding decorated homoallylic alcohols with good diastereomeric control. The mechanism of the reaction was investigated by photophysical analysis and DFT calculations. The oxidative addition of MBH acetate to Ni(0) is identified as the rate-limiting step, while the aldehyde insertion into Ni(I)-allyl is predicted to be the origin of the regio- and diastereoselectivity. The photophysical study and DFT calculations highlight the role of the Hantzsch ester in the photoredox reactions with nucleophilic organometallic reagents for the turnover of the nickel catalyst. Attempts to use suitable chiral ligands for the reaction gave only modest results, probably due the hindrance imposed by the transition state. Efforts to design better chiral ligands are currently under investigation in our laboratory.

## Supporting Information Available

Photochemical apparatus used and conditions for the setup of the reaction, complete characterization of the isolated product with spectra (^1^H NMR and ^13^C), photophysical studies on the reactions and Stern Volmer plot, computational details, energies, and Cartesian coordinates of optimized structures (PDF)

## Abbreviations

MBH: Morita–Baylis–Hilmann
HE: Hantzsch ester
HEH^+^: oxidated protonated Hantzsch ester
HE^•+^: radical cation of the Hantzsch ester
3DPAFIPN: tris(diphenylamino)-5-fluoroisophthalonitrile
4CzIPN: tetrakis(carbazol-9-yl)-4,6-dicyanobenzene
DPPP: 1,3-bis(diphenylphosphino)propane
DPPE: 1,2-bis(diphenylphosphino)ethane
(±)-BINAP: (±)-2,2′-Bis(diphenylphosphino)-1,1′-binaphthalene
TLC: thin layer chromatography
