# Supplementary material for: Photoredox/Nickel-Catalyzed Diastereoselective Allylation of Aldehydes with Morita–Baylis–Hillman Adducts
Source: ACS Catal. 2025 May 2;15(10):8303–16. doi: 10.1021/acscatal.5c00891 (PMC12090220; doi:10.1021/acscatal.5c00891)
Supplement: Supplementary file 1 — cs5c00891_si_001.pdf [file cs5c00891_si_001.pdf]

Supporting Information:

Photoredox/Nickel-Catalyzed Diastereoselective Allylation of Aldehydes with Morita-Baylis-Hillman Adducts

Francesco Calogero,[a,b] *Emanuele Pinosa,*[a,b] *Andrea Gualandi,**[a,b] *Luca Sensoli,*[a,b] *Sayan Dutta,*[c] *Bholanath Maity,*[c] *Luigi Cavallo,*[c]* *Andrea Fermi,*[a,b] *Paola Ceroni,*[a,b] *and Pier Giorgio Cozzi**[a,b]

[a]ALMA MATER STUDIORUM - Università di Bologna, Dipartimento di Chimica "G. Ciamician", Via Gobetti 83, 40129, Bologna, Italy

[b]Center for Chemical Catalysis - C3, Alma Mater Studiorum - Università di Bologna, Via Gobetti 83, 40129, Bologna, Italy

[c]Physical Science and Engineering Division, King Abdullah University of Science and Technology (KAUST), Thuwal 23955-6900, Saudi Arabia

Email: andrea.gualandi2@unibo.it; luigi.cavallo@kaust.edu.sa; piergiorgio.cozzi@unibo.it

Synthetic details 3
General procedures..5
General procedure for dual photoredox nickel-catalyzed reaction between aldehydes and MBH-acetates: .5
General procedure for 2 mmol scale: 5
Additional information: 6
Characterization of the products..7
Unreactive Substrates 23
Post Functionalizations..24
Synthesis of Methyl-4-((tert-butyldimethylsilyl)oxy)-4-(4-chlorophenyl)-2-methylene-3-phenylbutanoate (4a)..24
Synthesis of 5-(4-chlorophenyl)-4-phenyl-3-(piperidin-1-ylmethyl)dihydrofuran-2(3H)-one (4b) 24
Synthesis of Methyl 4-(4-chlorophenyl)-4-hydroxy-2-((naphthalen-2-ylthio)methyl)-3-phenylbutanoate (4c)..25
Enantioselective Variant..26
Photophysical details 27
Preparation and reaction wioth $Ni(COD)_2$ 30
Preparation of $Ni(COD)_2$ 30
Photocatalytic reaction with $Ni(COD)_2$ 31
Dependence of yields in function of Baylis-Hillman adduct 32
Procedure used:..32
Computational Modeling..33
Computational Methodology..33
Estimation of energy barriers of single electron transfer steps using Marcus–Hush theory 33
Figure S9. Free energy profile for the oxidative addition of 2a to the Ni(I) species INT-C. 34
Figure S10. Energetics for the generation of Ni(0) species (INT-D) from INT-C via disproportionation reaction. 34
NMR Traces 73
References 174

Synthetic details

^{1}H-NMR spectra were recorded on Varian Mercury 400 spectrometer. Chemical shifts are reported in ppm from TMS with the solvent resonance as the internal standard ($CHCl_3$: δ = 7.27 ppm). Data are reported as follows: chemical shift, multiplicity (s = singlet, d = duplet, t = triplet, q = quartet, dd = double duplet, m = multiplet), coupling constants (Hz). ^{13}C-NMR spectra were recorded on Varian Mercury 400 spectrometer. Chemical shifts are reported in ppm from TMS with the solvent as the internal standard ($CDCl_3$: δ = 77.0 ppm). GC-MS spectra were taken by EI ionization at 70 eV on a Hewlett-Packard 5971 with GC injection.

All the reagents were purchased from commercial sources (Sigma-Aldrich, Alfa Aesar, Fluorochem, Strem Chemicals, TCI) and used without further purification unless specified.

All reactions requiring inert atmosphere were set up under an argon in heat gun-dried glassware using standard Schlenk techniques.

Anhydrous solvents were supplied by Aldrich in Sureseal® bottles and, unless specified, were used without further treatment.

Anhydrous tetrahydrofuran (THF) was obtained by standard sodium/benzophenone ketyl distillation starting from reagent grade THF, supplied by TCI® or Sigma Aldrich®.

Freshly distilled THF was stored under argon atmosphere in a dry Schlenk tube, equipped with a Rotaflo® stopcock with activated 3Å molecular sieves (*ca.* 5g of MS for 100 mL of solvent).

3Å molecular sieves were supplied by Sigma-Aldrich**.** Activation of the MS was performed through five 6-minute cycles in microwave (750W). Once the cycles are completed the MS are rapidly inserted in a dry Schlenk tube, equipped with a Rotaflo® stopcock and then flame-dried for 5 minutes under vacuum before starting the distillation.

Photocatalytic reactions were irradiated with Kessil® PR160L@456 nm.[1]

Ligands employed for the dual nickel/photoredox catalytic reaction (**L1–5, dppe, dppp, BINAP**), as well as the nickel complexes **$NiCl_2$•glyme** and **$NiCl_2(PBu_3)_2$** were purchased from commercial sources (Sigma-Aldrich, Fluorochem) and were used without further treatment.

2,4,6-tri(diphenylamino)-5-fluoroisophthalonitrile (**3DPAFIPN**) and 2,4,5,6-tetrakis(carbazol-9-yl)-isophthalonitrile (**4CzIPN**), were prepared according to published literature procedures.[2]

Synthesis of Hantzsch's ethyl ester was achieved following the known literature procedure.[3]

Substrates **2a-s**[4] were prepared according to the two-step standard Morita–Baylis–Hillman acetate (MBH–acetate) synthetic protocol. Spectroscopic data for MBH–alcohols **2b, S1-17** and for MBH–acetates **2a-s** match or are consistent with what has already been reported in the literature. [5] In the section entitled **NMR traces** of the following manuscript, ^{1}H-NMR of previously unprecedented MBH-Acetates (**2g, 2h, 2k, 2n, 2s**) are reported for clarity.
Substrates **1k**, **1q, 1r, 1s, 1t, 1p and 1w, 5a** were synthetized according to the known literature protocols and the related spectroscopic data match with those already reported.[6]

Figure S1. Emission profile of the Kessil® PR160L@456 nm used to irradiate the solutions (form Kessil® website: https://www.kessil.com/science/PR160L.php).

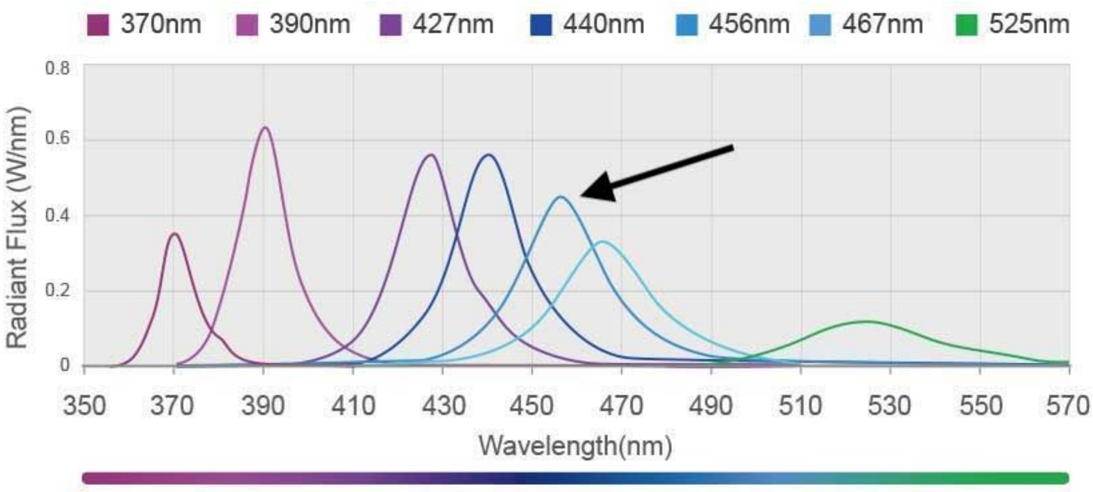

Figure S2. Reaction set-up with Kessil® PR160L@456 nm lamp.

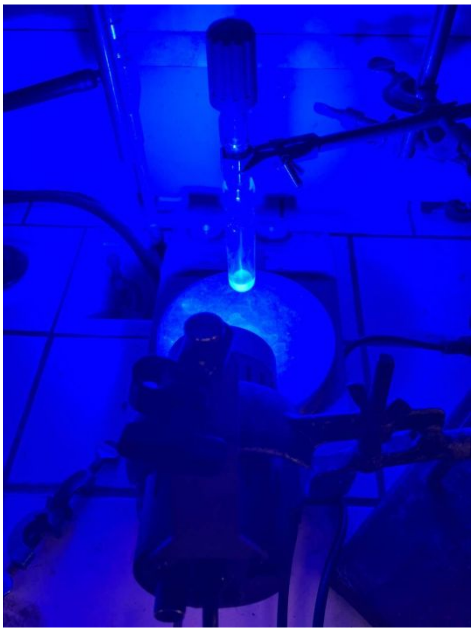

The reaction temperature was close to room temperature (*from 22-28°C)* during the irradiation as measured with a thermometer at 2 cm from the reaction flask.

General procedures

General procedure for dual photoredox nickel-catalyzed reaction between aldehydes and MBH-acetates:

All the reactions were performed on 0.2 mmol scale of aldehyde **1**. A heat-gun dried 10 mL Schlenk tube, equipped with a Rotaflo® stopcock, magnetic stirring bar and an argon supply tube, was firstly charged under vigorous argon flux with $NiCl_2$**.**glyme (10 mol%, 0.02 mmol, 4.4 mg), the substrate if solid **1** (0.2 mmol) the organic photocatalyst 3DPAFIPN (5 mol%, 0.01 mmol, 6.5 mg), diethyl 1,4-dihydro-2,6-dimethyl-3,5-pyridinedicarboxylate Hantzsch ester (2 equiv., 0.4 mmol, 101 mg) and the ligand **L1** (15 mol%, 0.03 mmol, 10.8 mg). Then, inhibitor-free and freshly distilled dry THF (2 mL in order to obtain a 0.1 M substrate solution) was added and the reaction mixture. Finally, substrate **2** (2.2 equiv. 0.44 mmol**)** and substrate **1** if liquid were added to the solution that was further subjected to a freeze-pump-thaw procedure (three cycles, 5 minutes per cycle) and then refilled with argon. The degassed reaction mixture was irradiated under vigorous stirring for 16 h. After that, the reaction mixture was quenched with HCl 1 M (10 mL approx.) and extracted with EtOAc (3 x 10 mL). The combined organic layers were dried over anhydrous Na_2SO_4 and the solvent was removed under reduced pressure. The reaction crude was analyzed by ^{1}H-NMR for the evaluation of the diastereomeric ratio. The NMR sample was carefully recovered, the solvent was removed under reduced pressure and purified by flash column chromatography (SiO_2) to afford products **3** in the stated yields.

Figure S3. a) Model reaction before the irradiation. b) Model reaction after quenching. c). Reagents of the model reaction.

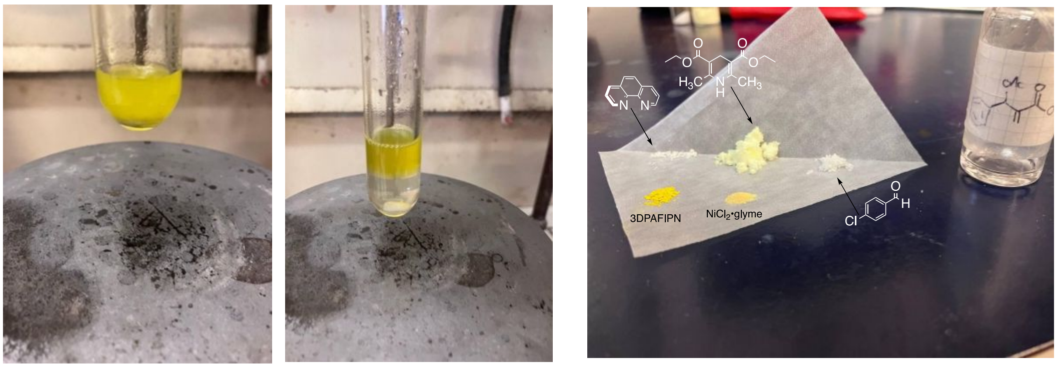

General procedure for 2 mmol scale:

A heat-gun dried 50 mL Schlenk tube, equipped with a magnetic stirring bar and an argon supply tube, was firstly charged under vigorous argon flux with $NiCl_2$**.**glyme (5 mol%, 0.1 mmol, 22 mg), the substrate if solid **1a** (1 equivalent, 2 mmol, 280 mg) the organic photocatalyst 3DPAFIPN (5 mol%, 0.06 mmol, 39 mg), diethyl 1,4-dihydro-2,6-dimethyl-3,5-pyridinedicarboxylate Hantzsch ester (2 equiv., 4 mmol, 1.010 g) and the ligand **L1** (7.5 mol %, 0.15 mmol, 27 mg). Then, inhibitor-free and freshly distilled dry THF (20 mL in order to obtain a 0.1 M substrate solution) was added and the reaction mixture. Finally, substrate **2a** (2 equiv., 4.4 mmol, 1.030 g**)** was added to the solution that was further subjected to a freeze-pump-thaw procedure (three cycles, 5 minutes per cycle) and then refilled with argon. The degassed reaction mixture was irradiated under vigorous stirring for 60 h. After that, the reaction mixture was quenched with HCl 1 M (approx. 20 mL) and extracted with EtOAc (3 x 15 mL). The combined organic layers were dried over anhydrous Na_2SO_4 and the solvent was removed under reduced pressure. The reaction crude was analyzed by ^{1}H-NMR for the evaluation of the diastereomeric ratio. The NMR sample was carefully recovered, the solvent was removed under reduced pressure and purified by flash chromatography (SiO_2 DCM/Hex from 6:4 to 8:2) to afford products **3a** in 80% yield (506 mg).

Additional information:

1. Before the freeze-pump-thaw procedure, the reaction mixture is stirred for approx. 5 min to favor the formation of the active complex between nickel and *o*-phenanthroline.

2. Careful drying of the reaction Schlenk is crucial for a successful outcome of the reaction.

3. Precautionary, once the solids were introduced inside the reaction vessel, the Schlenk was placed under vacuum for approx. 10 minutes to avoid contamination of the reaction mixture with atmospheric moisture.

4. Before irradiation, the light-yellow reaction mixture appears heterogeneous due to the non-complete solubility of HE in THF. At the end of the reaction, the reaction color appears light orange.

5. If during irradiation or at the end of the reaction, the reaction mixture appears green, this is normally detrimental to the reaction outcome, and poor conversion is observed. Empirically, this phenomenon has been attributed to insufficient dryness of the reaction mixture, normally depending on the solvent.

6. Vigorous stirring (1000 rpm) is beneficial given the heterogeneous nature of the reaction mixture.

7. Precautionary, due to the high sensitivity to oxygen and moisture of dual transition metal and photoredox processes, monitoring of reaction progress by TLC analysis was avoided to avoid contamination even under vigorous argon flow. The reaction time established for each of the pairs of substrates (**1** and **2**) considered derives from the optimization of the reaction conditions and is mainly influenced by the electronic characteristics of the substrates.

8. The use of aqueous HCl has a twofold beneficial effect in quenching the reaction. a) it promotes the dissolution of the Ni/*o*-phen organometallic complex by allowing its aqueous phase transition. b) it contributes to the partial protonation of pyridine (HEpyr) resulting from the oxidation of HE during the photomediated reaction, facilitating subsequent purification of the desired product by flash chromatography.

Characterization of the products

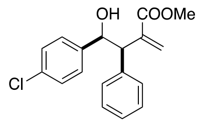

Methyl–4-(4-chlorophenyl)-4-hydroxy-2-methylene-3-phenylbutanoate (**3aa**)

Yield 87% (0.174 mmol, 55mg).

Physical form Pale yellow oil.

$dr_{syn:anti}$ > 95:5 wa determined from ^{1}H-NMR of the reaction crude by integration of the 2 C-**H** in the benzylic position.

3aa was obtained following the general procedure as stated above employing 0.2 mmol (28 mg, 1 equiv.) of **1a** and 0.44 mmol (103 mg, 2.2 equiv.) of **2a**.

The title compound was isolated by flash column chromatography (SiO_2, 50-30% Hexane in DCM).

Spectroscopical data are in accordance with the reported literature.[7]

^{1}HNMR (401 MHz, $CDCl_3$) δ 7.37 – 7.21 (m, 10H, overlapped with the residual peak of NMR solvent), 6.25 (s, 1H), 5.80 (s, 1H), 5.27 (d, *J* = 7.8 Hz, 1H), 4.24 (d, *J* = 7.7 Hz, 1H), 3.60 (s, 3H).

^{13}CNMR (101 MHz, $CDCl_3$) δ 166.6, 140.5, 140.2, 137.8, 133.1, 128.8 (2C), 128.3 (2C), 128.0 (2C), 128.0 (2C), 127.1, 126.8, 74.7, 54.2, 51.6.

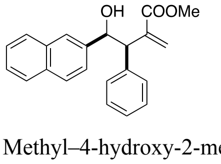

Methyl–4-hydroxy-2-methylene-4-(naphthalen-2-yl)-3-phenylbutanoate (**3ba**)

Yield 77% (0.154 mmol, 51mg).

Physical form Pale yellow oil.

$dr_{syn:anti}$ > 95:5 was determined from ^{1}H-NMR of the reaction crude by integration of the 2 C-**H** in the benzylic position.

3ba was obtained following the general procedure as stated above employing 0.2 mmol (31.2 mg, 1 equiv.) of **1b** and 0.44 mmol (103 mg, 2.2 equiv.) of **2a**.

The title compound was isolated by flash column chromatography (SiO_2, 50-30% Hexane in DCM).

Spectroscopical data are in accordance with the reported literature.[8]

^{1}HNMR (401 MHz, $CDCl_3$) δ 7.80 (m, 3H), 7.72 – 7.69 (m, 1H), 7.49 – 7.44 (m, 3H), 7.38 – 7.23 (m, 6H overlapped with the residual peak of NMR solvent), 6.22 (d, *J* = 0.9 Hz, 1H), 5.85 (s, 1H), 5.44 (d, *J* = 7.8 Hz, 1H), 4.43 (d, *J* = 7.9 Hz, 1H), 3.51 (s, 3H).

^{13}CNMR (101 MHz, $CDCl_3$) δ 166.6, 140.7, 139.1, 138.2, 132.7, 132.7, 128.9 (2C), 128.2 (2C), 127.7, 127.7, 127.3, 126.9, 126.59, 125.9, 125.7, 125.6, 124.4, 75.5, 53.9, 51.5.

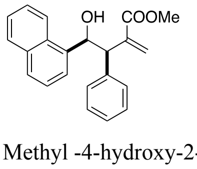

Methyl -4-hydroxy-2-methylene-4-(naphthalen-1-yl)-3-phenylbutanoate (**3ca**)

Yield 68% (0.136 mmol, 45 mg).

Physical form Pale yellow oil.

$dr_{syn:anti}$ > 95:5 was determined from ^{1}H-NMR of the reaction crude by integration of the 2 C-**H** in the benzylic position.

3ca was obtained following the general procedure as stated above employing 0.2 mmol (31.2 mg, 27.1 µl 1 equiv.) of **1c** and 0.44 mmol (103 mg, 2.2 equiv.) of **2a**.

1c was distilled prior the use.

The title compound was isolated by flash column chromatography (SiO_2, 50-30% Hexane in DCM)).

Spectroscopical data are in accordance with the reported literature.[8]

^{1}**HNMR (401 MHz, CDCl$_3$)** δ 8.24 (m, 1H), 7.92 – 7.86 (m, 1H), 7.76 (m, 1H), 7.54 (m, 2H), 7.37 – 7.16 (m, 8H overlapped with the residual peak of NMR solvent), 6.35 (s, 1H), 6.07 (d, J = 5.7 Hz, 1H), 6.00 (s, 1H), 4.61 (d, J = 5.7 Hz, 1H), 3.59 (s, 3H).

^{13}**CNMR (101 MHz, CDCl$_3$)** δ 167.5, 141.5, 137.9, 137.9, 134.6, 130.9, 129.9 (2C), 129.3, 128.5(2C), 128.5, 127.5, 127.2, 126.6, 125.8, 125.3, 124.7, 123.4, 72.0, 52.9, 52.2.

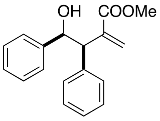

Methyl-4-hydroxy-2-methylene-3,4-diphenylbutanoate (**3da**)

Yield 54% (0.108 mmol, 30.5 mg).

Physical form Pale yellow oil.

dr$_{syn:anti}$ > 95:5 was determined from ^{1}H-NMR of the reaction crude by integration of the 2 C-**H** in the benzylic position.

3da was obtained following the general procedure as stated above employing 0.2 mmol (21.2 mg, 20.3 µl, 1 equiv.) of **1d** and 0.44 mmol (103 mg, 2.2 equiv.) of **2a**.

1d was distilled prior the use.

The title compound was isolated by flash column chromatography (SiO$_2$, 50-30% Hexane in DCM).

Spectroscopical data are in accordance with the reported literature.[8]

^{1}**HNMR (401 MHz, CDCl$_3$)** δ 7.34 – 7.21 (m, 10H overlapped with the residual peak of NMR solvent), 6.23 (s, 1H), 5.80 (s, 1H), 5.26 (d, J = 7.9 Hz, 1H), 4.31 (d, J = 7.6 Hz, 1H), 3.56 (s, 3H).

^{13}**CNMR (101 MHz, CDCl$_3$)** 166.9, 142.0, 141.1, 138.6, 129.2 (2C), 128.5(2C), 128.2(2C), 127.8, 127.2, 127.0(2C), 126.8, 75.8, 54.3, 51.8.

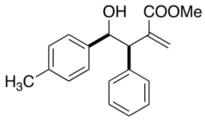

Methyl -4-hydroxy-2-methylene-3-phenyl-4-(p-tolyl)butanoate (**3ea**)

Yield 68% (0.136 mmol, 340.2 mg).

Physical form Pale yellow oil.

dr$_{syn:anti}$ > 95:5 was determined from ^{1}H-NMR of the reaction crude by integration of the 2 C-**H** in the benzylic position.

3ea was obtained following the general procedure as stated above employing 0.2 mmol (24.3 mg, 23.8 µl, 1 equiv.) of **1e** and 0.44 mmol (103 mg, 2.2 equiv.) of **2a**.

1e was distilled prior the use.

The title compound was isolated by flash column chromatography (SiO$_2$, 50-30% Hexane in DCM).

Spectroscopical data are in accordance with the reported literature.[9]

^{1}**HNMR (401 MHz, CDCl$_3$)** δ 7.29 – 7.17 (m, 5H overlapped with the residual peak of NMR solvent), 7.14 – 7.10 (m, 2H), 7.03 (d, J = 7.8 Hz, 2H), 6.15 (s, 1H), 5.72 (s, 1H), 5.16 (d, J = 8.3 Hz, 1H), 4.24 (d, J = 8.2 Hz, 1H), 3.50 (s, 3H), 2.25 (s, 3H).

^{13}**CNMR (101 MHz, CDCl$_3$)** δ 166.9, 141.1, 139.0, 138.8, 137.4, 136.0, 129.2 (2C), 128.9(2C), 128.5(2C), 127.2, 126.9(2C), 126.7, 75.6, 54.2, 51.8, 21.1.

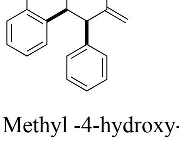

Methyl -4-hydroxy-2-methylene-3-phenyl-4-(o-tolyl)butanoate (**3fa**)

Yield 68% (0.134 mmol, 39.7 mg).

Physical form Pale yellow oil.

dr$_{syn:anti}$ > 95:5 was determined from ^{1}H-NMR of the reaction crude by integration of the 2 C-**H** in the benzylic position.

3fa was obtained following the general procedure as stated above employing 0.2 mmol (24.3 mg, 23.8 µl, 1 equiv.) of **1f** and 0.44 mmol (103 mg, 2.2 equiv.) of **2a**.

1f was distilled prior the use.

The title compound was isolated by flash column chromatography (SiO_2, 50-30% Hexane in DCM).

^{1}HNMR (401 MHz, $CDCl_3$) δ 7.25 (m, 5H overlapped with the residual peak of NMR solvent), 7.15 – 7.06 (m, 4H), 6.26 (s, 1H), 5.85 (s, 1H), 5.45 (d, *J* = 6.0 Hz, 1H), 4.38 (d, *J* = 7.0 Hz, 1H), 3.56 (s, 3H), 2.41 (s, 3H).

^{13}CNMR (101 MHz, $CDCl_3$) δ 167.4, 141.6, 140.2, 138.5, 135.4, 130.8, 129.8, 128.7, 127.8, 127.5 (2C), 126.9(2C), 126.8, 126.2, 72.2, 52.6, 52.2, 19.7.

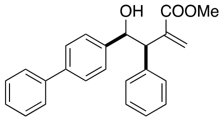

Methyl -4-([1,1'-biphenyl]-4-yl)-4-hydroxy-2-methylene-3-phenylbutanoate (**3ga**)

Yield 81% (0.162 mmol, 58.0 mg).

Physical form White solid.

$dr_{syn:anti}$ > 95:5 was determined from ^{1}H-NMR of the reaction crude by integration of the 2 C-**H** in the benzylic position.

3ga was obtained following the general procedure as stated above employing 0.2 mmol (36. 4mg, 1 equiv.) of **1g** and 0.44 mmol (103 mg, 2.2 equiv.) of **2a**.

The title compound was isolated by flash column chromatography (SiO_2, 50-30% Hexane in DCM).

^{1}HNMR (401 MHz, $CDCl_3$) δ 7.61 – 7.53 (m, 4H), 7.44 (dd, *J* = 8.3, 6.9 Hz, 2H), 7.41 – 7.27 (m, 9H), 6.28 (s, 1H), 5.85 (s, 1H), 5.33 (d, *J* = 8.0 Hz, 1H), 4.38 (d, *J* = 8.0 Hz, 1H), 3.59 (s, 3H).

^{13}CNMR (101 MHz, $CDCl_3$) δ 166.9, 141.0, 141.0, 140.7, 140.6, 138.6, 129.2 (2C), 128.7(2C), 128.6(2C), 127.6, 127.4 (2C), 127.3 (2C), 127.0, 126.9, 126.9, 75.5, 54.2, 51.8.

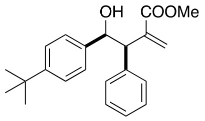

Methyl 4-(4-(*tert*-butyl)phenyl)-4-hydroxy-2-methylene-3-phenylbutanoate (**3ha**)

Yield 50% (0.10 mmol, 33.8 mg).

Physical form Pale yellow oil.

$dr_{syn:anti}$ > 95:5 was determined from ^{1}H-NMR of the reaction crude by integration of the 2 C-**H** in the benzylic position.

3ha was obtained following the general procedure as stated above employing 0.2 mmol (32. 4mg, 33.4 µl,1 equiv.) of **1h** and 0.44 mmol (103 mg, 2.2 equiv.) of **2a**.

1h was distilled prior the use.

The title compound was isolated by flash column chromatography (SiO_2, 50-30% Hexane in DCM).

^{1}HNMR (401 MHz, $CDCl_3$) δ 7.45 – 7.18 (m, 9H overlapped with the residual peak of NMR solvent), 6.24 (s, 1H), 5.79 (s, 1H), 5.22 (d, *J* = 8.4 Hz, 1H), 4.35 (d, *J* = 8.4 Hz, 1H), 3.56 (s, 3H), 1.31 (s, 9H).

^{13}CNMR (101 MHz, $CDCl_3$) δ 167.0, 150.7, 141.2, 139.0, 138.8, 129.2(2C), 128.5 (2C), 127.2, 126.8 (2C), 126.6, 125.2 (2C), 75.7, 53.9, 51.8, 34.4, 31.3 (3C).

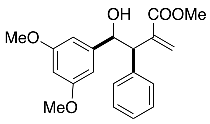

Methyl-4-(3,5-dimethoxyphenyl)-4-hydroxy-2-methylene-3-phenylbutanoate(**3ia**)

Yield 30% (0.06 mmol, 20.5 mg).

Physical form Pale yellow oil.

$dr_{syn:anti}$ = 90:10 was determined from ^{1}H-NMR of the reaction crude by integration of the 2 C-**H** in the benzylic position.

3ia was obtained modifying the general procedure extending the reaction time from 16 to 48h, employing 0.2 mmol (33. 2 mg,1 equiv.) of **1i** and 0.44 mmol (103 mg, 2.2 equiv.) of **2a.**

The title compound was isolated by flash column chromatography (SiO_2, 100% DCM).

Spectroscopical data are given only for the major diastereoisomer.

^{1}HNMR (401 MHz, $CDCl_3$) δ 7.34 – 7.30 (m, 4H), 6.45 (m, 2H), 6.35 (m, 1H), 6.26 (d, J = 0.8 Hz, 1H), 5.86 (s, 1H), 5.22 (d, J = 7.6 Hz, 1H), 4.27 (d, J = 7.1 Hz, 1H), 3.75 (s, 6H), 3.61 (s, 3H).

^{13}CNMR (101 MHz, $CDCl_3$) δ 166.7, 160.3, 144.2, 140.7, 138.17, 129.0 (2C), 128.2 (2C), 126.9, 126.5, 104.6, 99.6, 75.4, 55.0 (2C), 53.9, 51.6

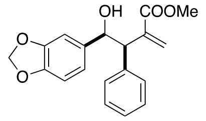

Methyl 4-(benzo[*d*][1,3]dioxol-5-yl)-4-hydroxy-2-methylene-3-phenylbutanoate(**3ja**)

Yield 46% (0.092 mmol, 30.0 mg).

Physical form Pale yellow oil.

dr$_{syn:anti}$> 95:5 was determined from ^{1}H-NMR of the reaction crude by integration of the 2 C-**H** in the benzylic position.

3ja was obtained modifying the general procedure extending the reaction time from 16 to 48h, employing 0.2 mmol (30.0 mg,1 equiv.) of **1j** and 0.44 mmol (103 mg, 2.2 equiv.) of **2a.**

The title compound was isolated by flash column chromatography (SiO_2, 100% DCM).

^{1}HNMR (401 MHz, $CDCl_3$) δ 7.39 – 7.23 (m, 5H, overlapped with the residual peak of NMR solvent), 6.84 (m, 1H), 6.80 – 6.67 (m, 2H), 6.23 (d, J = 0.9 Hz, 1H), 5.8 (s, 2H), 5.77 (s, 1H), 5.21 (d, J = 8.3 Hz, 1H), 4.24 (dd, J = 8.3, 0.9 Hz, 1H), 3.61 (s, J = 0.9 Hz, 3H).

^{13}CNMR (101 MHz, $CDCl_3$) δ 166.9, 147.6, 147.8, 141.0, 138.7, 136.0, 129.1(2C), 128.6(2C), 127.3, 126.8, 120.7, 107.9, 107.3, 100.9, 75.5, 54.6, 51.9.

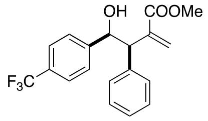

Methyl-4-hydroxy-2-methylene-3-phenyl-4-(4-(trifluoromethyl)phenyl)butanoate (**3ka**)

Yield 71% (0.142 mmol, 49.7 mg).

Physical form Pale yellow oil.

dr$_{syn:anti}$= 85:15 was determined from ^{19}F-NMR of the reaction crude.

3ka was obtained following the general procedure as stated above employing 0.2 mmol (34.8 mg, 27 µl, 1 equiv.) of **1k** and 0.44 mmol (103 mg, 2.2 equiv.) of **2a.**

1k was distilled prior the use.

The title compound was isolated by flash column chromatography (SiO_2, 50-30% Hexane in DCM).

Spectroscopical data are given only for the major diastereoisomer.

^{1}HNMR (401 MHz, $CDCl_3$) δ 7.50 (m, 2H), 7.37 (m, 2H), 7.33 – 7.16 (m, 5H overlapped with the residual peak of NMR solvent), 6.21 (s, 1H), 5.76 (s, 1H), 5.30 (d, J = 7.6 Hz, 1H), 4.22 (d, J = 7.6 Hz, 1H), 3.53 (s, 3H).

^{13}CNMR (101 MHz, $CDCl_3$) δ 167.2, 146.3, 141.0, 138.3, 130.2 (q, J = 32.4 Hz, 1C), 129.5 (2C), 129.4, 129.0 (2C),128.7, 127.8, 127.6 (2C), 125.4 (q, J = 3.8 Hz), 75.4, 54.9, 52.3.

^{19}F NMR (377 MHz, $CDCl_3$) δ -61.29 (s, 3F)

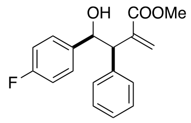

Methyl -4-(4-fluorophenyl)-4-hydroxy-2-methylene-3-phenylbutanoate (**3la**)

Yield 78% (0.156 mmol, 46.8 mg).

Physical form Pale yellow oil.

$dr_{syn:anti}$ >95:5 was determined from ^{19}F-NMR of the reaction crude.

3la was obtained following the general procedure as stated above employing 0.2 mmol (24.8 mg, 21.4 µl, 1 equiv.) of **1l** and 0.44 mmol (103 mg, 2.2 equiv.) of **2a.**

1l was distilled prior the use.

The title compound was isolated by flash column chromatography (SiO_2, 50-30% Hexane in DCM).

^{1}HNMR (401 MHz, CDCl$_3$) δ 7.36 – 7.21 (m, 7H overlapped with the residual peak of NMR solvent), 6.97 (m, 2H), 6.22 (s, 1H), 5.77 (s, 1H), 5.26 (d, *J* = 8.0 Hz, 1H), 4.24 (d, *J* = 8.0 Hz, 1H), 3.57 (s, 3H).

^{13}CNMR (101 MHz, CDCl$_3$) δ 166.9, 162.3 (d, *J* = 246.0 Hz, 1C), 140.9, 138.4, 137.7 (d, *J* = 3.2 Hz, 1C), 129.1 (2C), 128.64 (2C), 128.57 (d, *J* = 8.1 Hz, 2C), 127.3, 127.0, 115.1 (d, *J* = 21.4 Hz, 2C), 75.1, 54.6, 51.9.

^{19}F NMR (377 MHz, CDCl$_3$) δ -114.6 (m 1F)

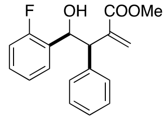

Methyl -4-(2-fluorophenyl)-4-hydroxy-2-methylene-3-phenylbutanoate (**3ma**)

Yield 30% (0.06 mmol, 18.0 mg).

Physical form Pale yellow oil.

$dr_{syn:anti}$ >95:5 was determined from ^{19}F-NMR of the reaction crude.

3ma was obtained following the general procedure as stated above employing 0.2 mmol (24.8 mg, 21.1 µl, 1 equiv.) of **1m** and 0.44 mmol (103 mg, 2.2 equiv.) of **2a.**

1m was distilled prior the use.

The title compound was isolated by flash column chromatography (SiO_2, 50-30% Hexane in DCM).

^{1}HNMR (401 MHz, CDCl$_3$) δ 7.36 – 7.21 (m, 7H overlapped with the residual peak of NMR solvent), 7.11 – 6.96 (m, 2H), 6.28 (s, 1H), 5.93 (s, 1H), 5.58 (d, *J* = 7.9 Hz, 1H), 4.40 (d, *J* = 7.9 Hz, 1H), 3.59 (s, 3H).

^{13}CNMR (101 MHz, CDCl$_3$) δ 166.6, 159.6 (d, *J* = 245.7 Hz, 1C), 140.3, 138.0, 129.0 (d, *J* = 8.5 Hz, 1C), 128.7 (2C), 128.3 (2C), 128.0 (d, *J* = 4.1 Hz, 1C), 127.0, 126.3 (d, *J* = 1.6 Hz, 1C), 123.9, 123.8, 114.9 (d, *J* = 22.3 Hz, 1C), 69.0 (d, *J* = 2.2 Hz, 1C), 52.7, 51.6.

^{19}F NMR (377 MHz, CDCl$_3$) δ -118.5 (m 1F).

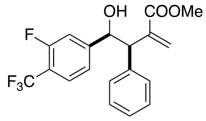

Methyl -4-(3-fluoro-4-(trifluoromethyl)phenyl)-4-hydroxy-2-methylene-3-phenylbutanoate (**3na**)

Yield 57% (0.114 mmol, 41.9 mg).

Physical form Pale yellow oil.

$dr_{syn:anti}$ = 92:8 was determined from ^{19}F-NMR of the reaction crude.

3na was obtained following the general procedure as stated above employing 0.2 mmol (38.4 mg, 1 equiv.) of **1n** and 0.44 mmol (103 mg, 2.2 equiv.) of **2a**.

The title compound was isolated by flash column chromatography (SiO_2, 50-30% Hexane in DCM).

^{1}HNMR (401 MHz, CDCl$_3$) Spectroscopical data are given only for the major diastereoisomer. δ 7.54 – 7.43 (m, 2H), 7.35 – 7.22 (m, 5H overlapped with the residual peak of NMR solvent), 7.13 – 7.07 (m, 1H), 6.25 (s, 1H), 5.78 (s, 1H), 5.34 (d, *J* = 8.4 Hz, 1H), 4.19 (dd, *J* = 7.7, 0.9 Hz, 1H), 3.60 (s, 3H).

^{13}CNMR (101 MHz, CDCl$_3$) all peaks are given without assignment. δ 166.9, 160.34, 160.32, 157.80, 157.78, 140.6, 138.33, 138.30, 137.7, 132.4, 132.30, 129.5, 129.22, 129.19, 129.1, 129.00, 128.95, 128.78, 128.75, 128.57, 128.55, 127.9, 127.6, 127.4, 126.0, 125.88, 125.86, 125.83, 125.82, 125.79, 125.77, 125.74, 125.73, 123.9, 123.5, 123.4, 122.7, 121.8, 121.2, 118.2, 118.1, 117.86, 117.74, 116.73, 116.6, 116.5, 116.4, 81.0, 74.6, 60.4, 55.0, 52.0, 51.5.

^{19}F NMR (377 MHz, CDCl$_3$) Spectroscopical data are given only for the major diastereoisomer. δ -61.42 (d, *J* = 12.7 Hz, 3F), -115.60 – -116.71 (m, 1F).

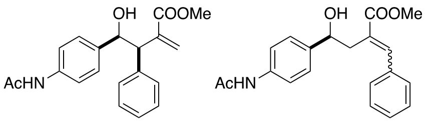

Methyl-4-(4-acetamidophenyl)-4-hydroxy-2-methylene-3-phenylbutanoate and methyl 4-(4-acetamidophenyl)-2-benzylidene-4-hydroxybutanoate (**3oa + 3oa')**

Yield 67% (0.13 mmol, 45 mg) reported as sum of two regioisomers.

Physical form Pale yellow oil.

3oa:3oa' 65:35 Regioisomeric ratio was determined ^{1}H-NMR of the reaction crude by integration of the two different C-**H** in the benzylic position.

For **3oa** $dr_{syn:anti}$ >95:5 was determined from ^{1}H-NMR of the reaction crude by integration of the C-**H** in the benzylic position.

For **3oa''** *E:Z*=56:4 was determined from ^{1}H-NMR of the reaction crude by integration of the C-**H** in the benzylic position.

3oa was obtained following the general procedure as stated, employing 0.2 mmol (32 mg, 1 equiv.) of **1o** and 0.4 mmol (93.6 mg, 2.0 equiv.) of **2a.**

The title compound was isolated by flash column chromatography (SiO_2, 100-20% Hexane in DCM) as a mixture of **3oa**:**3oa'**E: **3oa'**Z 6:3:1.

^{1}HNMR (600 MHz, $CDCl_3$) δ 7.54 – 7.36 (m, 39H), 7.36 – 7.28 (m, 23H), 7.28 – 7.22 (m, 22H, overlapped with the residual signal of the NMR solvent), 7.21 – 7.13 (m, 6H), 6.67 (s, 3H, **3oa'**), 6.23 (s, 6H, **3oa**), 5.80 (s, 6H, **3oa**), 5.24 (d, J = 8.1 Hz, 6H, **3oa**), 4.94 (dd, J = 8.9, 4.1 Hz, 1H, **3oa'**), 4.89 (dd, J = 8.2, 4.2 Hz, 3H, **3oa'**), 4.27 (d, J = 8.1 Hz, 6H, **3oa**), 3.83 (s, 3H, **3oa'**), 3.63 (s, 6H, **3oa'**), 3.58 (s, 18H, **3oa**), 2.97 (dd, J = 13.5, 9.5 Hz, 1H, **3oa'**), 2.90 (dd, J = 14.1, 4.2 Hz, 1H, **3oa'**), 2.81 (dd, J = 14.1, 4.3 Hz, 3H, **3oa'**), 2.73 (dd, J = 14.0, 8.4 Hz, 1H, **3oa'**).

^{13}CNMR (151 MHz, $CDCl_3$) all peaks are given without assignment. δ 169.8, 168.7, 167.4, 167.4, 166.0, 141.3, 139.9, 139.2, 138.5, 137.6, 136.8, 136.6, 136.4, 136.2, 134.7, 134.1, 129.6, 129.1, 129.0, 128.2, 128.1, 127.9, 127.6, 127.5, 127.2, 127.1, 127.1, 126.6, 126.3, 126.0, 125.5, 125.3, 118.9, 118.8, 118.5, 74.3, 72.3, 72.2, 53.3, 51.4, 50.94, 50.93, 45.2, 44.5, 36.4, 35.4, 29.9, 28.7, 23.6, 23.6.

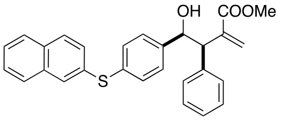

Methyl-4-hydroxy-2-methylene-4-(4-(naphthalen-2-ylthio)phenyl)-3-phenylbutanoate **(3pa)**

Yield 61% (0.122 mmol, 54 mg)

Physical form Yellow oil.

$dr_{syn:anti}$ >95:5 was determined from ^{1}H-NMR of the reaction crude by integration of the C-**H** in the benzylic position.

3pa was obtained following the general procedure as stated, employing 0.2 mmol (53 mg, , 1 equiv.) of **1p** and 0.4 mmol (47 mg, 2.0 equiv.) of **2a.**

The title compound was isolated by flash column chromatography (SiO_2, 100-20% Hexane in DCM).

^{1}HNMR (401 MHz, $CDCl_3$) δ 7.79 – 7.60 (m, 4H), 7.44 – 7.36 (m, 2H), 7.30 (dd, J = 8.5, 1.9 Hz, 1H), 7.26 – 7.23 (m, 5H, overlapped with the residual peak of NMR solvent), 7.22 – 7.17 (m, 4H), 6.19 (d, J = 0.8 Hz, 1H), 5.74 (s, 1H), 5.21 (d, J = 7.9 Hz, 1H), 4.21 (dd, J = 7.9, 1.0 Hz, 1H), 3.52 (s, 3H).

^{13}CNMR (151 MHz, $CDCl_3$) δ 166.9, 141.1, 140.8, 138.3, 135.0, 133.7, 133.0, 132.2, 131.0 (2C), 129.5, 129.2 (2C), 128.8, 128.7 (2C), 128.5, 127.9 (2C), 127.7, 127.4, 127.4, 127.1, 126.6, 126.2, 75.3, 54.4, 52.0.

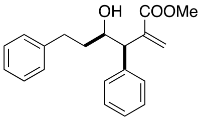

Methyl -4-hydroxy-2-methylene-3,6-diphenylhexanoate (**3qa**)

Yield 50% (0.10 mmol, 31.0 mg)

Physical form Pale yellow oil.

$dr_{syn:anti}$ >95:5 was determined from ^{1}H-NMR of the reaction crude by integration of the C-**H** in the benzylic position.

3oa was obtained modifying the general procedure extending the reaction time from 16 to 48h, employing 0.2 mmol (26.8 mg, 26.3 µl, 1 equiv.) of **1q** and 0.4 mmol (93.6 mg, 2.0 equiv.) of **2a.**

1q was distilled prior the use.

The title compound was isolated by flash column chromatography (SiO_2, 50-30% Hexane in DCM).

^{1}HNMR (401 MHz, $CDCl_3$) δ = 7.36 – 7.22 (m, 7H, overlapped with the residual signal of the NMR solvent), 7.23 – 7.13 (m, 3H), 6.32 (d, J = 0.8 Hz, 1H), 5.74 (d, J = 1.0 Hz, 1H), 4.13 (ddd, J = 9.4, 6.9, 3.0 Hz, 1H), 3.92 (dd, J = 6.9, 1.0 Hz, 1H), 3.66 (s, 3H), 2.86 (ddd, J = 14.4, 9.7, 5.2 Hz, 1H), 2.70 (ddd, J = 13.8, 9.4, 7.0 Hz, 1H), 1.90 (dddd, J = 13.9, 9.9, 7.1, 3.0 Hz, 1H), 1.70 (ddt, J = 13.7, 9.1, 4.6 Hz, 1H).

^{13}CNMR (101 MHz, $CDCl_3$) δ = 167.2, 141.9, 141.4, 138.8, 129.2 (2C), 128.6 (2C), 128.5 (2C), 128.3 (2C), 127.2, 126.1, 125.8, 72.1, 52.7, 52.0, 37.0, 32.2.

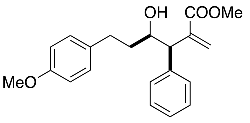

Methyl -4-hydroxy-6-(4-methoxyphenyl)-2-methylene-3-phenylhexanoate (**3ra**)

Yield 49% (0.10 mmol, 33.3 mg)

Physical form Pale yellow oil.

$dr_{syn:anti}$ >95:5 was determined from ^{1}H-NMR of the reaction crude by integration of the C-**H** in the benzylic position.

3ra was obtained modifying the general procedure extending the reaction time from 16 to 48h, employing 0.2 mmol (32.8, 1 equiv.) of **1r** and 0.4 mmol (93.6 mg, 2.0 equiv.) of **2a.**

The title compound was isolated by flash column chromatography (SiO_2, 40-30% Hexane in DCM).

^{1}HNMR (401 MHz, $CDCl_3$) δ = 7.29 (d, *J* = 4.3 Hz, 4H, overlapped with the residual signal of the NMR solvent), 7.25 – 7.19 (m, 1H), 7.10 – 7.05 (m, 2H), 6.83 – 6.77 (m, 2H), 6.31 (d, *J* = 0.8 Hz, 1H), 5.74 (d, *J* = 1.0 Hz, 1H), 4.10 (d, *J* = 8.6 Hz, 1H), 3.91 (dd, *J* = 6.9, 1.0 Hz, 1H), 3.76 (s, 3H), 3.65 (s, 3H), 2.78 (ddd, *J* = 14.4, 9.5, 5.2 Hz, 1H), 2.64 (ddd, *J* = 13.9, 9.2, 7.1 Hz, 1H), 1.85 (dddd, *J* = 13.9, 9.8, 7.1, 3.0 Hz, 1H), 1.66 (ddt, *J* = 13.6, 9.0, 4.6 Hz, 1H), 1.46 (d, *J* = 4.7 Hz, 1H).

^{13}CNMR (101 MHz, $CDCl_3$) δ = 167.2, 157.7, 141.4, 138.8, 133.9, 129.4 (2C), 129.2 (2C), 128.6 (2C), 127.2, 126.1, 113.8 (2C), 72.0, 55.2, 52.6, 52.0, 37.3, 31.2.

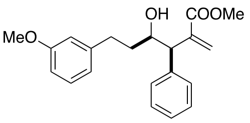

Methyl -4-hydroxy-6-(3-methoxyphenyl)-2-methylene-3-phenylhexanoate (**3sa**)

Yield 56% (0.112 mmol, 38.1 mg).

Physical form Pale yellow oil.

$dr_{syn:anti}$ >95:5 was determined from ^{1}H-NMR of the reaction crude by integration of the C-**H** in the benzylic position.

3pa was obtained modifying the general procedure extending the reaction time from 16 to 48h, employing 0.2 mmol (32.8, 1 equiv.) of **1s** and 0.4 mmol (93.6 mg, 2.0 equiv.) of **2a.**

The title compound was isolated by flash column chromatography (SiO_2, 60-30% Hexane in DCM).

^{1}HNMR (401 MHz, $CDCl_3$) δ = 7.29 (m, 4H), 7.25 – 7.22 (m, 1H), 7.20 – 7.14 (m, 1H), 6.78 – 6.69 (m, 3H), 6.32 (s, 1H), 5.75 (s, 1H), 4.13 (ddd, *J* = 9.3, 6.8, 2.9 Hz, 1H), 3.93 – 3.89 (m, 1H), 3.77 (s, 3H), 3.65 (s, 3H), 2.83 (ddd, *J* = 14.4, 9.7, 5.2 Hz, 1H), 2.67 (ddd, *J* = 13.7, 9.4, 7.0 Hz, 1H), 1.89 (dddd, *J* = 13.9, 9.9, 7.0, 3.0 Hz, 1H), 1.69 (ddt, *J* = 13.6, 9.0, 4.6 Hz, 1H).

^{13}CNMR (101 MHz, $CDCl_3$) δ = 167.2, 159.6, 143.6, 141.4, 138.8, 129.3, 129.2 (2C), 128.6 (2C), 127.2, 126.1, 120.9, 114.2, 111.1, 72.1, 55.1, 52.7, 52.0, 36.9, 32.3.

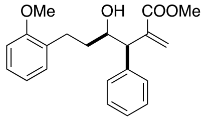

Methyl -4-hydroxy-6-(2-methoxyphenyl)-2-methylene-3-phenylhexanoate (**3ta**)

Yield 60% (0.12 mmol, 40.8 mg)

Physical form Pale yellow oil

dr$_{syn:anti}$ >95:5 was determined from ^{1}H-NMR of the reaction crude by integration of the C-**H** in the benzylic position.

3ra was obtained modifying the general procedure extending the reaction time from 16 to 48h, employing 0.2 mmol (32.8, 1 equiv.) of **1t** and 0.4 mmol (93.6 mg, 2.0 equiv.) of **2a.**

The title compound was isolated by flash column chromatography (SiO_2, 60-30% Hexane in DCM).

^{1}HNMR (401 MHz, $CDCl_3$) δ = 7.30 – 7.09 (m, 7H, overlapped with the residual signal of the NMR solvent), 6.89 – 6.80 (m, 2H), 6.31 (s, 1H), 5.79 (s, 1H), 4.13 – 4.05 (m, 1H), 3.93 – 3.90 (m, 1H), 3.78 (s, 3H), 3.63 (s, 3H), 2.83 – 2.70 (m, 2H), 1.92 – 1.82 (m, 1H), 1.76 (s, 1H), 1.64 (qd, J = 8.6, 4.4 Hz, 1H).

^{13}CNMR (101 MHz, $CDCl_3$) δ = 167.2, 157.3, 141.7, 138.9, 130.1, 130.1, 129.3 (2C), 128.4 (2C), 127.1, 127.0, 126.0, 120.6, 110.3, 72.1, 55.3, 52.2, 51.9, 35.7, 26.6.

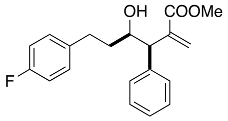

Methyl-6-(4-fluorophenyl)-4-hydroxy-2-methylene-3-phenylhexanoate (**3ua**)

Yield 34% (0.07 mmol, 22.3 mg).

Physical form Pale yellow oil.

dr$_{syn:anti}$ = 84:16 was determined from ^{19}F-NMR of the reaction crude.

3sa was obtained modifying the general procedure extending the reaction time from 16 to 48h, employing 0.2 mmol (30.4 mg, 1 equiv.) of **1u** and 0.4 mmol (93.6 mg, 2.0 equiv.) of **2a.**

The title compound was isolated by flash column chromatography (SiO_2, 60-30% Hexane in DCM).

Spectroscopical data are given only for the major diastereoisomer.

^{1}HNMR (401 MHz, $CDCl_3$) δ = 7.34 – 7.27 (m, 5H, overlapped with the residual signal of the NMR solvent), 7.14 – 7.09 (m, 2H), 6.97 – 6.91 (m, 2H), 6.32 (d, J = 0.8 Hz, 1H), 5.73 (s, 1H), 4.10 (ddd, J = 9.1, 7.0, 3.0 Hz, 1H), 3.91 (dd, J = 6.9, 1.0 Hz, 1H), 3.66 (s, 3H), 2.82 (ddd, J = 14.4, 9.5, 5.1 Hz, 1H), 2.72 – 2.63 (m, 1H), 1.86 (dddd, J = 13.9, 9.9, 7.2, 3.0 Hz, 1H), 1.72 – 1.60 (m, 1H).

^{13}CNMR (101 MHz, $CDCl_3$) δ = 167.1, 161.2 (d, J = 243 Hz), 141.3, 138.7, 137.5 (d, J = 3.3 Hz), 129.8, 129.7, 129.1 (2C), 129.0, 128.8, 128.7 (2C), 127.2, 126.1, 115.1, 114.9, 71.9, 52.7, 52.0, 37.1, 31.3.

^{19}F NMR (377 MHz, $CDCl_3$) δ = -116.6 (m, 1F).

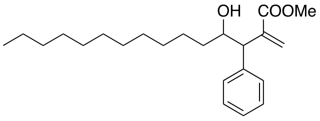

Methyl- 4-hydroxy-2-methylene-3-phenylpentadecanoate (**3va**)

Yield 56% (0.112 mmol, 40.3 mg).

Physical form Pale yellow oil.

dr$_{syn:anti}$ >95:5 was determined from ^{1}H-NMR of the reaction crude by integration of the C-**H** in the benzylic position.

3ta was obtained modifying the general procedure extending the reaction time from 16 to 48h, employing 0.2 mmol (36.8, 44.3 µl, 1 equiv.) of **1v** and 0.4 mmol (93.6 mg, 2.0 equiv.) of **2a.**

1v was distilled prior the use.

The title compound was isolated by flash column chromatography (SiO_2, 60-30% Hexane in DCM).

^{1}HNMR (401 MHz, $CDCl_3$) δ 7.33 – 7.19 (m, 6H, overlapped with the residual signal of the NMR solvent), 6.35 (s, 1H), 5.87 (s, 1H), 4.12 (ddt, J = 8.3, 6.4, 3.3 Hz, 1H), 3.90 (d, J = 6.4 Hz, 1H), 3.67 (s, 3H), 1.53 – 1.23 (m, 20H), 0.86 (t, J = 6.8 Hz, 3H).

^{13}CNMR (101 MHz, $CDCl_3$) 167.3, 141.7, 138.8, 129.3 (2C), 128.5 (2C), 127.1, 126.1, 72.8, 52.5, 52.0, 35.4, 31.9, 29.6, 29.6, 29.6, 29.5, 29.3, 25.9, 22.7, 21.5, 14.1.

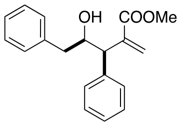

Methyl-4-hydroxy-2-methylene-3,5-diphenylpentanoate (**3wa**)
Yield 49% (0.098 mmol, 29.0 mg).
Physical form Pale yellow oil.
$dr_{syn:anti}$ >95:5 was determined from ^{1}H-NMR of the reaction crude by integration of the C-**H** in the benzylic position.
3ua was obtained modifying the general procedure extending the reaction time from 16 to 48h, employing 0.2 mmol (24.0 mg , 22,3 µl, 1 equiv.) of **1w** and 0.4 mmol (93.6 mg, 2.0 equiv.) of **2a.**
1w was distilled prior the use.
The title compound was isolated by flash column chromatography (SiO_2, 60-30% Hexane in DCM).
Spectroscopical data are in accordance with the reported literature.[10]
^{1}HNMR (401 MHz, $CDCl_3$) δ = 7.37 – 7.28 (m, 5H), 7.27 – 7.17 (m, 5H, overlapped with the residual signal of the NMR solvent), 6.38 (s, 1H), 5.91 (s, 1H), 4.41 (td, *J* = 6.4, 3.4 Hz, 1H), 3.98 (d, *J* = 1.0 Hz, 1H), 3.68 (s, 3H), 2.90 (dd, *J* = 13.9, 3.5 Hz, 1H), 2.63 (dd, *J* = 13.9, 9.0 Hz, 1H), 1.58 (s, 1H).
^{13}CNMR (101 MHz, $CDCl_3$) δ = 167.2, 141.6, 138.7, 138.6, 129.4 (2C), 129.3 (2C), 128.5 (2C), 128.5 (2C), 127.1, 126.5, 126.4, 73.7, 52.3, 52.0, 41.9.

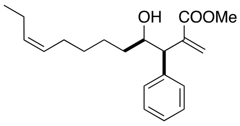

Methyl-4-hydroxy-2-methylene-3-phenyldodec-9-enoate (**3xa**)
Yield 68% (0.136 mmol, 42.9 mg).
Physical form Pale yellow oil.
$dr_{syn:anti}$ >95:5 was determined from ^{1}H-NMR of the reaction crude by integration of the C-**H** in the benzylic position.
3xa was obtained modifying the general procedure extending the reaction time from 16 to 48h, employing 0.2 mmol (28.4 mg, 33.8 µl, 1 equiv.) of **1x** and 0.4 mmol (93.6 mg, 2.0 equiv.) of **2a**.
The title compound was isolated by flash column chromatography (SiO_2, 60-30% Hexane in DCM).
^{1}HNMR (401 MHz, $CDCl_3$) δ = 7.32 – 7.28 (m, 4H, overlapped with the residual signal of the NMR solvent), 7.22 (ddt, *J* = 8.6, 3.6, 2.4 Hz, 1H), 6.34 (s, 1H), 5.86 (s, 1H), 5.40 – 5.22 (m, 2H), 4.15 – 4.08 (m, 1H), 3.89 (dd, *J* = 6.5, 1.0 Hz, 1H), 3.66 (s, 3H), 2.00 (qdd, *J* = 8.7, 4.6, 2.0 Hz, 4H), 1.61 – 1.25 (m, 7H), 0.92 (t, *J* = 7.6 Hz, 3H).
^{13}CNMR (101 MHz, $CDCl_3$) 167.2, 141.7, 138.8, 131.7, 129.3 (2C), 128.9, 128.5 (2C), 127.1, 126.1, 72.7, 52.5, 52.0, 35.3, 29.6, 27.0, 25.6, 20.5, 14.4.

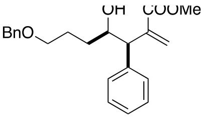

Methyl -7-(benzyloxy)-4-hydroxy-2-methylene-3-phenylheptanoate (**3ya**)
Yield 49% (0.098 mmol, 34.7 mg).
Physical form Pale yellow oil.
$dr_{syn:anti}$ = 92:8 was determined from ^{1}H-NMR of the reaction crude by integration of the C-**H** in the benzylic position.
3ya was obtained modifying the general procedure extending the reaction time from 16 to 48h, employing 0.2 mmol (35.6, 1 equiv.) of **1y** and 0.4 mmol (93.6 mg, 2.0 equiv.) of **2a.**
The title compound was isolated by flash column chromatography (SiO_2, 60-30% Hexane in DCM).

Spectroscopical data are given only for the major diastereoisomer.

^{1}HNMR (401 MHz, $CDCl_3$) δ = 7.33 – 7.21 (m, 10H, overlapped with the residual signal of the NMR solvent), 6.34 (s, 1H), 5.86 (s, 1H), 4.48 (s, 2H), 4.20 – 4.13 (m, 1H), 3.90 (d, J = 6.5 Hz, 1H), 3.67 (s, 3H), 3.47 (t, J = 5.9 Hz, 2H), 2.02 (d, J = 5.4 Hz, 1H), 1.74 (ddd, J = 12.4, 6.3, 4.4 Hz, 2H), 1.58 (m, 2H).

^{13}CNMR (101 MHz, $CDCl_3$) δ = 167.3, 141.7, 139.0, 138.3(2C), 129.3 (2C), 129.1(2C), 128.7(2C), 128.5, 128.3, 128.3, 127.6, 127.5, 127.5, 127.0, 126.1, 72.9, 72.5, 70.2, 52.6, 52.0, 32.5, 26.2.

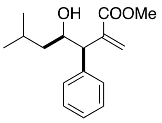

Methyl-4-hydroxy-6-methyl-2-methylene-3-phenylheptanoate (**3za**)

Yield 80% (0.160 mmol, 41.9 mg).

Physical form Pale yellow oil.

$dr_{syn:anti}$ >95:5 was determined from ^{1}H-NMR of the reaction crude by integration of the C-**H** in the benzylic position.

3xa was obtained modifying the general procedure extending the reaction time from 16 to 48h, employing 0.2 mmol (17.2, 21.4 µl, 1 equiv.) of **1z** and 0.4 mmol (93.6 mg, 2.0 equiv.) of **2a**.

1z was distilled prior the use.

The title compound was isolated by flash column chromatography (SiO_2, 60-30% Hexane in DCM).

^{1}HNMR (401 MHz, $CDCl_3$) δ = 7.34 – 7.29 (m, 4H , overlapped with the residual signal of the NMR solvent), 6.36 (s, 1H), 5.87 (s, 1H), 4.24 – 4.16 (m, 1H), 3.86 (dd, J = 6.5, 1.1 Hz, 1H), 3.67 (s, 3H), 1.83 (tt, J = 13.4, 6.7 Hz, 1H), 1.32 (dd, J = 7.9, 5.2 Hz, 2H), 0.91 (d, J = 3.6 Hz, 3H), 0.90 (d, J = 3.9 Hz, 3H).

^{13}CNMR (101 MHz, $CDCl_3$) δ = 167.2, 141.7, 138.9, 136.1, 129.3 (2C), 128.5 (2C), 127.1, 126.0, 70.8, 52.9, 52.0, 44.6, 24.7, 23.6.

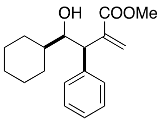

Methyl-4-cyclohexyl-4-hydroxy-2-methylene-3-phenylbutanoate (**3aaa**)

Yield 50% (0.100 mmol, 28.8 mg)

Physical form Pale yellow oil.

$dr_{syn:anti}$ = 92:8 was determined from ^{1}H-NMR of the reaction crude by integration of the C-**H** in the benzylic position.

3aaa was obtained modifying the general procedure extending the reaction time from 16 to 48h, employing 0.2 mmol (22.4, 24.2 µl, 1 equiv.) of **1aa** and 0.4 mmol (93.6 mg, 2.0 equiv.) of **2a.**

1aa was distilled prior the use.

The title compound was isolated by flash column chromatography (SiO_2, 60-30% Hexane in DCM).

Spectroscopical data are given only for the major diastereoisomer.

^{1}HNMR (401 MHz, $CDCl_3$) δ = 7.37 – 7.26 (m, 5H, overlapped with the residual signal of the NMR solvent), 7.23 (d, J = 9.1 Hz, 1H), 6.37 – 6.34 (m, 1H), 5.81 (s, 1H), 4.13 (d, J = 6.5 Hz, 1H), 3.88 (t, J = 6.0 Hz, 1H), 3.69 (s, 3H), 1.74 (s, 3H), 1.65 – 1.59 (m, 1H), 1.39 – 1.09 (m, 8H).

^{13}CNMR (101 MHz, $CDCl_3$) δ = 167.3, 141.7, 139.2, 129.4 (2C), 128.5 (2C), 127.0, 126.5, 76.8, 52.0, 49.0, 40.3, 30.3, 27.0, 26.4, 26.3, 26.0.

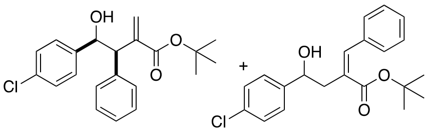

Tert-butyl-4-(4-chlorophenyl)-4-hydroxy-2-methylene-3-phenylbutanoate (**3ae**) and *tert*-butyl-2-benzylidene-4-(4-chlorophenyl) -4-hydroxybutanoate (**3ae'**)

Yield 81% (0.162 mmol, 57.9 mg) reported as sum of two regioisomers.

Physical form Pale yellow oil.

3ae:3ae' 80:20. Regioisomeric ratio was determined ^{1}H-NMR of the reaction crude by integration of the two different C-**H** in the benzylic position.

3ae, 3ae' were obtained following the general procedure as stated above employing 0.2 mmol (28 mg, 1 equiv.) of **1a** and 0.44 mmol (121 mg, 2.2 equiv.) of **2e**.

For **3ae** $dr_{syn:anti}$ >95:5 determined from ^{1}H-NMR of the reaction crude by integration of the C-**H** in the benzylic position.

For **3ae'** *E:Z*=1.2:1 determined from ^{1}H-NMR of the reaction crude by integration of the C-**H** in the benzylic position.

The title compound was isolated by flash column chromatography (SiO_2, 60-30% Hexane in DCM).

Spectroscopical data are given only for the major regioisomer.

^{1}HNMR (401 MHz, CDCl$_3$) δ 7.43 – 7.17 (m, 9H overlapped with the residual peak of the NMR solvent), 6.17 (d, *J* = 1.0 Hz, 1H), 5.69 (t, *J* = 1.0 Hz, 1H), 5.26 (d, *J* = 7.4 Hz, 1H), 4.19 (d, *J* = 7.4 Hz, 1H), 1.31 (s, 9H).

^{13}CNMR (101 MHz, CDCl$_3$) δ 165.7, 142.5, 141.5, 140.7, 138.5, 133.3, 129.2(2C), 129.0, 128.5(2C), 128.4, 128.4, 128.3(2C), 128.3 (2C), 127.2, 126.9, 126.0, 81.0, 75.0, 54.5, 27.8.

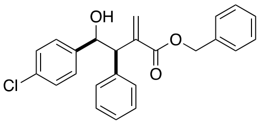

Benzyl-4-(4-chlorophenyl)-4-hydroxy-2-methylene-3-phenylbutanoate(**3af**)

Yield 65% (0.130 mmol, 51.0 mg).

Physical form Pale yellow oil.

$dr_{syn:anti}$ >95:5 was determined from ^{1}H-NMR of the reaction crude by integration of the C-**H** in the benzylic position.

3af was obtained following the general procedure as stated above employing 0.2 mmol (28 mg, 1 equiv.) of **1a** and 0.44 mmol (124.8 mg, 2.2 equiv.) of **2f**.

The title compound was isolated by flash column chromatography (SiO_2, 60-30% Hexane in DCM).

^{1}HNMR (401 MHz, CDCl$_3$) δ 7.32 – 7.22 (m, 10H, overlapped with the residual signal of the NMR solvent), 6.31 (s, 1H), 5.83 (s, 1H), 5.29 (d, *J* = 7.7 Hz, 1H), 5.08 – 4.98 (m, 2H), 4.26 (d, *J* = 7.7 Hz, 1H).

^{13}CNMR (101 MHz, CDCl$_3$) δ 166.2, 140.8, 140.1, 138.1, 135.6, 133.4, 129.2 (2C), 128.6 (2C), 128.6 (2C), 128.5 (2C), 128.4 (2C), 128.37, 128.3 (2C), 128.17, 128.14, 128.02, 127.97, 127.44, 127.39, 74.95, 66.65, 54.64.

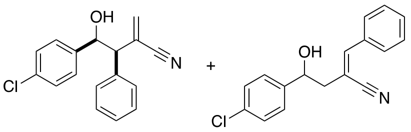

4-(4-chlorophenyl)-4-hydroxy-2-methylene-3-phenylbutanenitrile(**3ah**) and 2-benzylidene-4-(4-chlorophenyl)-4-hydroxybutanenitrile (**3ah'**)

Yield: 67% (0.134 mmol, 37.9 mg) reported as sum of two regioisomers.

Physical form Pale yellow oil.

3ah:3ah' 65:35. Regioisomeric ratio was determined ^{1}H-NMR of the reaction crude by integration of the two different C-**H** in the benzylic position.

For **3ah**: $dr_{syn:anti}$ = 94:6 determined from ^{1}H-NMR of the reaction crude by integration of the C-**H** in the benzylic position.

For **3ah'**: *E:Z*=1:1 determined from ^{1}H-NMR of the reaction crude by integration of the C-**H** in the benzylic position.

3eh, 3ah' were obtained following the general procedure as stated above employing 0.2 mmol (28 mg, 1 equiv.) of **1a** and 0.44 mmol (88.4 mg, 2.2 equiv.) of **2h**.

Spectroscopical data are given only for the major regioisomer.

The title compound was isolated by flash column chromatography (SiO_2, 60-30% Hexane in DCM).

^{1}HNMR (401 MHz, CDCl$_3$) δ 7.43 – 7.05 (m, 9H overlapped with the residual peak of the NMR solvent and the other regioisomer), 6.00 (s, 1H), 5.96 – 5.92 (m, 1H), 5.15 (d, *J* = 9.4 Hz, 1H), 3.67 (d, *J* = 9.4 Hz, 1H).

^{13}CNMR (101 MHz, CDCl$_3$) δ 147.0, 141.4, 139.9, 137.5, 128.8 (2C), 128.4 (2C), 128.0(4C), 124.11, 118.35, 74.97, 58.55.8.

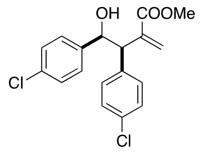

Methyl 3,4-bis(4-chlorophenyl)-4-hydroxy-2-methylenebutanoate (**3aj**)

Yield: 61% (0.122 mmol, 42.7.0 mg).

Physical form Pale yellow oil.

dr$_{syn:anti}$ >95:5 was determined from ^{1}H-NMR of the reaction crude by integration of the C-**H** in the benzylic position.

3aj was obtained following the general procedure as stated above employing 0.2 mmol (28 mg, 1 equiv.) of **1a** and 0.44 mmol (117.9 mg, 2.2 equiv.) of **2j**.

The title compound was isolated by flash column chromatography (SiO_2, 60-30% Hexane in DCM).

^{1}HNMR (401 MHz, $CDCl_3$) δ 7.32 – 7.12 (m, 8H overlapped with the residual peak of the NMR solvent), 6.25 (s, 1H), 5.80 (s, 1H), 5.26 (d, *J* = 7.1 Hz, 1H), 4.17 (d, *J* = 7.1 Hz, 1H), 3.61 (s, 3H).

^{13}CNMR (101 MHz, $CDCl_3$) δ 166.8, 140.6, 140.5, 136.6 (2C), 133.5(2C), 133.1(2C), 130.7(2C), 128.6, 128.4, 128.1, 127.4, 74.7, 53.9, 52.0.

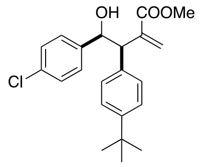

Methyl -3-(4-(*tert*-butyl)phenyl)-4-(4-chlorophenyl)-4-hydroxy-2-methylenebutanoate (**3ak**)

Yield: 49% (0.098 mmol, 36.5.0 mg).

Physical form: Pale yellow oil.

dr$_{syn:anti}$ = 94:6 was determined from ^{1}H-NMR of the reaction crude by integration of the two C-**H** in the benzylic position.

3ak was obtained following the general procedure as stated above employing 0.2 mmol (28 mg, 1 equiv.) of **1a** and 0.44 mmol (127.9 mg, 2.2 equiv.) of **2k.**

Spectroscopical data are given only for the major diastereoisomer.

The title compound was isolated by flash column chromatography (SiO_2, 60-30% Hexane in DCM).

^{1}HNMR (401 MHz, $CDCl_3$) δ 7.34 (m, 2H), 7.25 (m, 6H overlapped with the residual peak of NMR solvent), 6.29 (s, 1H), 5.74 (s, 1H), 5.23 (d, *J* = 8.4 Hz, 1H), 4.22 (d, *J* = 8.4 Hz, 1H), 3.58 (s, 3H), 1.30 (s, 9H).

^{13}CNMR (101 MHz, $CDCl_3$) δ 166.9, 150.3, 140.9, 140.5, 135.1, 133.4, 128.6 (2C), 128.5 (2C), 128.4 (2C), 126.9, 125.7 (2C), 75.3, 54.3, 51.9, 34.5, 31.3 (3C).

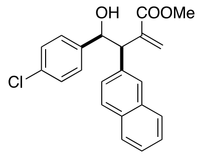

Methyl-4-(4-chlorophenyl)-4-hydroxy-2-methylene-3-(naphthalen-2-yl)butanoate (**3al**)

Yield: 51% (0.102 mmol, 37.3 mg)

Physical form: Pale yellow oil

dr$_{syn:anti}$ >95:5 was determined from ^{1}H-NMR of the reaction crude by integration of the two C-**H** in the benzylic position.

3al was obtained modifying the general procedure extending the reaction time from 16h to 48h, employing 0.2 mmol (28 mg, 1 equiv.) of **1a** and 0.4 mmol (113.6 mg, 2. equiv.) of **2l.**

The title compound was isolated by flash column chromatography (SiO_2, 60-30% Hexane in DCM).

^{1}HNMR (401 MHz, $CDCl_3$) δ 7.80 (d, *J* = 7.0 Hz, 4H), 7.48 – 7.36 (m, 3H), 7.26 (s, 4H overlapped with the residual peak of NMR solvent), 6.29 (s, 1H), 5.86 (s, 1H), 5.36 (d, *J* = 7.6 Hz, 1H), 4.42 (d, *J* = 7.7 Hz, 1H), 3.58 (s, 3H).

^{13}CNMR (101 MHz, $CDCl_3$) δ 166.9, 140.8, 140.5, 135.7, 133.5, 133.4, 132.7, 128.4 (2C), 128.3(2C), 128.3, 128.0, 127.9, 127.6, 127.2, 126.2, 126.0, 75.0, 54.5, 52.0.

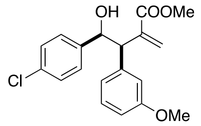

Methyl 4-(4-chlorophenyl)-4-hydroxy-3-(3-methoxyphenyl)-2-methylenebutanoate (**3am**)

Yield: 60% (0.120 mmol, 41.5 mg).

Physical form: Pale yellow oil

dr$_{syn:anti}$ >95:5 was determined from ^{1}H-NMR of the reaction crude by integration of the two C-**H** in the benzylic position.

3al was obtained modifying the general procedure extending the reaction time from 16h to 48h, employing 0.2 mmol (28 mg, 1 equiv.) of **1a** and 0.4 mmol (105.0 mg, 2. equiv.) of **2m.**

The title compound was isolated by flash column chromatography (SiO_2, in DCM).

^{1}HNMR (401 MHz, $CDCl_3$) δ 7.28 – 7.22 (m, 5H overlapped with the residual peak of NMR solvent), 6.93 – 6.81 (m, 3H), 6.25 (s, 1H), 5.80 (s, 1H), 5.25 (d, J = 7.8 Hz, 1H), 4.23 (d, J = 7.9 Hz, 1H), 3.78 (s, 3H), 3.60 (s, 3H).

^{13}CNMR (101 MHz, $CDCl_3$) δ 167.2, 160.0, 141.0, 140.8, 140.1, 133.7, 129.9 (2C), 128.7(2C), 128.6, 127.4, 121.6, 115.3, 112.97, 75.3, 55.4, 54.8, 52.3.

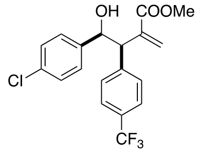

Methyl -4-(4-chlorophenyl)-4-hydroxy-2-methylene-3-(4-(trifluoromethyl)phenyl)butanoate (**3an**)

Yield: 50% (0.100 mmol, 38.4 mg).

Physical form: Pale yellow oil.

dr$_{syn:anti}$ = 95:5 was determined from ^{19}F-NMR of the reaction crude.

3an was obtained following the general procedure as stated above employing 0.2 mmol (28 mg, 1 equiv.) of **1a** and 0.4 mmol (120.9 mg, 2.0 equiv.) of **2n**.

Spectroscopical data are given only for the major diastereoisomer.

The title compound was isolated by flash column chromatography (SiO_2, 50%– 30% Hexane in DCM).

^{1}HNMR (401 MHz, $CDCl_3$) δ 7.56 (d, J = 8.1 Hz, 2H), 7.43 (d, J = 8.1 Hz, 2H), 7.32 – 7.10 (m, 5H overlapped with the residual peak of the nmr solvent), 6.30 (s, 1H), 5.84 (s, 1H), 5.35 (dd, J = 7.2, 3.3 Hz, 1H), 4.28 (d, J = 7.1 Hz, 1H), 3.63 (s, 3H).

^{13}CNMR (101 MHz, $CDCl_3$) δ 166.7, 142.5, 140.4, 140.3, 133.7, 129.7, 128.5 (2C), 128.1 (2C), 127.9, 127.8, 125.3 (q, J = 3.8 Hz), 74.7, 54.4, 52.1.

^{19}FNMR (377 MHz, $CDCl_3$): δ -62.54 (m, 3F).

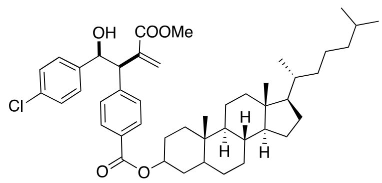

(8*R*,9*S*,10*S*,13*R*,14*S*,17*R*)-10,13-dimethyl-17-((*R*)-6-methylheptan-2-yl)hexadecahydro-1*H*-cyclopenta[*a*]phenanthren-3-yl 4-(1-(4-chlorophenyl)-1-hydroxy-3-(methoxycarbonyl)but-3-en-2-yl)benzoate (**3ao**)

Yield: 41% (0.082mmol, 59.9 mg).

Physical form: white solid.

dr$_{syn:anti}$ = 96:4 was determined from ^{1}H-NMR of the reaction crude by integration of two C-**H** in the benzylic positions.

3ao was obtained following the general procedure as stated above employing 0.2 mmol (28 mg, 1 equiv.) of **1a** and 0.4 mmol (256.0 mg, 2.0 equiv.) of **2o**.

Spectroscopical data are given only for the major diastereoisomer.

The title compound was isolated by two consecutive flash column chromatography (SiO_2, 1st 50%– 30% Hexane in DCM, 2nd 0%– 30% Et_2O in Hexane).

^{1}HNMR (401 MHz, $CDCl_3$) δ 7.96 (m, 2H), 7.36 (m, 2H), 7.27 (m, 3H), 7.21 (m 2H), 6.30 (s, 1H), 5.84 (s, 1H), 5.34 (d, J = 7.1 Hz, 1H), 4.96 – 4.91 (m, 1H), 4.29 (d, J = 7.1 Hz, 1H), 3.62 (s, 3H), 2.04 – 1.89 (m, 3H), 1.88 – 1.75 (m, 3H), 1.59 (m, 11H), 1.39 – 1.23 (m, 14H), 1.16 – 0.99 (m, 11H), 0.92 (m, 5H).

^{13}CNMR (101 MHz, $CDCl_3$) δ 166.7, 165.9, 143.3, 140.5, 140.4, 133.6, 129.9, 129.6 (2C), 129.3 (2C), 128.4(2C), 128.1 (2C), 127.6, 74.7, 74.4, 56.4, 56.3, 54.5, 54.2, 52.0, 44.7, 42.6, 39.9, 39.5, 36.8, 36.2, 35.8, 35.5, 34.1, 32.0, 28.6, 28.2, 28.0, 27.6, 24.2, 23.8, 22.8, 22.5, 21.2, 18.7, 12.2, 12.1.

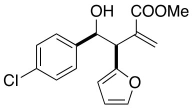

Methyl -4-(4-chlorophenyl)-3-(furan-2-yl)-4-hydroxy-2-methylenebutanoate (**3ap**)

Yield 58% (0.116 mmol, 35.5 mg).

Physical form Pale yellow oil

dr$_{syn:anti}$ >95:5 was determined from ^{1}H-NMR of the reaction crude by integration of the C-**H** in the benzylic position.

3ap was obtained following the general procedure as stated above employing 0.2 mmol (28 mg, 1 equiv.) of **1a** and 0.44 mmol (98.5 mg, 2.2 equiv.) of **2p**.

The title compound was isolated by flash column chromatography (SiO_2, 60%– 30% Hexane in DCM).

^{1}HNMR (401 MHz, $CDCl_3$) δ 7.37 (m, 1H), 7.29 – 7.16 (m, 4H overlapped with the residual peak of NMR solvent), 6.35 – 6.29 (m, 2H), 6.22 (d, J = 3.2 Hz, 1H), 5.78 (s, 1H), 5.15 (d, J = 6.7 Hz, 1H), 4.40 (d, J = 6.6 Hz, 1H), 3.66 (s, 3H).

^{13}CNMR (101 MHz, $CDCl_3$) δ 167.0, 152.4, 142.4, 140.2, 138.5, 133.6, 128.8, 128.5 (2C), 128.3(2C), 110.7, 108.9, 75.2, 52.4, 48.7.

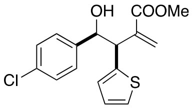

Methyl -4-(4-chlorophenyl)-4-hydroxy-2-methylene-3-(thiophen-2-yl)butanoate (**3aq**)

Yield 77% (0.154 mmol, 49.6 mg).

Physical form Pale yellow oil.

dr$_{syn:anti}$ >95:5 was determined from ^{1}H-NMR of the reaction crude by integration of the C-**H** in the benzylic position.

3aq was obtained modifying the general procedure extending the reaction time from 16 h to 48 h, employing 0.2 mmol (28 mg, 1 equiv.) of **1a** and 0.4 mmol (96.5 mg, 2.0 equiv.) of **2q**.

The title compound was isolated by flash column chromatography (SiO_2, 50%– 30% Hexane in DCM).

^{1}HNMR (401 MHz, $CDCl_3$) δ 7.26 (m, 4H overlapped with the residual peak of NMR solvent), 7.22 (m, 1H), 6.99 – 6.95 (m, 2H), 6.29 (s, 1H), 5.83 (s, 1H), 5.20 (d, J = 6.7 Hz, 1H), 4.53 (d, J = 6.7 Hz, 1H), 3.69 (s, 3H).

^{13}CNMR (101 MHz, $CDCl_3$) δ 166.8, 140.7, 140.4, 140.0, 133.4, 128.3 (2C), 128.1 (2C), 128.0, 127.0, 126.8, 125.2, 75.5, 52.1, 50.7.

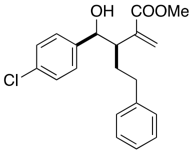

Methyl –(4-chlorophenyl)(hydroxy)methyl)-2-methylene-5-phenylpentanoate (**3ar)**

Yield 29% (0.058 mmol, 19.9 mg).

Physical form Pale yellow oil.

dr$_{syn:anti}$ = 88:12 was determined from ^{1}H-NMR of the reaction crude by integration of the C-**H** in the benzylic position.

3ar was obtained following the general procedure as stated above employing 0.2 mmol (28 mg, 1 equiv.) of **1a** and 0.4 mmol (104.8 mg, 2.0 equiv.) of **2r**.

The title compound was isolated by flash column chromatography (SiO_2, 50%– 30% Hexane in DCM).

Spectroscopical data are given only for the major diastereoisomer.

^{1}HNMR (401 MHz, CDCl$_3$) δ 7.31 – 7.09 (m, 7H overlapped with the residual peak of the NMR solvent), 7.11 – 6.80 (m, 2H), 6.29 (d, *J* = 1.0 Hz, 1H), 5.47 (s, 1H), 4.95 – 4.72 (m, 1H), 3.75 (s, 3H), 2.94 (dt, *J* = 10.9, 4.2 Hz, 1H), 2.55 (ddd, *J* = 14.6, 10.1, 4.9 Hz, 1H), 2.36 (ddd, *J* = 13.9, 9.9, 7.0 Hz, 1H), 1.94 (dtd, *J* = 13.7, 6.8, 3.6 Hz, 1H), 1.83 (ddt, *J* = 14.0, 9.7, 5.2 Hz, 1H).

^{13}CNMR (101 MHz, CDCl$_3$) δ 168.4, 141.7, 140.9, 140.0, 132.9, 128.3 (2C), 128.3(2C), 128.1(2C), 127.8(2C), 127.7, 125.8, 75.6, 52.2, 49.1, 33.5, 29.0.

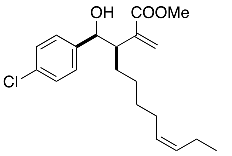

Methyl (*Z*)-3-((4-chlorophenyl)(hydroxy)methyl)-2-methyleneundec-8-enoate (**3as)**

Yield 26% (0.052 mmol, 18.0 mg).

Physical form Pale yellow oil.

dr$_{syn:anti}$> 95:5 was determined from ^{1}H-NMR of the reaction crude by integration of the C-**H** in the benzylic position.

3as was obtained following the general procedure as stated above employing 0.2 mmol (28 mg, 1 equiv.) of **1a** and 0.4 mmol (107.2 mg, 2.0 equiv.) of **2s**.

The title compound was isolated by flash column chromatography (SiO_2, 50%– 30% Hexane in DCM).

^{1}HNMR (401 MHz, CDCl$_3$) δ 7.80 (m, 1H), 7.72 – 7.68 (m, 2H), 7.64 (m, 1H), 7.57 – 7.40 (m, 3H), 7.32 – 7.04 (m, 12H overlapped with the residual peak of the nmr solvent), 5.31 (d, *J* = 8.0 Hz, 1H), 3.49 (d, *J* = 1.6 Hz, 3H), 3.41 (dd, *J* = 7.8, 5.8 Hz, 1H), 3.29 – 3.22 (m, 1H), 3.08 (m, 1H), 3.00 (dt, *J* = 8.3, 6.0 Hz, 1H).

^{13}CNMR (101 MHz, CDCl$_3$) δ 172.9, 140.4, 136.6, 133.6, 133.4, 132.2, 131.9, 129.4 (2C), 128.6, 128.4 (2C), 128.3, 128.3 (2C), 128.2 (2C), 127.9, 127.7(2C), 127.7, 127.1, 126.6, 126.0, 73.7, 54.2, 51.6, 46.7, 34.1.

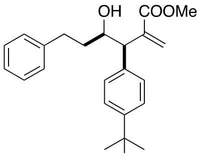

Methyl-3-(4-(*tert*-butyl)phenyl)-4-hydroxy-2-methylene-6-phenylhexanoate (**3qk)**

Yield 35% (0.07 mmol, 27 mg).

Physical form Pale yellow oil.

1q was distilled prior the use.

dr$_{syn:anti}$= 87:13 was determined from ^{1}H-NMR of the reaction crude by integration of the C-**H** in the benzylic position.

3qk was obtained following the general procedure as stated above extending the reaction time from 16 h to 48 h and employing 0.2 mmol (27 mg, 1 equiv.) of **1q** and 0.4 mmol (116 mg, 2.0 equiv.) of **2k**.

The title compound was isolated by flash column chromatography (SiO_2, 100-20% Hexane in DCM) and was obtained after column with a dr$_{syn:anti}$= 66:34

^{1}HNMR (600 MHz, CDCl$_3$) δ 7.39 – 7.31 (m, 6H, *syn*+*anti*), 7.30 – 7.26 (m, 4H, *syn*+*anti,* overlapped with the residual signal of the NMR solvent), 7.24 – 7.14 (m, 13H, *syn*+*anti*), 7.09 – 7.01 (m, 4H, *syn*+*anti*), 6.44 (d, J = 2.8 Hz, 1H, *anti*), 6.31 (d, J = 0.9 Hz, 2H, *syn*), 5.75 (t, J = 1.0 Hz, 2H, *syn*), 5.61 (d, J = 2.5 Hz, 1H, *anti*), 4.67 (ddd, J = 10.0, 7.9, 3.9 Hz, 1H, *anti*), 4.32 (dt, J = 8.0, 2.7 Hz, 1H, *anti*), 4.12 (ddd, J = 9.5, 7.1, 2.9 Hz, 2H, *syn*), 3.91 (d, J = 7.1 Hz, 2H, *syn*), 3.68 (s, 6H, *syn*), 3.49 (s, 3H, *anti*), 2.92 – 2.81 (m, 2H, *syn*), 2.79 – 2.64 (m, 3H, *syn*+*anti*), 2.61 – 2.46 (m, 1H, *anti*), 1.95 – 1.87 (m, 2H, *syn*), 1.76 – 1.67 (m, 2H, *syn*), 1.62 (ddt, J = 14.4, 9.5, 4.8 Hz, 1H, *anti*), 1.46 – 1.38 (m, 1H, *anti*), 1.31 (s, 9H, *anti*), 1.29 (s, 18H, *syn*).

^{13}CNMR (151 MHz, CDCl$_3$) δ 170.5, 167.3, 150.7, 150.0, 142.0, 141.5, 140.8, 139.1, 135.5, 134.2, 128.71 (2C), 128.7 (2C), 128.6 (2C), 128.5 (2C), 128.4 (2C), 128.3(2C), 126.0, 126.0, 125.8 (2C), 125.7 (2C), 125.6, 124.3, 80.6, 72.2, 52.2, 52.0, 48.8, 37.0, 34.5, 34.4, 34.2, 32.2, 31.30, 31.30 (3C), 31.27 (3C), 29.7.

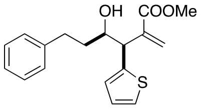

Methyl 4-hydroxy-2-methylene-6-phenyl-3-(thiophen-2-yl)hexanoate (**3qq)**

Yield 24% (0.048 mmol, 15 mg).

Physical form Pale yellow oil.

1q was distilled prior the use.

dr$_{syn:anti}$> 95:5 was determined from ^{1}H-NMR of the reaction crude by integration of the C-**H** in the benzylic position.

3qq was obtained following the general procedure as stated above extending the reaction time from 16 h to 48 h and employing 0.2 mmol (27 mg, 1 equiv.) of **1q** and 0.4 mmol (96 mg, 2.0 equiv.) of **2q**.

The title compound was isolated by flash column chromatography (SiO_2, 100-20% Hexane in DCM).

^{1}HNMR (600 MHz, $CDCl_3$) δ 7.29 – 7.26 (m, 2H, overlapped with the residual signal of the NMR solvent), 7.22 – 7.21 (m, 1H), 7.20 – 7.16 (m, 3H), 6.99 – 6.96 (m, 2H), 6.36 (d, J = 0.7 Hz, 1H), 5.83 (t, J = 0.7 Hz, 1H), 4.29 (d, J = 5.7 Hz, 1H), 4.07 (ddd, J = 9.1, 5.4, 3.6 Hz, 1H), 3.73 (s, 3H), 2.86 (ddd, J = 14.6, 9.9, 5.3 Hz, 1H), 2.71 (ddd, J = 13.8, 9.7, 6.8 Hz, 1H), 1.91 (bs, 1H), 1.84 (dddd, J = 13.6, 10.2, 6.9, 3.6 Hz, 1H), 1.76 (dddd, J = 14.0, 9.7, 8.9, 5.3 Hz, 1H).

^{13}CNMR (151 MHz, $CDCl_3$) δ 167.0, 141.8, 141.2, 141.0, 128.5 (2C), 128.4 (2C), 127.3, 126.82, 126.80, 125.8, 125.0, 72.5, 52.2, 48.3, 36.8, 32.2.

Unreactive Substrates

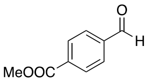

• No product formation observed
• Decomposition in the reaction mixture

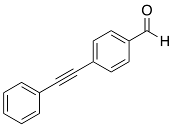

• No product formation observed
• Decomposition in the reaction mixture

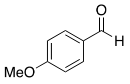

• 15% conversion after 72h of irradiation

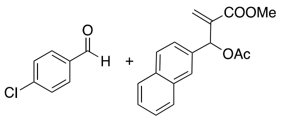

• Traces of product

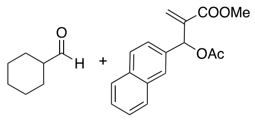

• Unreactive

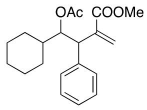

• Unreactive

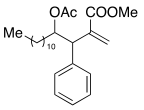

• Unreactive

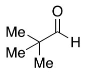

• Unreactive

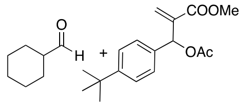

• Traces of product

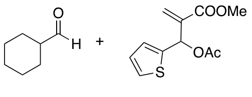

• Traces of product

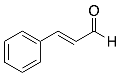

• Complex mixture resulting from the formation of byproducts by interactions between the double bond, [Ni] and $He_{pyr}H^+$

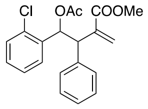

• 95% conversion after 16h of irradiation
• Pinacolization of **1a** observed as main byproduct
• Deacetylation of MBH–acetate observed

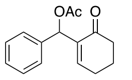

• 95% conversion after 16h of irradiation
• Pinacolization of **1a** observed as main byproduct
• Deacetylation of MBH–acetate observed

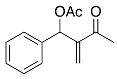

• Unreactive

Post Functionalizations

Synthesis of Methyl-4-((*tert*-butyldimethylsilyl)oxy)-4-(4-chlorophenyl)-2-methylene-3-phenylbutanoate (4a)

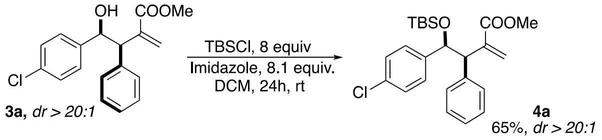

Product **4a** was synthetized slightly modifying the procedure reported by Ramachandran and co-workers.[5a] In a one-necked 5 mL round bottom flask equipped with a magnetic stirring bar, methyl -4-(4-chlorophenyl)-4-hydroxy-2-methylene-3-phenylbutanoate (**3a**, 0.1 mmol, 31.6 mg, 1 equiv.) was dissolved in 2 mL of reagent-grade DCM. Imidazole (0.55 mmol, 37 mg, 5.5 equiv.) was added to the reaction mixture. Finally, while stirring TBSCl (0.44 mmol, 81 mg., 4.4 equiv.) was added to the solution. The reaction was allowed to stir at room temperature and after 2 hours was monitored by TLC analysis (eluent 10% EtOAc in Hexane). Showing a non-total conversion of the starting material, the reaction mixture was charged with imidazole (0.26 mmol, 17.5 mg, 2.6 equiv.) and TBSCl (0.36 mmol, 66 mg, 3.6 equiv.) and was allowed to stir overnight. After 24 h TLC analysis (eluent 10% EtOAc in Hexane) showed a complete conversion of the starting material. The reaction was quenched with water (*ca.* 5 mL) and extracted with DCM (3 x 5 mL). The organic phase was dried over Na_2SO_4 and the solvent was removed under vacuum. Product **4a** was isolated as a white solid after flash chromatography (SiO_2, 5-15% EtOAc in Hexane) in 65% yield (28.0 mg).
^{1}H NMR (401 MHz, $CDCl_3$) δ 7.50 – 7.35 (m, 2H), 7.35 – 7.23 (m, 7H overlapped with the residual peak of the nmr solvent), 6.31 (s, 1H), 5.75 (t, *J* = 1.1 Hz, 1H), 5.30 (d, *J* = 7.0 Hz, 1H), 4.22 (d, *J* = 6.9 Hz, 1H), 3.74 (s, 3H), 0.79 (s, 9H), -0.14 (s, 3H), -0.27 (s, 3H).
^{13}C NMR (101 MHz, $CDCl_3$) δ 167.3, 142.1, 140.7, 139.3, 132.7, 129.9 (2C), 128.4 (2C), 127.9 (4C), 127.5, 126.7, 76.0, 55.9, 51.9, 25.6, 17.9, -4.7, -5.5.

Synthesis of 5-(4-chlorophenyl)-4-phenyl-3-(piperidin-1-ylmethyl)dihydrofuran-2(3*H*)-one (4b)

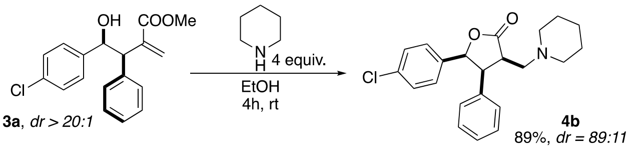

Product **4b** was synthetized slightly modifying the procedure reported by Ramachandran and co-workers.[5a] In a one-necked 5 mL round bottom flask equipped with a magnetic stirring bar, methyl -4-(4-chlorophenyl)-4-hydroxy-2-methylene-3-phenylbutanoate (**3a,** 0.1 mmol, 31.6 mg, 1 equiv.) was dissolved in 1 mL of reagent-grade EtOH (to obtain [**3a**] = 0.1 M). To the solution, piperidine (0.4 mmol, 34 mg, 39.5 µL, 4 equiv.) was added and the reaction was allowed to stir for 4 h at room temperature. The reaction crude was monitored by TLC analysis and, upon confirming a complete conversion of the starting material, it was quenched with water (*ca.* 1 mL). The reaction was extracted with Et_2O (3 x 5 mL), dried over Na_2SO_4 and the solvent was removed under vacuum. The product after the extraction was isolated without further purification as a white solid in 89% yield (32.8 mg, *dr* = 89:11). Spectroscopical data are given only for the major diastereoisomer.
^{1}H NMR (401 MHz, $CDCl_3$) δ 7.16 – 7.02 (m, 7H), 6.87 (dd, *J* = 6.7, 2.9 Hz, 2H), 5.78 (d, *J* = 5.2 Hz, 1H), 4.07 (dd, *J* = 7.5, 5.2 Hz, 1H), 3.43 (td, *J* = 8.2, 3.8 Hz, 1H), 2.72 (dd, *J* = 13.4, 3.8 Hz, 1H), 2.37 (br s, 2H), 2.19 (m, 1H), 2.08 (m, 2H), 1.50 (m, 4H), 1.38 (m, 2H), 1.26 (m,1H).
^{13}C NMR (101 MHz, $CDCl_3$) δ 177.5, 134.4, 134.4, 133.1, 129.4 (2C), 129.1, 128.2, 128.2, 127.3 (2C), 126.8 (2C), 82.3, 54.6, 54.4 (2C), 51.9, 45.9, 26.0 (2C), 24.1.

Synthesis of Methyl 4-(4-chlorophenyl)-4-hydroxy-2-((naphthalen-2-ylthio)methyl)-3-phenylbutanoate (4c)

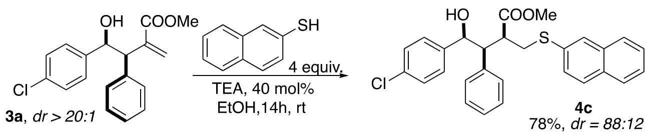

Product **4c** was synthetized applying standard condition for a sulfa-Micheal reaction. In a one-necked 5 mL round bottom flask equipped with a magnetic stirring bar, methyl -4-(4-chlorophenyl)-4-hydroxy-2-methylene-3-phenylbutanoate (**3a,** 0.1 mmol, 31.6 mg, 1 equiv.) was dissolved in 1 mL of reagent-grade EtOH (to obtain [**3a]** = 0.1 M). To the solution, of 2-thionaphtol (0.4 mmol, 63.6 mg, 4 equiv.) was added together with triethylamine (40 mol%, 0.02 mmol, 4.04 mg, 6 µL) and the reaction was allowed to stir for 14 h at room temperature. The reaction crude was monitored by TLC analysis and, upon confirming a complete conversion of the starting material, it was quenched with water (*ca.* 1 mL). The reaction was extracted with Et_2O (3 x 5 mL), dried over Na_2SO_4 and the solvent was removed under vacuum. The product after the extraction was isolated without further purification as a white solid in 78% yield (37.2 mg, *dr* = 88:12). Spectroscopical data are given only for the major diastereoisomer.

^{1}H NMR (401 MHz, $CDCl_3$) δ 7.80 (m, 1H), 7.72 – 7.68 (m, 2H), 7.64 (m, 1H), 7.57 – 7.40 (m, 3H), 7.32 – 7.04 (m, 12H overlapped with the residual peak of the nmr solvent), 5.31 (d, *J* = 8.0 Hz, 1H), 3.49 (d, *J* = 1.6 Hz, 3H), 3.41 (dd, *J* = 7.8, 5.8 Hz, 1H), 3.29 – 3.22 (m, 1H), 3.08 (m, 1H), 3.00 (dt, *J* = 8.3, 6.0 Hz, 1H).

^{13}C NMR (101 MHz, $CDCl_3$) δ 172.9, 140.4, 136.6, 133.6, 133.4, 132.2, 131.9, 129.4 (2C), 128.6, 128.4 (2C), 128.3, 128.3 (2C), 128.2 (2C), 127.9, 127.7(2C), 127.7, 127.1, 126.6, 126.0, 73.7, 54.2, 51.6, 46.7, 34.1.

Enantioselective Variant

Scheme S1. Screening of different chiral ligand tested

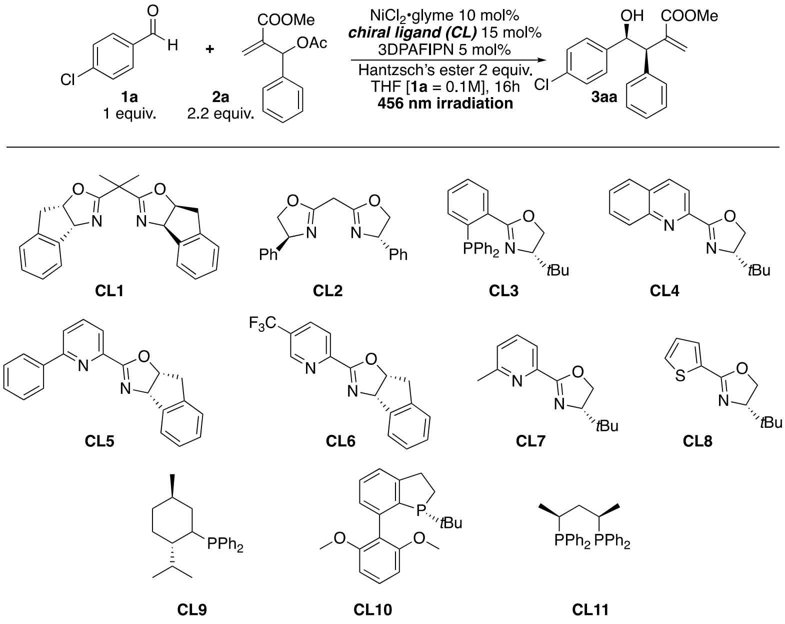

Entry	Chiral ligand	Yield (%)[a]	*d.r.*[b]	*ee(%)*
1[c,]	**CL1**	60	>95:5	33
2[c,d]	**CL1**	60	66:33	33
3	**CL2**	48	95:5	0
4	**CL3**	49	95:5	0
5	**CL4**	75	95:5	0
6	**CL5**	Traces	–	–
7	**CL6**	76	95:5	0
8[f]	**CL7**	Traces	–	–
9	**CL8**	No reaction	–	–
10	**CL9**	No reaction	–	–
11	**CL10**	No reaction	–	–
12	**CL11**	No reaction	–	–

[a] Reaction performed on 0.2 mmol scale and isolated after chromatographic purification.[b] Determined by ^{1}H-NMR analysis of the reaction crude. [c] Reaction performed at 10° C. [d] $Ni(OAc)_2$ instead of $NiCl_2$•glyme

Photophysical details

UV/vis absorbance spectra were recorded on an Agilent Cary 300 spectrophotometer, using sealed quartz suprasil (QS) cuvettes with an optical path of 10 mm. Emission spectra were recorded on a Perkin Elmer LS55 or an Edinburgh FS5 equipped with a PMT980 detector for UV and visible range. Emission lifetime measurements were performed on a Varian Cary Eclipse spectrofluorimeter equipped with a Hamamatsu R928 phototube, or on the above-mentioned Edinburgh FLS920 spectrofluorometer equipped with a TCC900 card for data acquisition in time-correlated single photon counting experiments (0.2 ns time resolution), with a 475 nm pulsed laser. The estimated experimental errors are 2 nm on the band maximum and 5% on the luminescence lifetime.

Quenching efficiencies η^i have been evaluated according to:

$$\eta^i = \frac{k_q^i \cdot [Q]^i}{k_r + k_{nr} + \sum_0^n k_q^n \cdot [Q]^n} \cdot 100$$

where k^i_q is the quenching constant for the ith quencher, $[Q]^i$ its concentration, k_{nr} the non-radiative constant and k_r the radiative constant. For compound HE: $k_q = 2.1x10^8\ M^{-1}s^{-1}$; [11] for the excited state T_1 of 3DPAFIPN: $k_r+k_{nr} = 9.6x10^3\ s^{-1}$. Considering the corresponding concentrations of HE (0.2 M) and the complex between Ni(II) species and o-phenantholine (0.01 M) at the reaction standard conditions: $\eta_{HE} \approx 98\%$; $\eta_{Ni} \approx 2\%$.

Figure S4. A Absorption spectra of solutions of 3DPAFIPN in degassed THF at r.t. (ca. 11 μM, blue line) obtained upon addition of increasing amounts of the complex between o-phenathroline and $Ni(OAc)_2$ (1.5:1.0 eqv in THF; up to ca. 0.12 mM). B Delayed fluorescence decays of 3DPAFIPN obtained from the same solutions at λ_{em}= 530 nm (λ_{ex}= 475 nm) and corresponding monoexponential fitting functions. C Stern-Volmer diagram relative to the determined decay lifetimes.

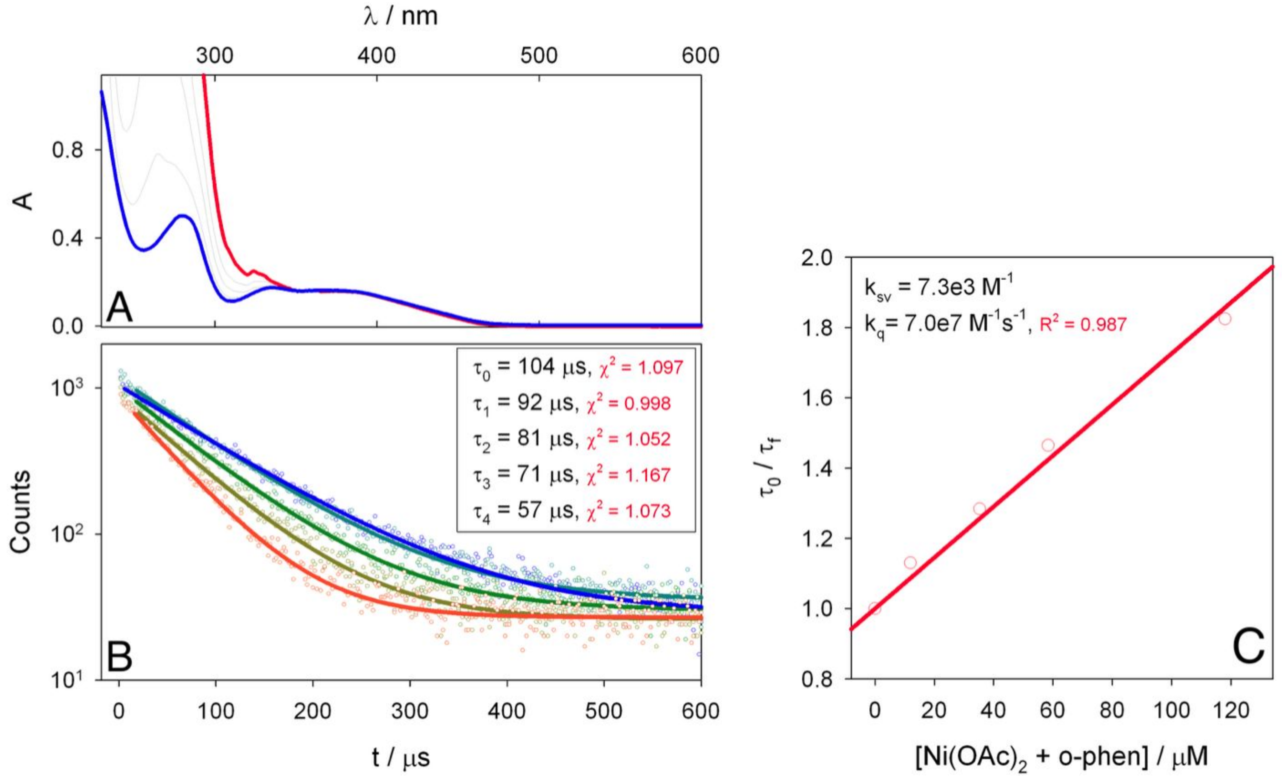

Figure S5. A) Absorption spectra of a solution of 3DPAFIPN in degassed THF at r.t. (ca. 81 μM, blue line) obtained upon addition of o-phenanthroline (ca. 29 mM). B) Delayed fluorescence decays of 3DPAFIPN obtained from the same two samples at λ_{em}= 530 nm (λ_{ex}= 475 nm) and corresponding monoexponential fitting functions. C Stern-Volmer diagram relative to the determined decay lifetimes.

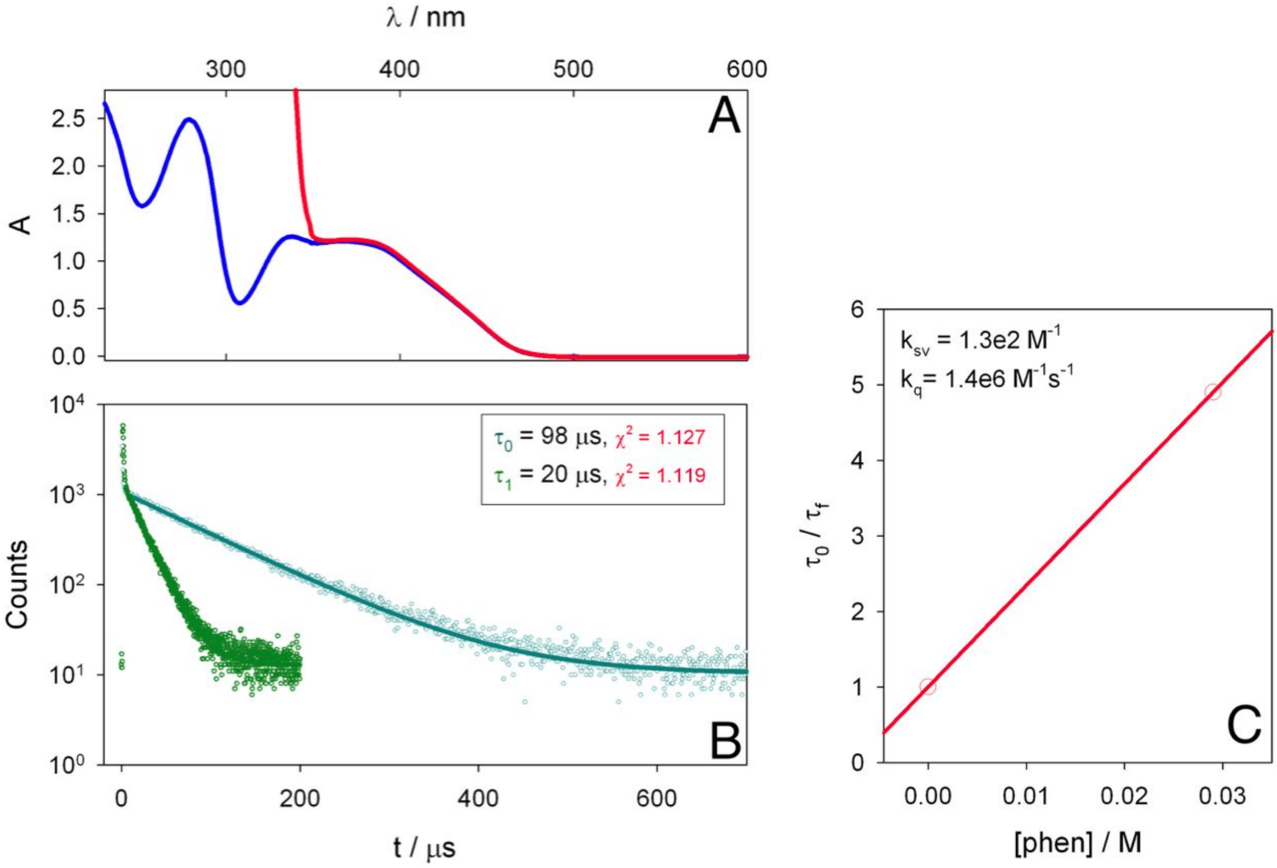

Figure S6. A) Absorption spectra of a solution of 3DPAFIPN in degassed THF at r.t. (ca. 13 μM, blue line) obtained upon addition of **2a** (ca. 220 mM, red line). B) Delayed fluorescence decays of 3DPAFIPN obtained from the same two samples at λ_{em}= 530 nm (λ_{ex}= 415 nm) and corresponding monoexponential fitting functions. As the quenching of the lifetime is negligible, the Stern-Volmer constant is not calculated.

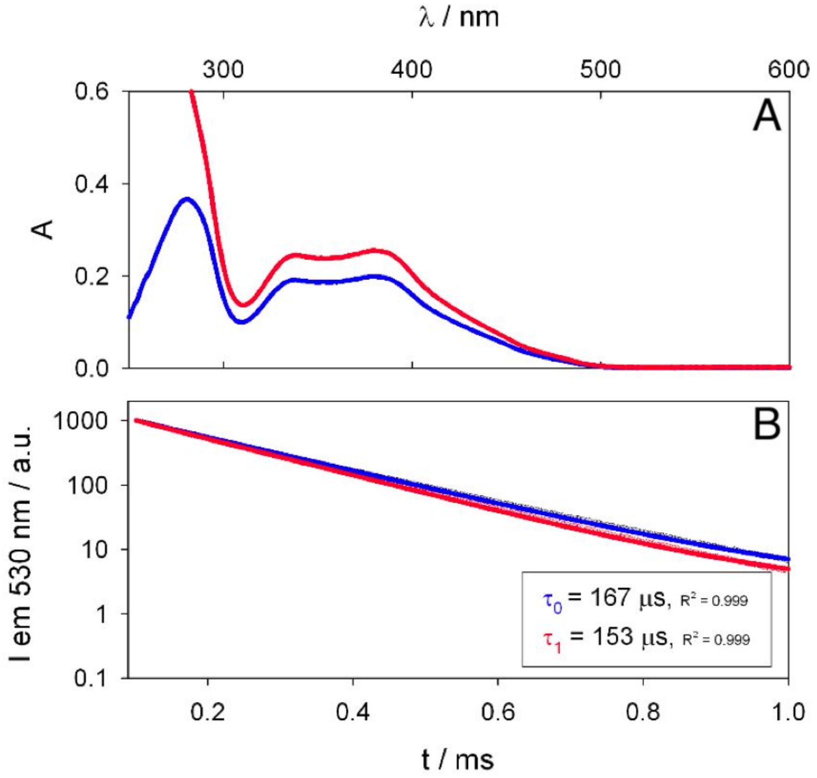

Preparation and reaction with $Ni(COD)_2$

Preparation of $Ni(COD)_2$

$NiCl_2$(pyridine)$_4$ was prepared according to a literature procedure by Percec[46] from $NiCl_2$(6 H_2O) and pyridine under refluxing conditions. The bright blue solid was collected by vacuum filtration and then allowed to air dry. A 100 mL round bottom flask with argon inlet was charged with a stirring bar and $NiCl_2$(pyridine)$_4$ (890 mg, 2 mmol). The flask was evacuated and refilled with argon three times. 1,5-Cyclooctadiene (3 equiv., 0.8 mL, 6 mmol) and THF (1 mL, distilled from Na/benzophenone) were introduced via syringes and the solution was cooled down to – 78 °C. Na (2 equiv., 98 mg) finely cut was added and the flask was evacuated and refilled with argon two times. The mixture was immediately warmed to room temperature and kept stirring vigorously at room temperature for 16 hours. Dry MeOH (1.5 mL) was added to induce the precipitation of $Ni(COD)_2$. Stirring was halted, and the yellow precipitate was allowed to settle. The black upper layer was removed by syringe and MeOH (1.5 mL) was added again to rinse the crystals, and the upper layer was removed by syringe. The procedure was repeated 3 more times until the upper layer was clear. The remaining solvent was evaporated by vacuum. The solid obtained was used in experiments by transferring under argon in another calibrated flask.
^{1}H NMR (400 MHz, C_6D_6): δ 4.10 (s, 4H), 2.0 (s, 8H).

Figure S7. Synthesis of $Ni(COD)_2$; left: reaction mixture at the beginning of the reaction; right: reaction mixture after 16 h and addition of MeOH.

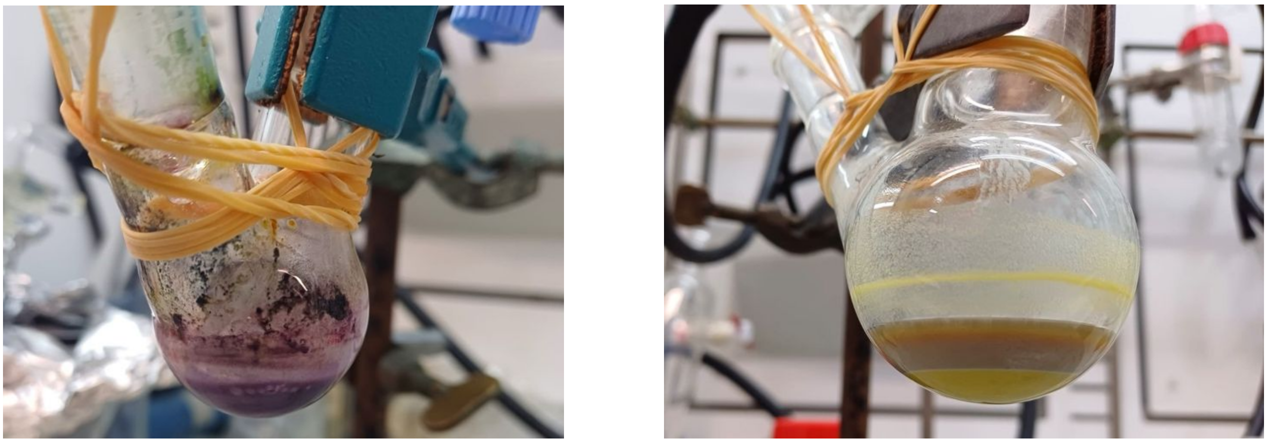

Notes. For ^{1}H NMR recording, the $Ni(COD)_2$ was transferred to another flask under argon, and C_6D_6 was directly transferred from a sealed ampoule. The resulting yellow solution was transferred by syringe into a NMR tube placed in a Schlenk flask, and the NMR tube was capped and parafilmed. The Schenck flask was opened just before the spectrum was recorded.
For all experiments with $Ni(COD)_2$, it was transferred to another calibrated flask under argon to prevent decomposition. The only experiment that gave a positive result was the photoredox reaction using $Ni(COD)_2$ as active Ni complex that was performed as the general procedure reported above.

Photocatalytic reaction with Ni(COD)$_2$

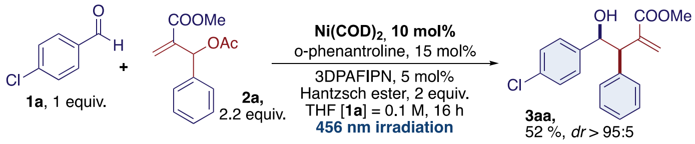

The reaction was conducted using standard condition but using Ni(COD)$_2$ instead of NiCl$_2$(glyme).

Figure S8. Photocatalytic reaction with Ni(COD)$_2$ as nickel source (Scheme 7C). Reaction mixture before (left) and during (right) irradiation.

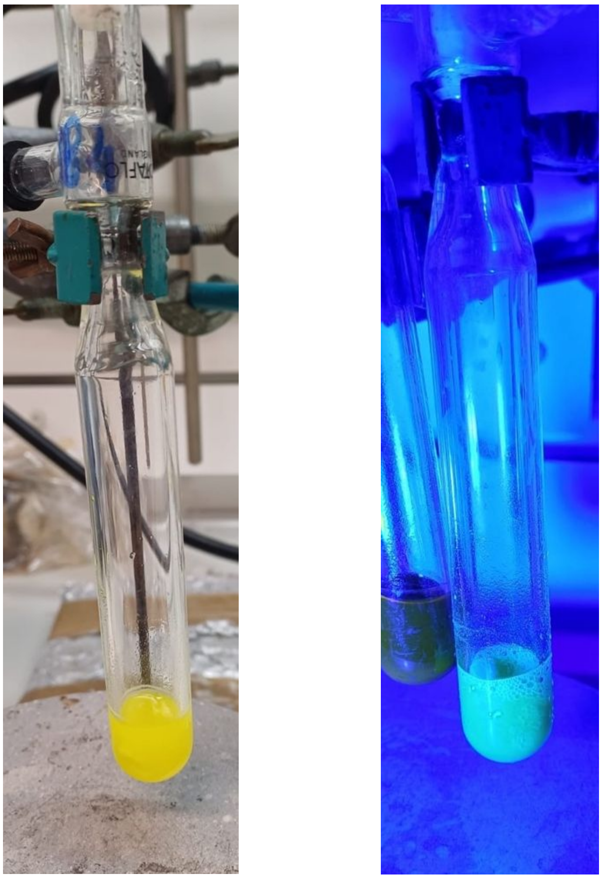

Dependence of yields in function of Baylis-Hillman adduct

Procedure used:

All the three reactions were performed on the 0.2 mmol scale of aldehyde **1**. A 0.2M solution of substrate **2** was prepared using dry THF. Three heat-gun dried 10 mL Schlenk tubes, equipped with a Rotaflo® stopcock, a magnetic stirring bar and an argon supply tube, were charged under vigorous argon flux with $NiCl_2$**.**glyme (5 mol%, 0.01 mmol, 2.2 mg), solid **1** (0.2 mmol), the organic photocatalyst 3DPAFIPN (5 mol%, 0.01 mmol, 6.5 mg), diethyl 1,4-dihydro-2,6-dimethyl-3,5-pyridinedicarboxylate Hantzsch ester (2 equiv, 0.4 mmol, 101 mg), and the ligand **L1** (7.5 mol%, 0.015 mmol, 2.8 mg). Three aliquots of **2** (0.5 mL, 1.0 mL, and 2.0 mL) were added to the flasks, and then THF was then added to the final concentration for the flasks (1.5 mL, 1.0 mL, and 0.0 mL), to obtain a 0.1 M substrate **1** solutions. The three flasks were further subjected to a freeze-pump-thaw procedure (three cycles, 5 minutes per cycle) and then refilled with argon. The degassed reaction mixtures were irradiated under vigorous stirring for 3 h. The reaction mixtures were then quenched with 1M HCl (approximately 10 mL) and extracted with EtOAc (3 x 10 mL). The combined organic layers were dried over anhydrous Na_2SO_4 and the solvent was removed under reduced pressure. To the reaction crude of the three reactions 0.4 mmol of trichloroethene was added, and the three samples were analyzed by ^{1}H-NMR to evaluate of the diastereomeric ratio and yields based on the internal standard.
The yields obtained were.
0.5 equiv of MBH adduct gave yield of 2%
1 equiv of MBH adduct gave yield of 8%
2 equiv of MBH adduct gave yield of 16%

Computational Modeling

Computational Methodology

Computations were carried out employing density functional theory (DFT) implemented in the Gaussian 16 suite of programs.[12] Geometry optimizations were carried out in tetrahydrofuran solvent (ε = 7.426) by a self-consistent reaction field (SCRF) approach using the SMD continuum solvation model[13] with the Perdew–Burke–Ernzerhof (PBE) exchange-correlation functional[14] including the Grimme's D3 empirical dispersion correction.[15] For geometry optimizations, we used Ahlrichs' split valence plus polarization basis set (def2-SVP) for all the atoms except for nickel which was treated with Ahlrichs' triple-ζ valence plus polarization basis set (def2-TZVP).[16] No symmetry constraints were imposed during structural optimizations. The frequency analyses were executed on the optimized geometries at the same level to ascertain the nature of stationary points on the potential energy surface either as minima or the transition states characterized by first-order saddle points and also to obtain the thermochemical energy values. The minima were identified by having a full set of real frequencies, whereas the transition states possess only one imaginary frequency. The transition states were searched using the linear synchronous transit (LST) method,[17] and subsequent optimizations were performed by utilizing the default Berny algorithm, implemented in the Gaussian 16 code. Intrinsic reaction coordinate (IRC) calculations were enforced to ensure that the transition state connects the corresponding real minima.[18] Furthermore, to improve the accuracy of the energies obtained from the PBE-D3(SMD,THF)/def2-TZVP(Ni)/def2-SVP level of theory, single-point calculations were performed on the optimized geometries with the M06 meta-hybrid functional[19] functional employing the def2-TZVPP basis set[xv] for all the atoms. Similar to geometry optimizations, solvation energies in tetrahydrofuran solvent were evaluated using the SMD continuum solvation model. All the energy values (ΔG) presented herein were obtained at the M06(SMD,THF)/def2-TZVPP//PBE-D3(SMD,THF)/def2-TZVP(Ni)/def2-SVP level. To enrich our understanding toward the origin of activation barriers, distortion−interaction analysis was performed.[20]The distortion−interaction model allows for partitioning the activation barrier ($\Delta^{‡}E$) of a transition state into destabilizing distortion energy ($\Delta^{‡}E_{\text{dist}}$) and stabilizing interaction ($\Delta^{‡}E_{\text{int}}$) energy terms. All the intermediates and transition states were reported in their most stable ground electronic states. Activation energy barriers ($\Delta G^{‡}_{\text{MH}}$) of single electron transfer (SET) steps were estimated using the Marcus–Hush theory.[21] Tight wave function convergence criteria and "ultrafine" (99,950) grid were used in numerical integration during all theoretical calculations.

Estimation of energy barriers of single electron transfer steps using Marcus–Hush theory

Applying the Marcus–Hush theory of electron transfer, the free energy barrier ($\Delta G^{‡}_{\text{MH}}$) of a singlet electron transfer process can be estimated according to the following equation (1):

$$\Delta G^{‡}_{\text{MH}} = \frac{(\Delta G_r + \lambda)^2}{4\lambda} \qquad (1)$$

where ΔG_r is the free energy change of the step, λ is the reorganization energy, which has two components, inner sphere and outer sphere. However, the first one is considered to be neglected, and hence, the total λ will the outer sphere reorganization energy, which can be calculated by the equation (2):

$$\lambda = \lambda_{\text{outer}} = (332\ \text{kcal/mol})\left(\frac{1}{2a_1} + \frac{1}{2a_2} - \frac{1}{\text{R}}\right)\left(\frac{1}{\varepsilon_{\text{opt}}} - \frac{1}{\varepsilon}\right) \qquad (2)$$

where a_1 and a_2 are the radii of donor and acceptor, respectively. R is the sum of a_1 and a_2. ε_{opt} and ε are the optical dielectric constant (ε_{opt} = 1.974) and static dielectric constant (ε = 7.426) of tetrahydrofuran solvent, respectively.

Table S1. Calculated free energy barriers ($\Delta G^{‡}_{\text{MH}}$) of single electron transfer (SET) steps and their relevant parameters.

SET	a_1 (Å)	a_2 (Å)	R (Å)	λ	ΔG_r	$\Delta G^{‡}_{\text{MH}}$
SET1	8.09	5.05	13.14	10.46	-26.6	6.2
SET2	8.09	4.63	12.72	11.26	-17.4	0.8
SET3	8.09	6.00	14.09	9.16	-23.4	5.5

Figure S9. Free energy profile for the oxidative addition of **2a** to the Ni(I) species **INT-C**.

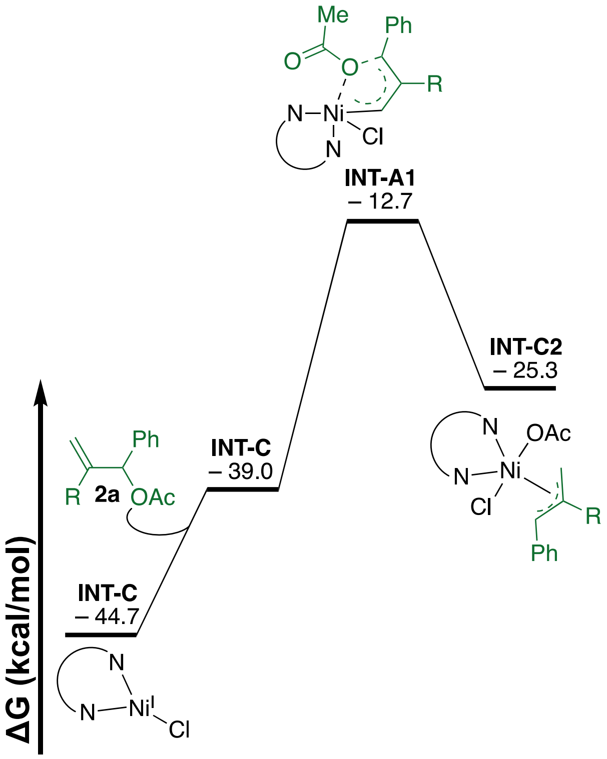

Figure S10. Energetics for the generation of Ni(0) species (**INT-D**) from **INT-C** via disproportionation reaction.

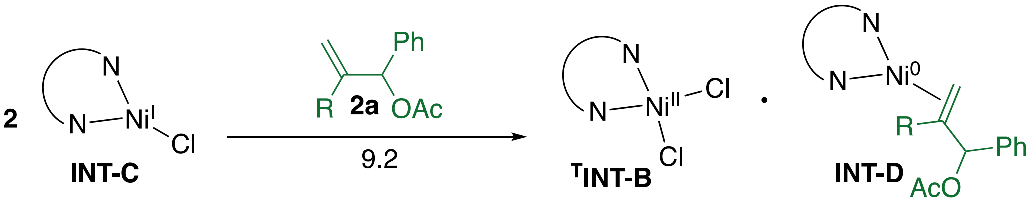

Figure S11. Comparison of energetics for the insertion step for p-chlorobenzaldehyde and 3.5-dimethoxybenzaldehyde.

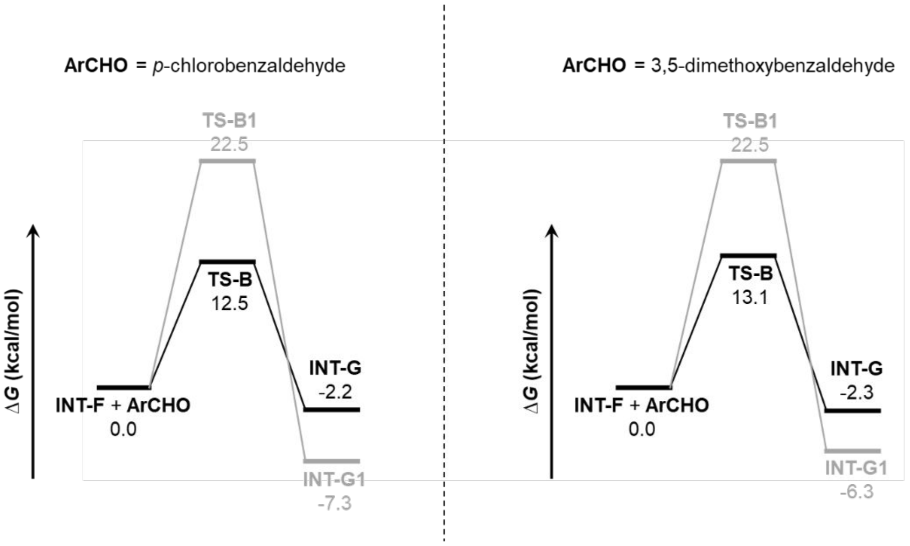

Table S2. Absolute energies (in hartree) of all the intermediates and transition states at 298.15 K. E = electronic energy at the M06(SMD,THF)/def2-TZVPP//PBE-D3(SMD,THF)/def2-TZVP(Ni)/def2-SVP level, G_{corr} = thermal correction to Gibbs free energy at the PBE-D3(SMD,THF)/def2-TZVP(Ni)/def2-SVP level, and G = Gibbs free energy at the M06(SMD,THF)/def2-TZVPP//PBE-D3(SMD,THF)/def2-TZVP(Ni)/def2-SVP level.

Species	E	G_{corr}	G	No. of imaginary frequency
**^{T}NiCl$_2$(Glyme)**	-2737.60740109	0.099836	-2737.5075650900	0
**L1**	-571.393997335	0.130996	-571.2630013350	0
**^{T}INT-A**	-3309.04895633	0.257262	-3308.7916943300	0
**Glyme**	-308.785426641	0.103860	-308.6815666410	0
**^{T}INT-B**	-3000.24659621	0.126031	-3000.1205652100	0
**3DPAFIPN•HE$^{•-}$**	-2929.96755520	0.799848	-2929.1677072000	0
**3DPAFIPN•HE**	-2929.86542870	0.797991	-2929.0674377000	0
**HE**	-862.288834823	0.248015	-862.0408198230	0
**HE$^{•+}$**	-862.092482878	0.247219	-861.8452638780	0
**HE$^{•+}$/Cl^{-}**	-1322.47366118	0.241962	-1322.2316991800	0
**INT-C**	-2540.00446800	0.127640	-2539.8768280000	0
**2a**	-804.512166387	0.190515	-804.3216513870	0
**INT-D**	-2884.28699880	0.346953	-2883.9400458000	0
**INT-C1**	-3344.53173710	0.347180	-3344.1845571000	0
**TS-A1**	-3344.48750199	0.344881	-3344.1426209900	1
**INT-C2**	-3344.50720939	0.344534	-3344.1626753900	0
**TS-A**	-2884.24885586	0.344845	-2883.9040108600	1
**^{T}INT-E**	-2884.29307368	0.343784	-2883.9492896800	0
**HE$^{•+}$/AcO^{-}**	-1090.72138091	0.285554	-1090.4358269100	0
**INT-F**	-2655.79682175	0.300507	-2655.4963147500	0
**1a**	-805.059335325	0.064686	-804.9946493250	0
**TS-B**	-3460.86164164	0.390629	-3460.4710126400	1
**TS-B1**	-3460.84303277	0.387911	-3460.4551217700	1
**INT-G**	-3460.88472547	0.390259	-3460.4944664700	0
**^{T}INT-H**	-4551.64323563	0.705063	-4550.9381726300	0
**^{T}TS-C**	-4551.60735192	0.702025	-4550.9053269200	1
**HP**	-861.096087758	0.226952	-860.8691357580	0
**AcOH**	-229.066201523	0.032578	-229.0336235230	0

**INT-I**	-3461.50156900	0.404282	-3461.0972870000	0
**3aa**	-1381.72320434	0.247840	-1381.4753643400	0

Table S3. Cartesian coordinates (Å) of the reactants, intermediates and transition states.

$^{T}NiCl_2$(Glyme)

Charge = 0; Spin multiplicity = 3

Ni	-0.036593000	-0.607783000	0.013895000
C	-0.587302000	2.210813000	0.126886000
H	-1.179640000	2.270176000	-0.814093000
H	-0.784253000	3.118796000	0.737215000
C	0.887363000	2.092166000	-0.170796000
H	1.484456000	2.044889000	0.767630000
H	1.226735000	2.961641000	-0.774550000
O	1.064551000	0.878613000	-0.924304000
C	2.429788000	0.600885000	-1.272698000
H	3.050868000	0.456725000	-0.364465000
H	2.829682000	1.434959000	-1.887404000
H	2.425647000	-0.330174000	-1.868956000
O	-0.965243000	1.036329000	0.868213000
C	-2.367918000	0.962018000	1.165412000
H	-2.524977000	0.037478000	1.750639000
H	-2.971265000	0.917961000	0.234936000
H	-2.662200000	1.843328000	1.773942000
Cl	1.506181000	-1.333808000	1.428946000
Cl	-1.644006000	-1.331081000	-1.319680000

L1

Charge = 0; Spin multiplicity = 1

N	1.385934000	-1.569634000	-0.001431000
N	-1.385929000	-1.569638000	0.001428000
C	2.716315000	-1.559885000	-0.001539000
C	0.731666000	-0.380342000	-0.000370000
C	-2.716310000	-1.559893000	0.001496000
C	-0.731665000	-0.380345000	0.000376000
C	3.499585000	-0.378378000	-0.000602000
H	3.217565000	-2.545630000	-0.002361000
C	1.424501000	0.878477000	-0.000044000
C	-3.499583000	-0.378389000	0.000545000
H	-3.217555000	-2.545640000	0.002340000
C	-1.424503000	0.878474000	0.000040000

H	4.598124000	-0.442256000	-0.000488000
C	2.844849000	0.846579000	0.000002000
C	0.686849000	2.113326000	0.000123000
C	-2.844850000	0.846571000	-0.000041000
H	-4.598122000	-0.442264000	0.000395000
C	-0.686853000	2.113324000	-0.000103000
H	3.406448000	1.794336000	0.000507000
H	1.249580000	3.060203000	0.000274000
H	-3.406454000	1.794324000	-0.000554000
H	-1.249586000	3.060200000	-0.000241000

^{T}INT-A

Charge = 0; Spin multiplicity = 3

C	-3.818838000	-0.554506000	-0.510723000
H	-3.732911000	-0.127264000	-1.536113000
H	-4.774043000	-1.124021000	-0.432203000
C	-3.819104000	0.553585000	0.509878000
H	-3.733323000	0.126417000	1.535309000
H	-4.774447000	1.122828000	0.431077000
O	-2.716460000	1.430061000	0.258290000
C	-2.652748000	2.465590000	1.236752000
H	-2.397954000	2.049602000	2.235584000
H	-3.621201000	3.013741000	1.283219000
H	-1.862260000	3.176584000	0.933790000
O	-2.715989000	-1.430675000	-0.258955000
C	-2.651774000	-2.465999000	-1.237617000
H	-1.860871000	-3.176668000	-0.934937000
H	-2.397273000	-2.049693000	-2.236393000
H	-3.619942000	-3.014652000	-1.284100000
Cl	-1.024274000	-0.219878000	2.346605000
Cl	-1.024405000	0.219846000	-2.347125000
Ni	-0.864854000	-0.000004000	-0.000272000
N	0.710930000	-1.341741000	0.094327000
N	0.710778000	1.342063000	-0.094585000
C	0.674038000	-2.661522000	0.286792000
C	1.919495000	-0.717320000	0.064444000
C	0.673784000	2.661880000	-0.286794000
C	1.919416000	0.717789000	-0.064536000
C	1.843458000	-3.444706000	0.409457000
H	-0.322265000	-3.121495000	0.359322000
C	3.156564000	-1.422167000	0.161593000

C	1.843139000	3.445211000	-0.409151000
H	-0.322539000	3.121806000	-0.359304000
C	3.156427000	1.422790000	-0.161344000
H	1.750785000	-4.529978000	0.559840000
C	3.088915000	-2.829380000	0.333206000
C	4.388996000	-0.682559000	0.082827000
C	3.088653000	2.830016000	-0.332789000
H	1.750367000	4.530495000	-0.559387000
C	4.388930000	0.683323000	-0.082358000
H	4.018390000	-3.413977000	0.412241000
H	5.336666000	-1.238195000	0.152742000
H	4.018074000	3.414729000	-0.411589000
H	5.336547000	1.239076000	-0.152070000

Glyme

Charge = 0; Spin multiplicity = 1

C	0.650105000	-0.394977000	0.000087000
C	-0.649933000	0.395412000	-0.000490000
H	0.679220000	-1.063597000	-0.897751000
H	0.678968000	-1.062852000	0.898486000
H	-0.679009000	1.064128000	0.897265000
H	-0.678748000	1.063082000	-0.899040000
O	1.722469000	0.521292000	-0.000136000
O	-1.722284000	-0.520847000	-0.000097000
C	-2.981224000	0.111484000	0.000268000
H	-3.759119000	-0.678633000	0.000463000
H	-3.133675000	0.755215000	0.902151000
H	-3.134200000	0.755257000	-0.901497000
C	2.981390000	-0.111063000	-0.000851000
H	3.759312000	0.679030000	-0.000710000
H	3.134398000	-0.755250000	0.900615000
H	3.133791000	-0.754392000	-0.903030000

^{T}INT-B

Charge = 0; Spin multiplicity = 3

Ni	1.637905000	-0.000234000	0.000017000
N	0.151301000	1.329690000	0.035534000
N	0.151030000	-1.329722000	-0.036258000
C	0.211068000	2.664538000	0.062647000
C	-1.066355000	0.717661000	0.016956000
C	0.210569000	-2.664584000	-0.063187000

C	-1.066506000	-0.717457000	-0.017949000
C	-0.955699000	3.461275000	0.075606000
H	1.216745000	3.112645000	0.072739000
C	-2.295379000	1.436037000	0.030096000
C	-0.956351000	-3.461101000	-0.076288000
H	1.216165000	-3.112877000	-0.073001000
C	-2.295664000	-1.435602000	-0.031271000
H	-0.855287000	4.555833000	0.097769000
C	-2.209049000	2.853097000	0.060000000
C	-3.526998000	0.688966000	0.013310000
C	-2.209589000	-2.852680000	-0.061029000
H	-0.856177000	-4.555682000	-0.098329000
C	-3.527138000	-0.688287000	-0.014759000
H	-3.130403000	3.455298000	0.069854000
H	-4.475670000	1.246873000	0.024150000
H	-3.131056000	-3.454709000	-0.071011000
H	-4.475923000	-1.245999000	-0.025763000
Cl	2.492041000	0.152128000	-2.045205000
Cl	2.486576000	-0.152468000	2.047757000

3DPAFIPN•HE•−

Charge = -1; Spin multiplicity = 2

N	-1.489280000	4.417322000	-2.782609000
C	-1.154423000	5.365919000	-2.155988000
C	-0.787306000	6.497693000	-1.405325000
C	-0.090890000	7.571597000	-2.027965000
C	-1.208232000	6.625868000	-0.009675000
C	0.059117000	8.806617000	-1.356207000
N	0.454169000	7.388729000	-3.327405000
C	-1.152307000	7.899386000	0.572323000
N	-1.606619000	5.492031000	0.746137000
C	-0.597908000	9.021977000	-0.067535000
C	0.887983000	9.820730000	-1.898319000
C	1.398702000	6.346523000	-3.525637000
C	0.057562000	8.268445000	-4.368356000
F	-1.635060000	8.050126000	1.826965000
C	-0.631165000	4.523667000	1.083963000
C	-2.936653000	5.454273000	1.239350000
N	-0.656388000	10.317605000	0.499682000
N	1.576138000	10.677940000	-2.331085000
C	2.300595000	6.012654000	-2.488671000

C	1.437280000	5.609758000	-4.731554000
C	0.968184000	8.682726000	-5.366691000
C	-1.262814000	8.774295000	-4.390806000
C	0.745353000	4.856987000	1.024520000
C	-0.987745000	3.200228000	1.445615000
C	-3.226807000	5.058268000	2.566302000
C	-3.999840000	5.866848000	0.402112000
C	-1.247982000	11.349805000	-0.275727000
C	-0.124179000	10.543561000	1.790636000
C	3.218529000	4.967059000	-2.657857000
H	2.277524000	6.582892000	-1.548374000
C	2.374079000	4.579550000	-4.897256000
H	0.721579000	5.841049000	-5.533728000
C	0.556726000	9.571972000	-6.369048000
H	2.005264000	8.318158000	-5.343152000
C	-1.656121000	9.680422000	-5.384766000
H	-1.978491000	8.452767000	-3.619749000
C	1.724706000	3.901761000	1.333271000
H	1.041118000	5.876736000	0.739129000
C	0.000346000	2.262035000	1.771214000
H	-2.044364000	2.899076000	1.464057000
C	-4.549583000	5.061207000	3.030101000
H	-2.407537000	4.763197000	3.237567000
C	-5.317582000	5.875212000	0.878811000
H	-3.779925000	6.175932000	-0.630813000
C	-0.740914000	12.671341000	-0.259660000
C	-2.318474000	11.041064000	-1.148202000
C	0.961160000	9.762934000	2.257211000
C	-0.685536000	11.511531000	2.659650000
C	3.269245000	4.248416000	-3.864578000
H	3.907043000	4.717008000	-1.835303000
H	2.388370000	4.014006000	-5.842237000
C	-0.753331000	10.081462000	-6.384628000
H	1.281037000	9.887919000	-7.136323000
H	-2.686151000	10.070476000	-5.379329000
C	1.364761000	2.600625000	1.720932000
H	2.787065000	4.188699000	1.276559000
H	-0.305387000	1.241046000	2.047917000
C	-5.604891000	5.467849000	2.194031000
H	-4.755264000	4.754624000	4.067938000

H	-6.131288000	6.194373000	0.208625000
C	-1.310178000	13.655131000	-1.077917000
H	0.115210000	12.918000000	0.384782000
C	-2.867718000	12.029787000	-1.976230000
H	-2.713100000	10.014305000	-1.170568000
C	1.471712000	9.953551000	3.547389000
H	1.401990000	9.003657000	1.593908000
C	-0.158990000	11.699712000	3.944596000
H	-1.547511000	12.107966000	2.327411000
H	3.992853000	3.429081000	-3.994332000
H	-1.066285000	10.789790000	-7.166852000
H	2.134399000	1.853870000	1.967018000
H	-6.641005000	5.470532000	2.565342000
C	-2.374594000	13.345016000	-1.944621000
H	-0.898702000	14.676673000	-1.053818000
H	-3.697384000	11.765671000	-2.651171000
C	0.921637000	10.925483000	4.402514000
H	2.318861000	9.335970000	3.885661000
H	-0.614842000	12.455404000	4.604093000
H	-2.807637000	14.119897000	-2.595425000
H	1.326245000	11.073334000	5.415364000
H	-1.473829000	2.555655000	-2.408880000
N	-1.382144000	1.666760000	-1.879513000
C	-0.083010000	1.225107000	-1.667168000
C	-2.449681000	1.332008000	-1.059780000
C	0.102731000	0.107135000	-0.878507000
C	0.967289000	2.104557000	-2.285040000
C	-2.312407000	0.232848000	-0.233199000
C	-3.614742000	2.278137000	-1.157003000
C	-1.121696000	-0.681710000	-0.453702000
C	1.389789000	-0.361340000	-0.350811000
H	1.409610000	2.771898000	-1.516143000
H	0.526500000	2.749935000	-3.067852000
H	1.796122000	1.516414000	-2.713343000
C	-3.199452000	-0.118579000	0.880705000
H	-3.606698000	2.795584000	-2.136116000
H	-3.558132000	3.062926000	-0.373613000
H	-4.577139000	1.756791000	-1.022057000
H	-1.367472000	-1.460252000	-1.219428000
H	-0.901920000	-1.242355000	0.473217000

O	1.514448000	-1.315815000	0.417356000
O	2.466537000	0.373097000	-0.760601000
O	-3.053209000	-1.113584000	1.591208000
O	-4.202860000	0.783798000	1.109190000
C	3.744076000	0.010506000	-0.210390000
C	-5.089303000	0.490429000	2.205435000
C	4.749560000	1.053813000	-0.654915000
H	4.023458000	-1.008210000	-0.559562000
H	3.666671000	-0.043708000	0.897192000
C	-6.196465000	1.523911000	2.197658000
H	-4.512029000	0.513844000	3.155888000
H	-5.483037000	-0.543192000	2.096397000
H	5.753002000	0.812337000	-0.249980000
H	4.824128000	1.094976000	-1.761097000
H	4.461102000	2.061479000	-0.291484000
H	-6.884958000	1.346558000	3.048573000
H	-5.786302000	2.549867000	2.291216000
H	-6.786638000	1.471895000	1.259814000

3DPAFIPN•HE

Charge = 0; Spin multiplicity = 1

N	-1.169923000	4.401003000	-2.103609000
C	-0.856512000	5.429158000	-1.628416000
C	-0.555516000	6.700409000	-1.070399000
C	-0.013786000	7.723743000	-1.896299000
C	-0.942677000	6.972457000	0.282710000
C	0.033513000	9.053273000	-1.395299000
N	0.468087000	7.420056000	-3.180986000
C	-0.967100000	8.316509000	0.704491000
N	-1.332633000	5.933172000	1.137104000
C	-0.533856000	9.380237000	-0.118360000
C	0.758630000	10.048120000	-2.113289000
C	1.411234000	6.362793000	-3.346225000
C	0.028610000	8.203868000	-4.290045000
F	-1.420271000	8.594575000	1.937494000
C	-0.475534000	4.797240000	1.268303000
C	-2.539747000	6.037123000	1.886374000
N	-0.602502000	10.707462000	0.316788000
N	1.398817000	10.851229000	-2.685540000
C	2.391341000	6.132258000	-2.355347000
C	1.374003000	5.535820000	-4.490016000

C	0.928683000	8.591933000	-5.305151000
C	-1.318477000	8.620543000	-4.356200000
C	0.925727000	4.970203000	1.251897000
C	-1.014628000	3.500580000	1.406083000
C	-2.608056000	5.565740000	3.215286000
C	-3.679264000	6.624613000	1.296010000
C	-1.089860000	11.701589000	-0.588390000
C	-0.190163000	11.063858000	1.633772000
C	3.313623000	5.087309000	-2.507516000
H	2.432189000	6.779785000	-1.466974000
C	2.317611000	4.510782000	-4.642996000
H	0.600846000	5.696814000	-5.255112000
C	0.479052000	9.383590000	-6.369969000
H	1.982894000	8.284968000	-5.246509000
C	-1.751545000	9.432220000	-5.414163000
H	-2.024303000	8.303973000	-3.573776000
C	1.771464000	3.855974000	1.355145000
H	1.349541000	5.981479000	1.160796000
C	-0.159677000	2.398485000	1.538808000
H	-2.104669000	3.359180000	1.398979000
C	-3.806446000	5.674086000	3.932906000
H	-1.718481000	5.121104000	3.684323000
C	-4.867604000	6.743566000	2.030089000
H	-3.631804000	6.980113000	0.255242000
C	-0.465877000	12.963497000	-0.674721000
C	-2.190571000	11.406601000	-1.420560000
C	0.928003000	10.430360000	2.216717000
C	-0.893039000	12.047478000	2.362441000
C	3.290060000	4.277337000	-3.654864000
H	4.067611000	4.915160000	-1.723634000
H	2.278268000	3.873263000	-5.539831000
C	-0.858165000	9.814369000	-6.428372000
H	1.190563000	9.685387000	-7.154137000
H	-2.802910000	9.757452000	-5.449970000
C	1.235480000	2.566318000	1.506487000
H	2.862565000	4.002908000	1.329132000
H	-0.591816000	1.391048000	1.634990000
C	-4.940853000	6.265046000	3.348886000
H	-3.848432000	5.303782000	4.968996000
H	-5.749672000	7.203758000	1.558362000

C	-0.943819000	13.915216000	-1.584880000
H	0.404275000	13.186967000	-0.040778000
C	-2.645406000	12.358412000	-2.344037000
H	-2.686489000	10.427560000	-1.341210000
C	1.329185000	10.771543000	3.515652000
H	1.486443000	9.674233000	1.644010000
C	-0.472134000	12.392488000	3.653474000
H	-1.771910000	12.534502000	1.915684000
H	4.020662000	3.463048000	-3.774890000
H	-1.201185000	10.446881000	-7.261146000
H	1.899471000	1.692234000	1.575585000
H	-5.877610000	6.351848000	3.920002000
C	-2.028705000	13.617511000	-2.428221000
H	-0.446536000	14.895273000	-1.650236000
H	-3.499310000	12.114402000	-2.994863000
C	0.636203000	11.756170000	4.239550000
H	2.202846000	10.269986000	3.960007000
H	-1.027959000	13.160415000	4.213606000
H	-2.390691000	14.364974000	-3.150358000
H	0.958430000	12.027733000	5.256258000
H	-1.257060000	2.410470000	-2.018304000
N	-1.119638000	1.450753000	-1.664391000
C	-2.173522000	0.881073000	-0.968109000
C	0.196096000	1.018176000	-1.580381000
C	-1.993486000	-0.366589000	-0.404675000
C	-3.401231000	1.750202000	-0.893706000
C	0.451061000	-0.204779000	-0.995186000
C	1.188011000	2.002628000	-2.131099000
C	-0.708880000	-1.130923000	-0.676889000
C	-2.974299000	-1.080298000	0.423688000
H	-4.289339000	1.226993000	-1.296583000
H	-3.250804000	2.692444000	-1.454683000
H	-3.644551000	2.000381000	0.156888000
C	1.783485000	-0.727566000	-0.664002000
H	0.716571000	2.624954000	-2.915355000
H	2.074169000	1.499423000	-2.549287000
H	1.544200000	2.690678000	-1.336211000
H	-0.458929000	-1.761146000	0.198279000
H	-0.862142000	-1.864757000	-1.508191000
O	-2.785905000	-2.206215000	0.883836000

O	-4.133616000	-0.394290000	0.655224000
O	1.991699000	-1.877321000	-0.279194000
O	2.791990000	0.190477000	-0.770782000
C	-5.119381000	-1.067782000	1.461815000
C	4.118745000	-0.267059000	-0.449903000
C	-6.303767000	-0.137663000	1.623767000
H	-4.669350000	-1.338745000	2.441480000
H	-5.407339000	-2.022153000	0.969314000
C	5.060984000	0.909213000	-0.607232000
H	4.393121000	-1.110112000	-1.121023000
H	4.127192000	-0.667934000	0.586900000
H	-7.085544000	-0.626136000	2.239712000
H	-6.006878000	0.804949000	2.127666000
H	-6.749571000	0.118961000	0.641025000
H	6.095926000	0.602887000	-0.354096000
H	5.059250000	1.289299000	-1.649552000
H	4.773054000	1.743601000	0.065097000

HE

Charge = 0; Spin multiplicity = 1

H	0.000001000	-1.608712000	0.861934000
C	0.000000000	-0.910193000	-0.006253000
C	-1.232920000	1.294692000	-0.002571000
C	1.232922000	1.294691000	-0.002570000
N	0.000001000	1.927194000	-0.003529000
C	1.274803000	-0.082608000	-0.003074000
C	-1.274803000	-0.082607000	-0.003070000
H	-0.000001000	-1.602521000	-0.879548000
H	0.000001000	2.949089000	-0.003158000
C	-2.403167000	2.242298000	-0.000668000
H	-3.050820000	2.080005000	-0.883290000
H	-2.058251000	3.295067000	-0.003162000
H	-3.045653000	2.082652000	0.886281000
C	2.403168000	2.242297000	-0.000667000
H	2.058254000	3.295066000	-0.003110000
H	3.050793000	2.080037000	-0.883319000
H	3.045684000	2.082615000	0.886252000
C	-2.499381000	-0.895622000	-0.001459000
O	-2.484283000	-2.126006000	-0.003106000
O	-3.666917000	-0.187903000	0.002167000
C	2.499381000	-0.895624000	-0.001469000

O	2.484282000	-2.126008000	-0.003079000
O	3.666918000	-0.187906000	0.002150000
C	4.879793000	-0.967385000	0.003592000
C	6.051702000	-0.008182000	0.007246000
H	4.890218000	-1.630234000	-0.888782000
H	4.886590000	-1.632518000	0.894303000
H	7.003385000	-0.576895000	0.008653000
H	6.040690000	0.642384000	-0.891120000
H	6.036751000	0.640280000	0.907075000
C	-4.879793000	-0.967382000	0.003588000
C	-6.051701000	-0.008178000	0.007228000
H	-4.886604000	-1.632517000	0.894297000
H	-4.890205000	-1.630228000	-0.888788000
H	-7.003385000	-0.576890000	0.008624000
H	-6.036761000	0.640283000	0.907058000
H	-6.040678000	0.642389000	-0.891138000

$HE^{•+}$

Charge = +1; Spin multiplicity = 2

H	0.000017000	-1.578320000	0.827811000
C	-0.000001000	-0.842823000	-0.018193000
C	-1.239058000	1.323112000	-0.015273000
C	1.239024000	1.323131000	-0.015300000
N	-0.000021000	1.920383000	-0.016430000
C	1.268654000	-0.071314000	-0.015001000
C	-1.268667000	-0.071333000	-0.014967000
H	-0.000008000	-1.569980000	-0.871696000
H	-0.000029000	2.949921000	-0.018353000
C	-2.406056000	2.258910000	-0.015446000
H	-3.050492000	2.076481000	-0.896858000
H	-2.074158000	3.314595000	-0.024598000
H	-3.040635000	2.088733000	0.875668000
C	2.406008000	2.258947000	-0.015494000
H	2.074095000	3.314628000	-0.024542000
H	3.050374000	2.076594000	-0.896974000
H	3.040663000	2.088711000	0.875554000
C	-2.500569000	-0.918315000	-0.015166000
O	-2.441641000	-2.139881000	-0.029925000
O	-3.643731000	-0.213491000	0.004172000
C	2.500566000	-0.918280000	-0.015224000
O	2.441653000	-2.139847000	-0.029971000

O	3.643723000	-0.213443000	0.003985000
C	4.879179000	-0.982956000	0.008968000
C	6.034162000	-0.010247000	0.065719000
H	4.900335000	-1.613495000	-0.904875000
H	4.859574000	-1.669018000	0.881788000
H	6.988086000	-0.574357000	0.069900000
H	6.037751000	0.664136000	-0.814314000
H	5.995942000	0.607113000	0.985903000
C	-4.879180000	-0.983018000	0.009233000
C	-6.034167000	-0.010326000	0.066181000
H	-4.859473000	-1.669143000	0.882001000
H	-4.900428000	-1.613490000	-0.904654000
H	-6.988085000	-0.574447000	0.070440000
H	-5.995846000	0.606975000	0.986401000
H	-6.037870000	0.664113000	-0.813809000

$HE^{\bullet+}/Cl^{-}$

Charge = 0; Spin multiplicity = 2

H	-0.000039000	-2.122582000	0.854858000
C	-0.000032000	-1.399725000	-0.000031000
C	-1.228147000	0.778255000	-0.000072000
C	1.228148000	0.778218000	-0.000069000
N	0.000010000	1.392972000	-0.000062000
C	1.267010000	-0.616275000	-0.000067000
C	-1.267051000	-0.616236000	-0.000058000
H	-0.000048000	-2.122682000	-0.854829000
C	-2.403128000	1.710350000	-0.000093000
H	-3.042547000	1.528007000	-0.885398000
H	-2.057542000	2.762172000	-0.000245000
H	-3.042375000	1.528242000	0.885391000
C	2.403157000	1.710278000	-0.000086000
H	2.057602000	2.762110000	-0.000098000
H	3.042482000	1.528020000	-0.885479000
H	3.042487000	1.528046000	0.885310000
C	-2.495259000	-1.457635000	-0.000062000
O	-2.445050000	-2.682252000	-0.000166000
O	-3.647561000	-0.757886000	0.000065000
C	2.495192000	-1.457710000	-0.000096000
O	2.444947000	-2.682326000	-0.000026000
O	3.647516000	-0.757997000	-0.000003000
C	4.872439000	-1.535188000	0.000056000

C	6.036541000	-0.569830000	0.000024000
H	4.873033000	-2.195571000	-0.893166000
H	4.873006000	-2.195473000	0.893352000
H	6.988211000	-1.138012000	0.000076000
H	6.021910000	0.077808000	-0.899908000
H	6.021876000	0.077914000	0.899879000
C	-4.872509000	-1.535039000	0.000093000
C	-6.036579000	-0.569644000	0.000424000
H	-4.872989000	-2.195535000	0.893231000
H	-4.873230000	-2.195211000	-0.893287000
H	-6.988268000	-1.137795000	0.000460000
H	-6.021780000	0.077889000	0.900428000
H	-6.022041000	0.078204000	-0.899359000
H	0.000026000	2.493481000	-0.000073000
Cl	0.000093000	4.360313000	-0.001216000

INT-C

Charge = 0; Spin multiplicity = 2

Ni	-1.762178000	-0.071103000	-0.000201000
N	-0.334285000	1.267751000	0.000011000
N	-0.292316000	-1.344958000	0.000003000
C	-0.410937000	2.609435000	0.000039000
C	0.911861000	0.697527000	0.000024000
C	-0.319602000	-2.690176000	0.000026000
C	0.935543000	-0.732681000	0.000020000
C	0.736584000	3.431722000	0.000069000
H	-1.424544000	3.040176000	0.000029000
C	2.123673000	1.441904000	0.000049000
C	0.855908000	-3.471007000	0.000052000
H	-1.316152000	-3.158597000	0.000017000
C	2.172242000	-1.434547000	0.000041000
H	0.608489000	4.523970000	0.000089000
C	2.008486000	2.857782000	0.000069000
C	3.367459000	0.714071000	0.000056000
C	2.107422000	-2.853634000	0.000054000
H	0.765956000	-4.567062000	0.000068000
C	3.390494000	-0.664272000	0.000052000
H	2.914613000	3.482643000	0.000087000
H	4.307926000	1.286323000	0.000067000
H	3.034628000	-3.446573000	0.000068000
H	4.349622000	-1.204689000	0.000060000

Cl	-3.910360000	0.089983000	-0.000826000

2a

Charge = 0; Spin multiplicity = 1

C	-0.815123000	-0.898102000	0.435818000
C	0.460440000	-0.419964000	-0.239616000
C	-0.832285000	-1.631167000	1.568082000
H	0.393427000	-0.668991000	-1.317119000
H	-1.782657000	-1.947748000	2.021715000
H	0.105802000	-1.936915000	2.056559000
O	1.611104000	-1.091206000	0.325324000
C	2.029938000	-2.234960000	-0.294109000
C	3.186394000	-2.843854000	0.453412000
H	3.713686000	-3.565467000	-0.195660000
H	3.882011000	-2.064758000	0.819397000
H	2.795512000	-3.384821000	1.340988000
O	1.511652000	-2.687441000	-1.297705000
C	-2.063330000	-0.483006000	-0.278647000
O	-2.065335000	0.047397000	-1.381532000
O	-3.186952000	-0.761004000	0.416097000
C	-4.421540000	-0.414888000	-0.230521000
H	-5.225667000	-0.703962000	0.470134000
H	-4.469804000	0.673850000	-0.437207000
H	-4.536606000	-0.962560000	-1.188396000
C	0.674951000	1.076939000	-0.076099000
C	0.777342000	1.909319000	-1.205993000
C	0.785795000	1.642697000	1.210023000
C	0.989480000	3.289269000	-1.054306000
H	0.682459000	1.470365000	-2.211297000
C	1.006806000	3.019700000	1.362219000
H	0.696151000	0.997407000	2.098254000
C	1.106985000	3.846740000	0.229573000
H	1.064596000	3.932428000	-1.945050000
H	1.096565000	3.451997000	2.371128000
H	1.274752000	4.928611000	0.348982000

INT-D

Charge = 0; Spin multiplicity = 1

C	1.397034000	0.856309000	-0.474578000
C	2.143167000	-0.408769000	-0.079029000
C	1.086821000	1.884458000	0.493418000

H	1.971070000	-1.172571000	-0.865220000
H	1.008394000	2.931589000	0.150815000
H	1.479985000	1.764169000	1.519233000
O	1.596119000	-0.930458000	1.169081000
C	0.834897000	-2.054950000	1.108836000
C	0.179988000	-2.307702000	2.441947000
H	-0.270518000	-3.315680000	2.461413000
H	0.906327000	-2.188614000	3.269289000
H	-0.614109000	-1.545918000	2.591240000
O	0.695657000	-2.741902000	0.110969000
C	1.326701000	1.079644000	-1.932245000
O	1.485160000	0.208813000	-2.793306000
O	1.042967000	2.375538000	-2.268130000
C	0.837463000	2.618934000	-3.662010000
H	0.642182000	3.703202000	-3.760643000
H	1.729680000	2.340647000	-4.260387000
H	-0.032751000	2.046619000	-4.047974000
Ni	-0.495787000	0.868203000	0.039580000
N	-2.031941000	1.238018000	1.141433000
N	-1.627527000	-0.444653000	-0.834885000
C	-2.200783000	2.112585000	2.149706000
C	-3.063777000	0.369387000	0.872488000
C	-1.384032000	-1.260327000	-1.874382000
C	-2.844589000	-0.543994000	-0.210182000
C	-3.379548000	2.158923000	2.926008000
H	-1.363439000	2.799438000	2.343992000
C	-4.286120000	0.343486000	1.599279000
C	-2.327102000	-2.213240000	-2.320275000
H	-0.403550000	-1.143793000	-2.364487000
C	-3.849757000	-1.478240000	-0.581855000
H	-3.453236000	2.898348000	3.736823000
C	-4.427390000	1.278908000	2.660287000
C	-5.287487000	-0.618783000	1.215583000
C	-3.557986000	-2.335332000	-1.677940000
H	-2.069030000	-2.855828000	-3.174686000
C	-5.079211000	-1.490754000	0.168519000
H	-5.353571000	1.299176000	3.254604000
H	-6.232426000	-0.641990000	1.779994000
H	-4.301307000	-3.077544000	-2.006840000
H	-5.856812000	-2.217381000	-0.113258000

C	3.645473000	-0.243024000	0.114680000
C	4.394033000	-1.301980000	0.673065000
C	4.324325000	0.923309000	-0.288802000
C	5.783536000	-1.195115000	0.828846000
H	3.878168000	-2.221873000	0.990672000
C	5.717718000	1.029926000	-0.137921000
H	3.757069000	1.760857000	-0.721804000
C	6.452245000	-0.026431000	0.422900000
H	6.350173000	-2.031130000	1.268550000
H	6.230735000	1.950418000	-0.458417000
H	7.543503000	0.058761000	0.544190000

INT-C1

Charge = 0; Spin multiplicity = 2

C	1.318190000	1.127668000	-0.847436000
C	1.843210000	-0.168207000	-0.258559000
C	0.993899000	2.245721000	-0.020585000
H	1.614215000	-0.999440000	-0.953896000
H	0.908775000	3.241214000	-0.480767000
H	1.246948000	2.214599000	1.053210000
O	1.083950000	-0.405566000	0.980907000
C	0.581414000	-1.654391000	1.202847000
C	-0.174726000	-1.695273000	2.504920000
H	0.543337000	-1.928532000	3.319471000
H	-0.644604000	-0.722100000	2.738131000
H	-0.931465000	-2.499961000	2.470828000
O	0.752657000	-2.601008000	0.455059000
C	1.416474000	1.215580000	-2.328037000
O	1.393852000	0.242960000	-3.083126000
O	1.532021000	2.488238000	-2.766695000
C	1.306572000	2.690555000	-4.166143000
H	1.525723000	3.756142000	-4.364037000
H	1.963907000	2.044801000	-4.782866000
H	0.244732000	2.476600000	-4.407712000
Ni	-0.634149000	1.177995000	-0.388803000
N	-2.076934000	1.288558000	1.010220000
N	-1.608828000	-0.511085000	-0.916104000
C	-2.286945000	2.214311000	1.952845000
C	-2.902372000	0.201216000	0.962346000
C	-1.355858000	-1.362471000	-1.915582000
C	-2.652605000	-0.765582000	-0.077214000

C	-3.323067000	2.099507000	2.907166000
H	-1.607222000	3.080518000	1.948195000
C	-3.973020000	-0.002133000	1.880439000
C	-2.123970000	-2.532545000	-2.114839000
H	-0.507034000	-1.101643000	-2.570703000
C	-3.478232000	-1.920231000	-0.194612000
H	-3.448806000	2.889293000	3.661822000
C	-4.166693000	0.992146000	2.876150000
C	-4.784366000	-1.184721000	1.748399000
C	-3.180477000	-2.818459000	-1.254349000
H	-1.871460000	-3.205131000	-2.947173000
C	-4.547366000	-2.105157000	0.751958000
H	-4.980759000	0.878605000	3.608607000
H	-5.605943000	-1.336748000	2.465143000
H	-3.788781000	-3.726764000	-1.384927000
H	-5.177417000	-3.003265000	0.660787000
C	3.332512000	-0.201129000	0.060135000
C	3.936784000	-1.442158000	0.361878000
C	4.130301000	0.959745000	0.056809000
C	5.302143000	-1.515379000	0.672265000
H	3.322412000	-2.355841000	0.348161000
C	5.499847000	0.885347000	0.362867000
H	3.679108000	1.930710000	-0.196316000
C	6.089020000	-0.349894000	0.675112000
H	5.758438000	-2.490370000	0.905225000
H	6.110303000	1.801962000	0.353847000
H	7.162638000	-0.407255000	0.913559000
Cl	-1.627348000	2.546964000	-1.969144000

TS-A1

Charge = 0; Spin multiplicity = 2

C	1.672335000	1.110209000	-1.301801000
C	2.515139000	0.021086000	-0.939535000
C	1.159853000	2.052160000	-0.345770000
H	2.485299000	-0.844250000	-1.621606000
H	0.751786000	3.009030000	-0.702367000
H	1.580452000	2.043349000	0.672407000
O	0.935634000	-0.701554000	0.464442000
C	0.845462000	-1.865014000	1.068609000
C	-0.274682000	-2.016797000	2.093319000
H	-0.008540000	-2.816069000	2.810488000

H	-0.475989000	-1.071852000	2.632170000
H	-1.209298000	-2.322184000	1.580187000
O	1.626127000	-2.798093000	0.845894000
C	1.273304000	1.141579000	-2.750018000
O	1.129715000	0.151893000	-3.461324000
O	1.156580000	2.407896000	-3.196799000
C	0.576769000	2.559201000	-4.498896000
H	0.616348000	3.639379000	-4.730499000
H	1.138451000	1.983407000	-5.262008000
H	-0.478305000	2.216717000	-4.478727000
Ni	-0.315980000	0.704568000	-0.411661000
N	-1.610180000	0.962400000	1.146651000
N	-1.606592000	-0.784987000	-0.886581000
C	-1.573299000	1.844586000	2.147219000
C	-2.690924000	0.140938000	1.028884000
C	-1.552000000	-1.630183000	-1.916904000
C	-2.689433000	-0.797682000	-0.065103000
C	-2.615524000	1.950333000	3.097456000
H	-0.685423000	2.494026000	2.199893000
C	-3.789309000	0.171324000	1.935761000
C	-2.589642000	-2.555737000	-2.177301000
H	-0.660025000	-1.555691000	-2.561245000
C	-3.783046000	-1.692127000	-0.245091000
H	-2.534505000	2.692392000	3.904857000
C	-3.724442000	1.115426000	2.994905000
C	-4.882559000	-0.744308000	1.732759000
C	-3.704243000	-2.589860000	-1.343632000
H	-2.501408000	-3.236340000	-3.036334000
C	-4.880548000	-1.638368000	0.685634000
H	-4.549122000	1.176725000	3.721687000
H	-5.726730000	-0.714559000	2.438534000
H	-4.524769000	-3.301334000	-1.524157000
H	-5.722668000	-2.332703000	0.542629000
C	3.631308000	0.057624000	-0.003631000
C	4.241188000	-1.164896000	0.389312000
C	4.196675000	1.274085000	0.465270000
C	5.347799000	-1.168962000	1.244042000
H	3.781207000	-2.107352000	0.058557000
C	5.310549000	1.263202000	1.313713000
H	3.781523000	2.234275000	0.126947000

C	5.886882000	0.043946000	1.712596000
H	5.797183000	-2.126287000	1.551240000
H	5.742015000	2.216003000	1.657952000
H	6.762859000	0.039126000	2.379984000
Cl	-1.608507000	2.112229000	-1.723098000

INT-C2

Charge = 0; Spin multiplicity = 2

C	1.935765000	1.373207000	-1.585199000
C	3.096450000	0.643702000	-1.419656000
C	0.906249000	1.643250000	-0.589307000
H	3.621792000	0.396142000	-2.358822000
H	0.304482000	2.544744000	-0.787644000
H	1.196824000	1.516353000	0.464491000
O	0.845479000	-1.090485000	-0.131768000
C	1.146673000	-1.646376000	1.018374000
C	0.552014000	-1.014884000	2.273190000
H	1.036492000	-1.440914000	3.171288000
H	0.669428000	0.086347000	2.272748000
H	-0.536219000	-1.227110000	2.321807000
O	1.871871000	-2.642839000	1.080202000
C	1.696714000	1.848941000	-2.997623000
O	2.061029000	1.269532000	-4.010046000
O	1.079355000	3.052093000	-3.008823000
C	0.666194000	3.528776000	-4.293784000
H	0.241275000	4.535966000	-4.128745000
H	1.520190000	3.585243000	-4.999199000
H	-0.110300000	2.858061000	-4.716192000
Ni	-0.438292000	0.165845000	-0.768373000
N	-1.669116000	0.845295000	0.700313000
N	-1.809602000	-1.349748000	-0.820625000
C	-1.575808000	1.958660000	1.433056000
C	-2.756092000	0.037089000	0.868265000
C	-1.814239000	-2.438128000	-1.590188000
C	-2.826437000	-1.147800000	0.056226000
C	-2.556188000	2.321716000	2.383179000
H	-0.691183000	2.591735000	1.267611000
C	-3.793210000	0.318109000	1.805138000
C	-2.856060000	-3.392424000	-1.527939000
H	-0.965631000	-2.556452000	-2.281890000
C	-3.922866000	-2.048166000	0.190170000

H	-2.424664000	3.248968000	2.959050000
C	-3.665293000	1.503712000	2.575070000
C	-4.893831000	-0.604193000	1.918275000
C	-3.912061000	-3.199216000	-0.641376000
H	-2.817850000	-4.274242000	-2.183467000
C	-4.955758000	-1.741466000	1.144877000
H	-4.441639000	1.762551000	3.311190000
H	-5.689696000	-0.378246000	2.644173000
H	-4.736870000	-3.925499000	-0.576202000
H	-5.801190000	-2.439415000	1.243709000
C	3.732519000	0.135500000	-0.209187000
C	4.557020000	-1.014761000	-0.319864000
C	3.609513000	0.739922000	1.069849000
C	5.181453000	-1.570248000	0.802514000
H	4.680417000	-1.487272000	-1.307157000
C	4.244297000	0.189153000	2.189227000
H	3.037292000	1.671894000	1.180470000
C	5.023405000	-0.975268000	2.065031000
H	5.796681000	-2.476856000	0.692505000
H	4.138468000	0.679089000	3.169987000
H	5.515591000	-1.409595000	2.949126000
Cl	-1.458334000	1.156846000	-2.524151000

TS-A

Charge = 0; Spin multiplicity = 1

C	1.576921000	1.146844000	-0.938636000
C	2.409007000	0.023319000	-0.651470000
C	1.184214000	2.090436000	0.086351000
H	2.265245000	-0.857648000	-1.297133000
H	0.709114000	3.036551000	-0.218714000
H	1.783275000	2.140623000	1.010690000
O	1.283679000	-0.975347000	1.099604000
C	1.041217000	-2.223400000	0.843238000
C	0.084100000	-2.894473000	1.836471000
H	0.114581000	-3.995397000	1.730412000
H	0.320680000	-2.602245000	2.878710000
H	-0.950283000	-2.547310000	1.627522000
O	1.491928000	-2.875417000	-0.120501000
C	0.919891000	1.139153000	-2.285533000
O	0.975522000	0.218926000	-3.097637000
O	0.282371000	2.312914000	-2.549969000

C	-0.417529000	2.364657000	-3.798002000
H	-0.834989000	3.385567000	-3.874976000
H	0.261616000	2.166591000	-4.652419000
H	-1.240488000	1.619494000	-3.822506000
Ni	-0.060283000	0.584995000	0.066203000
N	-1.564633000	1.200751000	1.053626000
N	-1.294910000	-0.769831000	-0.667911000
C	-1.662219000	2.212166000	1.938766000
C	-2.686319000	0.434834000	0.830400000
C	-1.107221000	-1.748679000	-1.567300000
C	-2.538464000	-0.634069000	-0.108781000
C	-2.860717000	2.507222000	2.619523000
H	-0.751541000	2.805159000	2.106541000
C	-3.935235000	0.661867000	1.473646000
C	-2.143659000	-2.632118000	-1.943515000
H	-0.099402000	-1.826343000	-1.998799000
C	-3.639012000	-1.480768000	-0.419933000
H	-2.873894000	3.349120000	3.327011000
C	-4.004522000	1.740356000	2.394296000
C	-5.035824000	-0.210642000	1.150778000
C	-3.409431000	-2.509016000	-1.372338000
H	-1.931421000	-3.414666000	-2.686319000
C	-4.894605000	-1.237236000	0.242416000
H	-4.948561000	1.957716000	2.916579000
H	-6.002089000	-0.036616000	1.648757000
H	-4.227407000	-3.191671000	-1.649118000
H	-5.747223000	-1.891941000	0.004893000
C	3.656054000	0.037257000	0.113600000
C	4.288164000	-1.197683000	0.418699000
C	4.320930000	1.240032000	0.461843000
C	5.523547000	-1.226286000	1.070708000
H	3.761166000	-2.127141000	0.150267000
C	5.562147000	1.206283000	1.113721000
H	3.876452000	2.207907000	0.189503000
C	6.164681000	-0.023414000	1.426895000
H	5.996278000	-2.192560000	1.306723000
H	6.068029000	2.150009000	1.370850000
H	7.138372000	-0.046789000	1.940907000

^{T}INT-E

Charge = 0; Spin multiplicity = 3

C	1.270503000	0.786823000	-0.944539000
C	2.463825000	0.138597000	-1.271238000
C	0.812546000	1.271339000	0.334064000
H	2.528389000	-0.155331000	-2.331748000
H	0.045233000	2.058731000	0.280516000
H	1.571800000	1.467722000	1.107043000
O	0.840864000	-0.283762000	3.055362000
C	1.403155000	-1.371588000	2.691316000
C	2.366416000	-2.080076000	3.613125000
H	1.824938000	-2.888041000	4.149276000
H	3.180950000	-2.551993000	3.031387000
H	2.782305000	-1.383455000	4.364647000
O	1.130152000	-1.876529000	1.548092000
C	0.342549000	0.941657000	-2.124866000
O	0.524083000	0.478265000	-3.244389000
O	-0.758182000	1.684033000	-1.829969000
C	-1.683077000	1.880075000	-2.905162000
H	-2.545249000	2.422750000	-2.475948000
H	-1.225852000	2.482491000	-3.717671000
H	-2.015275000	0.912531000	-3.332397000
Ni	-0.215843000	-0.321403000	1.203593000
N	-1.883995000	0.719764000	1.553382000
N	-1.312720000	-1.086053000	-0.305669000
C	-2.121728000	1.642439000	2.496985000
C	-2.853777000	0.475303000	0.620675000
C	-0.976363000	-2.002836000	-1.223653000
C	-2.548587000	-0.509242000	-0.378378000
C	-3.334838000	2.360544000	2.560704000
H	-1.314261000	1.810525000	3.227031000
C	-4.107661000	1.151594000	0.602766000
C	-1.853544000	-2.384579000	-2.261717000
H	0.031212000	-2.437617000	-1.130634000
C	-3.498958000	-0.825723000	-1.390603000
H	-3.477426000	3.102658000	3.359652000
C	-4.333233000	2.119714000	1.615976000
C	-5.053184000	0.816733000	-0.432155000
C	-3.116290000	-1.798482000	-2.351327000
H	-1.524648000	-3.138537000	-2.991258000
C	-4.762257000	-0.133236000	-1.386486000
H	-5.286694000	2.668999000	1.645360000

H	-6.020913000	1.341401000	-0.444741000
H	-3.814529000	-2.075987000	-3.155794000
H	-5.494283000	-0.376609000	-2.171868000
C	3.645666000	-0.191482000	-0.498233000
C	4.743133000	-0.774221000	-1.202554000
C	3.818717000	0.006298000	0.903272000
C	5.933376000	-1.125897000	-0.558945000
H	4.638767000	-0.946265000	-2.286209000
C	5.013697000	-0.344795000	1.544285000
H	3.007090000	0.425527000	1.508137000
C	6.080753000	-0.911632000	0.824478000
H	6.756164000	-1.572584000	-1.139542000
H	5.109032000	-0.178928000	2.629285000
H	7.015850000	-1.187233000	1.336379000

HE•+/AcO−

Charge = 0; Spin multiplicity = 2

H	-0.161652000	-2.676115000	-0.626492000
C	-0.149456000	-1.857584000	0.138056000
C	1.175906000	0.251075000	-0.005517000
C	-1.216698000	0.371448000	-0.237561000
N	0.015983000	0.949905000	-0.212307000
C	-1.362529000	-1.010344000	-0.024005000
C	1.156547000	-1.142122000	0.111235000
H	-0.221776000	-2.468189000	1.073348000
H	0.138326000	2.562849000	-0.486658000
C	2.395166000	1.124327000	0.100591000
H	3.249768000	0.608749000	0.565535000
H	2.129079000	2.027823000	0.686645000
H	2.712716000	1.464807000	-0.908417000
C	-2.359483000	1.318697000	-0.493327000
H	-1.973650000	2.325547000	-0.731879000
H	-3.017812000	1.388288000	0.395565000
H	-3.000380000	0.961901000	-1.321750000
C	2.333884000	-2.040649000	0.264890000
O	2.241591000	-3.173696000	0.724392000
O	3.492669000	-1.520158000	-0.194950000
C	-2.634820000	-1.773895000	0.043670000
O	-2.662664000	-2.994740000	0.166769000
O	-3.750470000	-1.013645000	-0.028849000
C	-5.012969000	-1.719557000	0.041550000

C	-6.124424000	-0.699351000	-0.070785000
H	-5.055073000	-2.281087000	0.999390000
H	-5.048925000	-2.470941000	-0.776043000
H	-7.105912000	-1.211477000	-0.015465000
H	-6.074890000	0.040763000	0.753535000
H	-6.073939000	-0.154211000	-1.035147000
C	4.665120000	-2.365920000	-0.082791000
C	5.844703000	-1.604478000	-0.645911000
H	4.474694000	-3.310863000	-0.635077000
H	4.810771000	-2.636907000	0.984607000
H	6.753946000	-2.236253000	-0.591993000
H	5.676064000	-1.333123000	-1.707713000
H	6.037172000	-0.675114000	-0.072435000
O	0.111550000	3.570075000	-0.756358000
C	0.499087000	4.339416000	0.264431000
O	0.921524000	3.903767000	1.332204000
C	0.362750000	5.814616000	-0.044625000
H	-0.702416000	6.065332000	-0.227906000
H	0.915132000	6.061681000	-0.973983000
H	0.747719000	6.420079000	0.795067000

INT-F

Charge = 0; Spin multiplicity = 2

C	1.463422000	1.709676000	-1.723860000
C	1.925862000	0.438992000	-1.218314000
C	1.097697000	2.743962000	-0.789607000
H	1.838019000	-0.396469000	-1.932587000
H	0.565679000	3.625732000	-1.180550000
H	1.698500000	2.927131000	0.114827000
C	0.985450000	1.714717000	-3.143105000
O	1.042396000	0.755215000	-3.906758000
O	0.466836000	2.914270000	-3.506675000
C	-0.078404000	2.978551000	-4.830590000
H	-0.445759000	4.012874000	-4.962300000
H	0.693286000	2.750054000	-5.594205000
H	-0.916718000	2.261369000	-4.952597000
Ni	0.049636000	1.072058000	-0.556006000
N	-1.170016000	1.364048000	0.892100000
N	-1.006704000	-0.528733000	-0.919411000
C	-1.252066000	2.378190000	1.785595000
C	-2.108885000	0.351608000	0.987294000

C	-0.889504000	-1.473111000	-1.873198000
C	-2.003487000	-0.685694000	0.022473000
C	-2.227826000	2.423151000	2.791878000
H	-0.497789000	3.173084000	1.688042000
C	-3.128307000	0.318036000	1.984540000
C	-1.733579000	-2.596519000	-1.936029000
H	-0.105138000	-1.304598000	-2.628853000
C	-2.902492000	-1.794937000	0.034644000
H	-2.229401000	3.271050000	3.492261000
C	-3.176566000	1.391068000	2.905600000
C	-4.027595000	-0.810590000	1.986118000
C	-2.743755000	-2.770750000	-0.976662000
H	-1.595953000	-3.321615000	-2.751216000
C	-3.916001000	-1.824142000	1.059254000
H	-3.945029000	1.408684000	3.693140000
H	-4.815142000	-0.847200000	2.754998000
H	-3.414079000	-3.643249000	-1.009373000
H	-4.613939000	-2.675672000	1.080047000
C	2.738921000	0.155713000	-0.035245000
C	2.765316000	-1.181116000	0.458521000
C	3.544701000	1.117296000	0.637165000
C	3.519325000	-1.527412000	1.583842000
H	2.161298000	-1.946175000	-0.055496000
C	4.301324000	0.764042000	1.762257000
H	3.602435000	2.144885000	0.250432000
C	4.289096000	-0.554711000	2.251612000
H	3.510706000	-2.567660000	1.946255000
H	4.920795000	1.528327000	2.258254000
H	4.883355000	-0.825927000	3.137845000

1a

Charge = 0; Spin multiplicity = 1

C	2.923887000	0.340161000	0.000068000
C	1.447259000	0.213649000	0.000032000
C	0.641104000	1.371152000	0.000021000
C	-0.754926000	1.267840000	-0.000040000
C	-1.338772000	-0.011849000	-0.000072000
C	-0.552896000	-1.182257000	0.000006000
C	0.838177000	-1.061245000	0.000040000
H	3.296258000	1.407758000	0.000070000
H	1.480645000	-1.955353000	0.000076000

H	1.116725000	2.365486000	0.000034000
H	-1.391693000	2.164081000	-0.000055000
H	-1.037359000	-2.169314000	0.000027000
Cl	-3.078804000	-0.155397000	-0.000065000
O	3.704808000	-0.601742000	0.000127000

TS-B

Charge = 0; Spin multiplicity = 2

C	0.390137000	-0.679333000	2.119554000
C	-1.013701000	-0.651163000	1.882123000
C	-1.812077000	0.482045000	1.556720000
C	-1.260930000	0.528415000	-0.664737000
O	0.018547000	0.652108000	-0.587562000
C	-1.909587000	-0.719736000	-1.107420000
C	-3.306601000	-0.762779000	-1.331010000
C	-3.946451000	-1.953400000	-1.683731000
C	-3.180383000	-3.126982000	-1.823423000
C	-1.788377000	-3.109878000	-1.644056000
C	-1.163420000	-1.906391000	-1.293983000
H	0.858645000	0.235946000	2.520779000
H	-0.064266000	-1.883476000	-1.163983000
H	-3.902535000	0.155266000	-1.201511000
H	-5.034581000	-1.985609000	-1.838955000
H	-1.202101000	-4.030791000	-1.776857000
Cl	-3.979654000	-4.640077000	-2.210730000
H	0.785100000	-1.602860000	2.571923000
C	-1.549750000	1.900534000	1.821293000
C	-0.263686000	2.508076000	1.860848000
C	-2.672275000	2.758907000	1.996346000
C	-0.119162000	3.886096000	2.084622000
H	0.633217000	1.907243000	1.670425000
C	-2.524781000	4.132125000	2.224793000
H	-3.682431000	2.319188000	1.956488000
C	-1.241948000	4.708537000	2.275515000
H	0.892110000	4.323665000	2.104182000
H	-3.419101000	4.760204000	2.365253000
H	-1.120771000	5.788032000	2.456318000
H	-2.883125000	0.239620000	1.472190000
C	-1.775051000	-1.942477000	1.834080000
O	-2.995021000	-2.044401000	1.792579000
O	-0.971532000	-3.047100000	1.876881000

C	-1.656266000	-4.304541000	1.846905000
H	-2.266623000	-4.406769000	0.926135000
H	-0.871772000	-5.084056000	1.867675000
H	-2.324354000	-4.419778000	2.725212000
Ni	1.237649000	-0.590131000	0.208169000
N	2.512623000	-2.037896000	0.481729000
N	2.857386000	0.389748000	-0.463577000
C	2.293893000	-3.279292000	0.955888000
C	3.801254000	-1.693571000	0.157135000
C	2.971005000	1.639601000	-0.936998000
C	3.987729000	-0.369888000	-0.353288000
C	3.331140000	-4.220352000	1.115712000
H	1.251283000	-3.514133000	1.221615000
C	4.912245000	-2.571751000	0.302868000
C	4.214683000	2.188535000	-1.315085000
H	2.031310000	2.209101000	-1.015153000
C	5.285486000	0.094444000	-0.712216000
H	3.085873000	-5.221721000	1.498715000
C	4.646057000	-3.875596000	0.796258000
C	6.218236000	-2.083601000	-0.064070000
C	5.377461000	1.422094000	-1.204903000
H	4.251576000	3.219912000	-1.695103000
C	6.398151000	-0.806493000	-0.549621000
H	5.469722000	-4.594733000	0.921758000
H	7.078441000	-2.761053000	0.050720000
H	6.356779000	1.832711000	-1.494836000
H	7.403645000	-0.452187000	-0.824625000
H	-1.852155000	1.458080000	-0.820104000

TS-B1

Charge = 0; Spin multiplicity = 2

C	-1.735278000	0.543017000	1.398929000
C	-0.586641000	1.149443000	2.044001000
C	0.630996000	0.448441000	2.320667000
C	-1.760423000	1.461589000	-0.692970000
O	-0.694223000	2.182269000	-0.764316000
C	-1.989256000	0.279360000	-1.577433000
C	-3.106997000	-0.567796000	-1.395613000
C	-3.328425000	-1.666695000	-2.235648000
C	-2.430293000	-1.915858000	-3.289338000
C	-1.329974000	-1.069857000	-3.515110000

C	-1.118547000	0.018525000	-2.660565000
H	-1.788093000	-0.552846000	1.332652000
H	-2.710575000	1.968864000	-0.391266000
H	-0.258061000	0.686639000	-2.816088000
H	-3.811102000	-0.369760000	-0.571530000
H	-4.190958000	-2.330946000	-2.079016000
H	-0.645508000	-1.268835000	-4.352812000
Cl	-2.685290000	-3.302080000	-4.334474000
H	-2.705861000	1.038552000	1.531053000
C	0.851565000	-0.979517000	2.600916000
C	-0.170861000	-1.915236000	2.905476000
C	2.188969000	-1.462369000	2.568171000
C	0.127718000	-3.269760000	3.118988000
H	-1.210645000	-1.572407000	3.002480000
C	2.483776000	-2.814874000	2.773247000
H	3.002654000	-0.751020000	2.353978000
C	1.451888000	-3.733651000	3.041980000
H	-0.687919000	-3.971029000	3.357393000
H	3.529560000	-3.157686000	2.724235000
H	1.680630000	-4.798504000	3.203283000
H	1.456228000	1.105773000	2.644191000
C	-0.545429000	2.628952000	2.254704000
O	0.428345000	3.274821000	2.636170000
O	-1.738303000	3.211213000	1.965654000
C	-1.752630000	4.641365000	1.988150000
H	-1.430752000	5.035725000	2.973753000
H	-1.082082000	5.052766000	1.205018000
H	-2.796073000	4.943450000	1.781497000
Ni	0.379328000	0.779314000	0.283078000
N	1.085761000	-0.901517000	-0.528709000
N	2.236762000	1.460521000	-0.134703000
C	0.478401000	-2.086407000	-0.696541000
C	2.414574000	-0.809494000	-0.838235000
C	2.773116000	2.664375000	0.099970000
C	3.035519000	0.470309000	-0.628932000
C	1.155986000	-3.226358000	-1.178122000
H	-0.591417000	-2.130394000	-0.438492000
C	3.183881000	-1.904853000	-1.327217000
C	4.133150000	2.945351000	-0.160381000
H	2.097303000	3.422259000	0.527603000

C	4.420339000	0.654300000	-0.911948000
H	0.600577000	-4.167936000	-1.296796000
C	2.511036000	-3.143620000	-1.496087000
C	4.580541000	-1.693215000	-1.611092000
C	4.960944000	1.944378000	-0.667798000
H	4.522603000	3.953151000	0.045142000
C	5.174333000	-0.466063000	-1.413009000
H	3.062085000	-4.019698000	-1.870882000
H	5.170238000	-2.542177000	-1.990214000
H	6.024657000	2.138797000	-0.874629000
H	6.243678000	-0.321743000	-1.631694000

INT-G

Charge = 0; Spin multiplicity = 2

C	0.394637000	-0.343365000	2.088573000
C	-0.914389000	-0.547956000	1.629348000
C	-1.697203000	0.527743000	0.911381000
C	-1.299956000	0.514157000	-0.639400000
O	0.062408000	0.597854000	-0.806462000
C	-1.946090000	-0.707977000	-1.303481000
C	-3.321236000	-0.710286000	-1.612657000
C	-3.947609000	-1.841317000	-2.152641000
C	-3.182152000	-2.996327000	-2.389780000
C	-1.807779000	-3.020133000	-2.108927000
C	-1.203911000	-1.871051000	-1.574157000
H	0.746742000	0.691619000	2.246375000
H	-0.118004000	-1.877367000	-1.363133000
H	-3.923103000	0.193007000	-1.418044000
H	-5.022149000	-1.835127000	-2.387109000
H	-1.217960000	-3.926471000	-2.310630000
Cl	-3.956269000	-4.425136000	-3.057561000
H	0.861439000	-1.108015000	2.729238000
C	-1.554421000	1.902239000	1.546485000
C	-0.597151000	2.850019000	1.123488000
C	-2.402942000	2.246461000	2.622649000
C	-0.494868000	4.101029000	1.756274000
H	0.067370000	2.583572000	0.287342000
C	-2.300322000	3.493700000	3.257663000
H	-3.156994000	1.518876000	2.965506000
C	-1.342820000	4.428646000	2.826622000
H	0.257857000	4.826550000	1.408037000

H	-2.975578000	3.739416000	4.092777000
H	-1.260464000	5.408552000	3.322745000
H	-2.763251000	0.234705000	0.975342000
C	-1.631851000	-1.830787000	1.808149000
O	-2.832130000	-2.000051000	1.613383000
O	-0.831966000	-2.846189000	2.261219000
C	-1.492651000	-4.099874000	2.470296000
H	-1.940649000	-4.478400000	1.528035000
H	-0.716872000	-4.802128000	2.828837000
H	-2.296863000	-4.009607000	3.229310000
Ni	1.178520000	-0.568013000	0.163043000
N	2.453839000	-2.009585000	0.603804000
N	2.885262000	0.357603000	-0.483048000
C	2.209094000	-3.207819000	1.167558000
C	3.752346000	-1.719497000	0.263407000
C	3.031202000	1.558317000	-1.055561000
C	3.983068000	-0.435391000	-0.330788000
C	3.222805000	-4.159280000	1.401647000
H	1.162320000	-3.397395000	1.448470000
C	4.839428000	-2.617445000	0.464192000
C	4.288810000	2.034931000	-1.487237000
H	2.105871000	2.143581000	-1.178182000
C	5.292641000	-0.044598000	-0.733628000
H	2.952659000	-5.121080000	1.861642000
C	4.543161000	-3.874804000	1.052304000
C	6.158013000	-2.199930000	0.057744000
C	5.424157000	1.239901000	-1.323591000
H	4.357596000	3.031541000	-1.947191000
C	6.376858000	-0.967626000	-0.518140000
H	5.349320000	-4.603811000	1.225450000
H	6.995487000	-2.896888000	0.215602000
H	6.415015000	1.592983000	-1.648549000
H	7.390575000	-0.667842000	-0.825775000
H	-1.798572000	1.418409000	-1.076515000

^{T}INT-H

Charge = 0; Spin multiplicity = 3

C	2.526250000	-0.745734000	2.532995000
C	2.456203000	-0.165133000	1.283942000
C	1.462776000	0.934096000	0.950588000
C	2.012832000	2.342057000	1.393644000

O	2.591913000	2.303325000	2.657950000
C	2.968346000	3.038248000	0.410777000
C	3.184780000	2.660844000	-0.927969000
C	4.121738000	3.334492000	-1.731869000
C	4.840616000	4.412144000	-1.195535000
C	4.616241000	4.839005000	0.124960000
C	3.686370000	4.148319000	0.911883000
H	3.523877000	4.450770000	1.956710000
H	2.657488000	1.800740000	-1.361676000
H	4.300069000	3.012351000	-2.768187000
H	5.177194000	5.692492000	0.533444000
Cl	6.043742000	5.229203000	-2.177005000
C	0.092273000	0.649260000	1.559224000
C	-0.313927000	1.186003000	2.799660000
C	-0.797129000	-0.205623000	0.872931000
C	-1.577832000	0.880322000	3.332621000
H	0.370879000	1.842088000	3.358904000
C	-2.057847000	-0.515459000	1.406454000
H	-0.492514000	-0.633010000	-0.096628000
C	-2.453579000	0.027677000	2.641185000
H	-1.869468000	1.305799000	4.304801000
H	-2.737522000	-1.181472000	0.851588000
H	-3.442784000	-0.211931000	3.062188000
H	1.338442000	0.931192000	-0.148812000
C	3.322724000	-0.667826000	0.173348000
O	3.098685000	-0.483145000	-1.017251000
O	4.379893000	-1.387246000	0.618061000
C	5.256188000	-1.909363000	-0.390796000
H	5.657314000	-1.094997000	-1.027823000
H	6.079642000	-2.410252000	0.149758000
H	4.728106000	-2.638938000	-1.038483000
Ni	3.835478000	1.010311000	3.184568000
N	4.705211000	-0.369651000	4.451643000
N	5.656345000	0.851214000	2.276676000
C	4.182777000	-0.953376000	5.535909000
C	5.996686000	-0.634098000	4.113178000
C	6.079882000	1.465099000	1.168363000
C	6.505085000	0.014401000	2.933912000
C	4.940994000	-1.832360000	6.345326000
H	3.126081000	-0.725283000	5.766596000

C	6.831937000	-1.514271000	4.859071000
C	7.382556000	1.276405000	0.653898000
H	5.360511000	2.129972000	0.670125000
C	7.838564000	-0.235265000	2.497288000
H	4.467702000	-2.283868000	7.229143000
C	6.261842000	-2.116410000	6.012270000
C	8.179245000	-1.741683000	4.404029000
C	8.265262000	0.428001000	1.315973000
H	7.675326000	1.810860000	-0.261359000
C	8.661435000	-1.131393000	3.267783000
H	6.867904000	-2.801452000	6.625058000
H	8.819124000	-2.422245000	4.986291000
H	9.287000000	0.266056000	0.939373000
H	9.691771000	-1.317162000	2.928252000
H	1.108551000	3.000949000	1.443175000
O	1.015602000	-0.375143000	6.047391000
C	0.327827000	0.613329000	6.343947000
C	0.849462000	1.978235000	6.359540000
O	-0.993977000	0.476823000	6.652169000
C	2.293385000	2.070561000	5.929590000
C	0.162282000	3.135826000	6.714091000
C	-1.528101000	-0.860554000	6.590283000
H	2.440549000	1.406947000	5.047529000
C	2.736079000	3.472338000	5.581379000
H	2.968199000	1.621578000	6.701569000
N	0.727156000	4.381074000	6.561748000
C	-1.246543000	3.169367000	7.257556000
C	-3.003647000	-0.788909000	6.923464000
H	-0.974160000	-1.509932000	7.302450000
H	-1.354156000	-1.275490000	5.574004000
C	1.931302000	4.560597000	5.953476000
C	3.994409000	3.535549000	4.881739000
H	0.132362000	5.517661000	7.240094000
H	-1.468675000	4.172031000	7.667460000
H	-1.406804000	2.403757000	8.038396000
H	-1.984099000	2.953474000	6.456408000
H	-3.445741000	-1.805110000	6.890998000
H	-3.167138000	-0.373641000	7.938852000
H	-3.549851000	-0.153115000	6.196869000
C	2.295934000	6.003785000	5.707453000

O	4.661446000	2.510041000	4.582668000
O	4.476227000	4.763988000	4.557877000
O	-0.353838000	6.362973000	7.774808000
H	2.496435000	6.182873000	4.633116000
H	3.222722000	6.283483000	6.245847000
H	1.474032000	6.663875000	6.037443000
C	5.762731000	4.808114000	3.905418000
C	0.379644000	6.748033000	8.811252000
C	6.038824000	6.248130000	3.526838000
H	5.750550000	4.141584000	3.017742000
H	6.535108000	4.409030000	4.598519000
O	1.475637000	6.275889000	9.110272000
C	-0.284103000	7.860072000	9.610492000
H	7.021096000	6.321038000	3.018038000
H	5.262456000	6.638049000	2.837078000
H	6.063609000	6.902265000	4.421941000
H	-1.261232000	7.513052000	10.005721000
H	-0.496548000	8.727992000	8.952914000
H	0.363820000	8.175906000	10.448428000
H	3.181198000	-1.611492000	2.698649000
H	1.751586000	-0.551403000	3.293194000

HP

Charge = 0; Spin multiplicity = 1

C	0.000001000	-0.731048000	0.000198000
C	-1.181521000	1.376209000	0.000254000
C	1.181516000	1.376213000	-0.000029000
N	-0.000003000	2.025027000	0.000083000
C	1.225610000	-0.049755000	0.000083000
C	-1.225610000	-0.049758000	0.000247000
C	-2.392792000	2.268155000	0.000477000
H	-3.033379000	2.082004000	-0.884463000
H	-2.056514000	3.321255000	0.000766000
H	-3.033461000	2.081504000	0.885245000
C	2.392785000	2.268162000	-0.000329000
H	2.056505000	3.321262000	-0.000601000
H	3.033363000	2.081526000	-0.885173000
H	3.033469000	2.081991000	0.884529000
C	-2.448840000	-0.919434000	0.000312000
O	-2.405555000	-2.142092000	0.000704000
O	-3.603566000	-0.222774000	-0.000192000

C	2.448842000	-0.919428000	-0.000020000
O	2.405560000	-2.142085000	-0.000580000
O	3.603567000	-0.222765000	0.000449000
C	4.826625000	-1.001361000	0.000169000
C	5.992167000	-0.037318000	-0.000022000
H	4.828340000	-1.661587000	-0.893070000
H	4.828697000	-1.661621000	0.893387000
H	6.943435000	-0.606473000	-0.000197000
H	5.978676000	0.610726000	-0.899783000
H	5.979003000	0.610704000	0.899760000
C	-4.826624000	-1.001372000	-0.000148000
C	-5.992166000	-0.037330000	-0.000336000
H	-4.828561000	-1.661508000	0.893157000
H	-4.828471000	-1.661721000	-0.893300000
H	-6.943434000	-0.606484000	-0.000365000
H	-5.978914000	0.610789000	0.899375000
H	-5.978763000	0.610618000	-0.900168000
H	0.000001000	-1.830807000	0.000208000

AcOH

Charge = 0; Spin multiplicity = 1

O	0.774592000	-1.049837000	0.000140000
C	0.091592000	0.122317000	-0.000051000
O	0.650648000	1.205400000	-0.000121000
C	-1.397390000	-0.104344000	0.000014000
H	-1.926442000	0.864384000	-0.001281000
H	-1.691484000	-0.696642000	-0.890279000
H	-1.691582000	-0.694137000	0.891956000
H	1.729285000	-0.808160000	0.000230000

INT-I

Charge = 0; Spin multiplicity = 1

C	2.808735000	-1.211692000	3.113299000
C	2.468592000	-0.791670000	1.772959000
C	1.423481000	0.276086000	1.425355000
C	1.330561000	1.515677000	2.355794000
O	0.906297000	1.117885000	3.653558000
C	2.621266000	2.328196000	2.377635000
C	2.983949000	3.107158000	1.259994000
C	4.164701000	3.862168000	1.252283000
C	4.983803000	3.856772000	2.395095000

C	4.633784000	3.116426000	3.533074000
C	3.464082000	2.338423000	3.508026000
H	3.177495000	1.755364000	4.394436000
H	2.334187000	3.120345000	0.369861000
H	4.450946000	4.454878000	0.371617000
H	5.284441000	3.123647000	4.419258000
Cl	6.465550000	4.797800000	2.396845000
C	0.044059000	-0.342728000	1.213882000
C	-0.563216000	-1.150905000	2.198855000
C	-0.659934000	-0.106538000	0.014863000
C	-1.838878000	-1.698813000	1.991602000
H	-0.023387000	-1.351656000	3.135172000
C	-1.935861000	-0.655031000	-0.197486000
H	-0.191981000	0.512174000	-0.768166000
C	-2.531518000	-1.453268000	0.792786000
H	-2.296700000	-2.326466000	2.772912000
H	-2.464605000	-0.462058000	-1.144587000
H	-3.530581000	-1.887490000	0.629708000
H	1.728582000	0.689600000	0.443407000
C	2.826303000	-1.623080000	0.600232000
O	2.676678000	-1.286296000	-0.579584000
O	3.366335000	-2.839672000	0.925917000
C	3.855861000	-3.607972000	-0.173784000
H	4.684933000	-3.083658000	-0.695856000
H	4.229502000	-4.557550000	0.253687000
H	3.056742000	-3.817380000	-0.914949000
Ni	4.273342000	-0.220781000	2.312793000
N	5.821008000	-0.022842000	3.462880000
N	5.392731000	0.597161000	0.944453000
C	5.997891000	-0.345015000	4.756824000
C	6.840954000	0.643149000	2.827698000
C	5.144876000	0.859969000	-0.349938000
C	6.609364000	0.975665000	1.452664000
C	7.173016000	-0.018499000	5.468071000
H	5.167908000	-0.879800000	5.242889000
C	8.060947000	1.010589000	3.460455000
C	6.074633000	1.527337000	-1.178034000
H	4.173915000	0.508686000	-0.736301000
C	7.603510000	1.658140000	0.698477000
H	7.253123000	-0.308319000	6.526030000

C	8.210439000	0.660035000	4.829498000
C	9.050078000	1.712376000	2.682650000
C	7.302255000	1.939216000	-0.661326000
H	5.810803000	1.717068000	-2.228840000
C	8.831352000	2.020779000	1.357876000
H	9.134237000	0.924123000	5.366550000
H	9.992258000	2.000522000	3.173911000
H	8.035961000	2.469501000	-1.287507000
H	9.597160000	2.558307000	0.777594000
H	0.551868000	2.165158000	1.886529000
H	3.110603000	-2.261664000	3.277176000
H	2.275432000	-0.741071000	3.954502000
H	0.688858000	1.931689000	4.148355000

3aa

Charge = 0; Spin multiplicity = 1

C	2.636702000	-1.169772000	3.065577000
C	2.296083000	-0.831879000	1.800940000
C	1.273514000	0.223522000	1.414464000
C	1.236270000	1.491474000	2.315554000
O	0.775552000	1.135464000	3.611022000
C	2.582107000	2.211581000	2.332870000
C	3.037404000	2.878767000	1.176906000
C	4.280375000	3.524845000	1.153504000
C	5.079758000	3.509136000	2.309675000
C	4.646010000	2.862795000	3.477026000
C	3.399603000	2.217206000	3.478150000
H	3.052723000	1.709655000	4.390112000
H	2.408219000	2.898340000	0.272169000
H	4.629447000	4.042931000	0.248480000
H	5.279598000	2.862966000	4.375861000
Cl	6.636484000	4.316061000	2.293754000
C	-0.125327000	-0.363082000	1.231382000
C	-0.720234000	-1.186269000	2.209131000
C	-0.868840000	-0.044021000	0.076278000
C	-2.026256000	-1.671720000	2.038442000
H	-0.151649000	-1.453469000	3.111870000
C	-2.174593000	-0.530576000	-0.099083000
H	-0.411347000	0.591812000	-0.699265000
C	-2.758709000	-1.346307000	0.883796000
H	-2.475096000	-2.313311000	2.813236000

H	-2.736012000	-0.274807000	-1.011567000
H	-3.781243000	-1.732393000	0.748584000
H	1.584869000	0.589932000	0.416388000
C	2.922817000	-1.522498000	0.623006000
O	2.758167000	-1.178065000	-0.540493000
O	3.697872000	-2.572715000	0.974765000
C	4.348971000	-3.257556000	-0.104425000
H	5.027800000	-2.576737000	-0.658355000
H	4.929846000	-4.076348000	0.357941000
H	3.607847000	-3.673648000	-0.817435000
H	0.501767000	2.172320000	1.821079000
H	3.385737000	-1.952712000	3.255181000
H	2.175537000	-0.663821000	3.925143000
H	0.601061000	1.964036000	4.098564000

NMR Traces

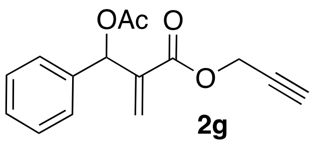

2g

¹H NMR (400 MHz, $CDCl_3$)

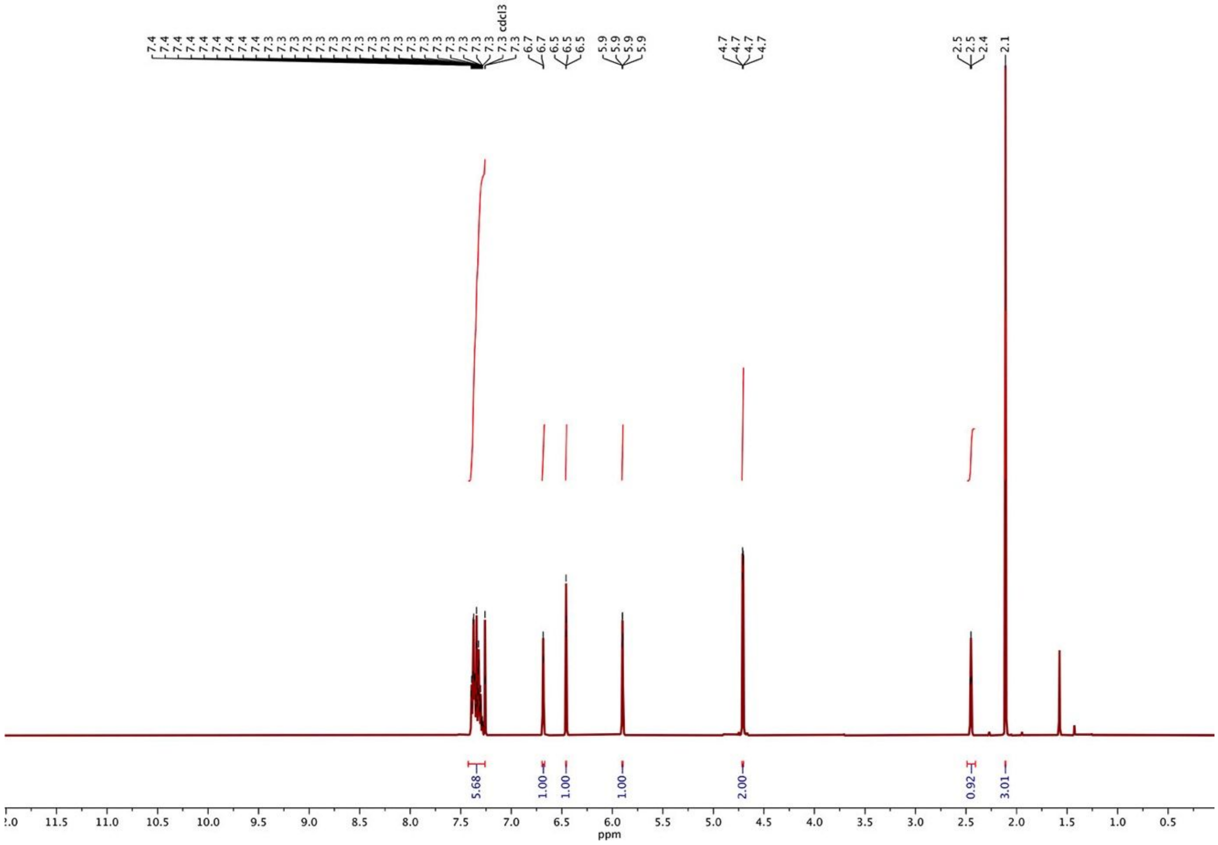

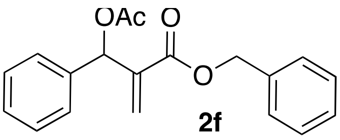

2f

^{1}H NMR (400 MHz, $CDCl_3$)

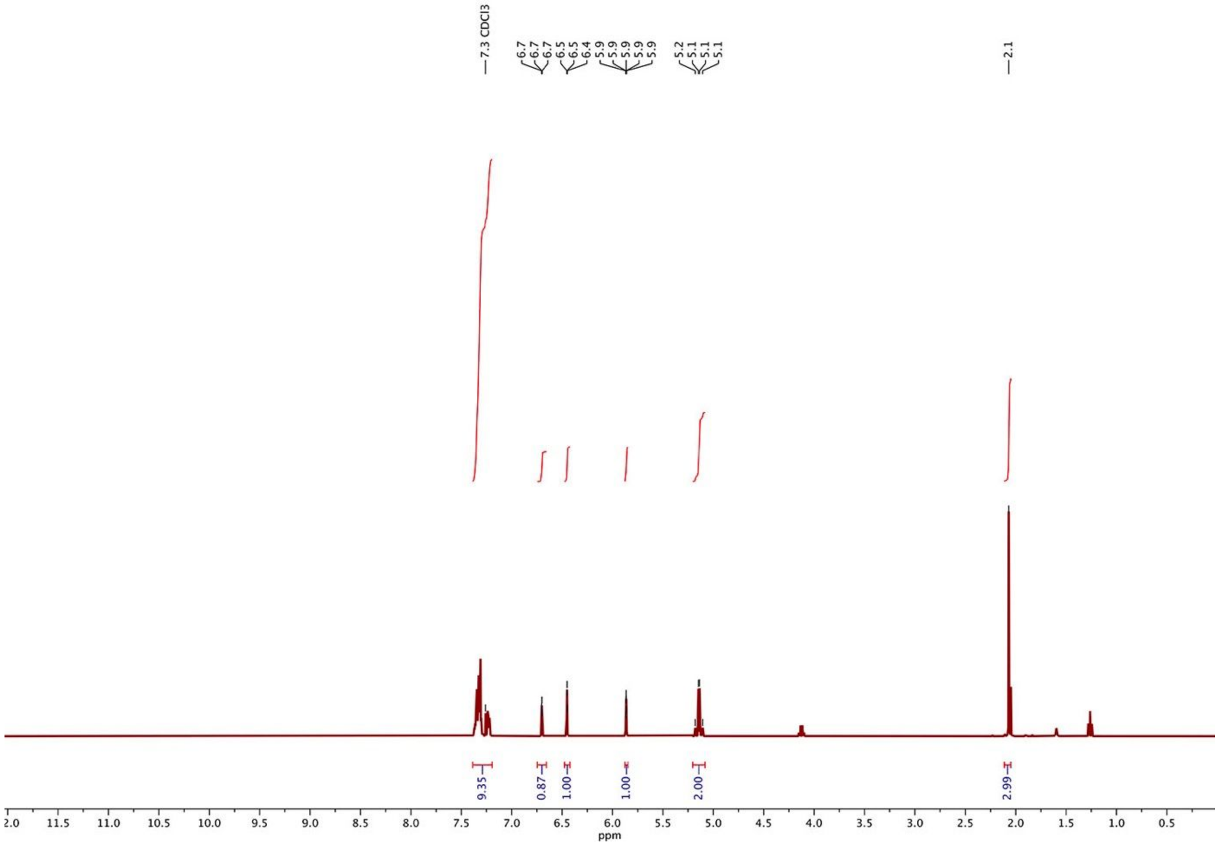

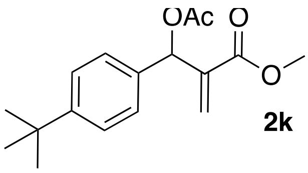

2k

^{1}H NMR (400 MHz, $CDCl_3$)

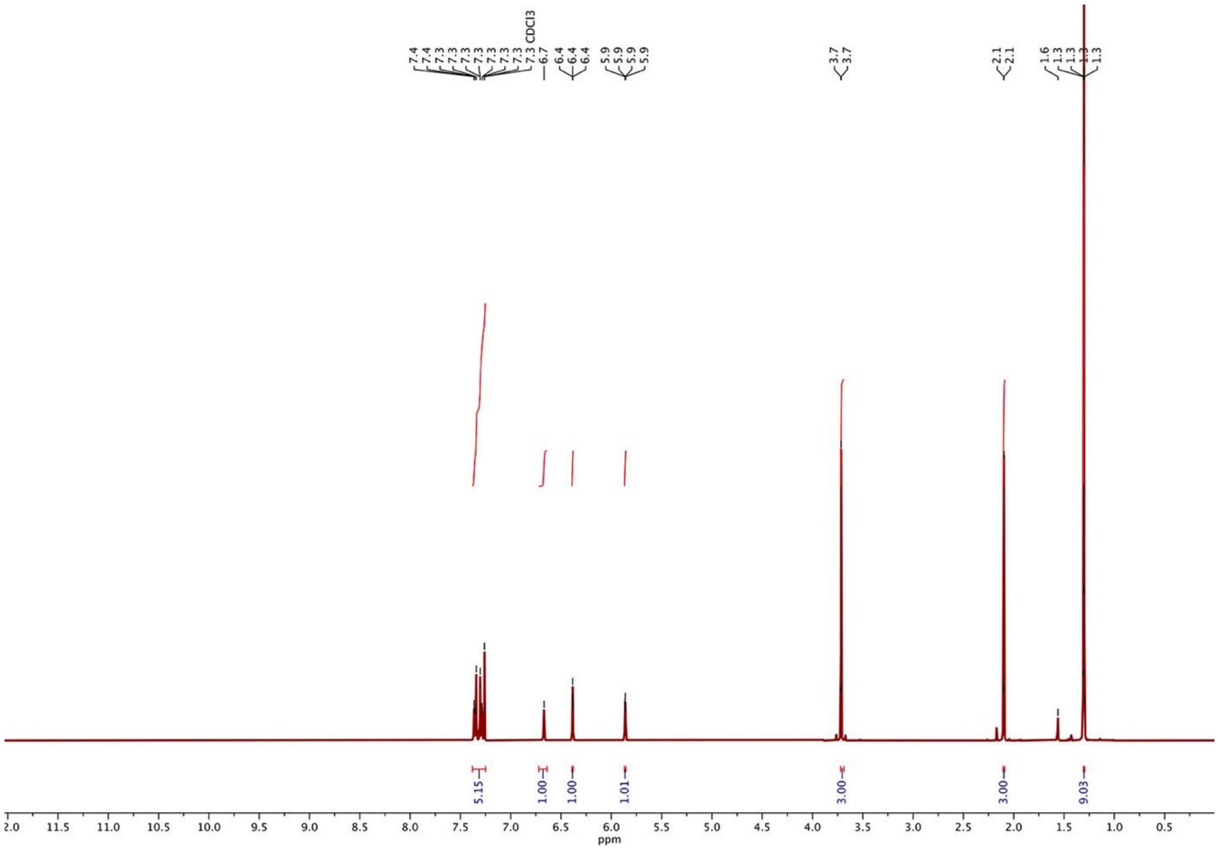

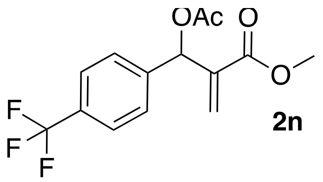
2n

^{1}H NMR (400 MHz, $CDCl_3$)

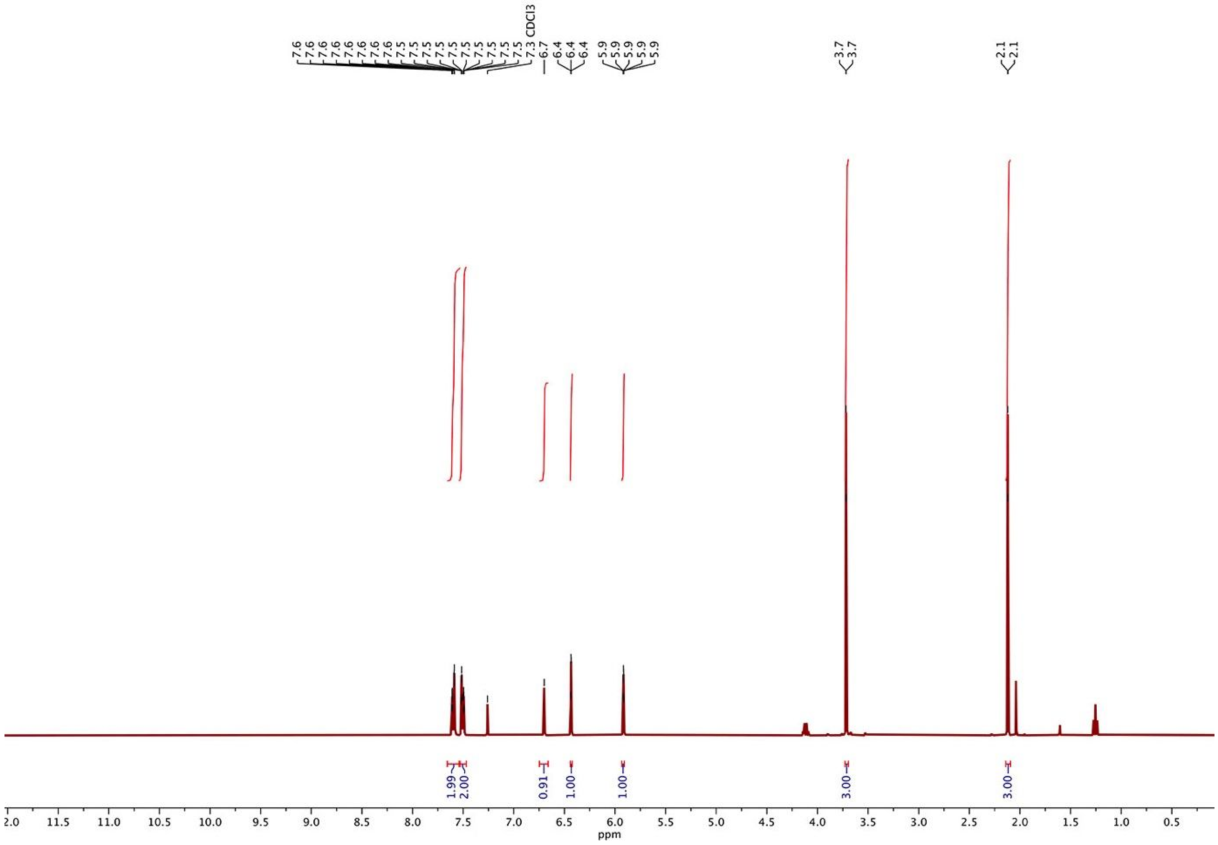

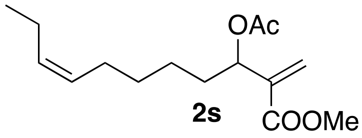

2s

¹H NMR (400 MHz, $CDCl_3$)

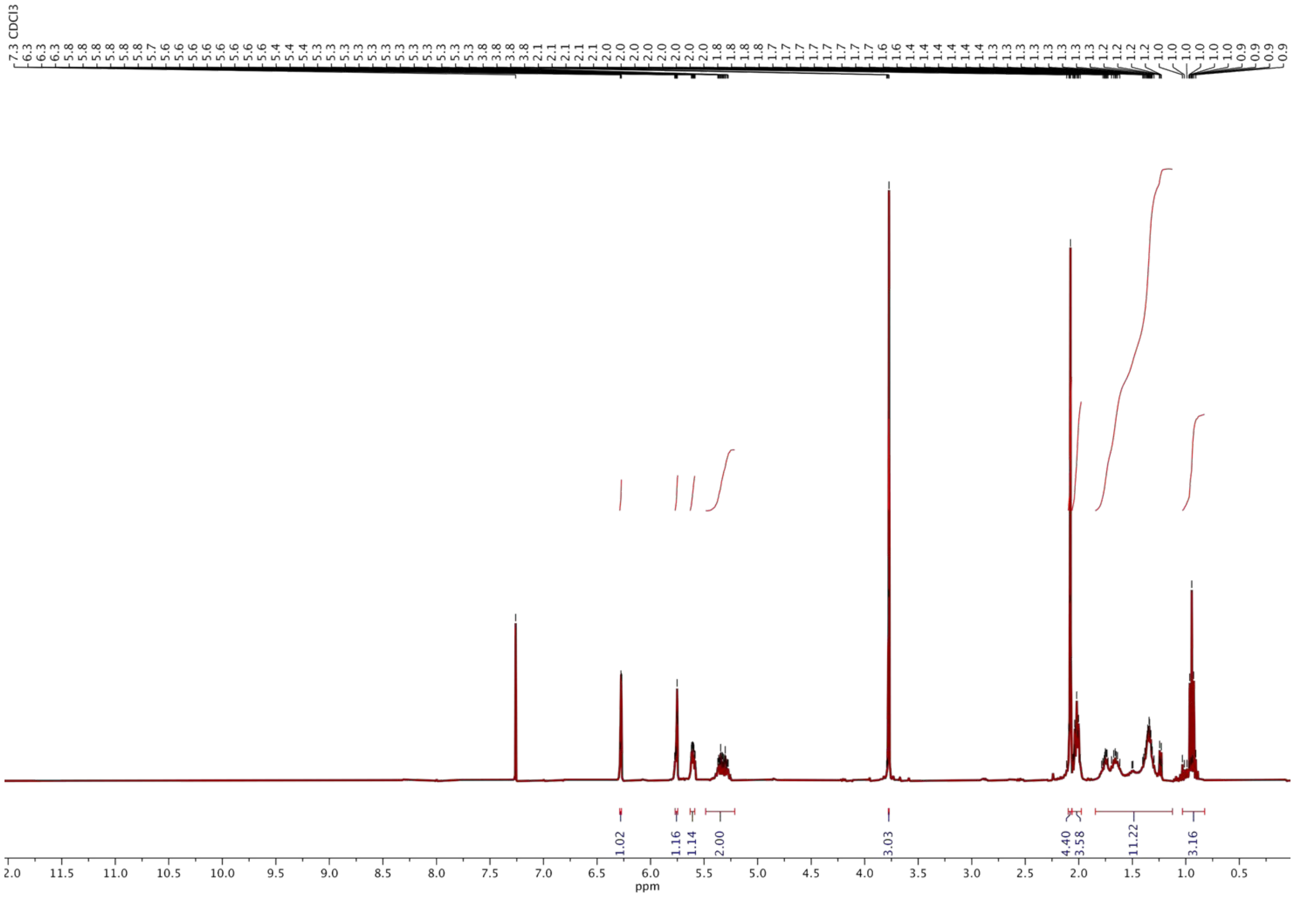

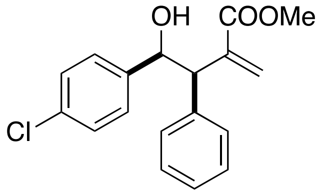

3aa, 87% *dr* > 95:5

^{1}H NMR (400 MHz, $CDCl_3$)

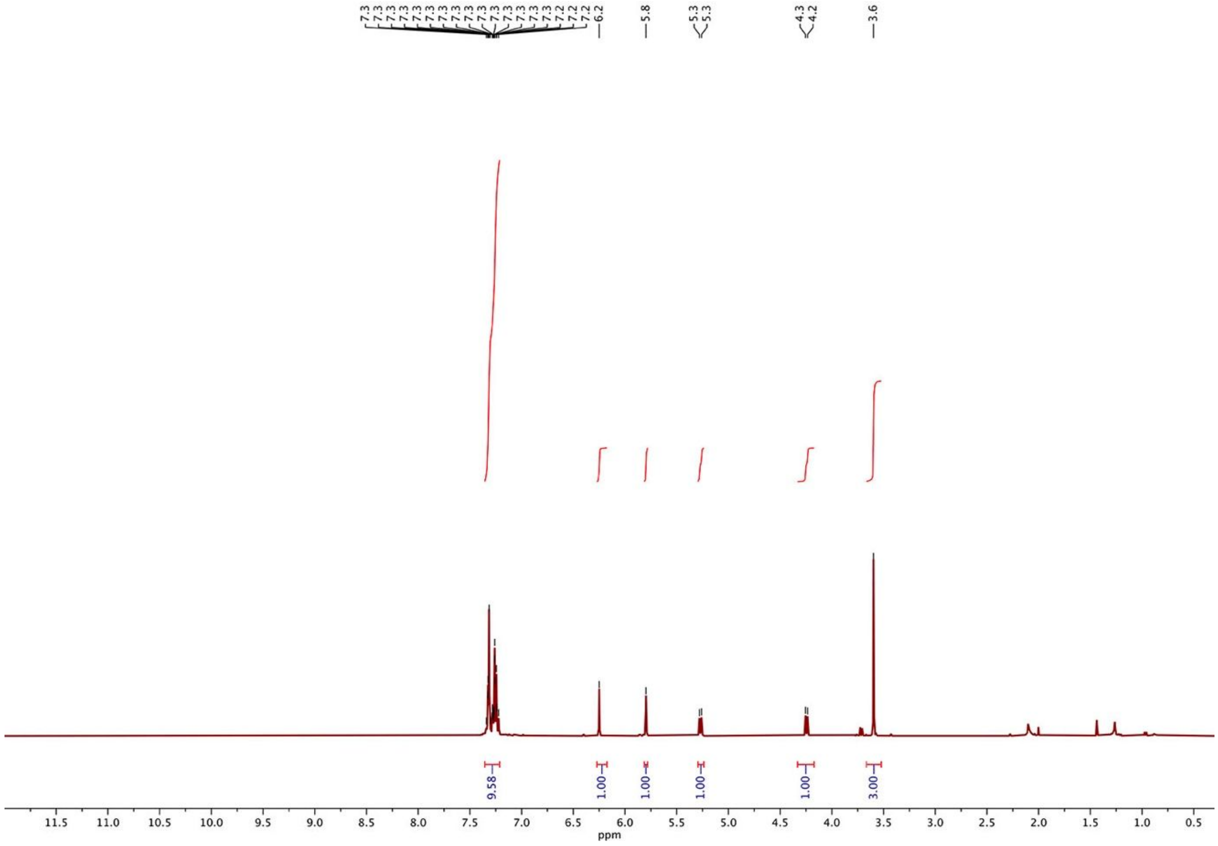

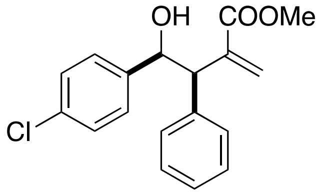

3aa, 87% *dr* > 95:5

^{13}C{^{1}H} NMR (100 MHz, CDCl$_3$)

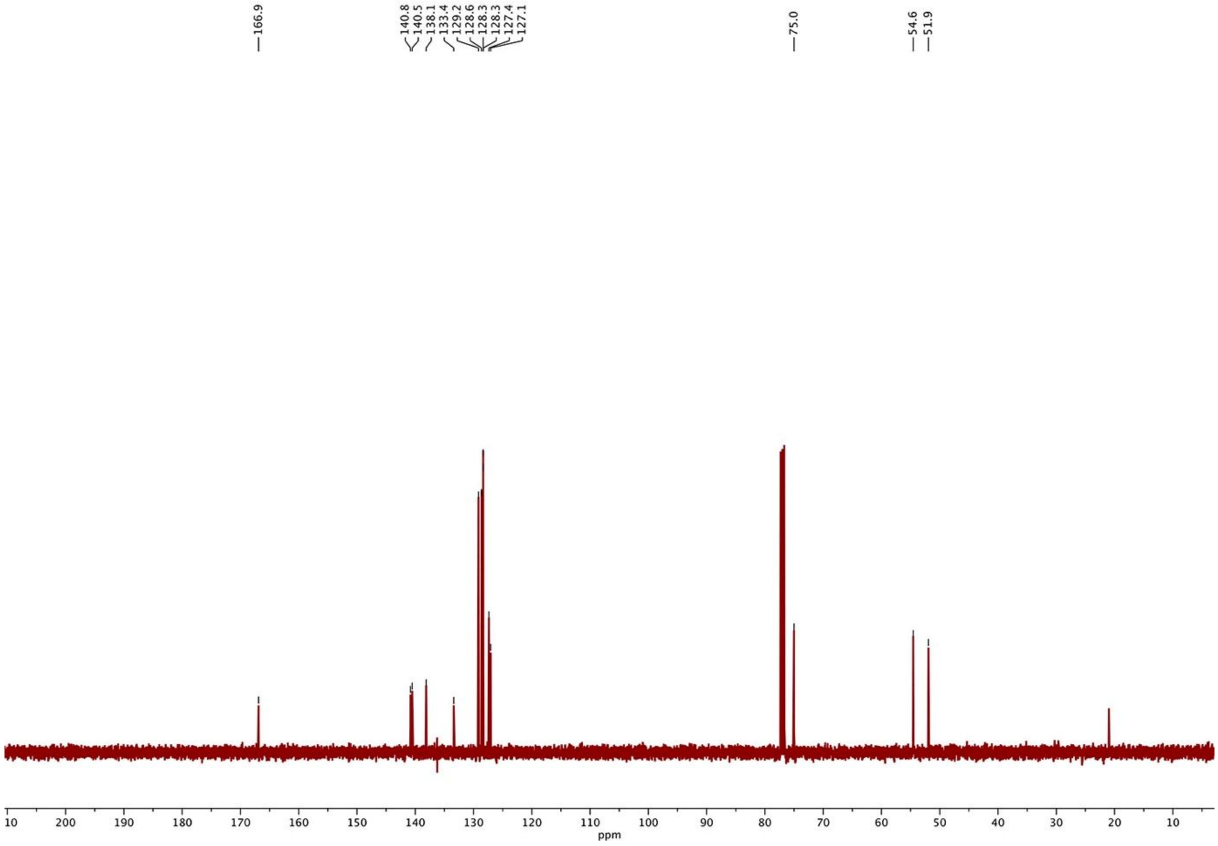

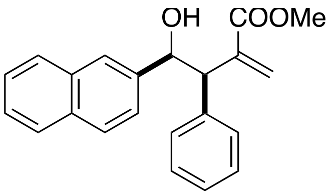

3ba, 77% *dr* > 95:5

^{1}H NMR (400 MHz, $CDCl_3$)

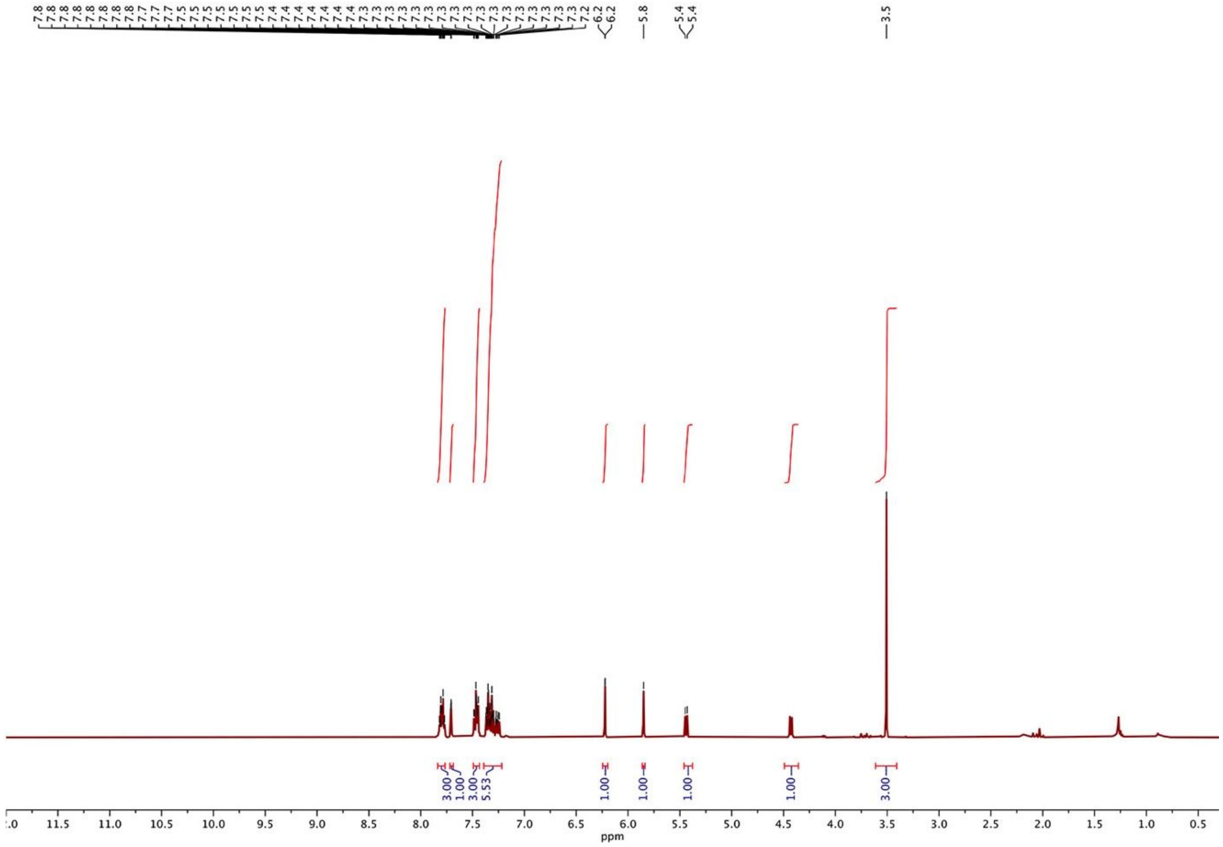

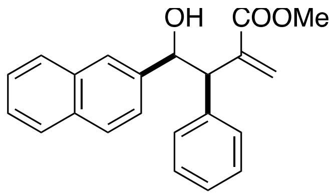

3ba, 77% *dr* > 95:5

^{13}C{^{1}H} NMR (100 MHz, $CDCl_3$)

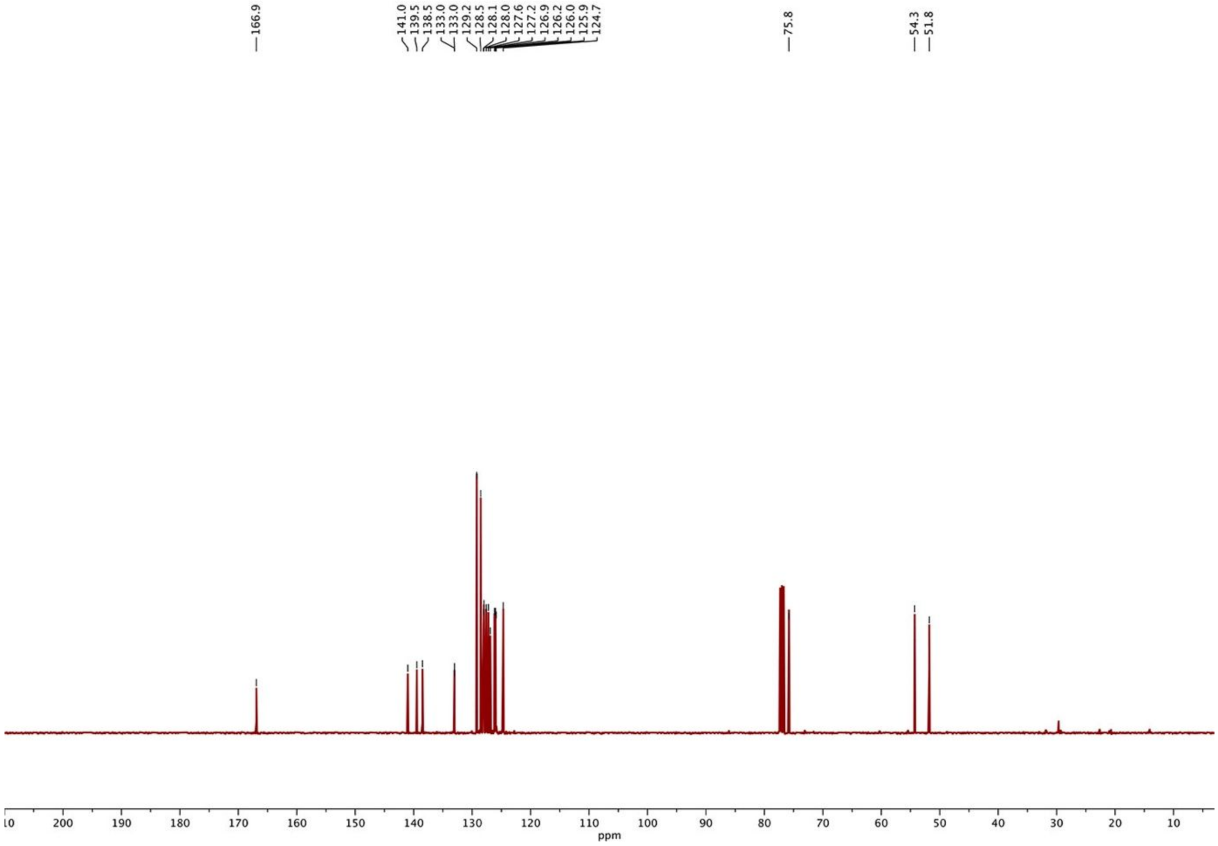

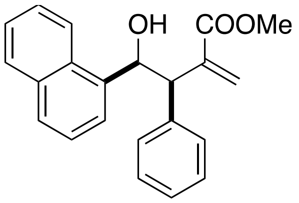

3ca, 68% *dr* > 95:5

^{1}H NMR (400 MHz, $CDCl_3$)

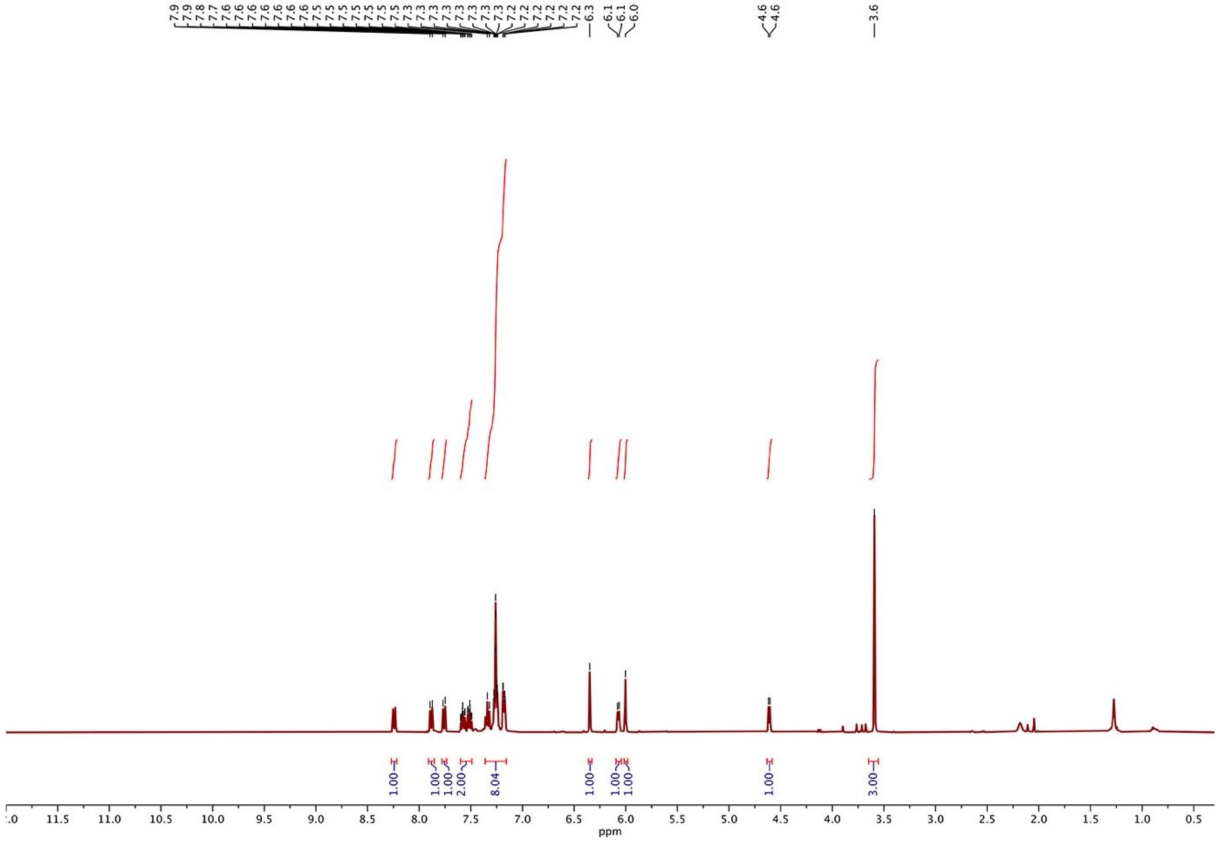

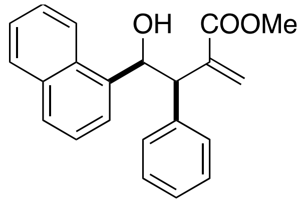

3ca, 68% *dr* > 95:5

^{13}**C{^{1}H} NMR** (100 MHz, $CDCl_3$)

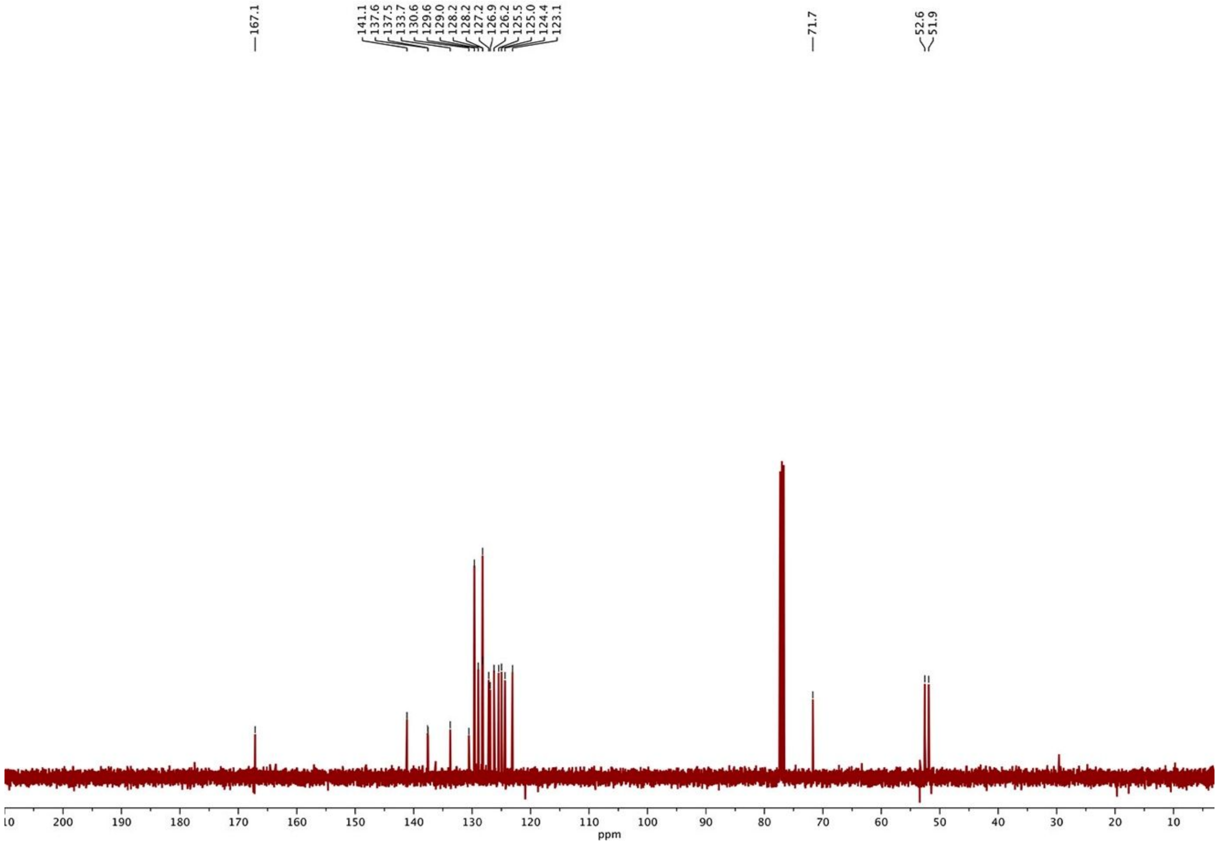

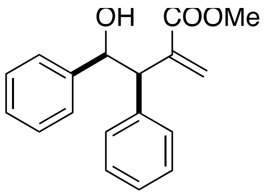

3da, 54%
dr > 95:5

^{1}H NMR (400 MHz, $CDCl_3$)

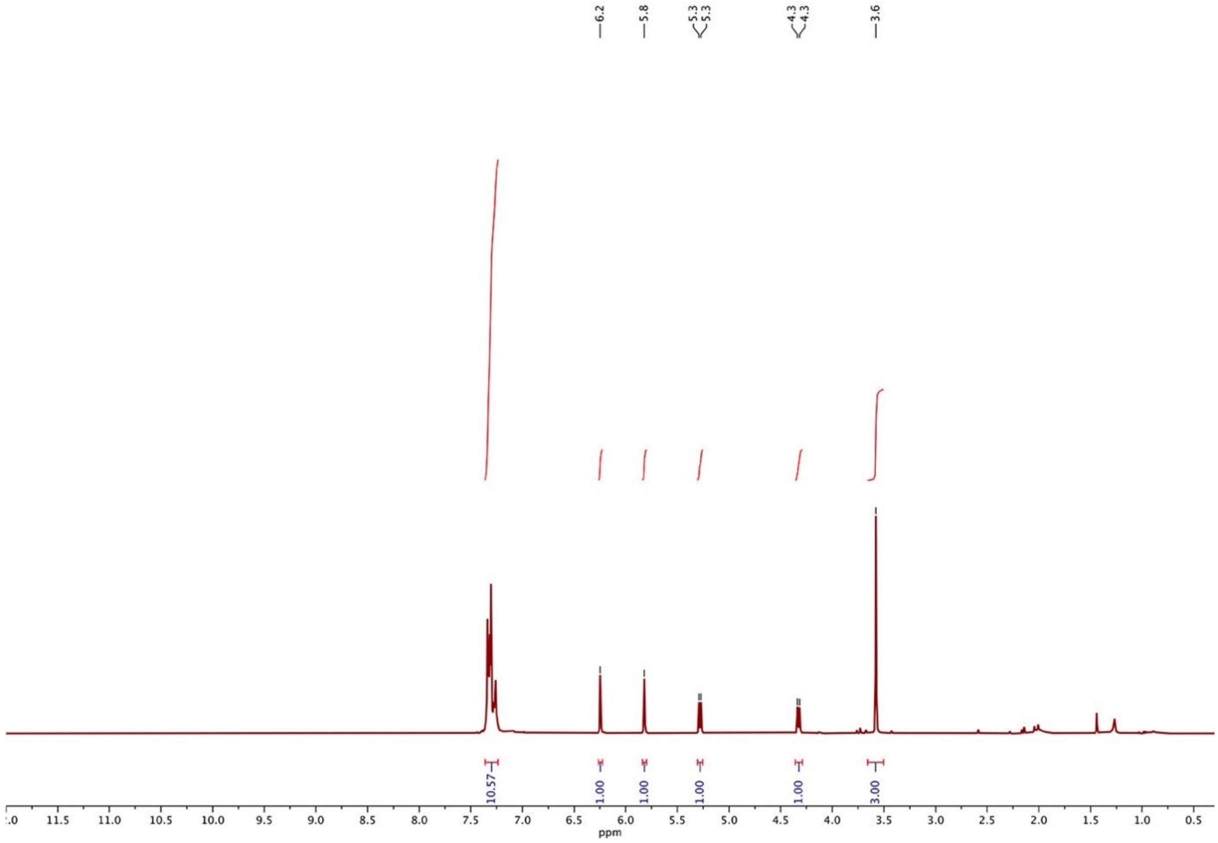

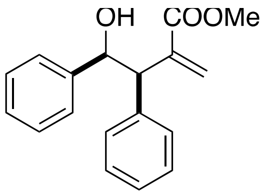

3da, 54%
dr > 95:5

^{13}C{^{1}H} NMR (100 MHz, $CDCl_3$)

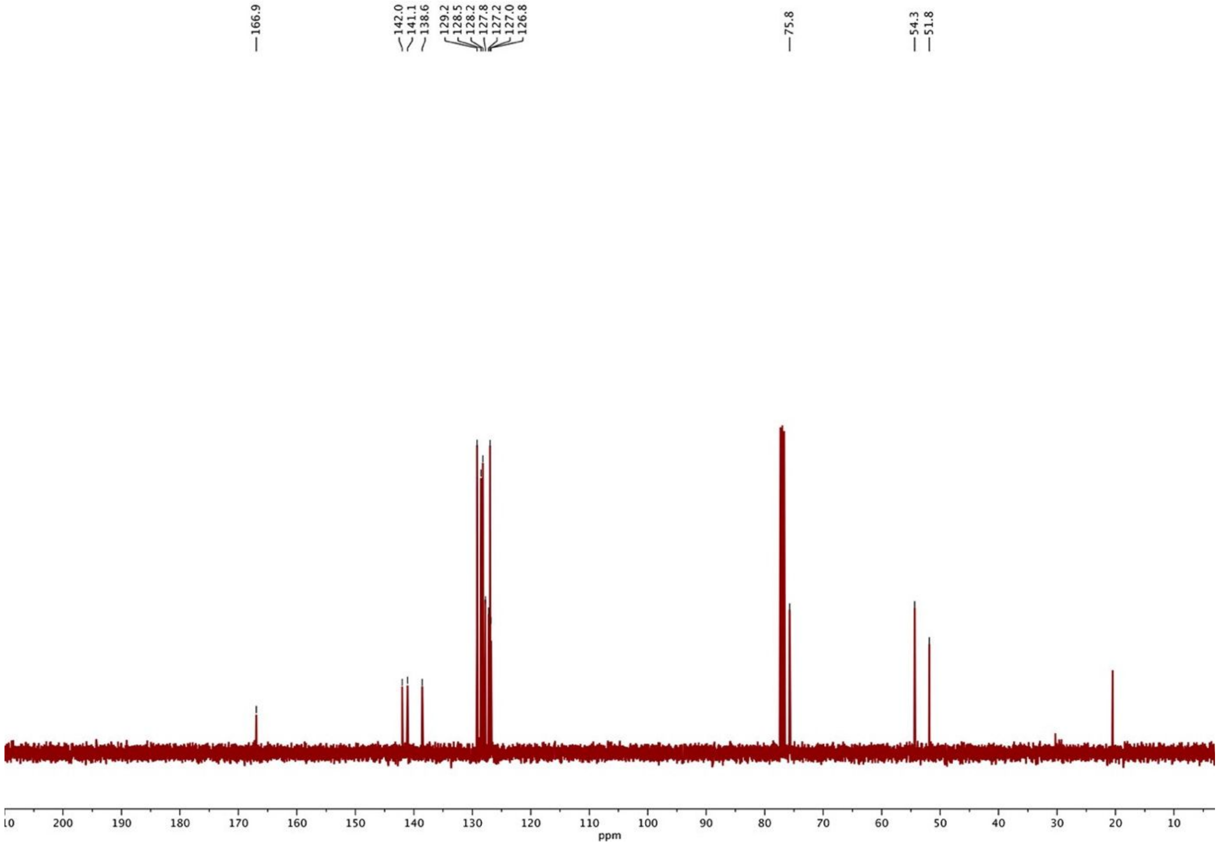

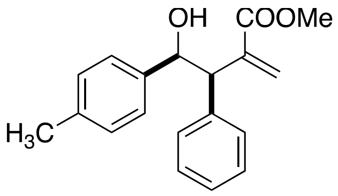

3ea, 68% *dr* > 95:5

^{1}H NMR (400 MHz, $CDCl_3$)

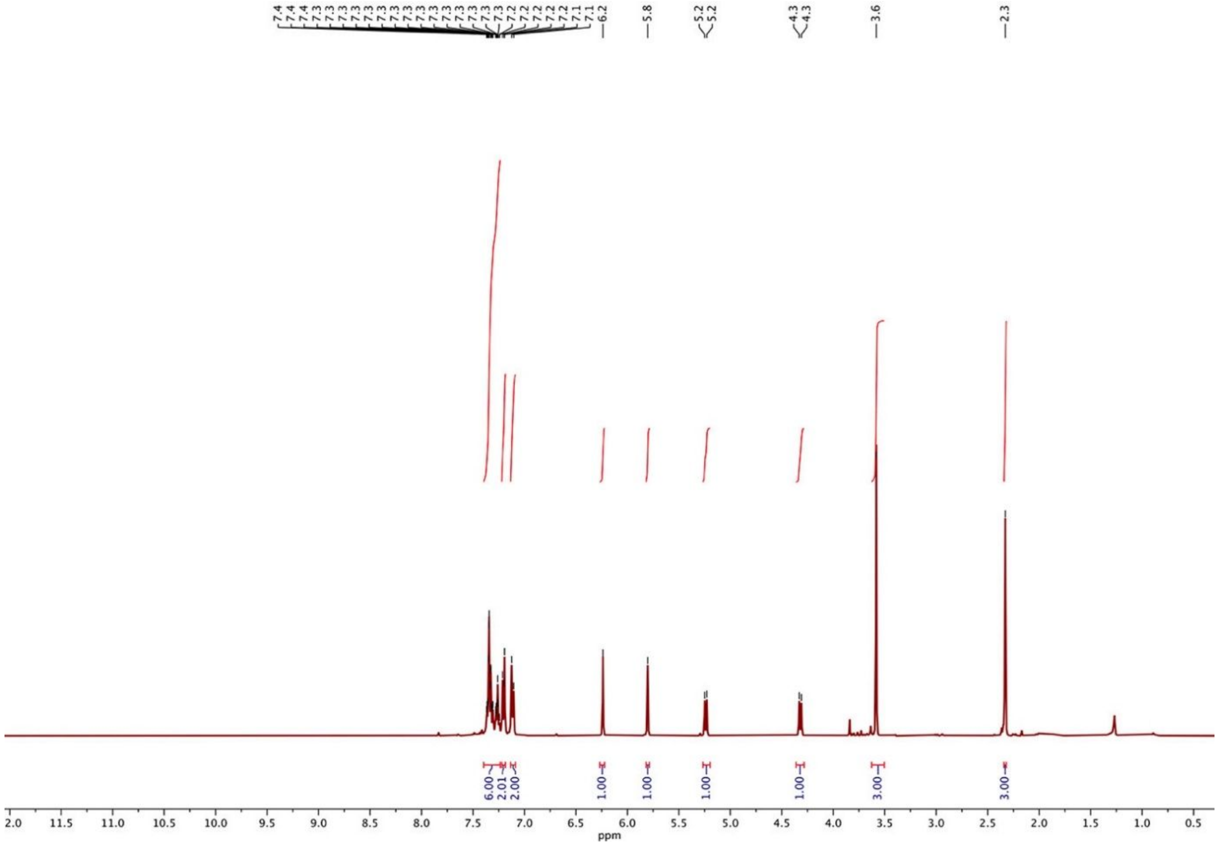

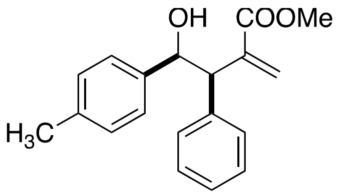

3ea, 68% *dr* > 95:5

$^{13}C\{^{1}H\}$ NMR (100 MHz, $CDCl_3$)

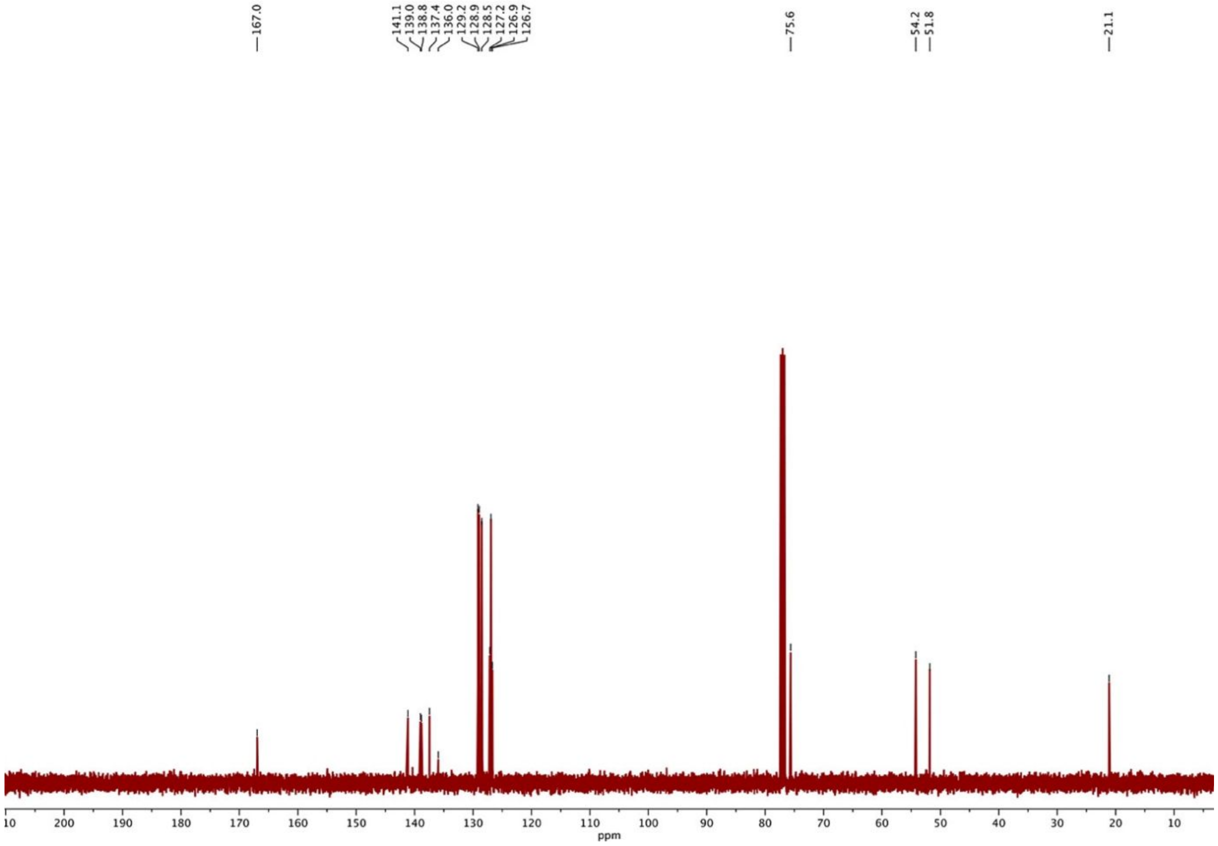

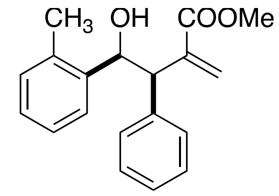

3fa, 67% *dr* > 95:5

^{1}H NMR (400 MHz, $CDCl_3$)

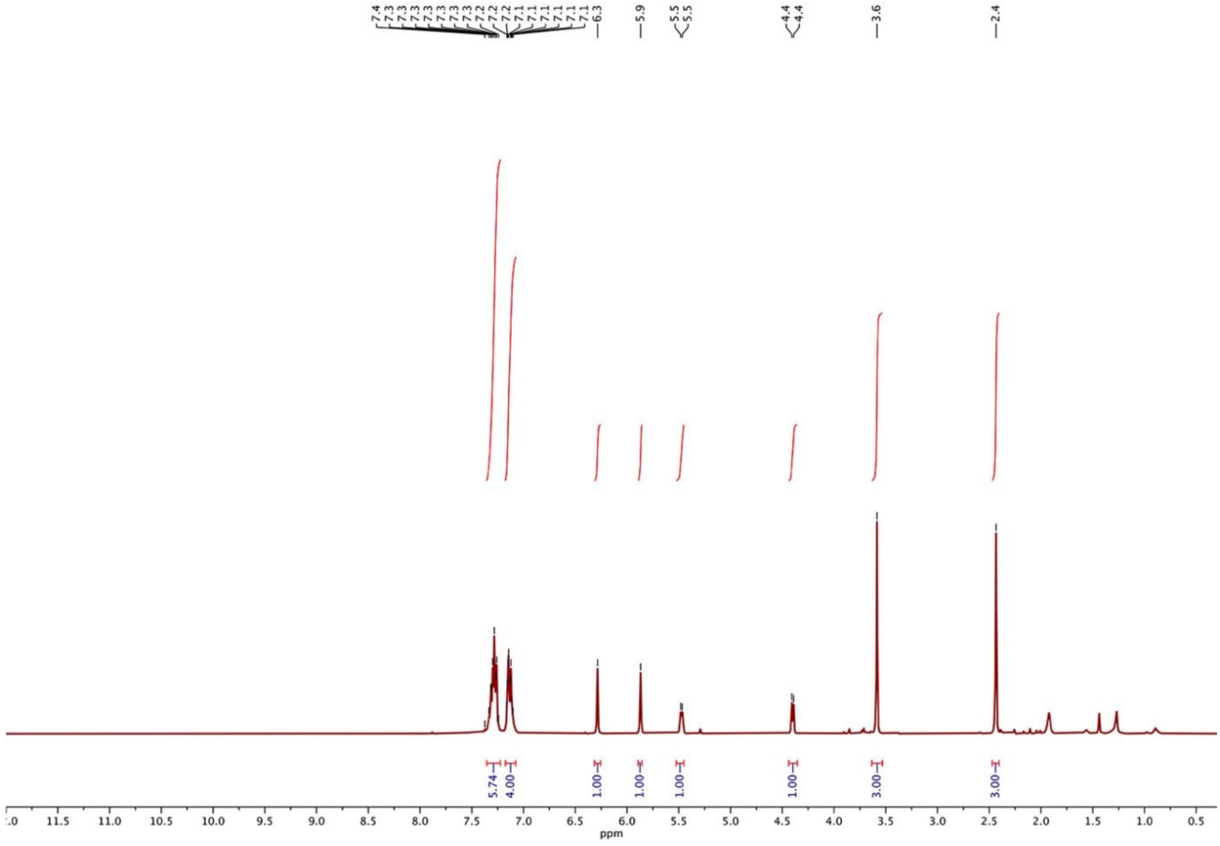

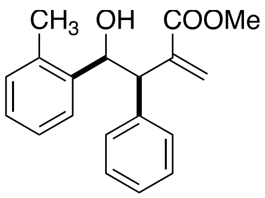

3fa, 67% *dr* > 95:5

^{13}C{^{1}H} NMR (100 MHz, $CDCl_3$)

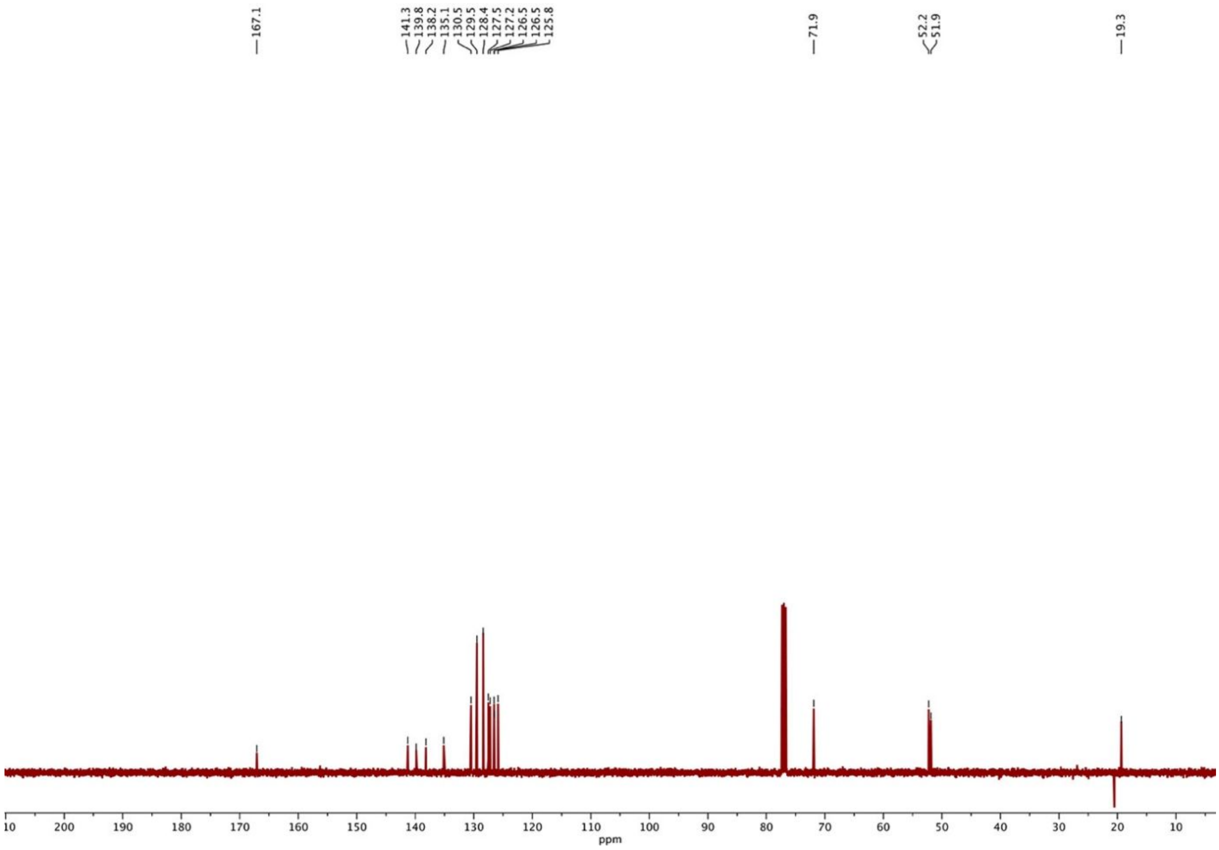

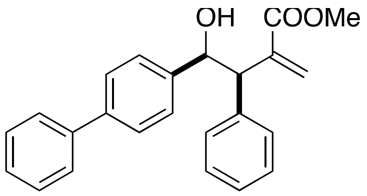

3ga, 81% *dr* > 95:5

^{1}H NMR (400 MHz, $CDCl_3$)

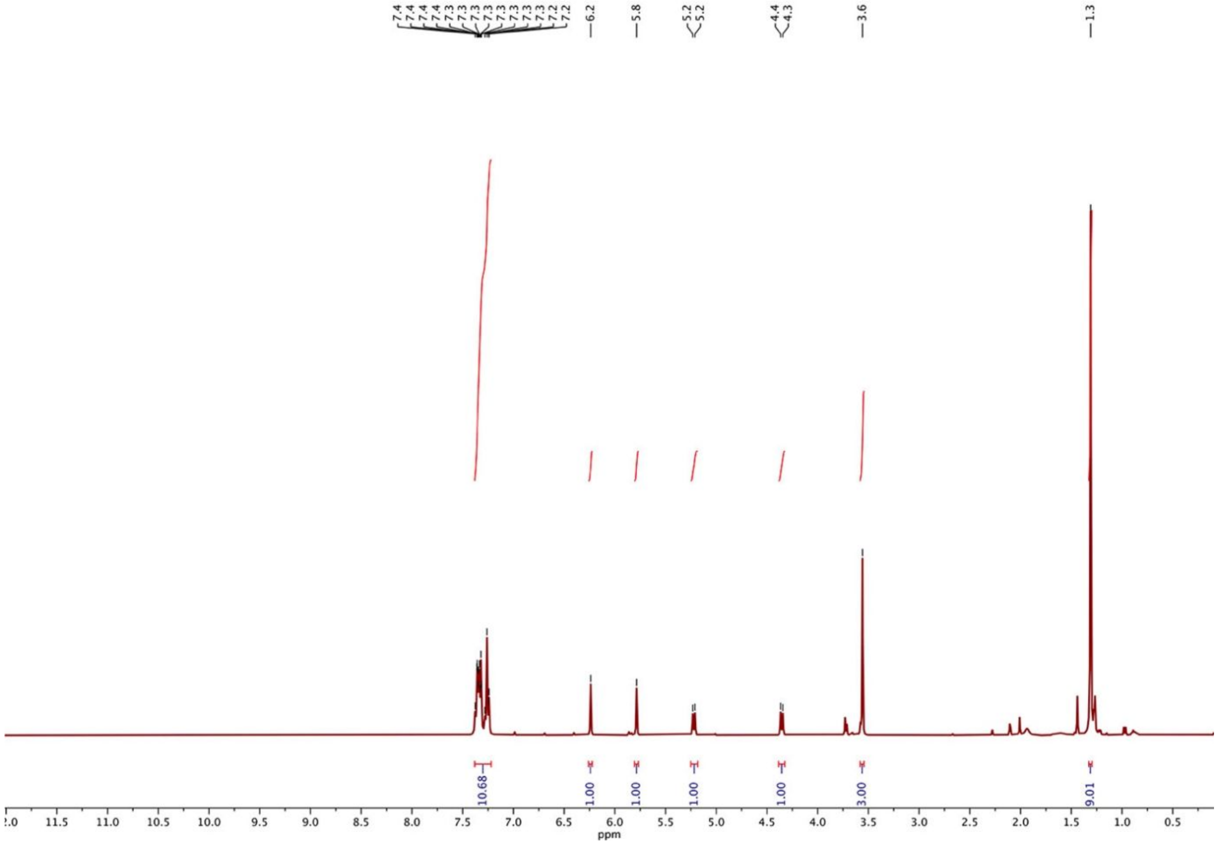

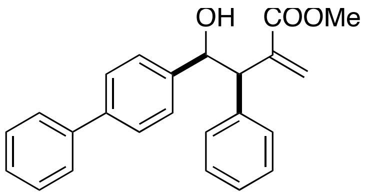

3ga, 81% *dr* > 95:5

^{13}C{^{1}H} NMR (100 MHz, $CDCl_3$)

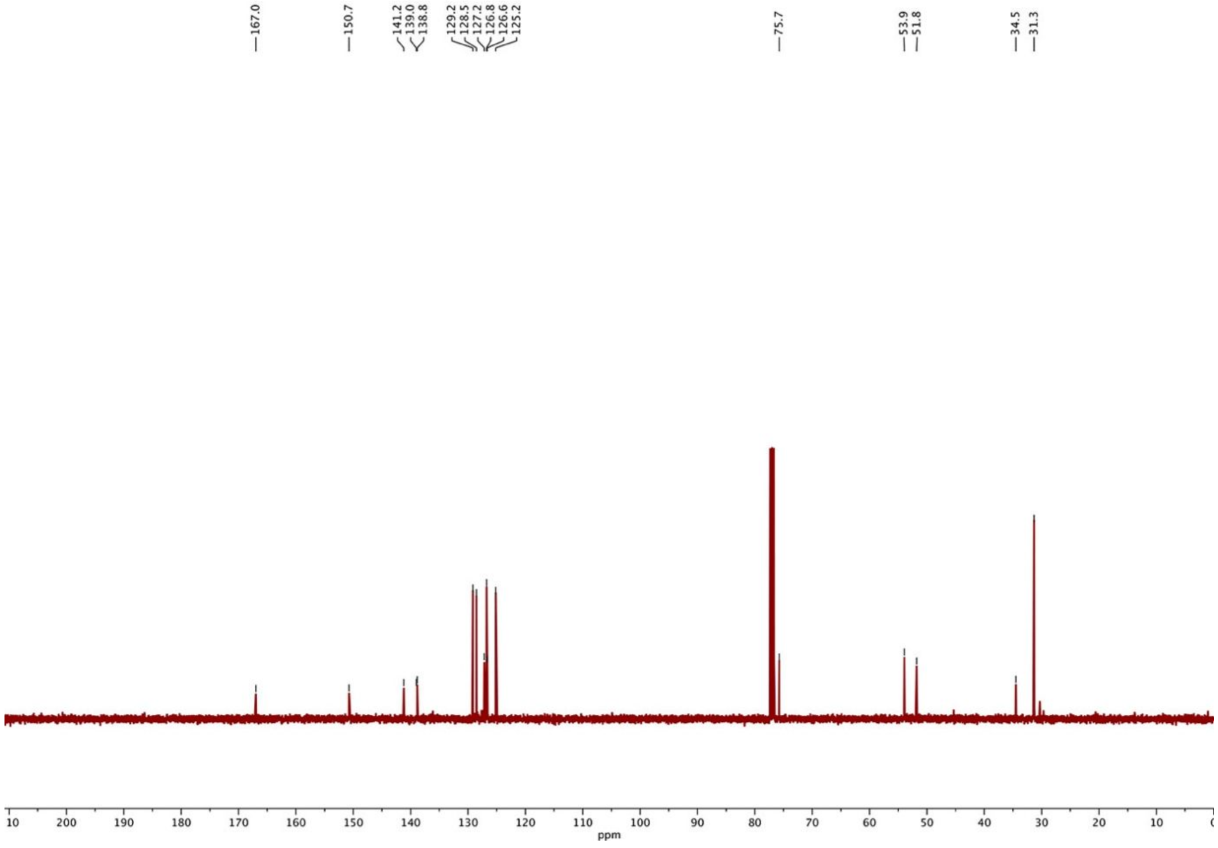

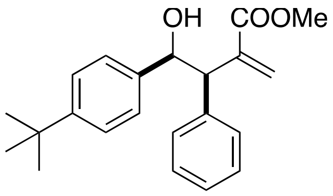

3ha, 50% *dr* > 95:5

^{1}H NMR (400 MHz, $CDCl_3$)

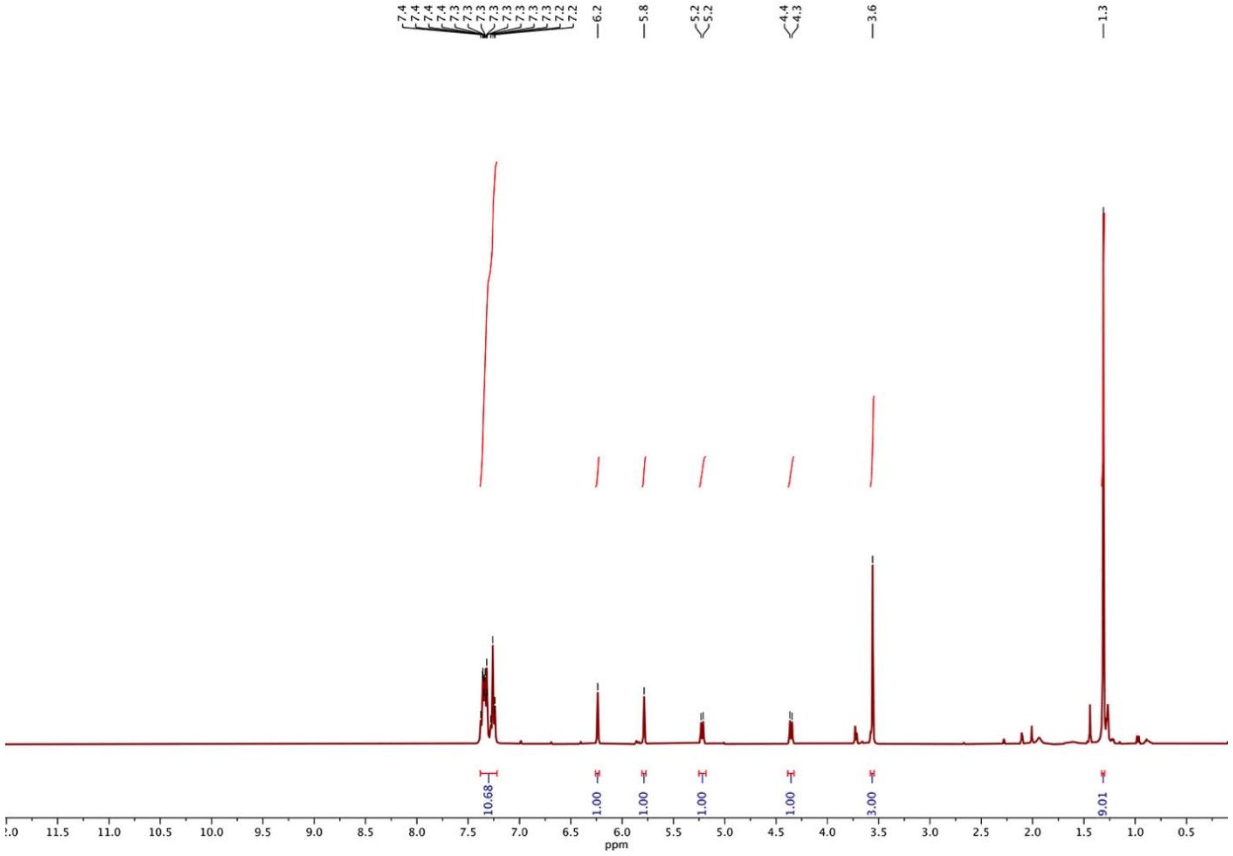

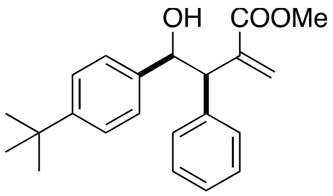

3ha, 50% *dr* > 95:5

^{13}C{^{1}H} NMR (100 MHz, $CDCl_3$)

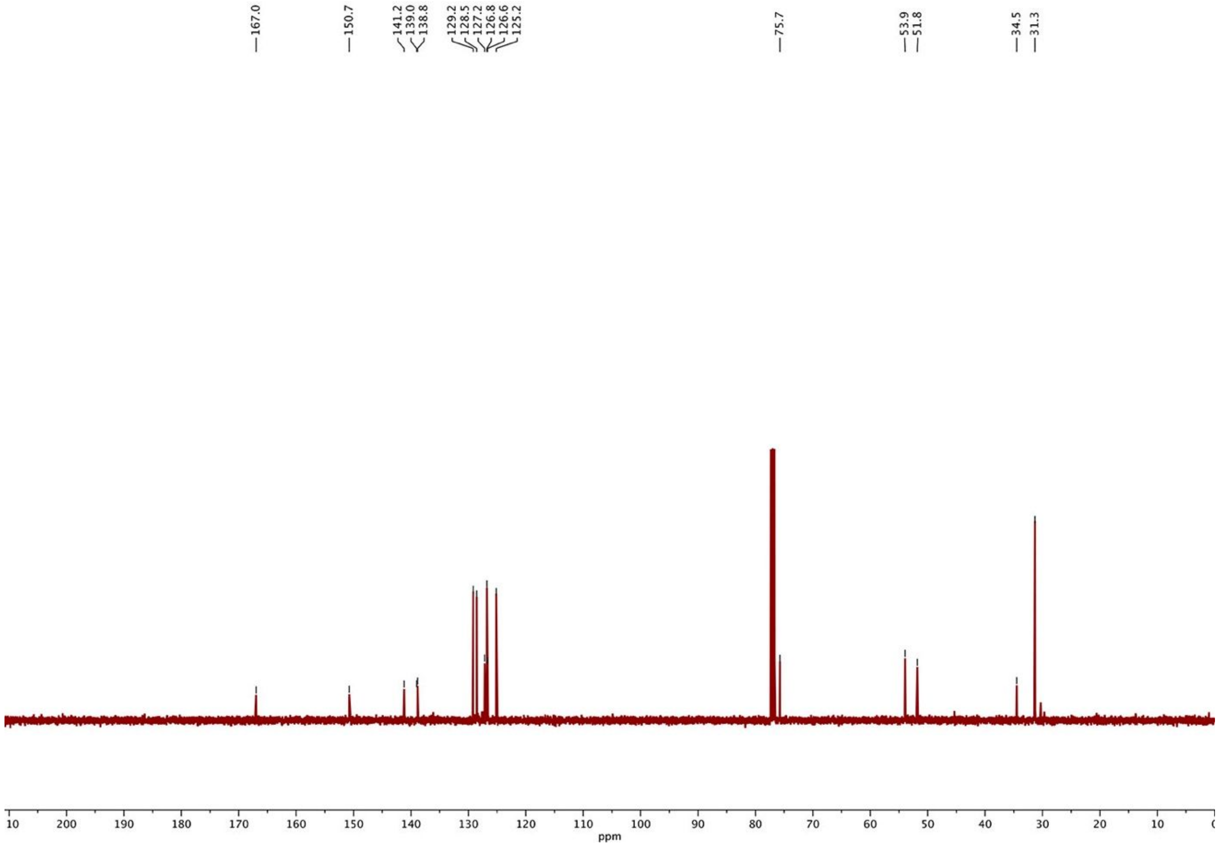

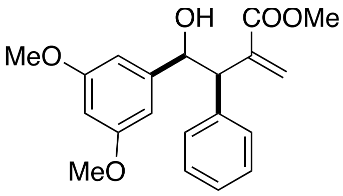

3ia*, 30% *dr* 90:10

[1]**H NMR** (400 MHz, $CDCl_3$)

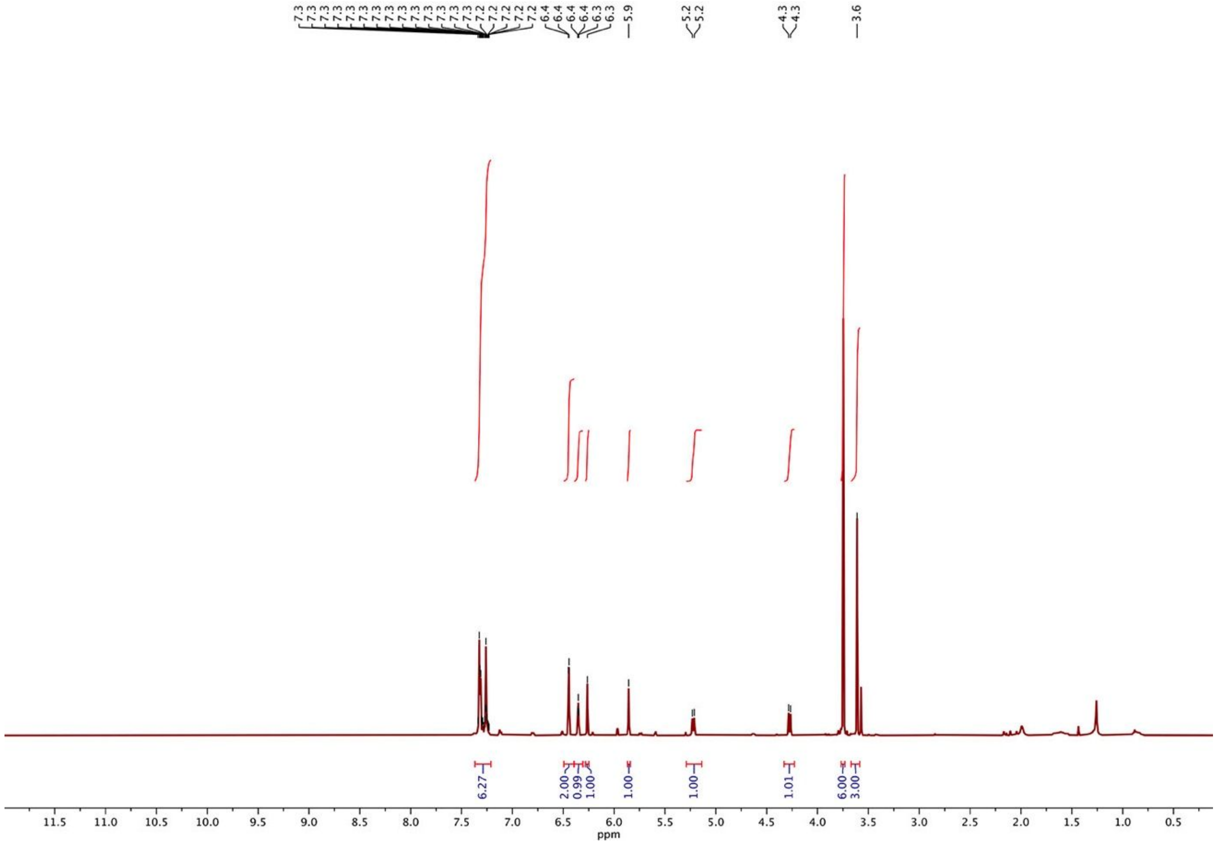

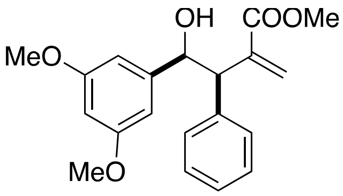

3ia*, 30% *dr* 90:10

^{13}C{^{1}H} NMR (100 MHz, $CDCl_3$)

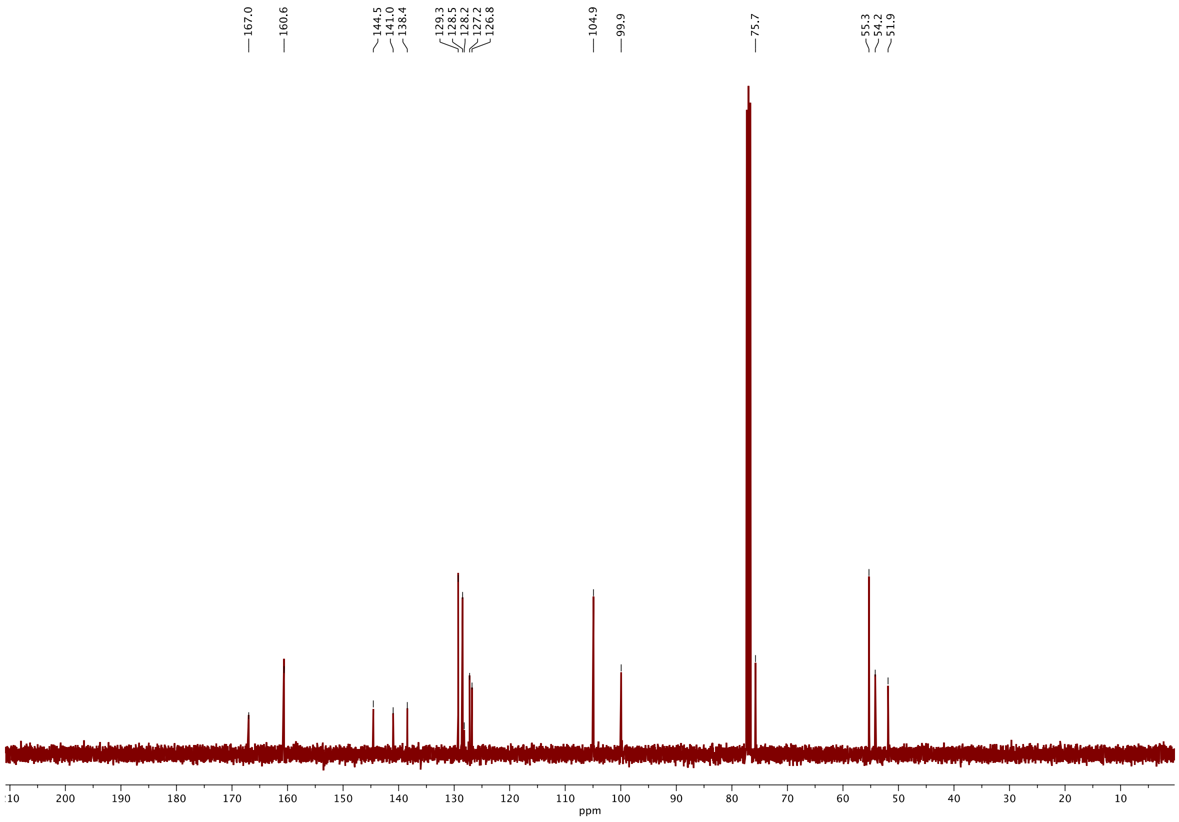

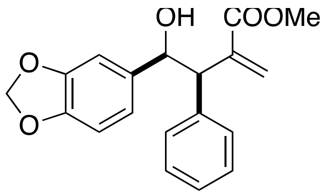

3ja*, 46%[b] *dr* > 95:5

^{1}H NMR (400 MHz, $CDCl_3$)

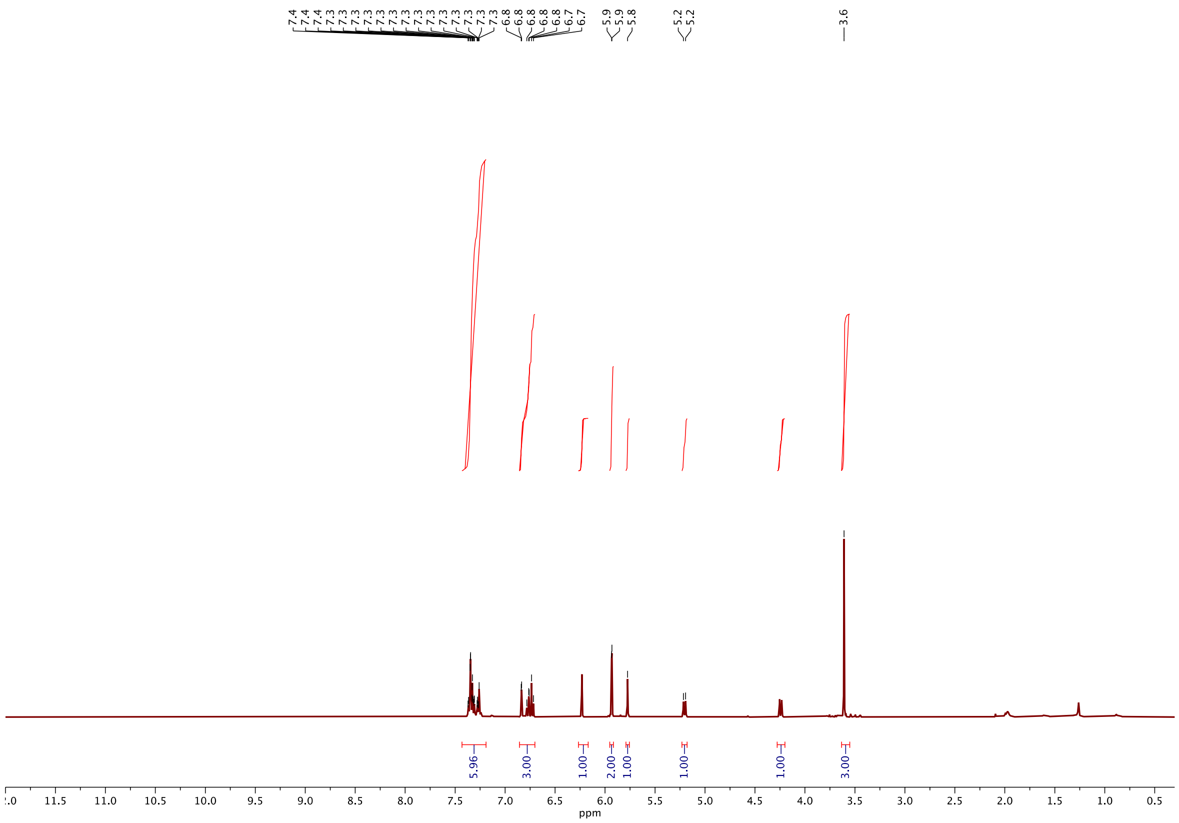

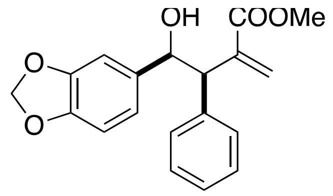

3ja*, 46%[b] *dr* > 95:5

$^{13}C\{^1H\}$ **NMR** (100 MHz, $CDCl_3$)

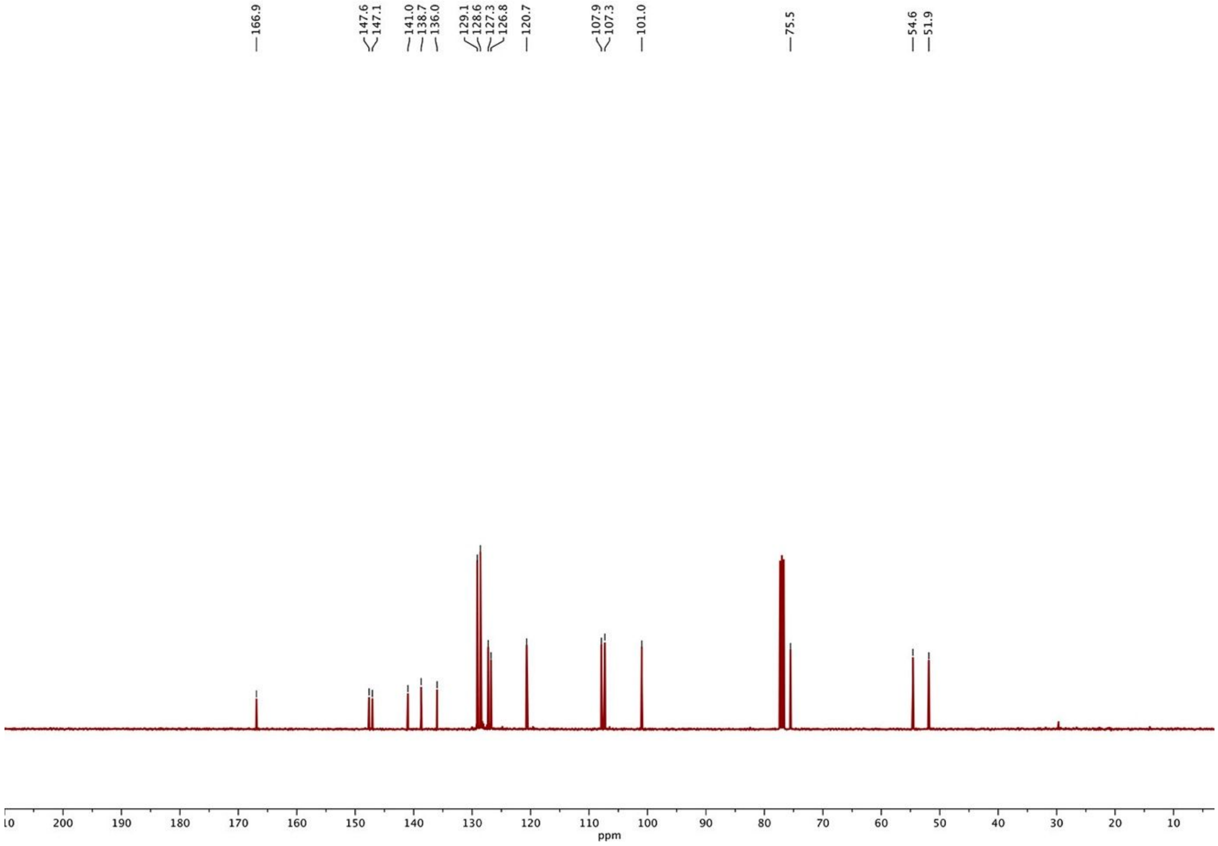

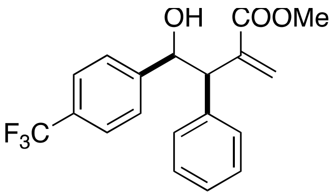

3ka, 71% *dr* 85:15

^{1}H NMR (400 MHz, $CDCl_3$)

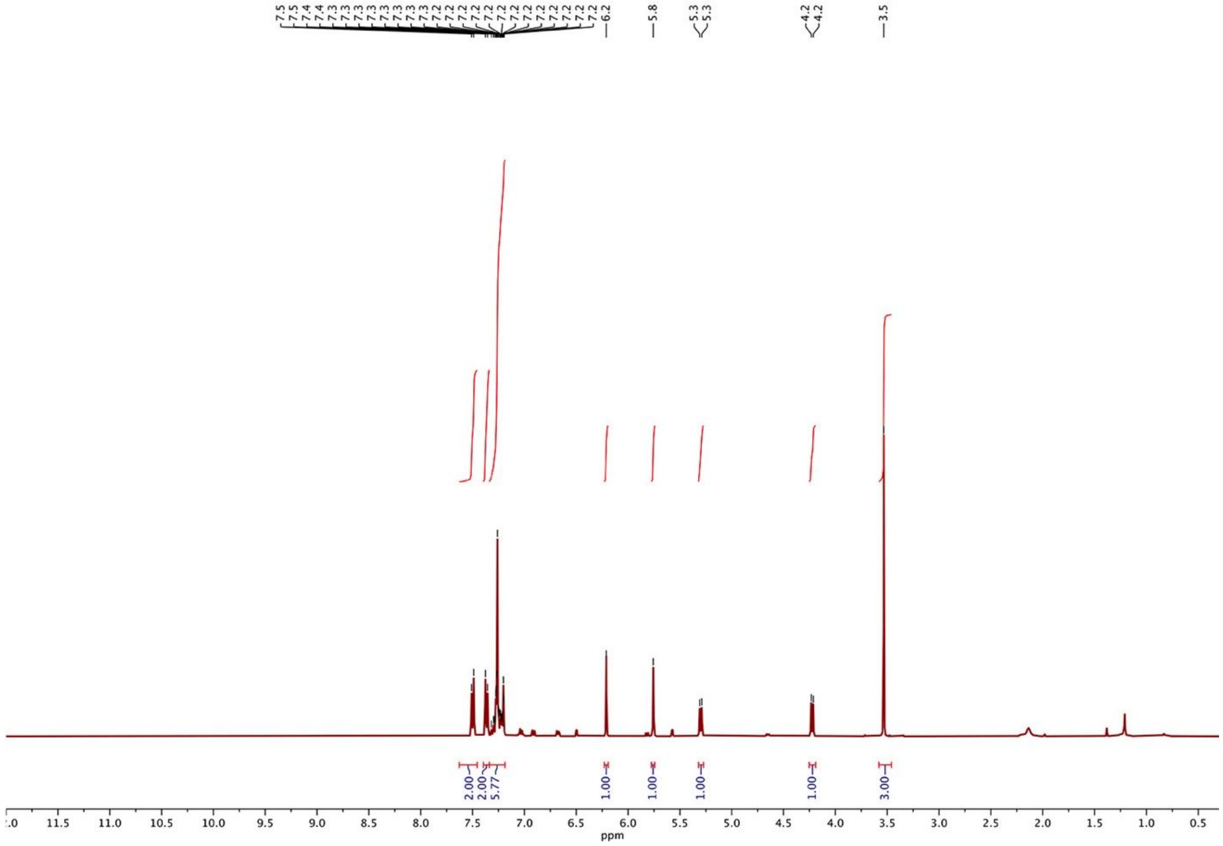

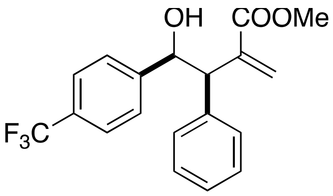

3ka, 71% *dr* 85:15

^{13}C{^{1}H} NMR (100 MHz, $CDCl_3$)

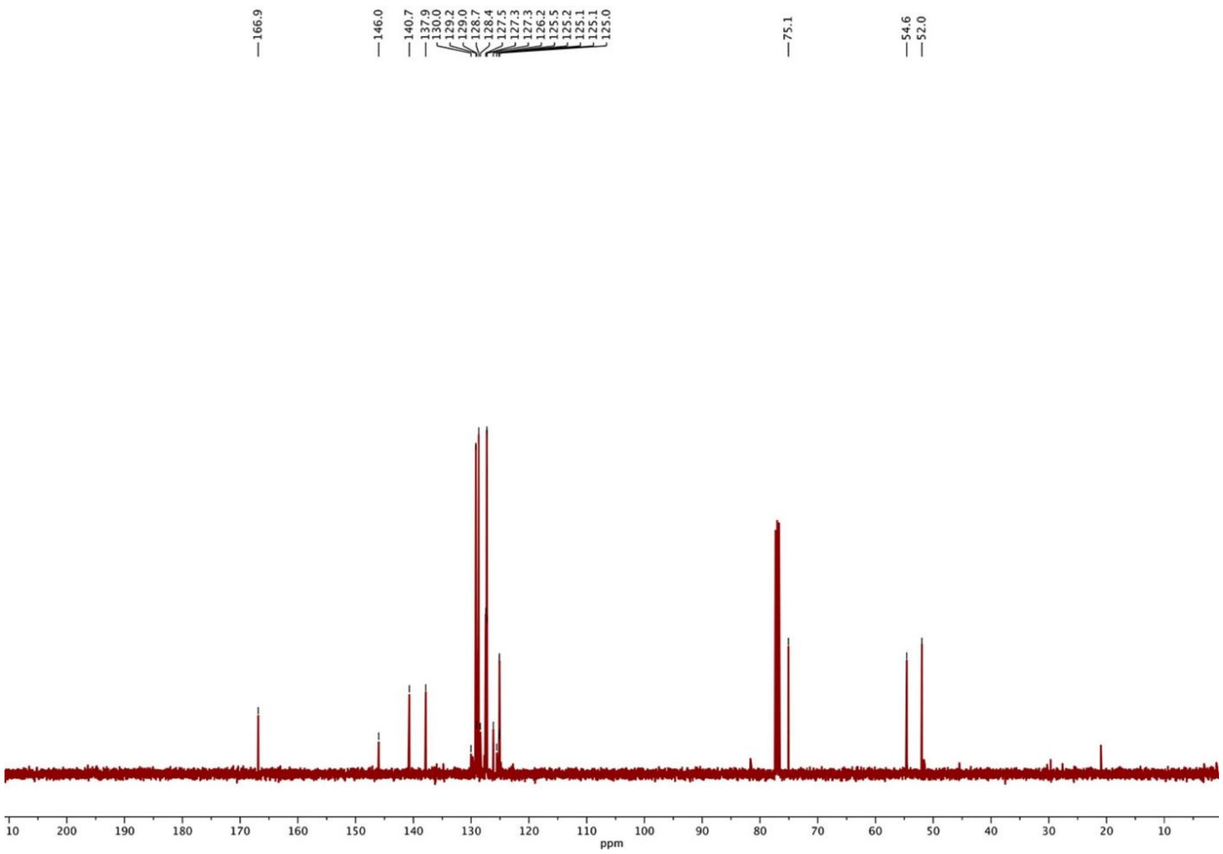

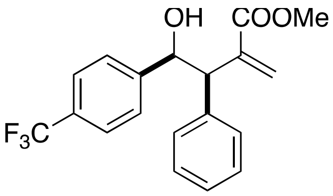

3ka, 71% *dr* 85:15

^{19}F NMR (377 MHz, $CDCl_3$)

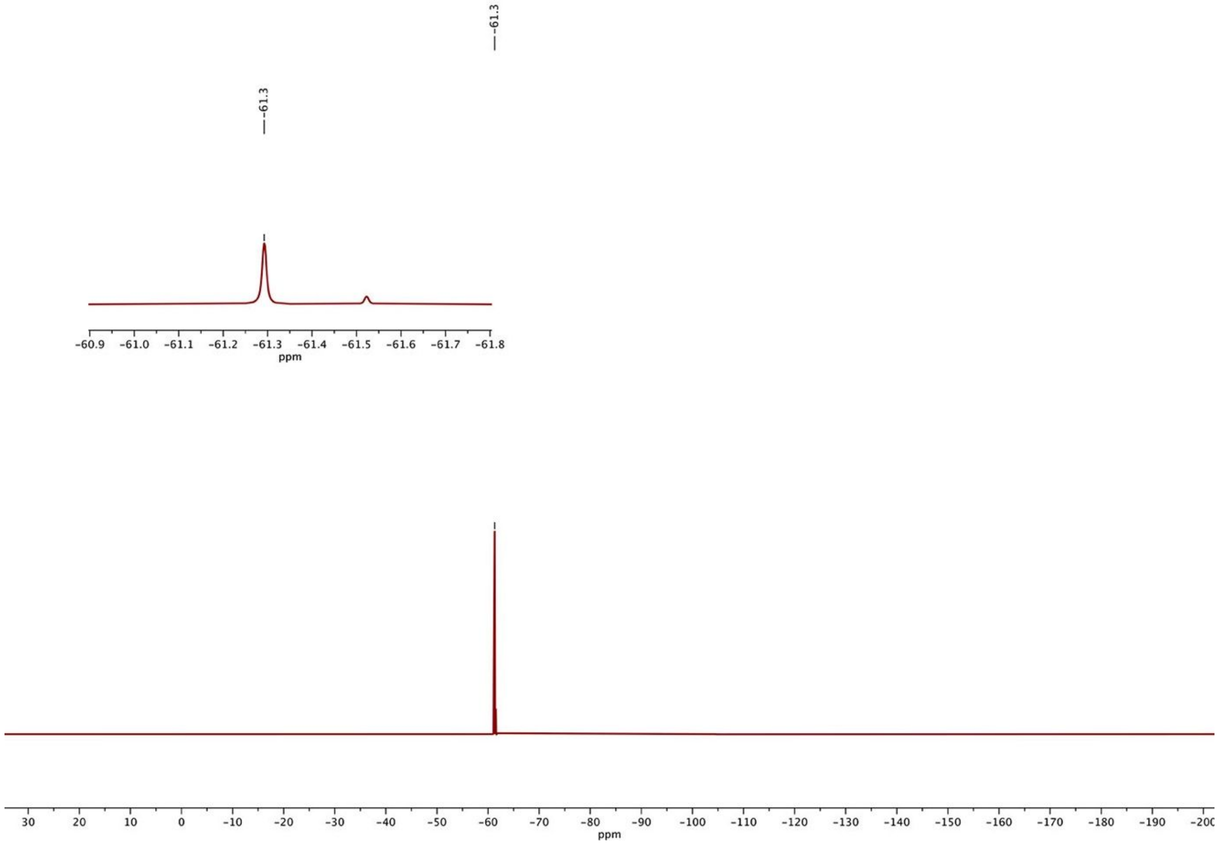

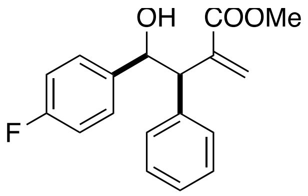

3la, 78% *dr* > 95:5

^{1}H NMR (400 MHz, $CDCl_3$)

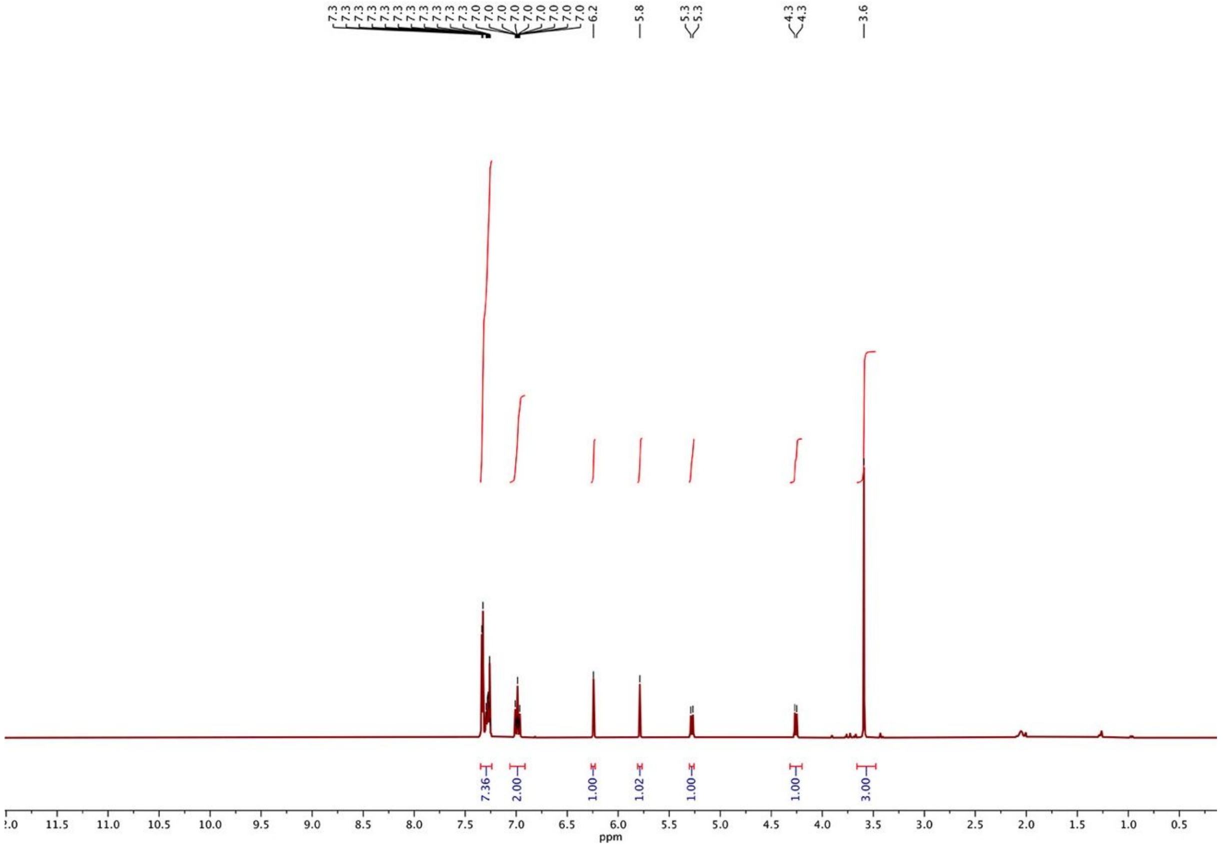

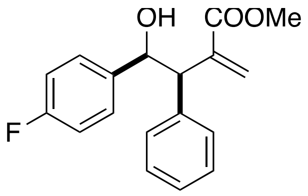

3la, 78% *dr* > 95:5

$^{13}C\{^1H\}$ NMR (100 MHz, $CDCl_3$)

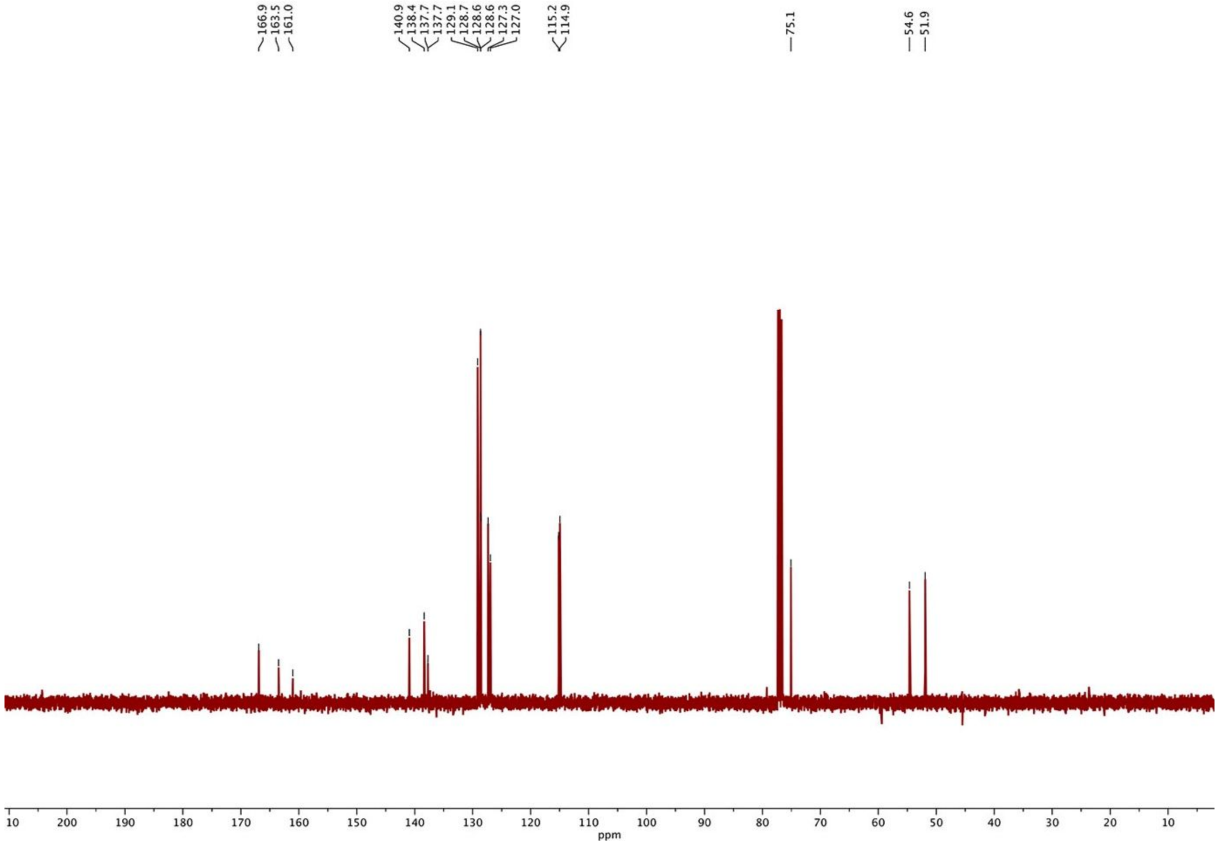

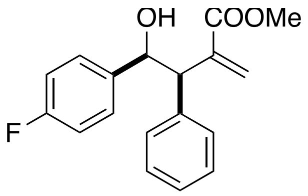

3la, 78% *dr* > 95:5

^{19}F NMR (377 MHz, $CDCl_3$)

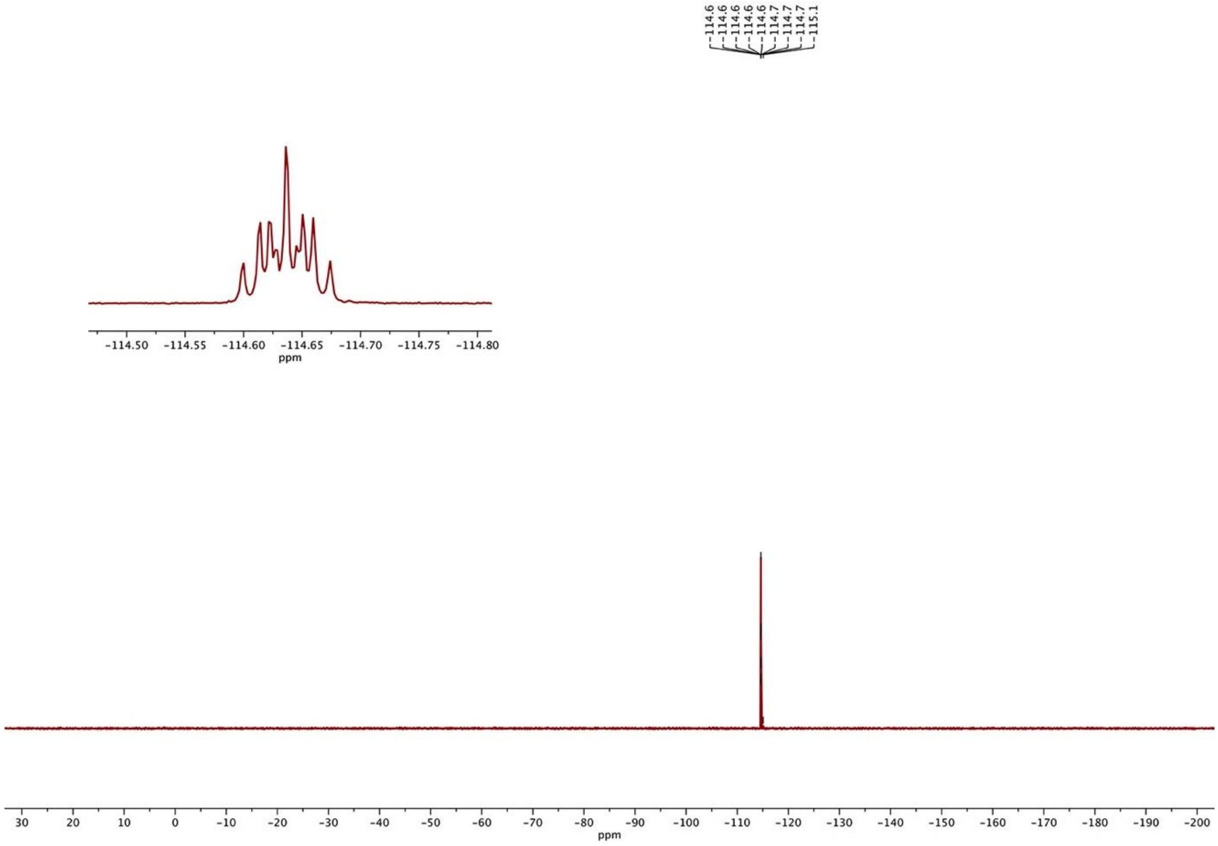

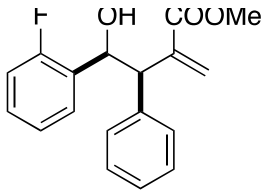

3ma, 30% *dr* > 95:5

^{1}H NMR (400 MHz, $CDCl_3$)

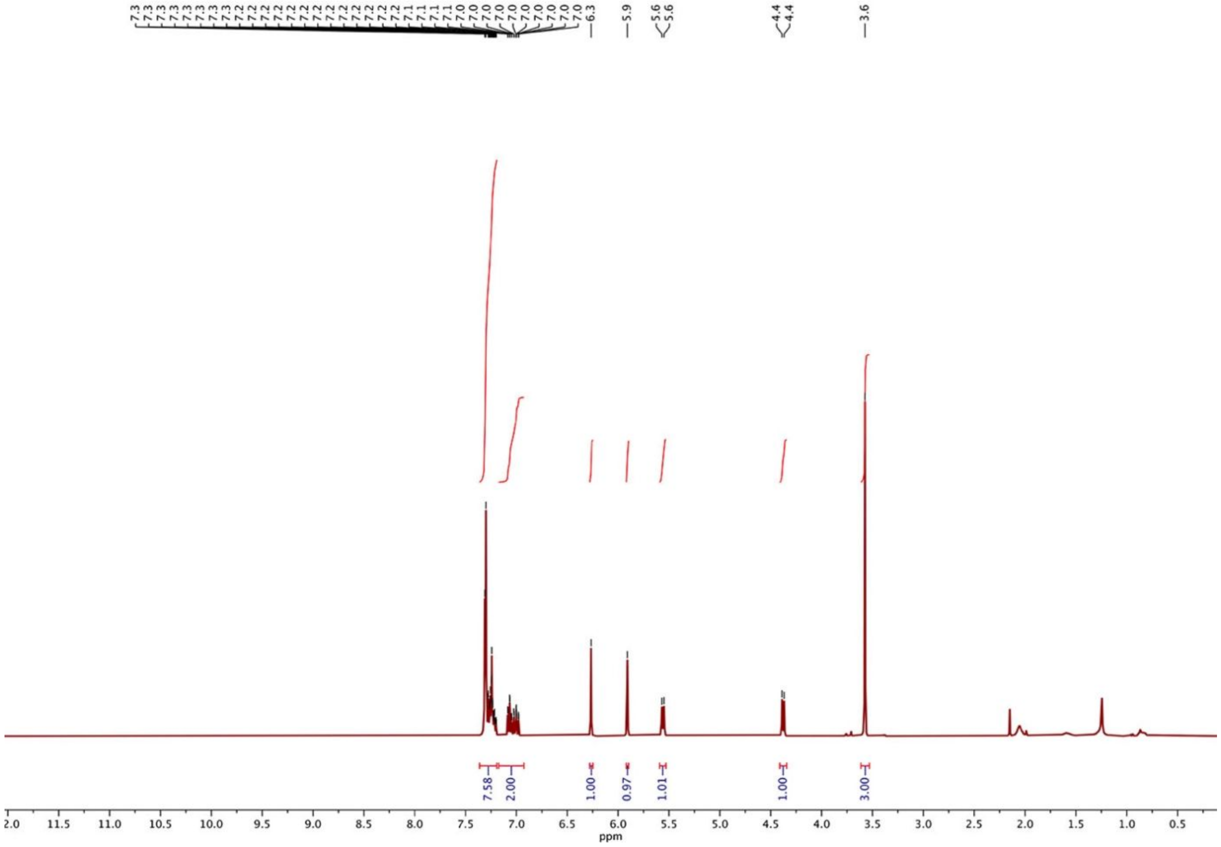

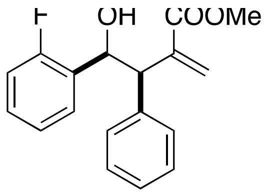

3ma, 30% *dr* > 95:5

^{13}C{^{1}H} NMR (100 MHz, $CDCl_3$)

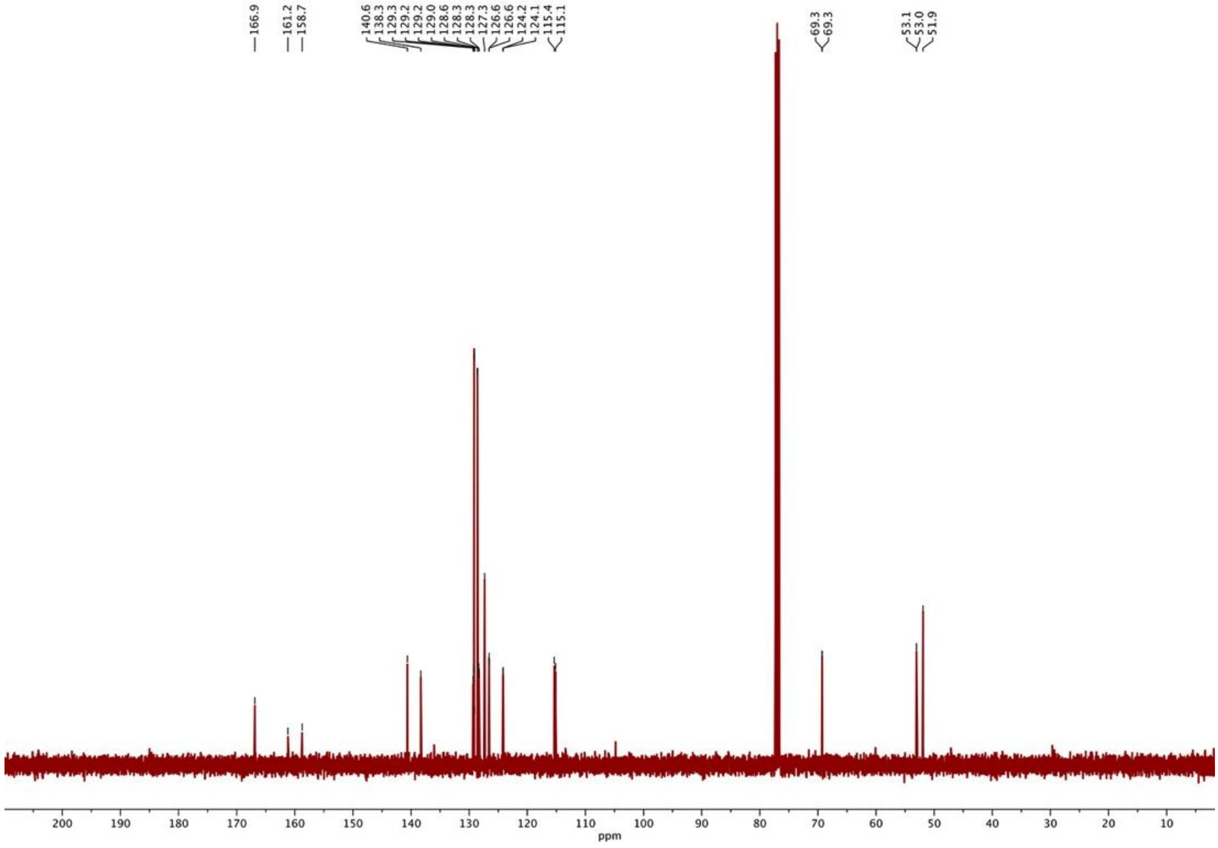

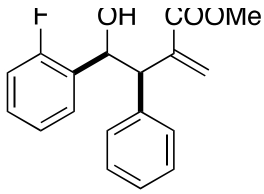

3ma, 30% *dr* > 95:5

^{19}F NMR (377 MHz, $CDCl_3$)

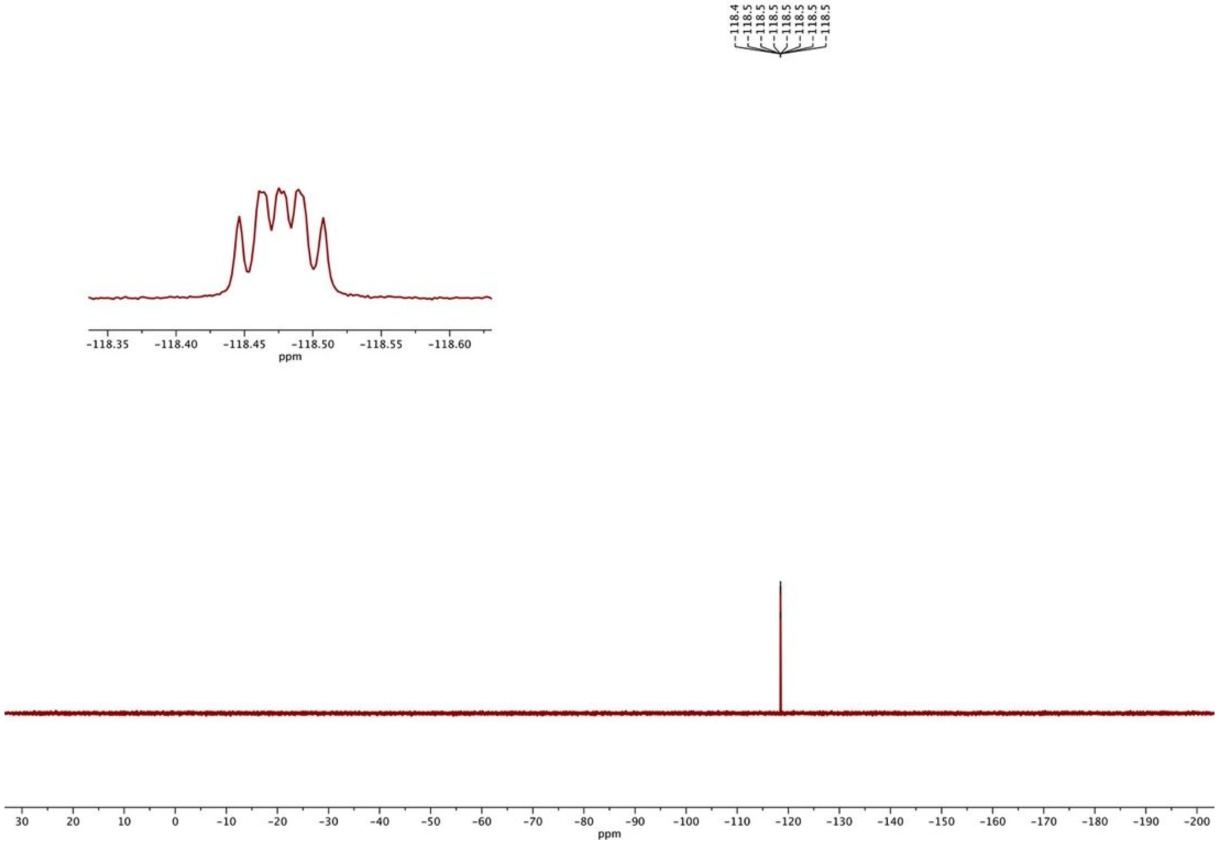

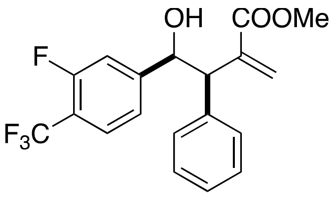

3na, 57% dr 92:8

^{1}H NMR (400 MHz, $CDCl_3$)

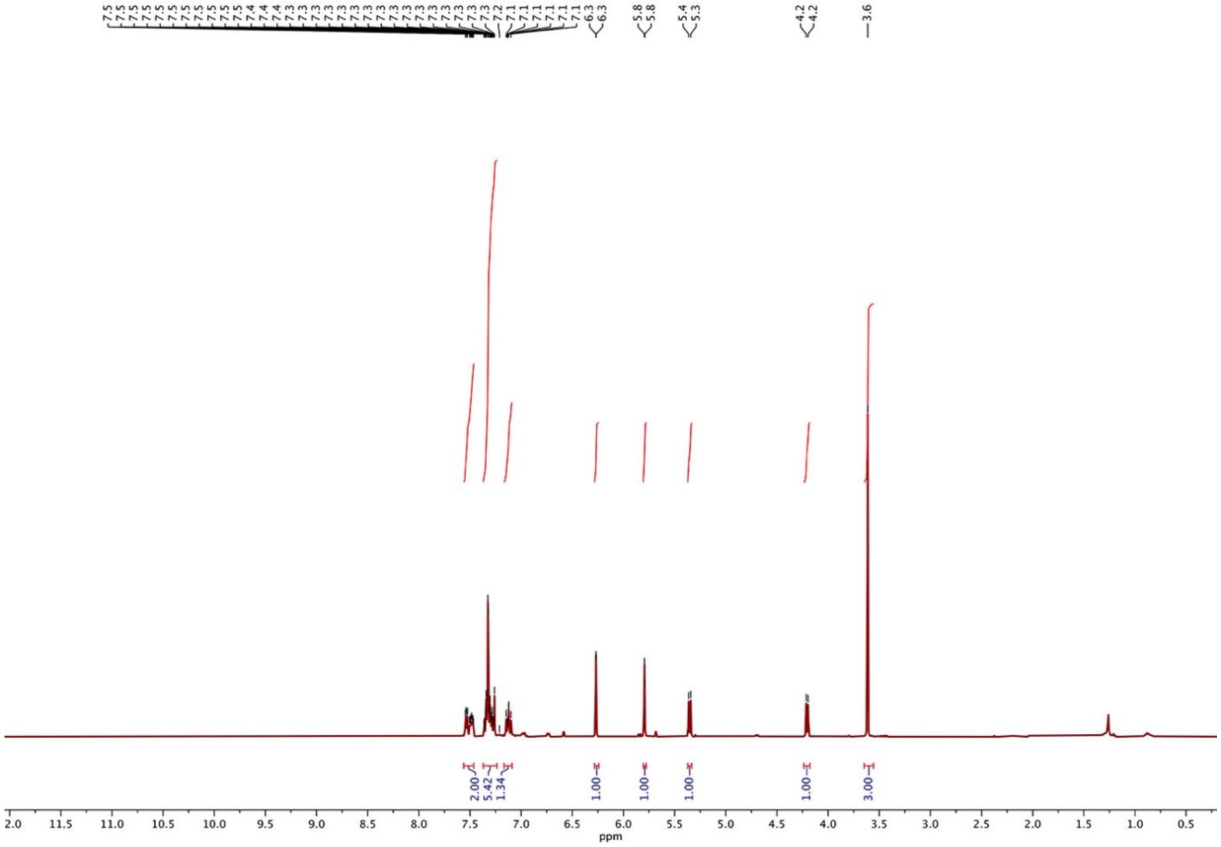

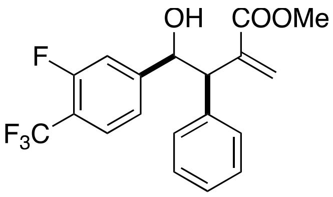

3na, 57% dr 92:8

^{13}C{^{1}H} NMR (100 MHz, $CDCl_3$)

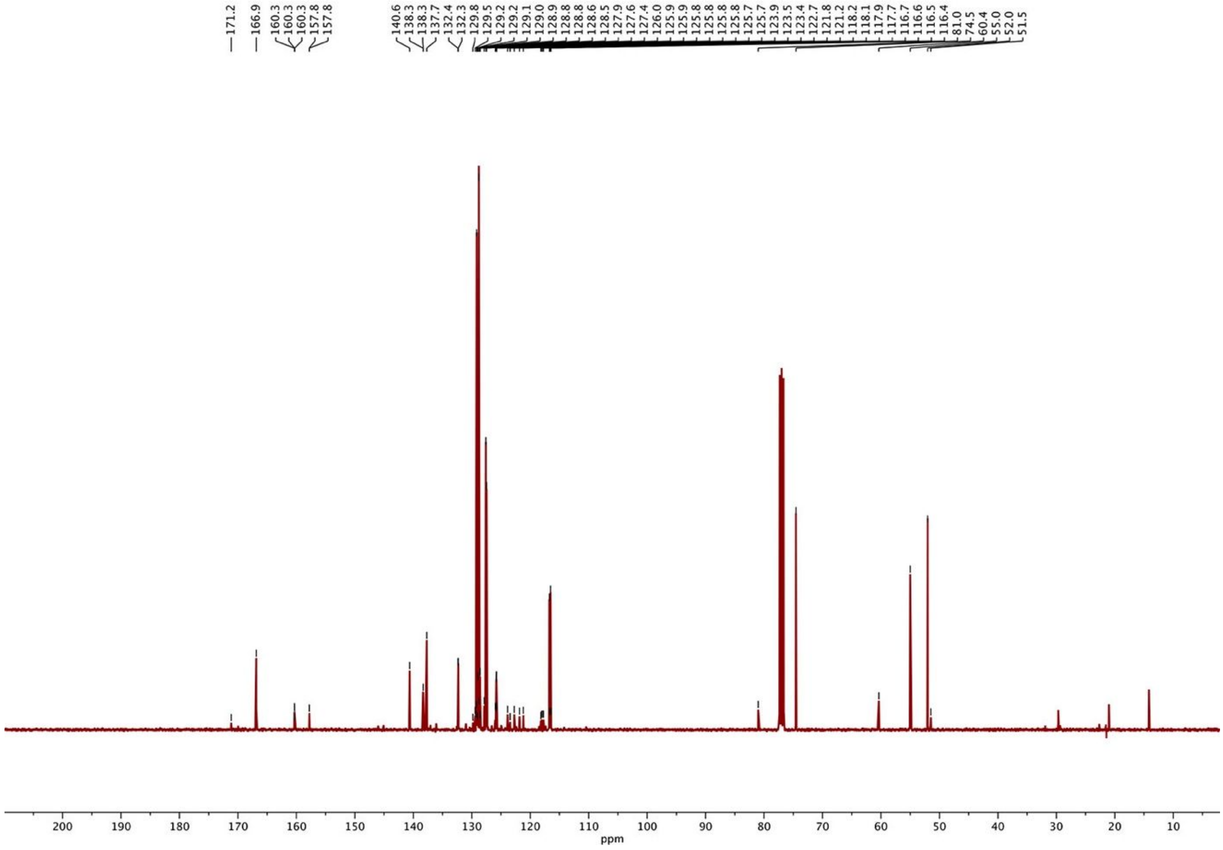

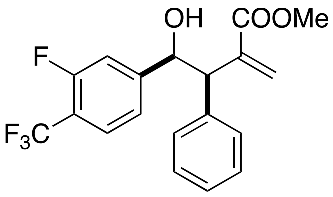

3na, 57% dr 92:8

^{19}F NMR (377 MHz, $CDCl_3$)

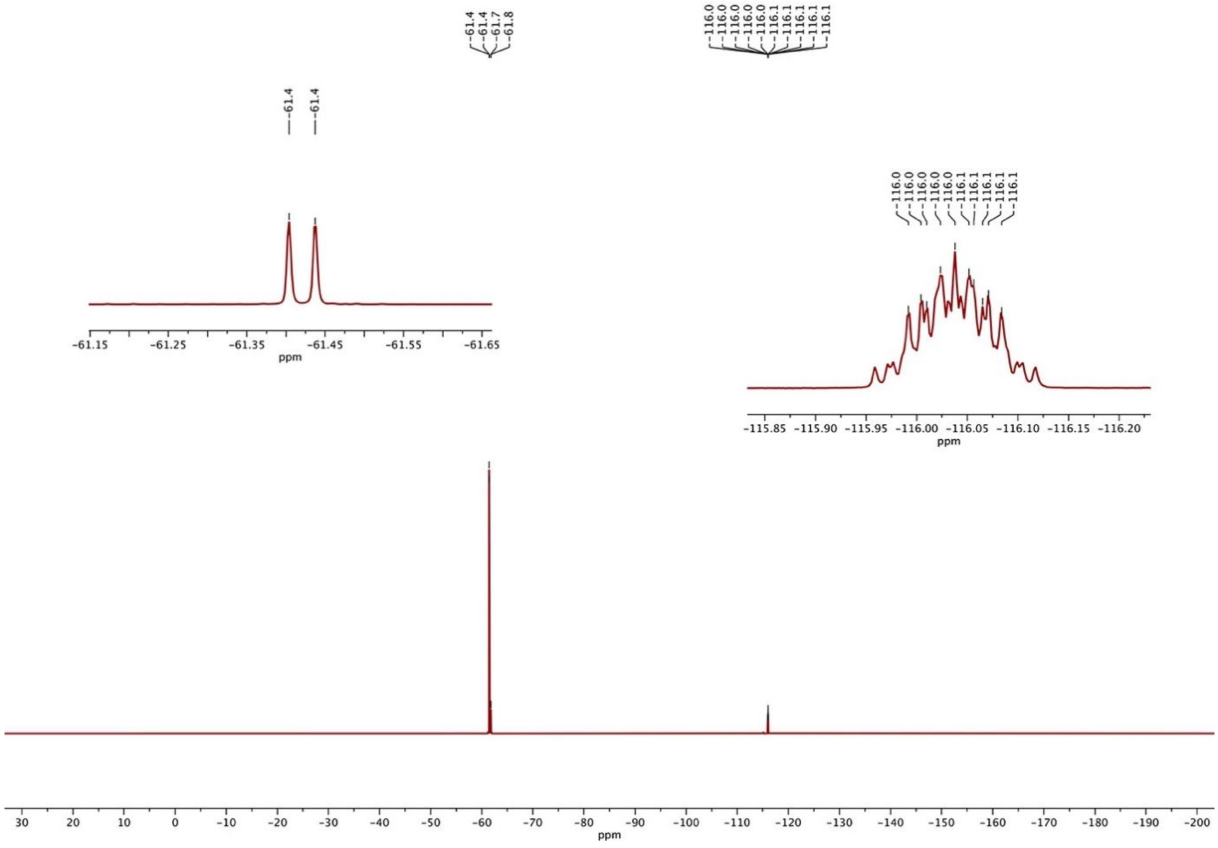

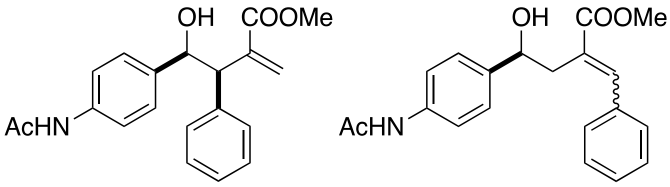

3oa+ 3oa', 67% *dr* > 95:5 *rr* = 65:35; E/Z 56:44

^{1}H NMR (400 MHz, $CDCl_3$)

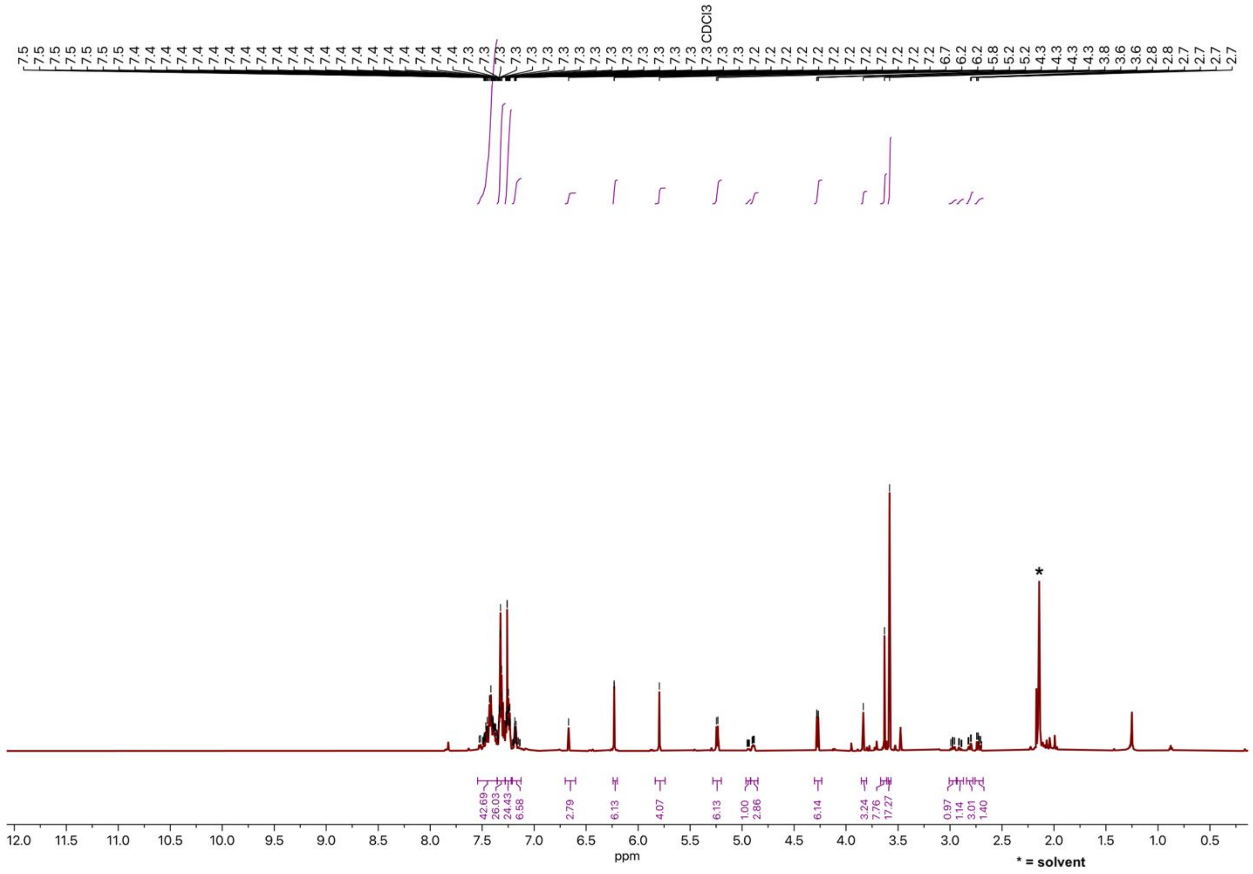

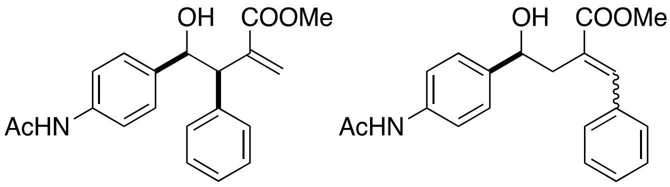

3oa+ 3oa', 67% *dr* > 95:5 *rr* = 65:35; E/Z 56:44

^{13}C{^{1}H} NMR (100 MHz, $CDCl_3$)

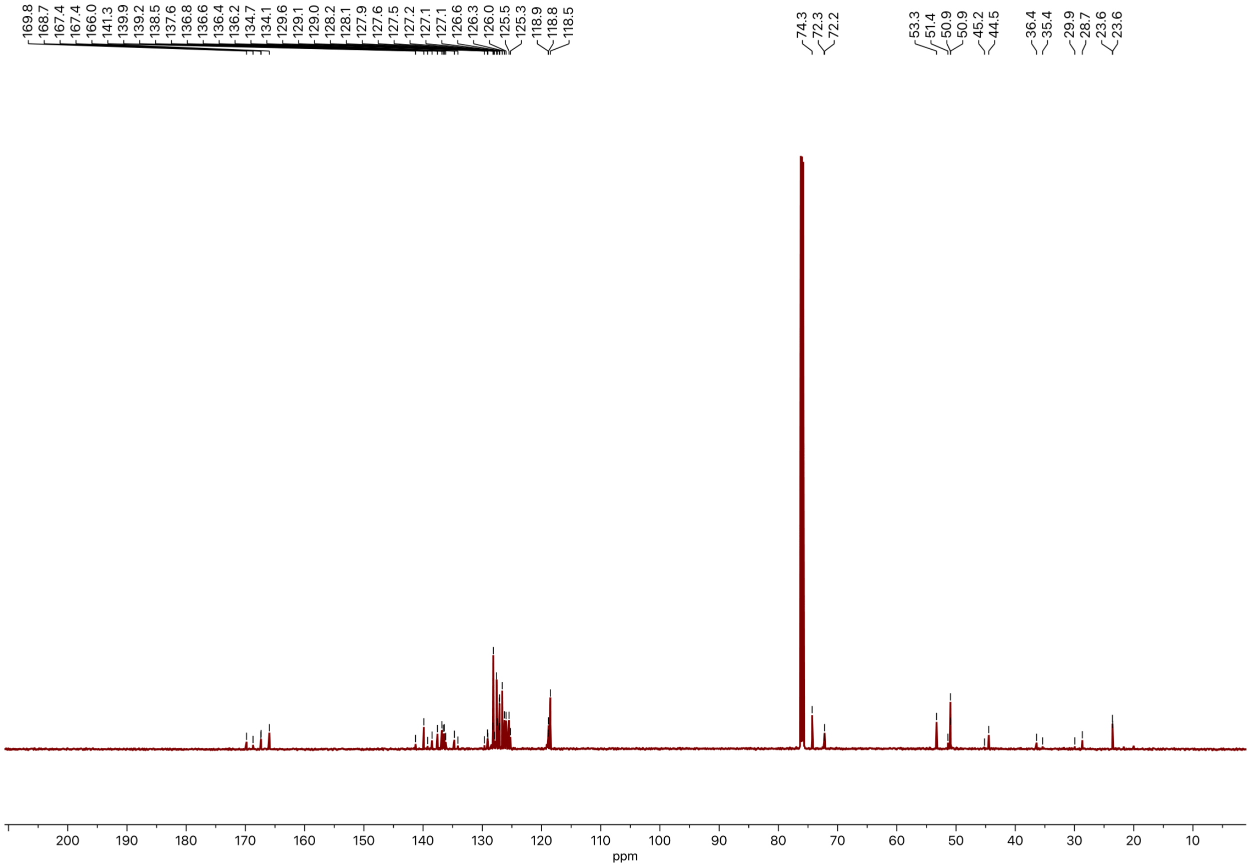

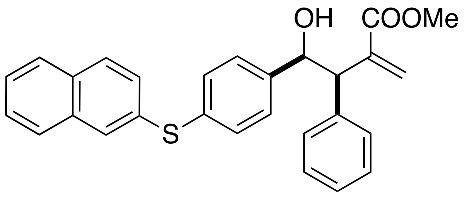

3pa, 61% dr >95:5

^{1}H NMR (400 MHz, $CDCl_3$)

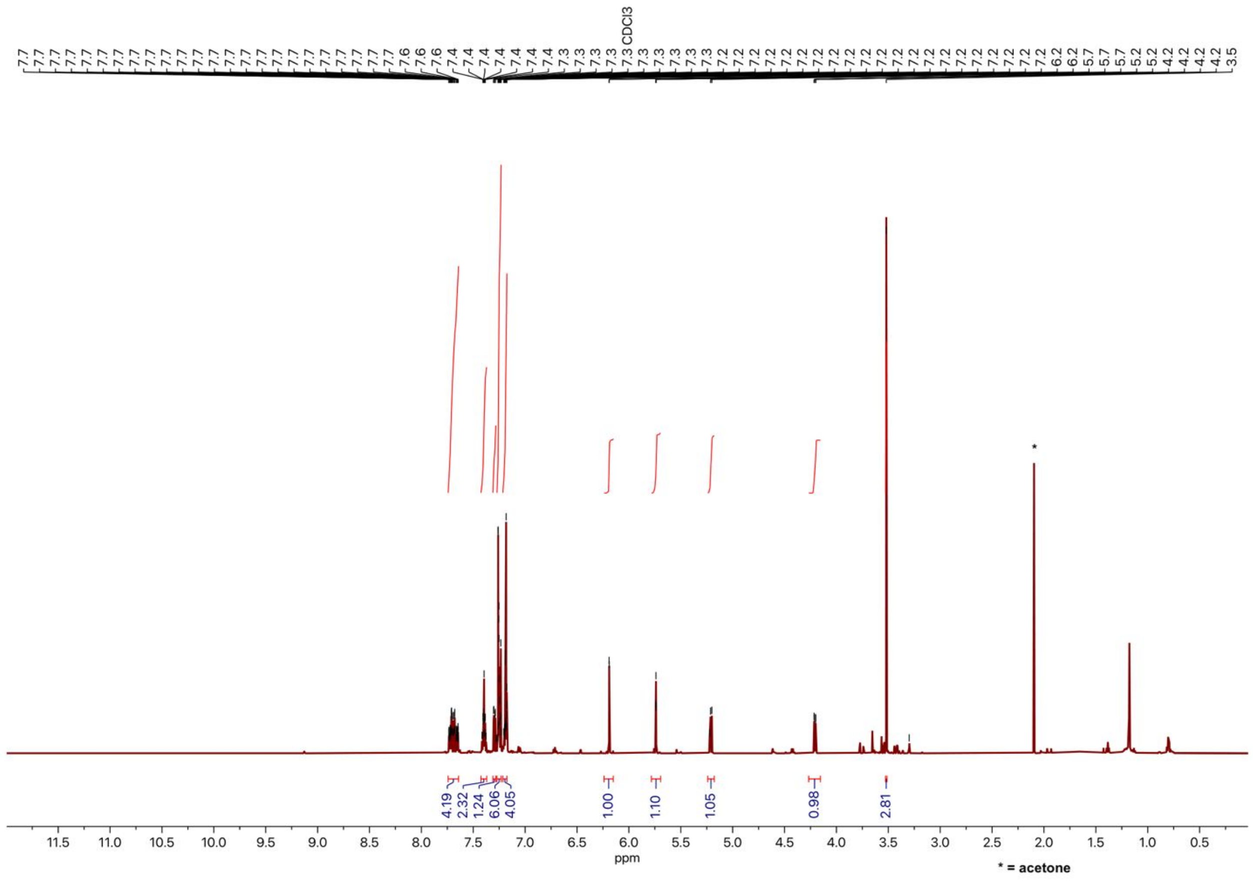

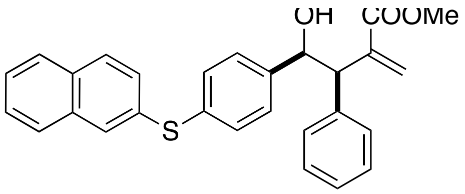

3pa, 61% dr >95:5

^{13}C{^{1}H} NMR (100 MHz, $CDCl_3$)

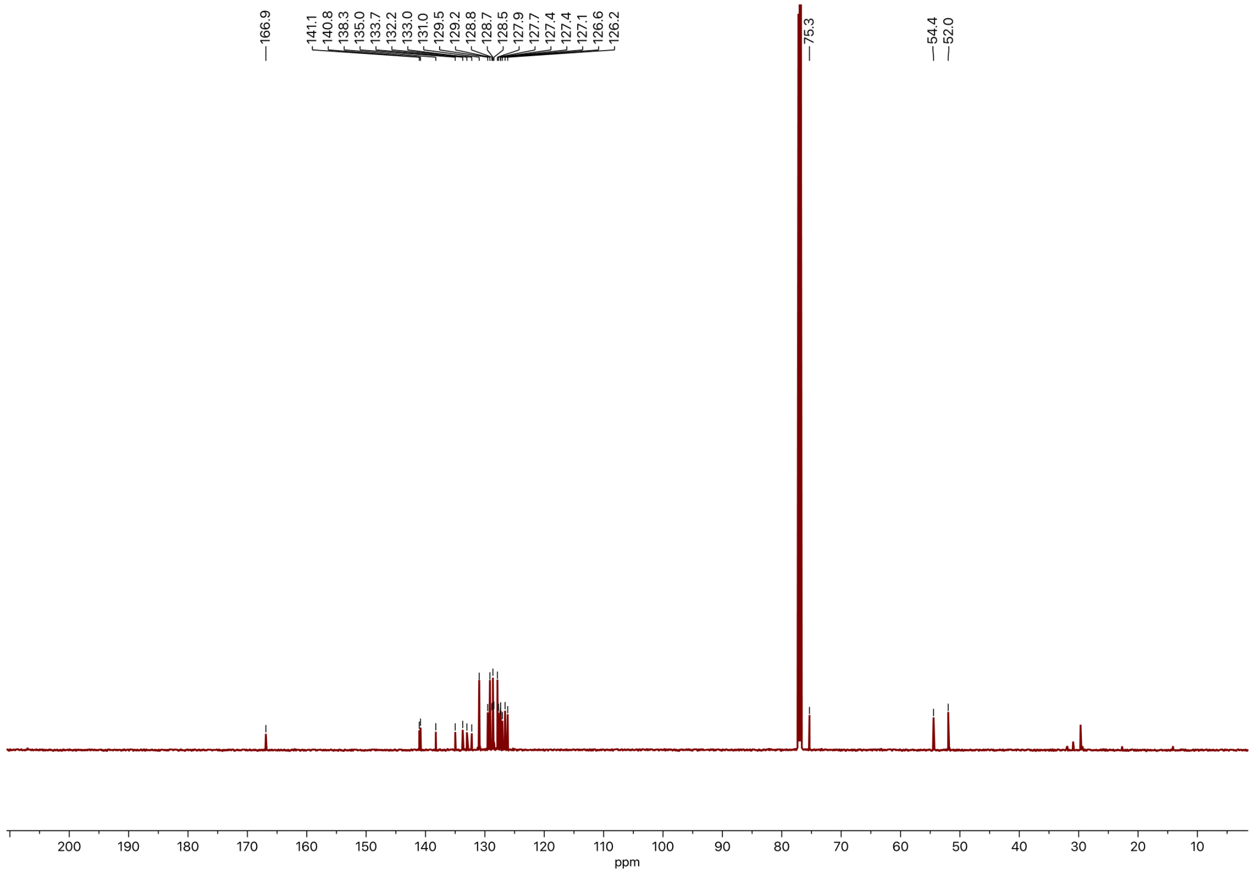

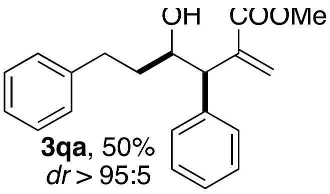

3qa, 50%
dr > 95:5

^{1}**H NMR** (400 MHz, $CDCl_3$)

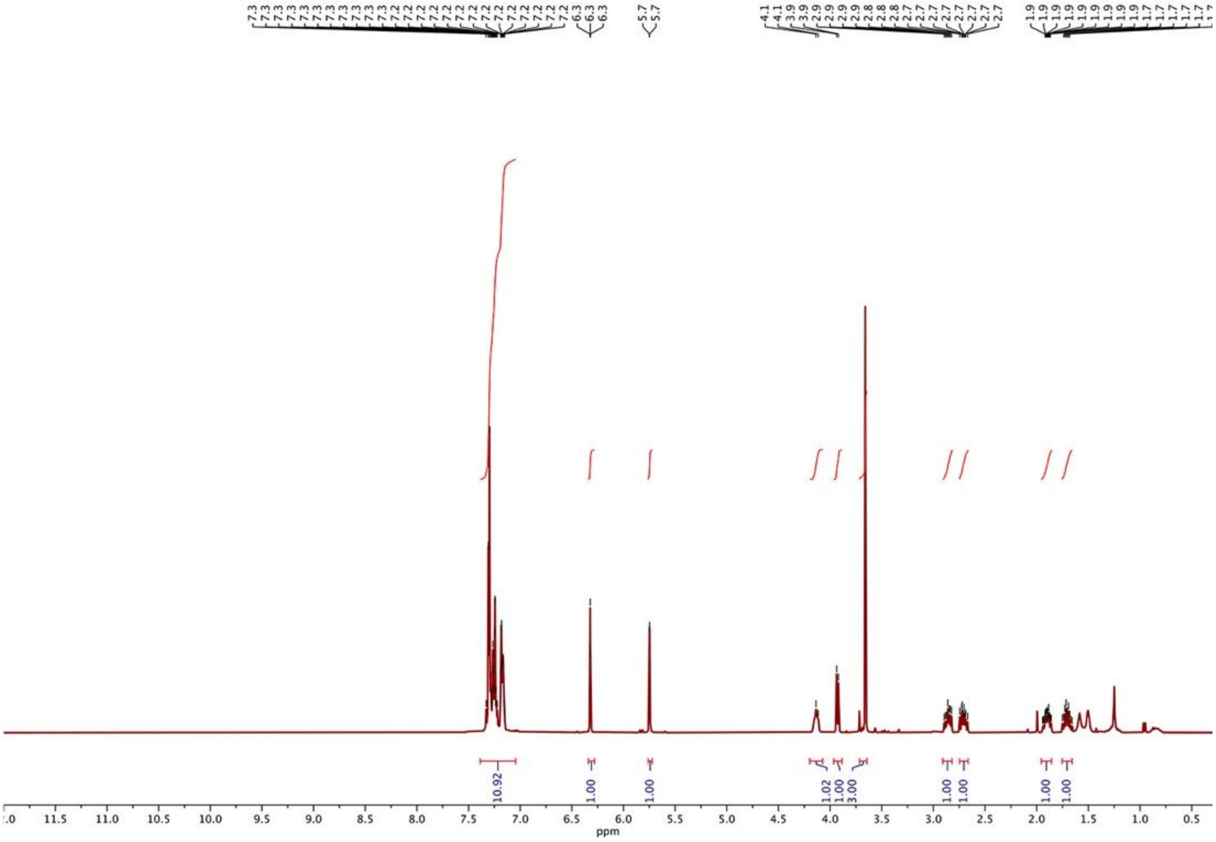

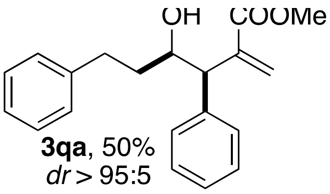

3qa, 50%
dr > 95:5

$^{13}C\{^{1}H\}$ NMR (100 MHz, $CDCl_3$)

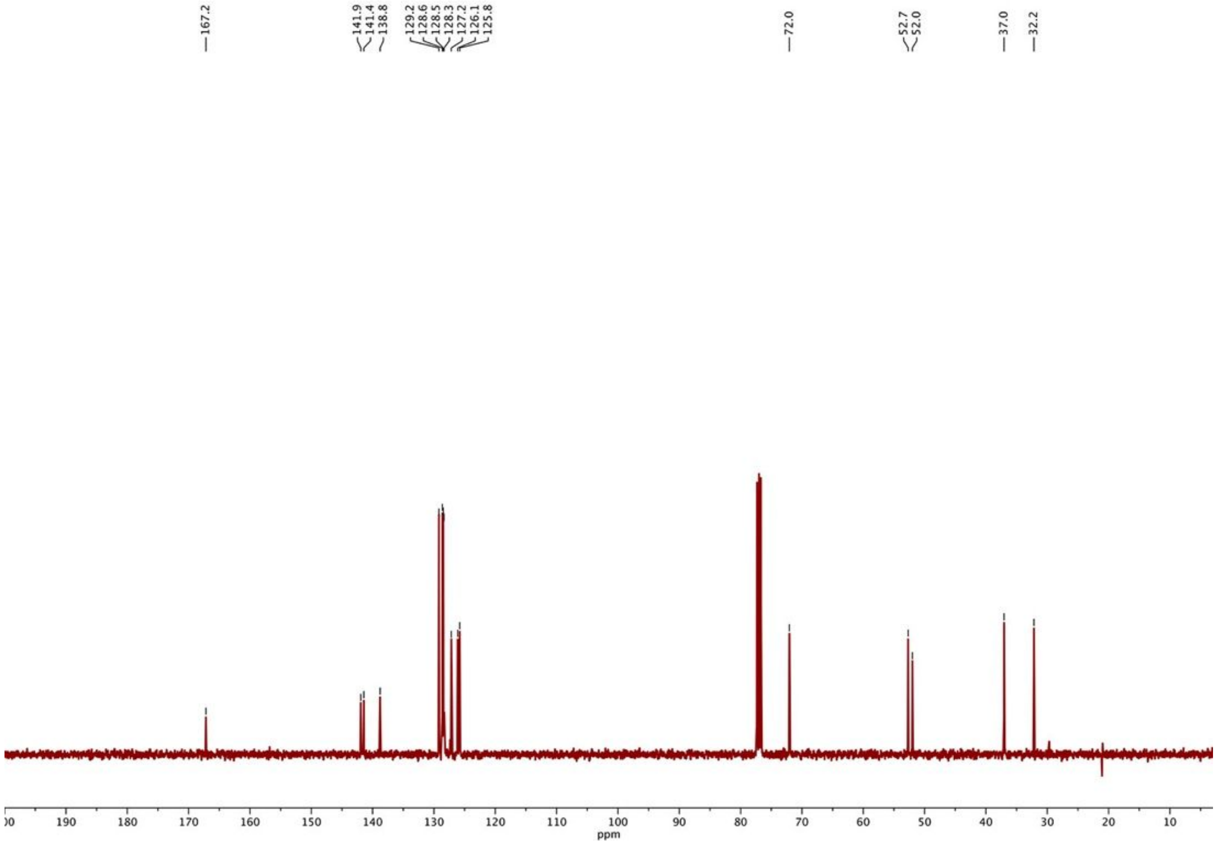

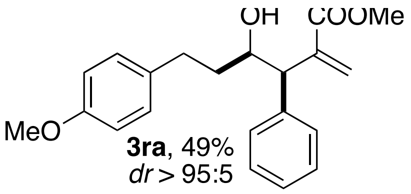

3ra, 49%
dr > 95:5

^{1}H NMR (400 MHz, $CDCl_3$)

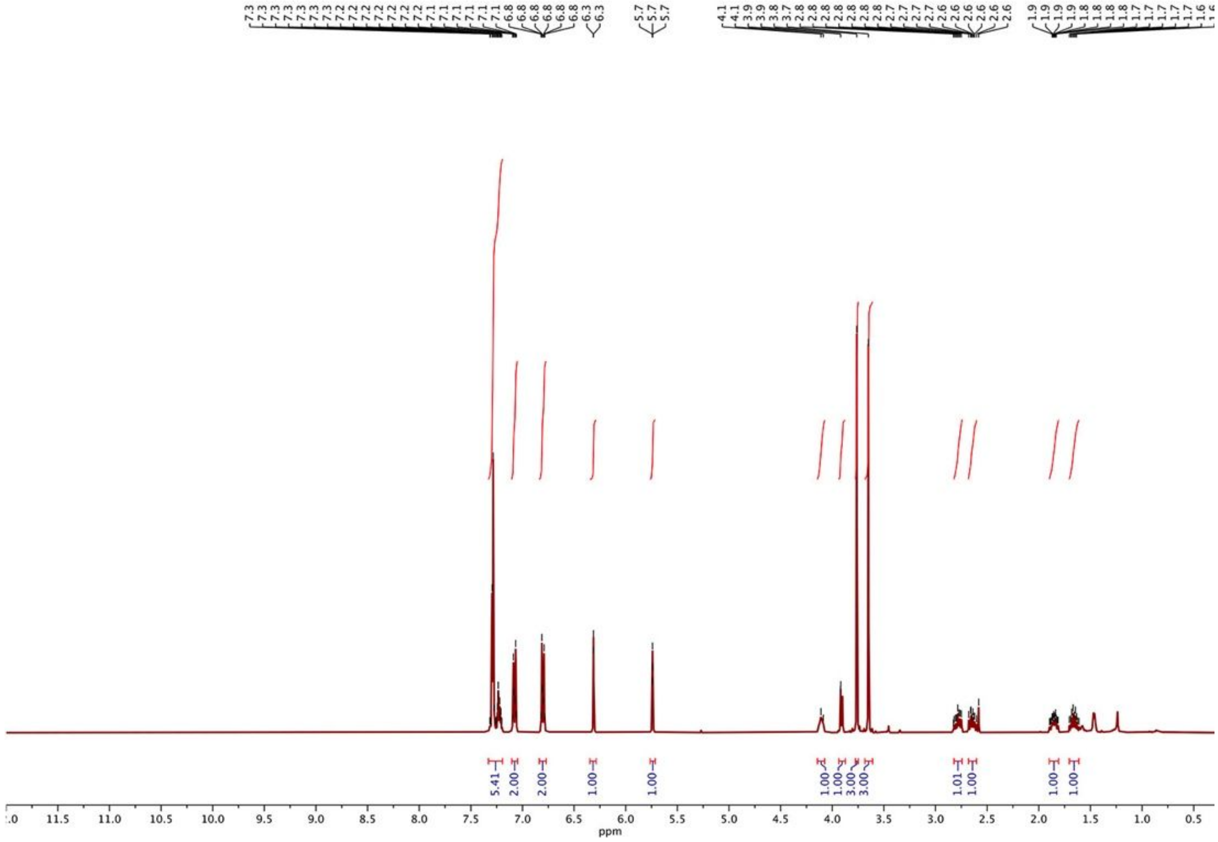

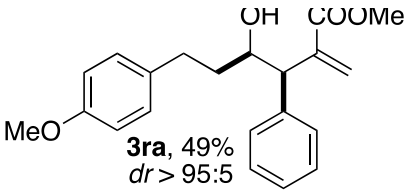

3ra, 49%
dr > 95:5

^{13}**C{^{1}H} NMR** (100 MHz, $CDCl_3$)

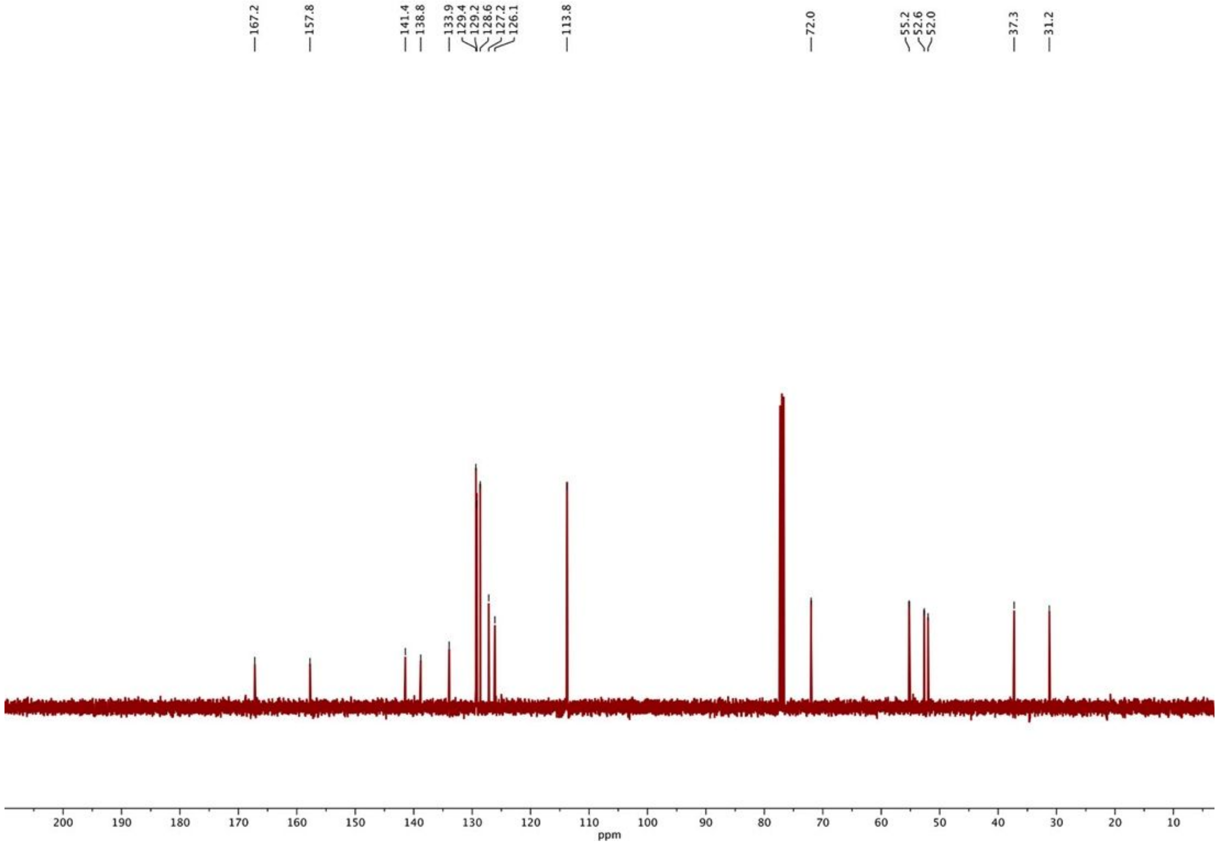

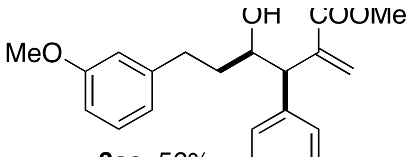

3sa, 56%
dr > 95:5

^{1}H NMR (400 MHz, $CDCl_3$)

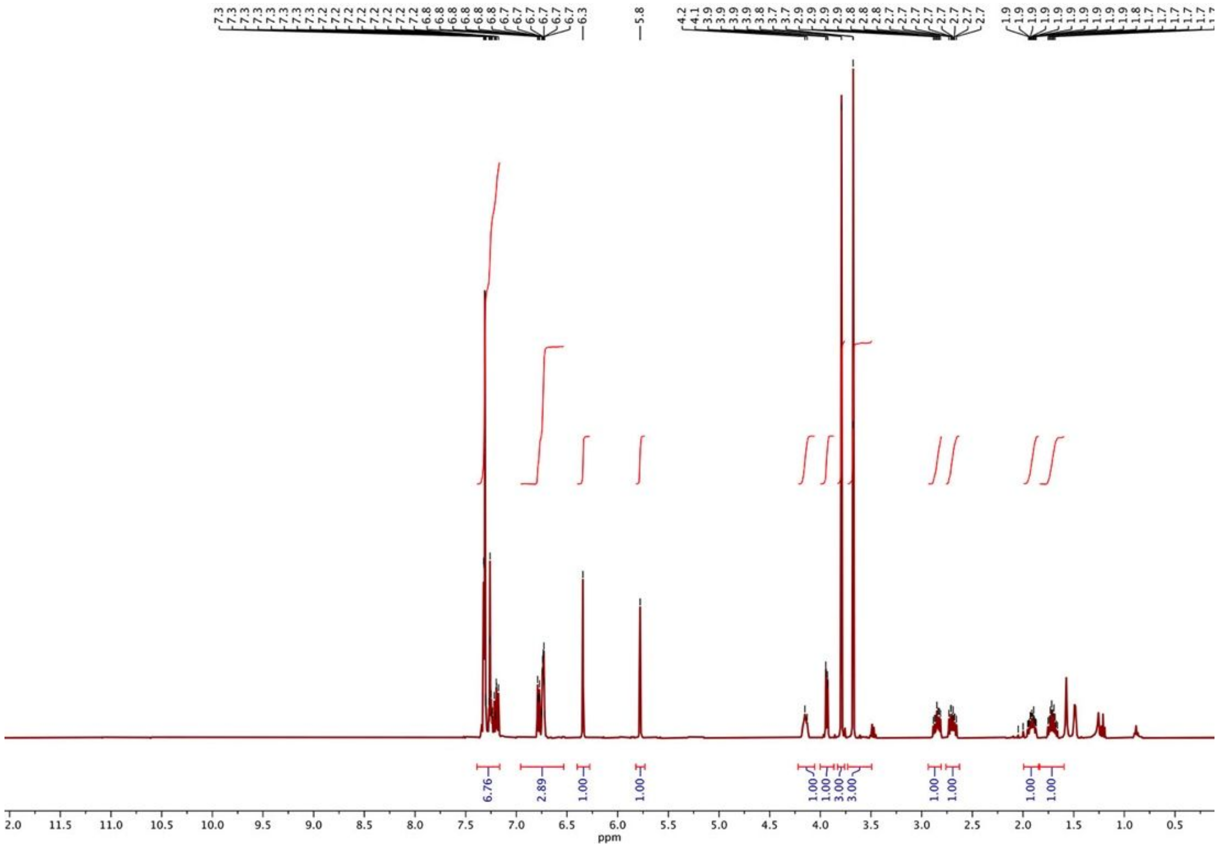

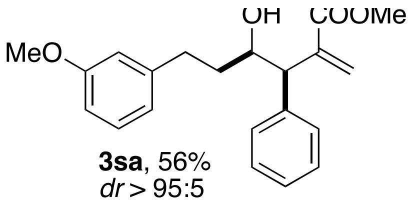

3sa, 56%
dr > 95:5

$^{13}C\{^1H\}$ NMR (100 MHz, $CDCl_3$)

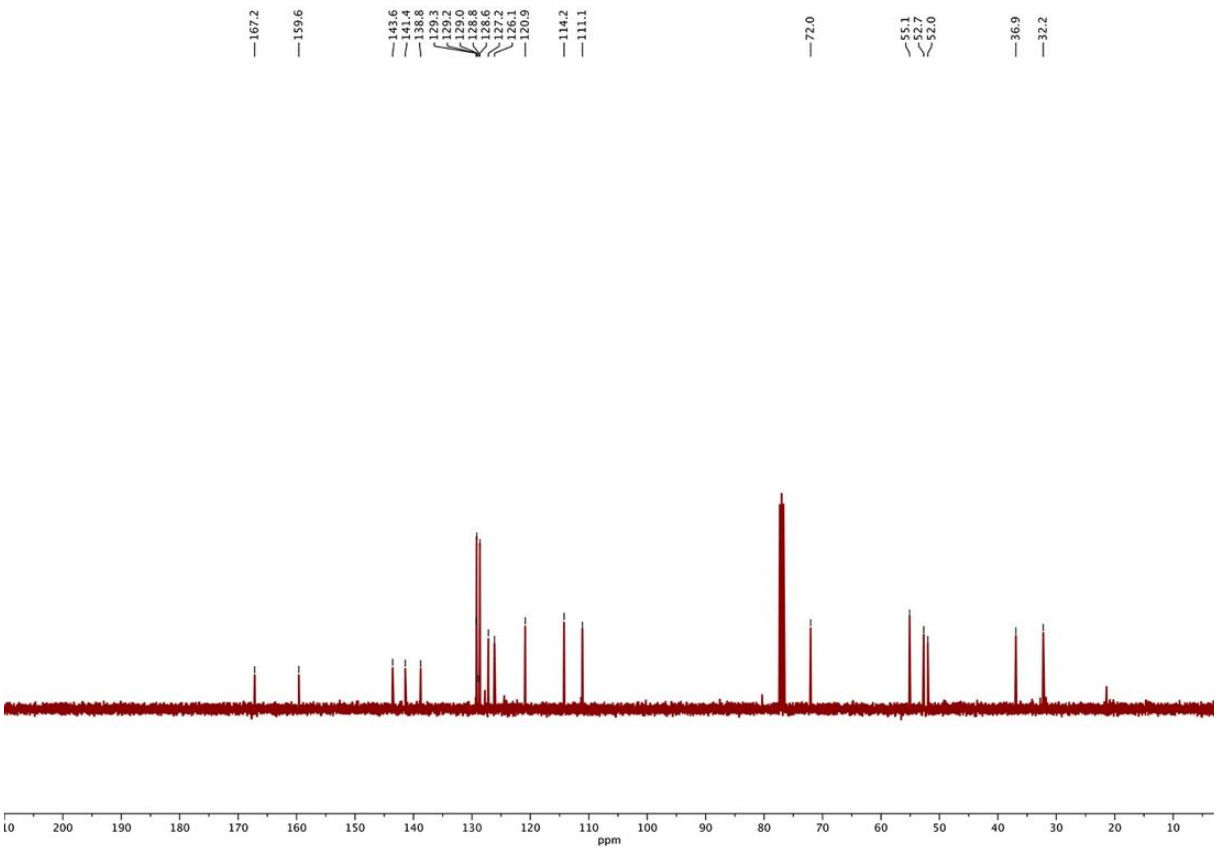

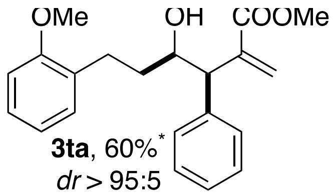

3ta, 60%*
dr > 95:5

^{1}H NMR (400 MHz, $CDCl_3$)

^{1}H NMR (400 MHz, $CDCl_3$)

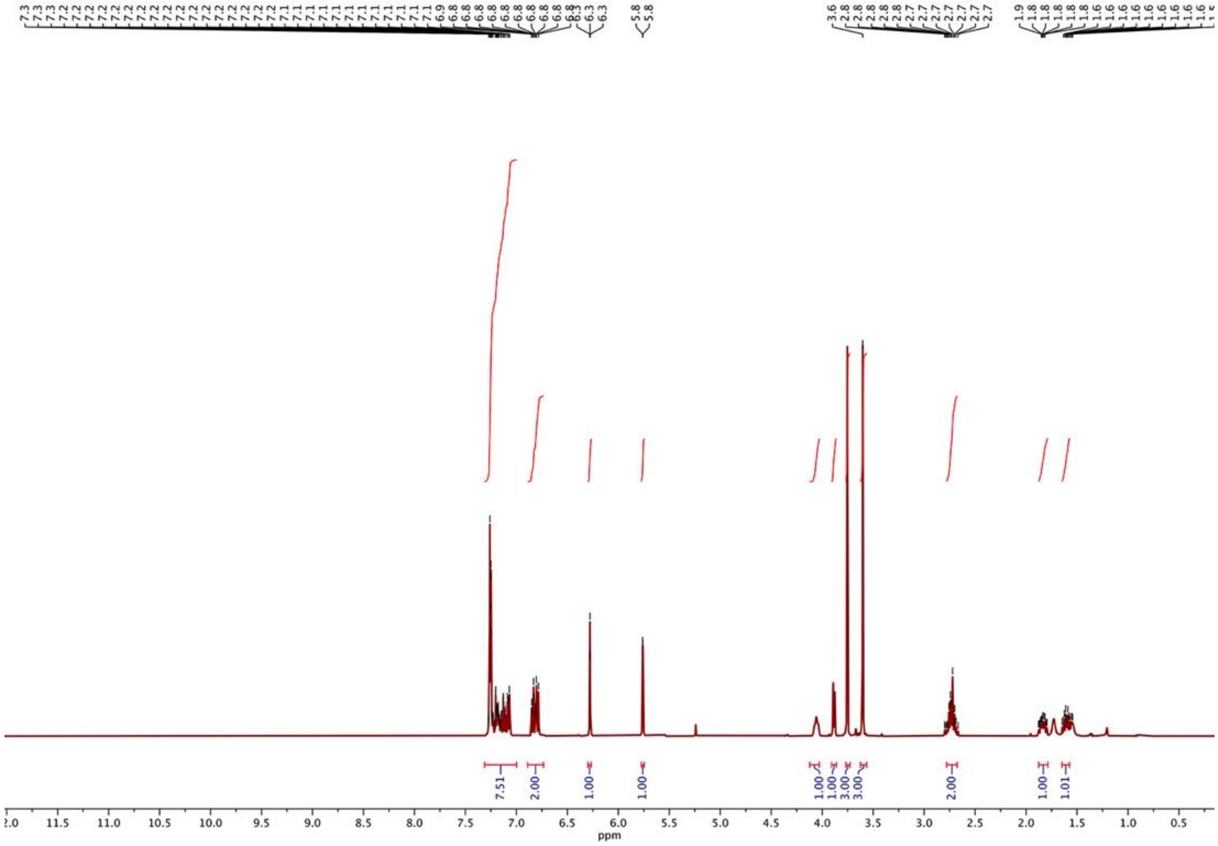

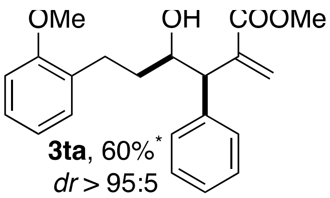

3ta, 60%*
dr > 95:5

^{13}**C{^{1}H} NMR** (100 MHz, $CDCl_3$)

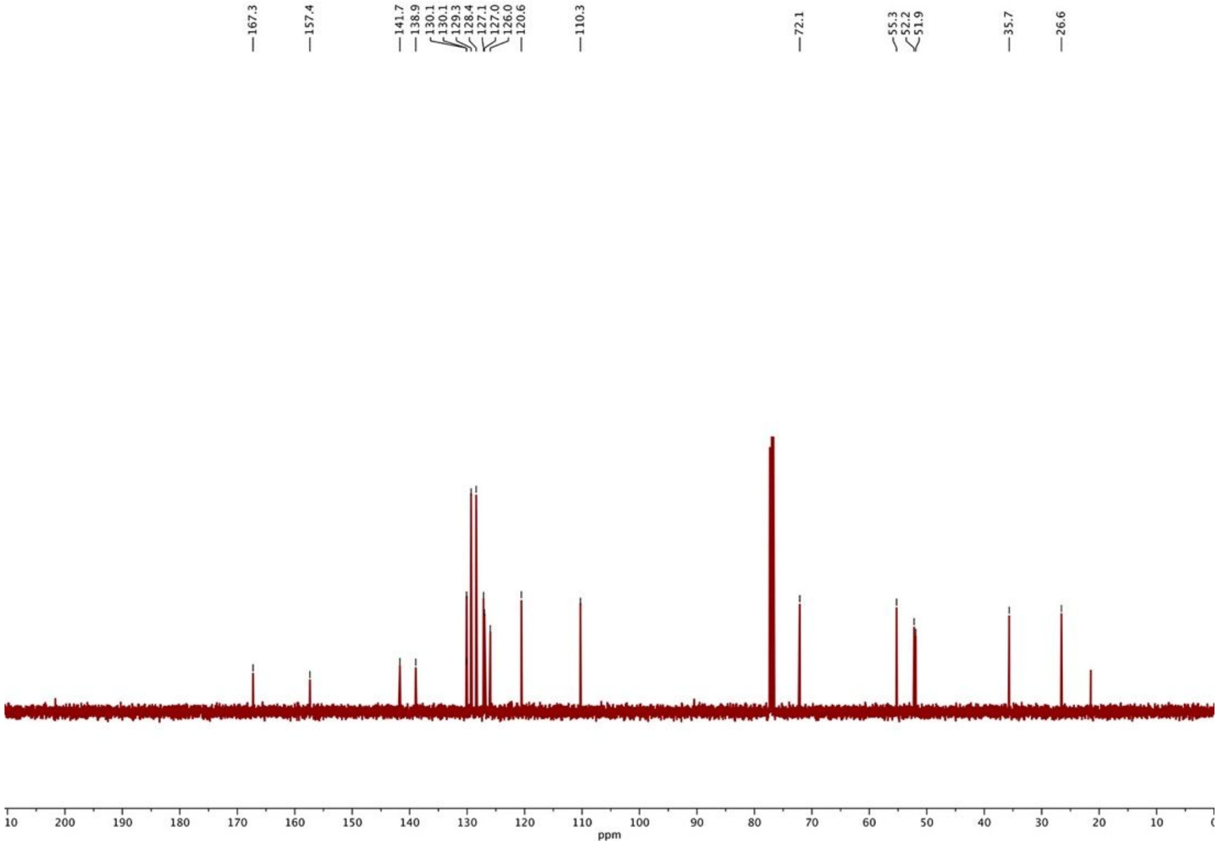

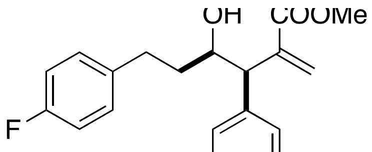

3ua, 34%
dr = 84:16

^{1}H NMR (400 MHz, $CDCl_3$)

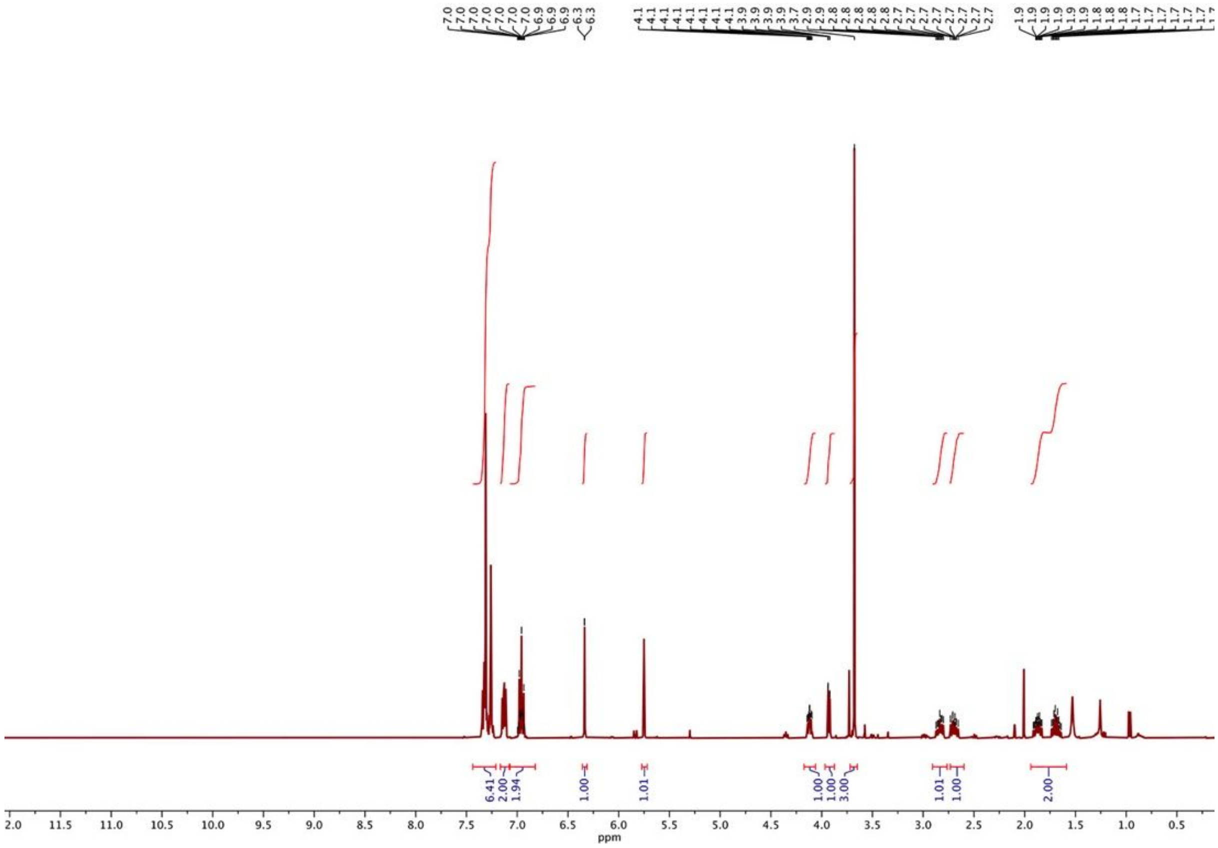

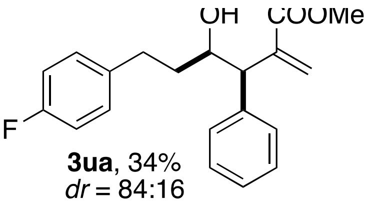

3ua, 34%
dr = 84:16

^{13}C{^{1}H} NMR (100 MHz, $CDCl_3$)

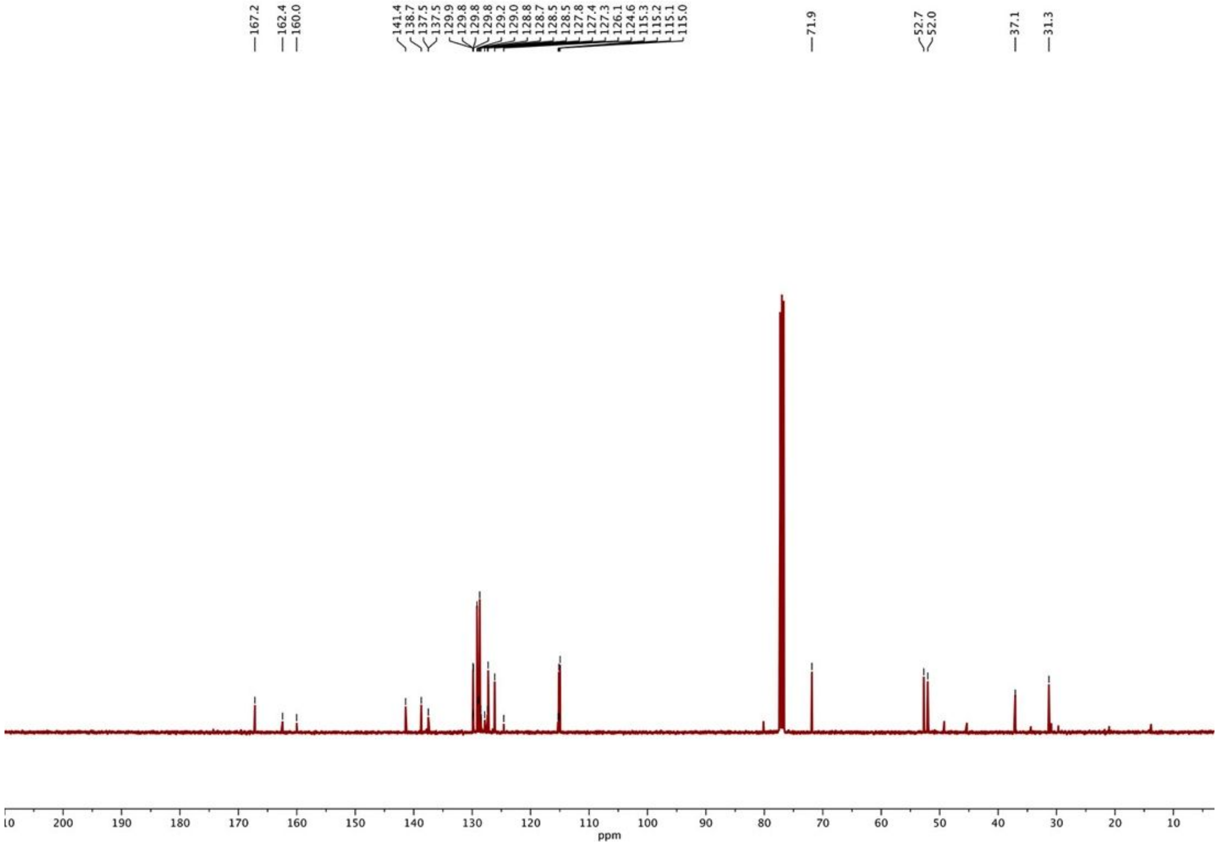

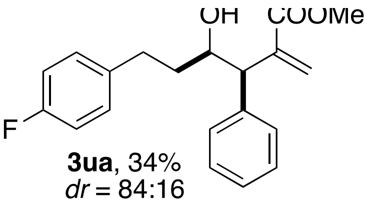

3ua, 34%
dr = 84:16

^{19}**F NMR** (377 MHz, $CDCl_3$)

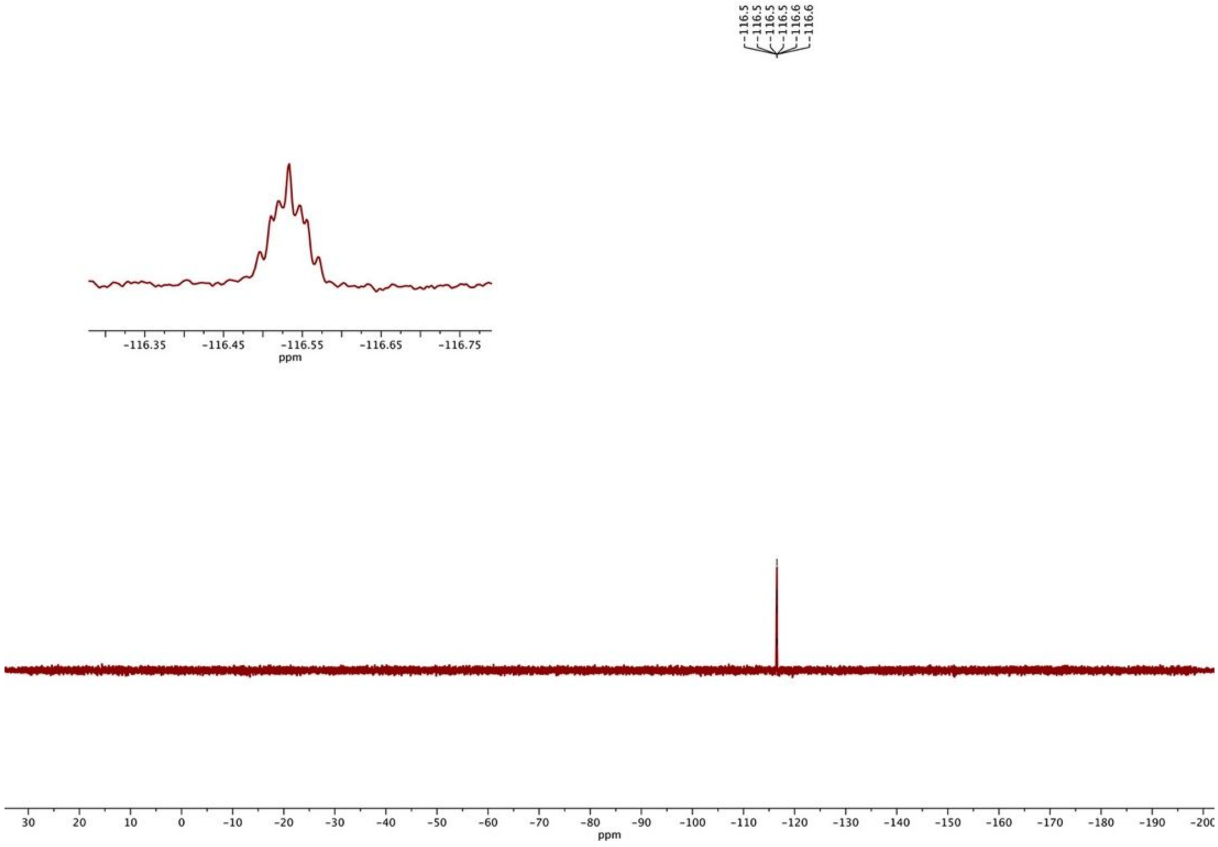

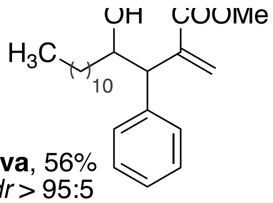

3va, 56%
dr > 95:5

^{1}H NMR (400 MHz, $CDCl_3$)

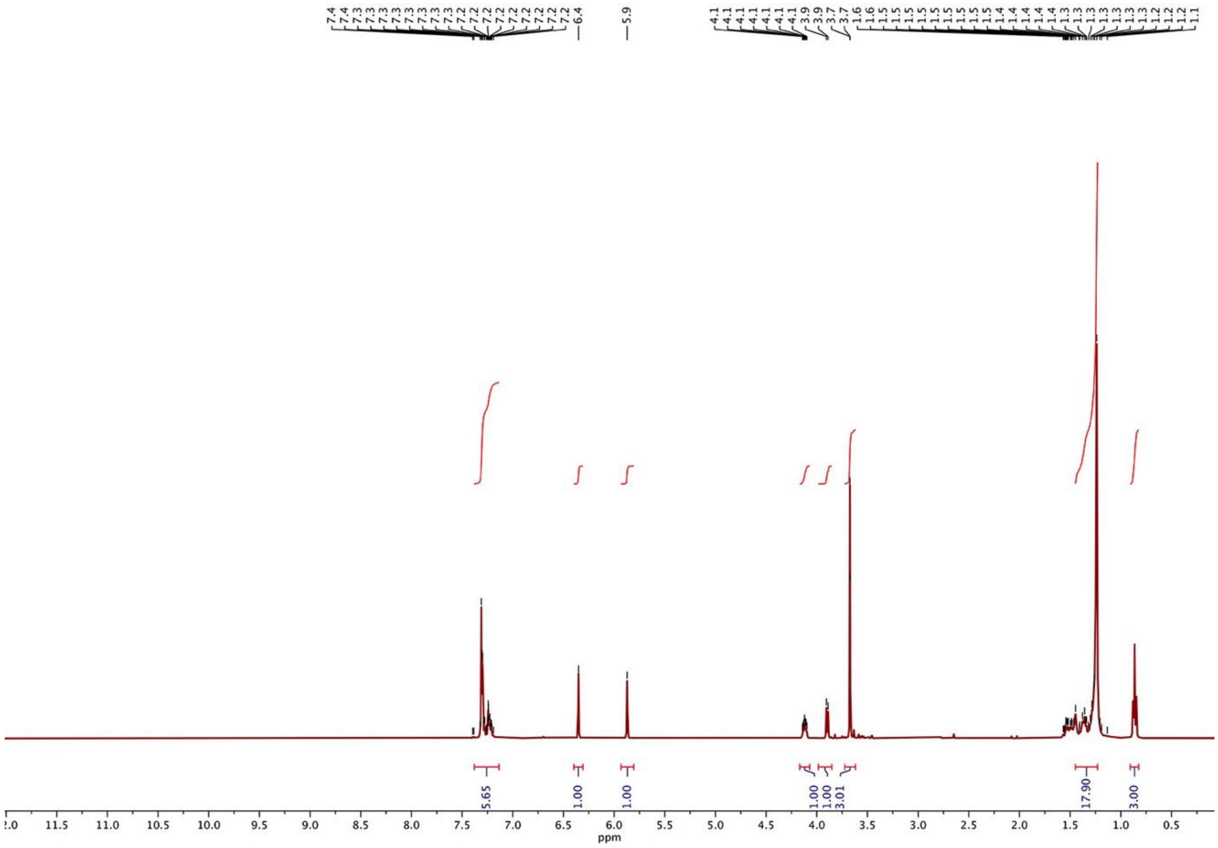

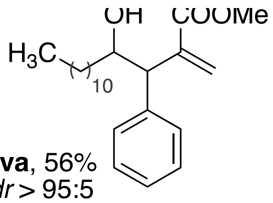

3va, 56%
dr > 95:5

$^{13}C\{^{1}H\}$ NMR (100 MHz, $CDCl_3$)

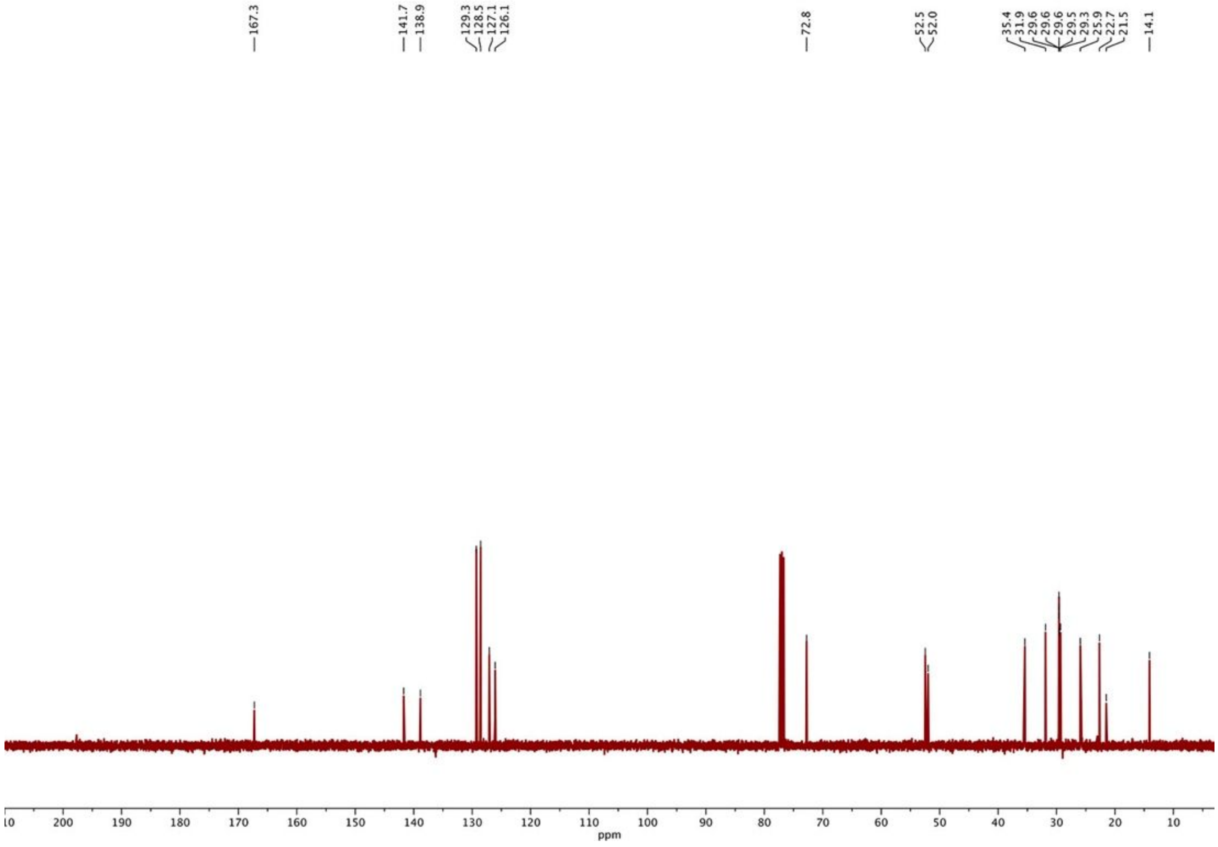

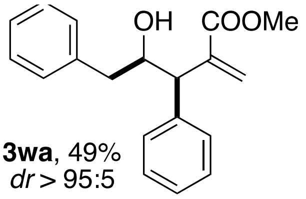

3wa, 49%
dr > 95:5

^{1}H NMR (400 MHz, $CDCl_3$)

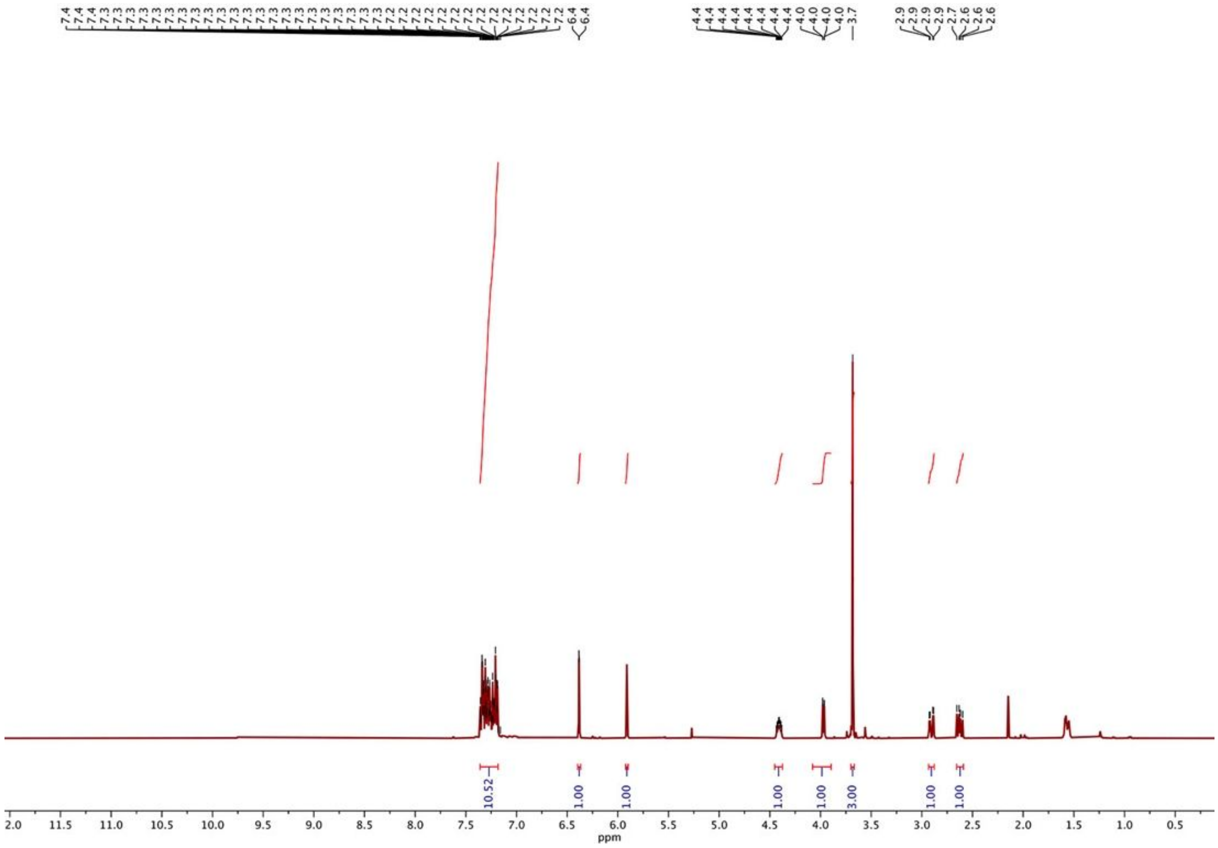

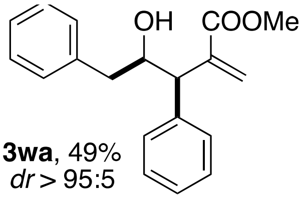

3wa, 49%
dr > 95:5

$^{13}C\{^{1}H\}$ NMR (100 MHz, $CDCl_3$)

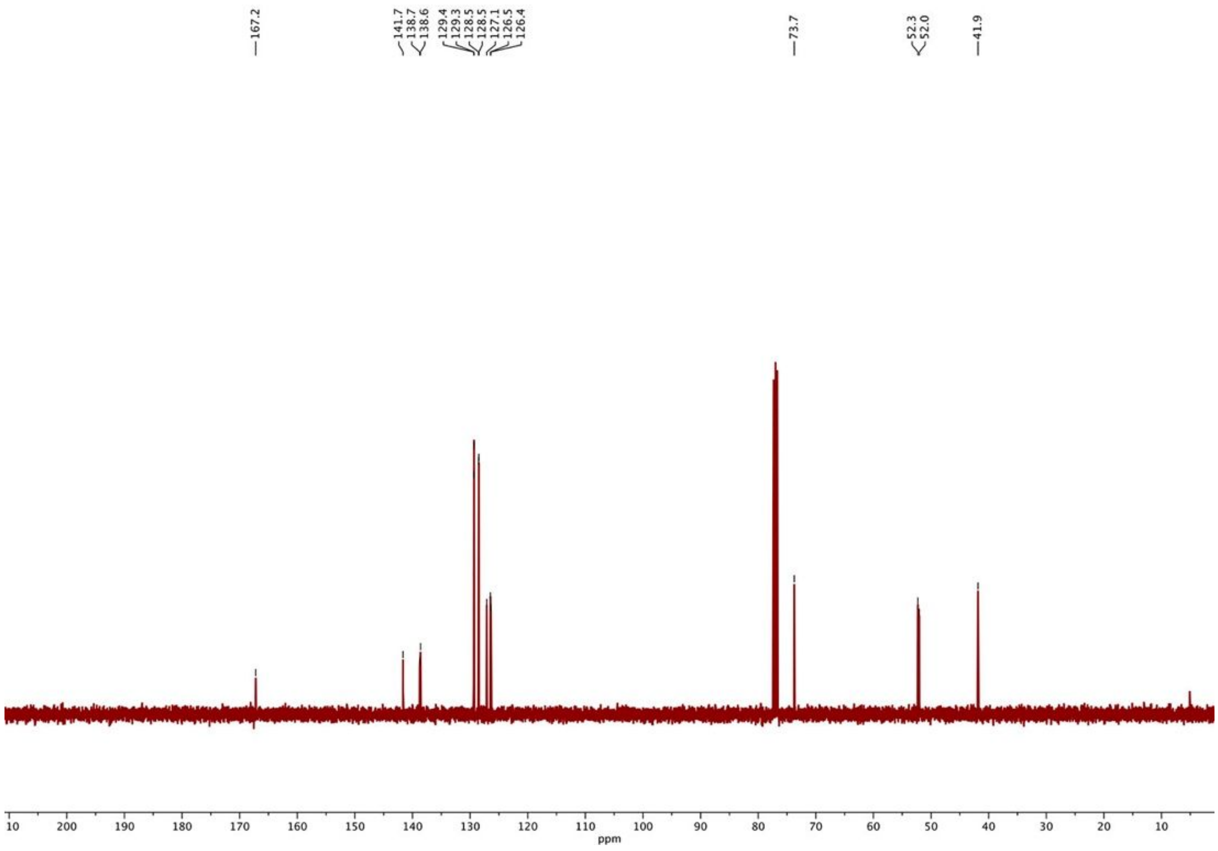

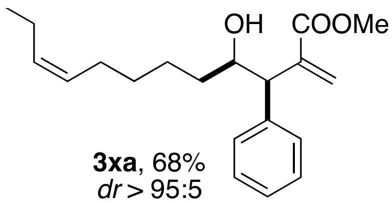

3xa, 68%
dr > 95:5

^{1}H NMR (400 MHz, $CDCl_3$)

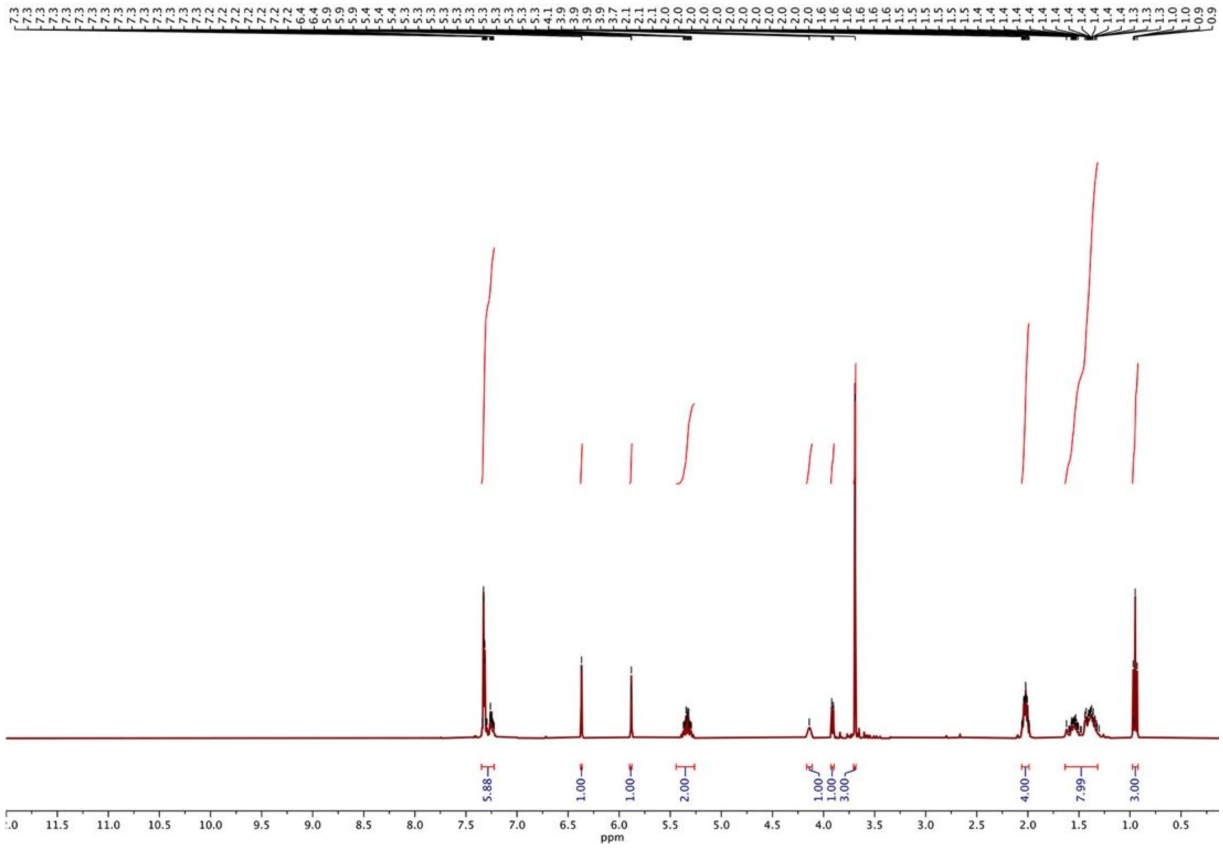

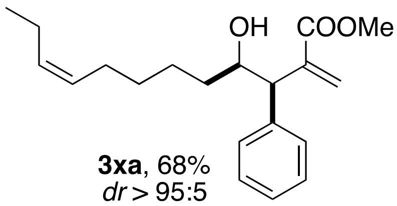

3xa, 68%
dr > 95:5

^{13}C{^{1}H} NMR (100 MHz, $CDCl_3$)

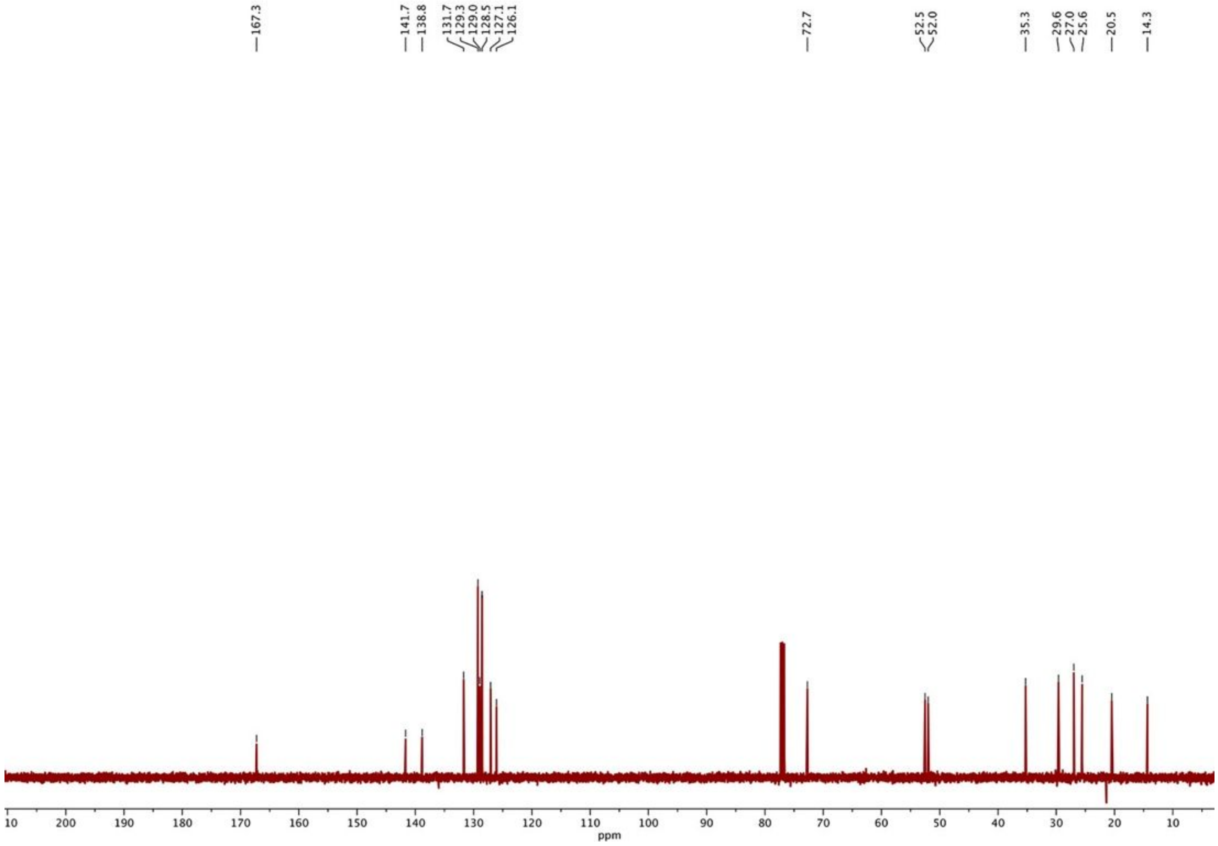

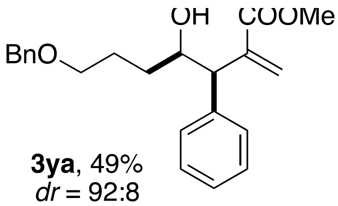

3ya, 49%
dr = 92:8

¹H NMR (400 MHz, $CDCl_3$)

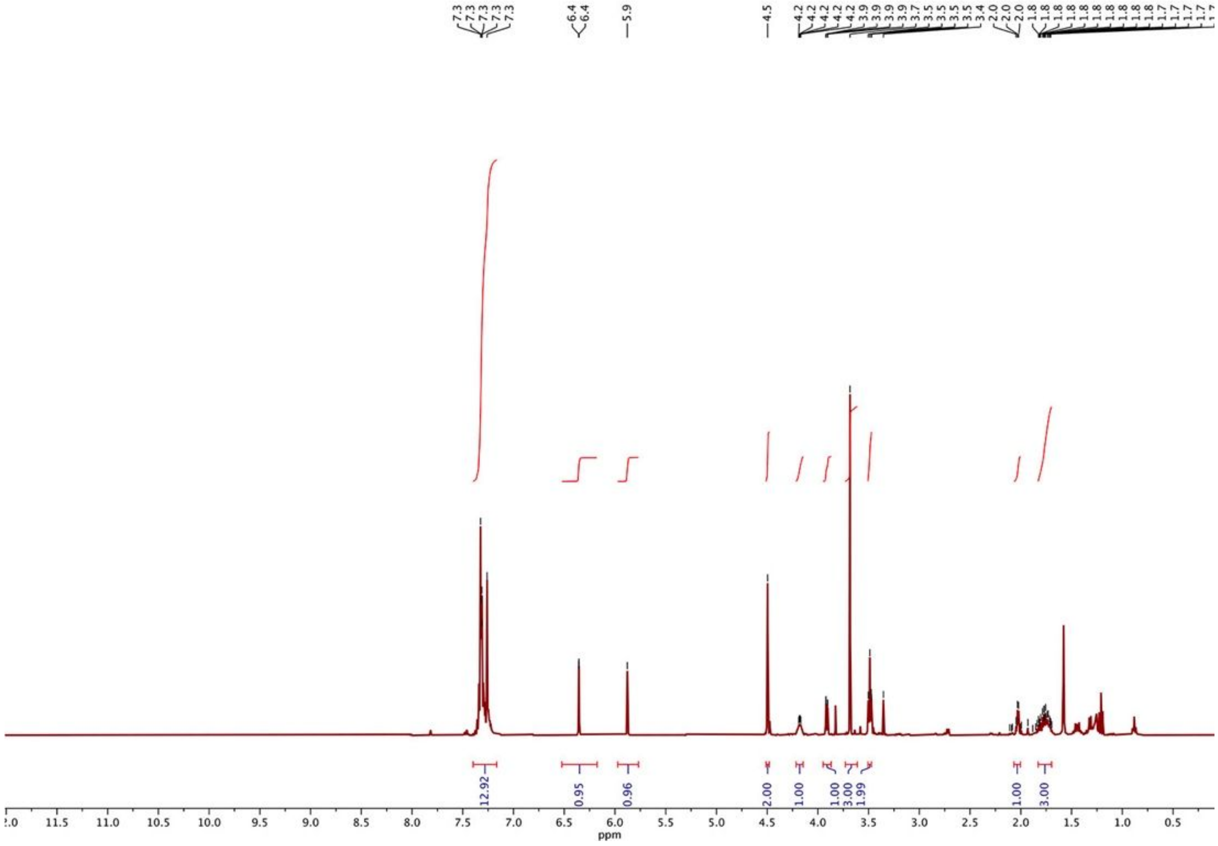

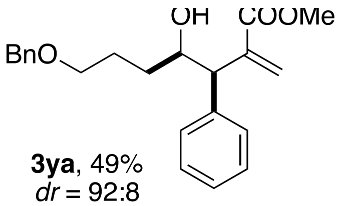

3ya, 49%
dr = 92:8

^{13}C{^{1}H} NMR (100 MHz, CDCl$_3$)

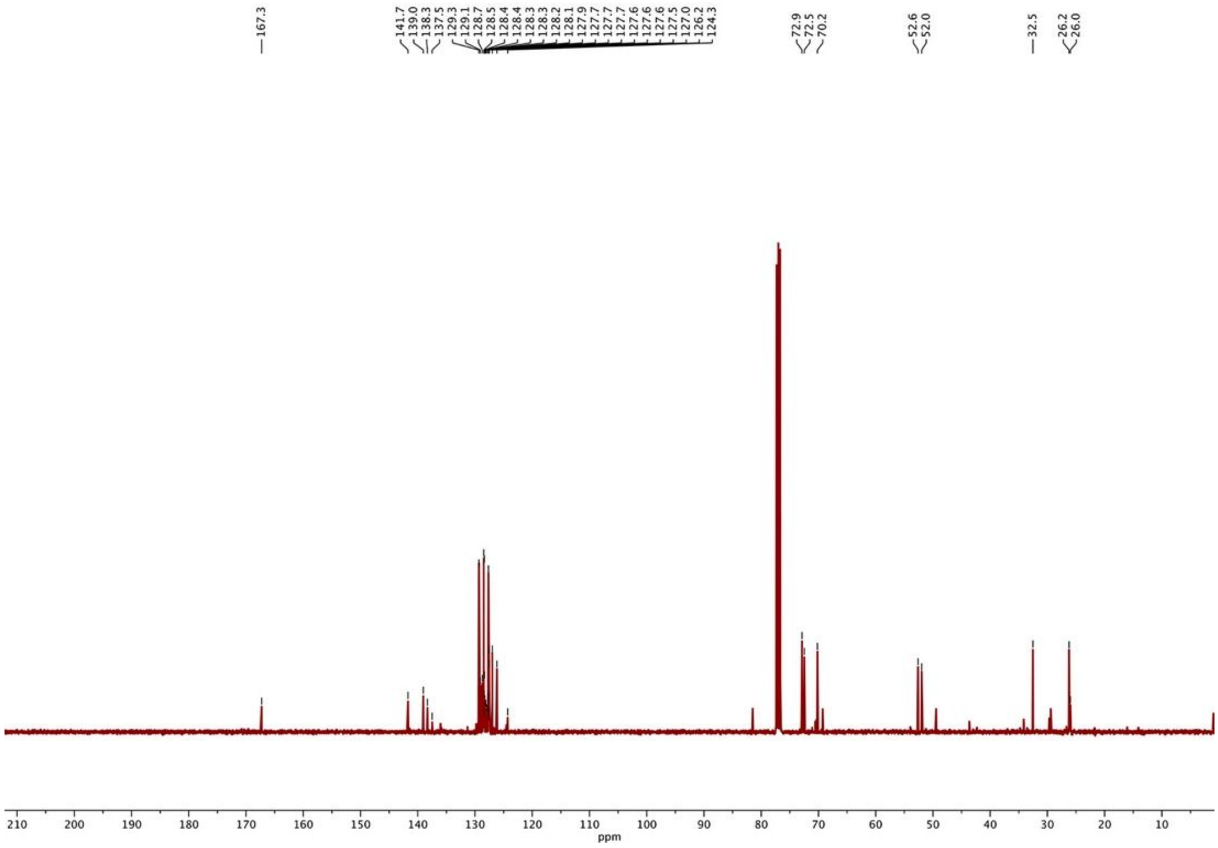

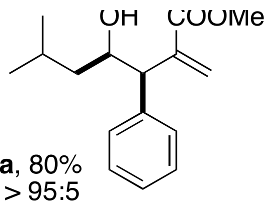

3za, 80%
dr > 95:5

^{1}H NMR (400 MHz, $CDCl_3$)

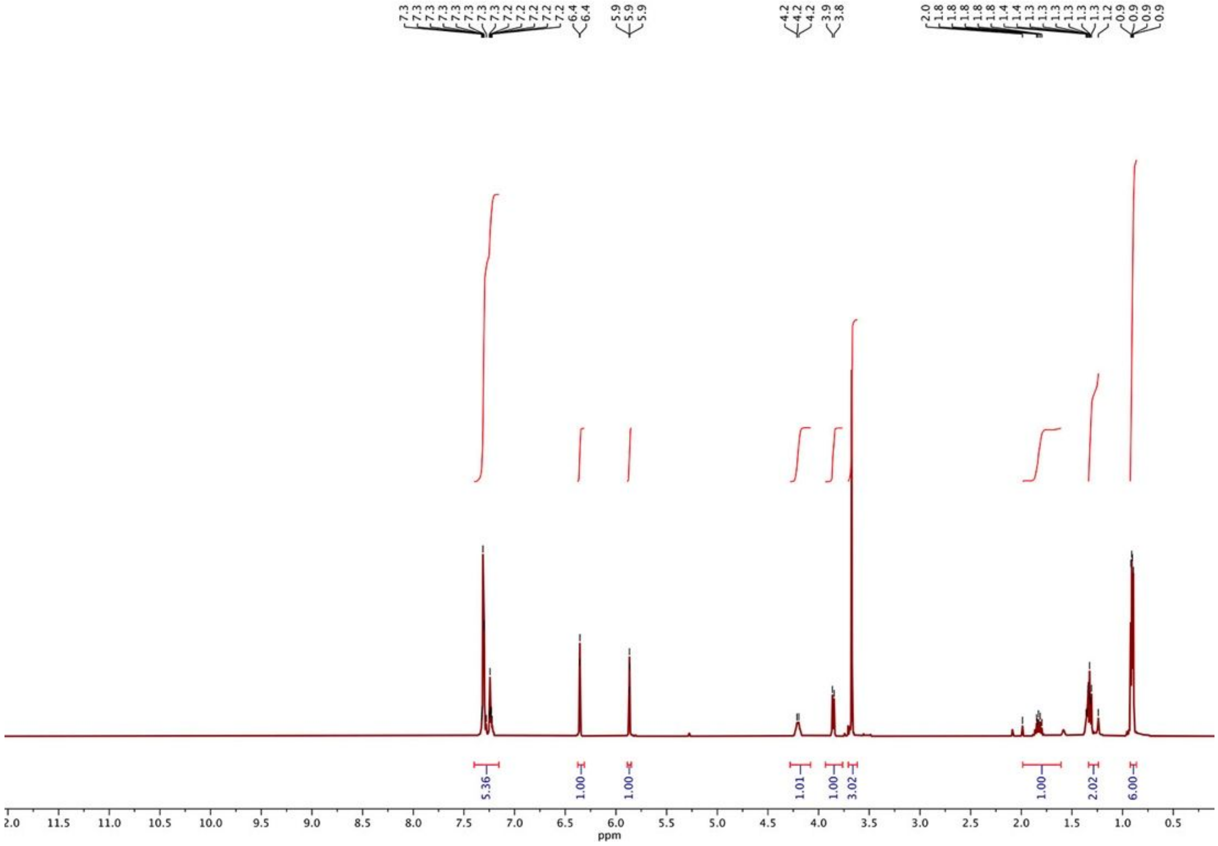

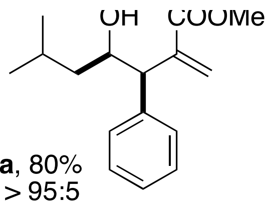

3za, 80%
dr > 95:5

^{13}C{^{1}H} NMR (100 MHz, $CDCl_3$)

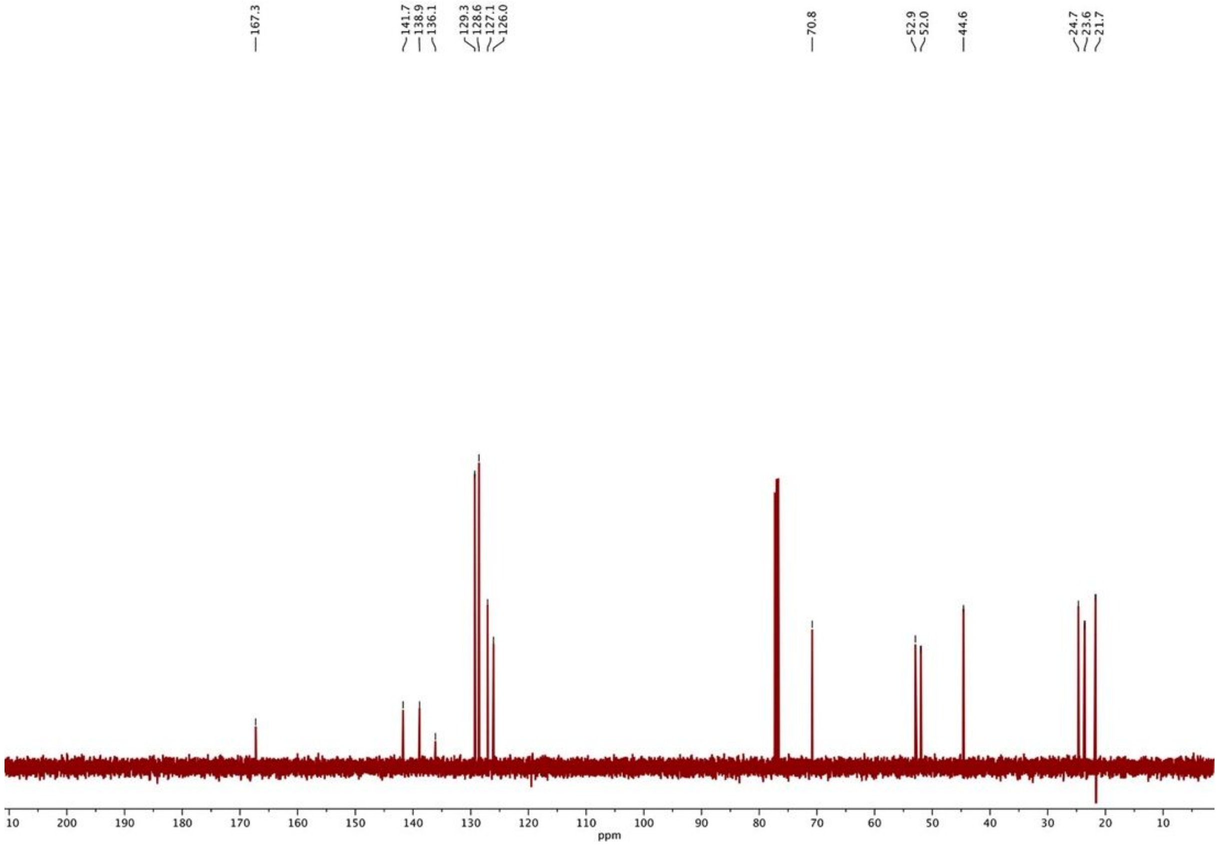

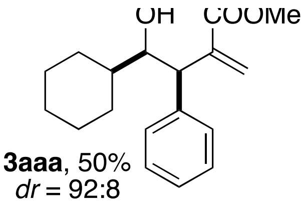

3aaa, 50%
dr = 92:8

^{1}H NMR (400 MHz, $CDCl_3$)

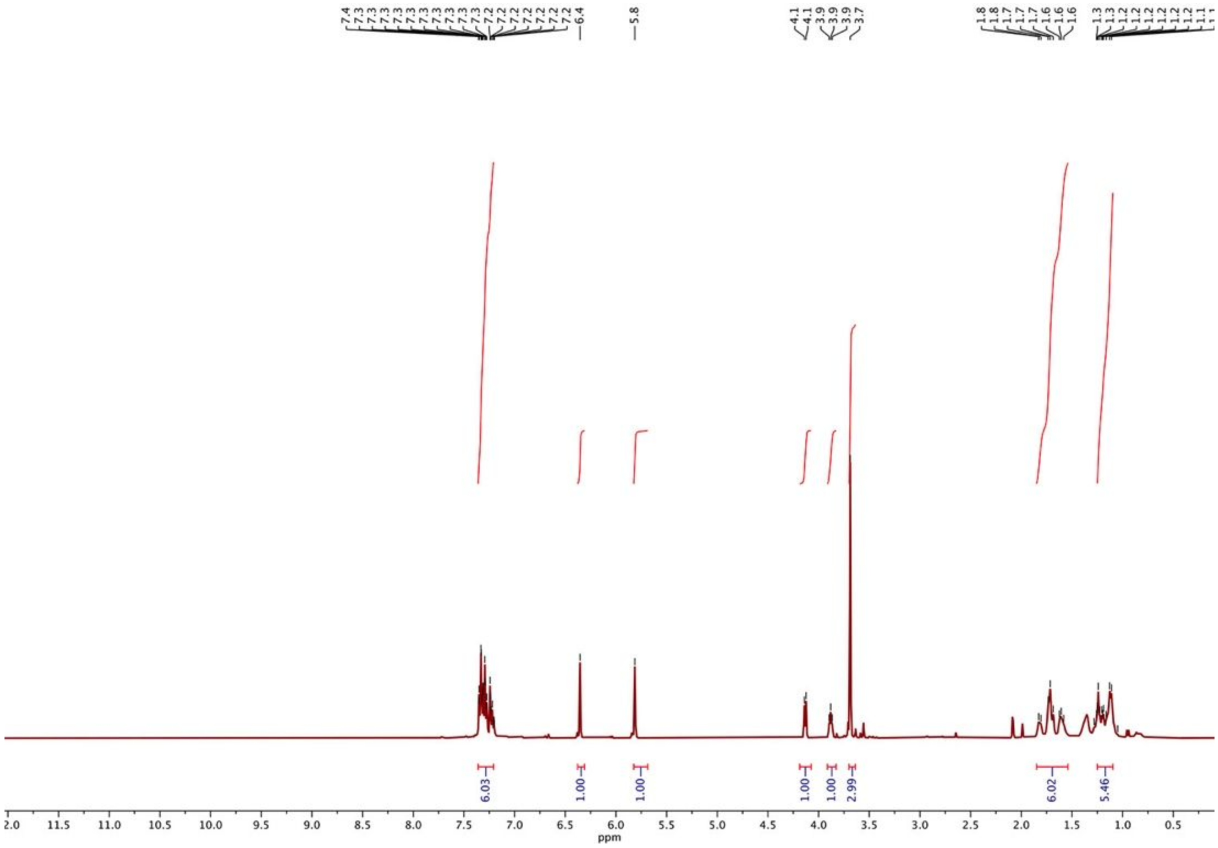

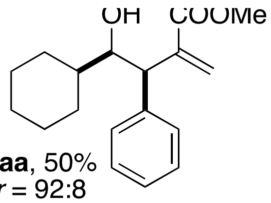

3aaa, 50%
dr = 92:8

$^{13}C\{^1H\}$ NMR (100 MHz, $CDCl_3$)

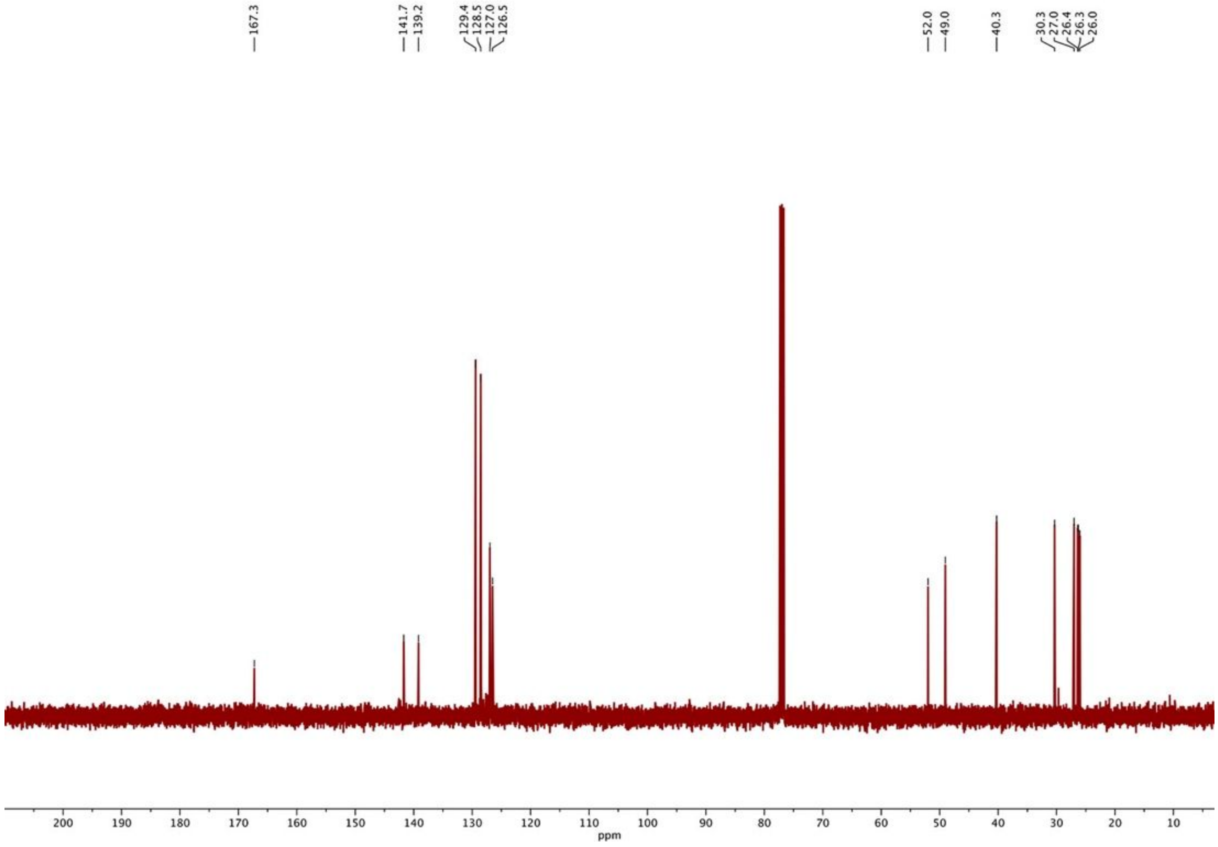

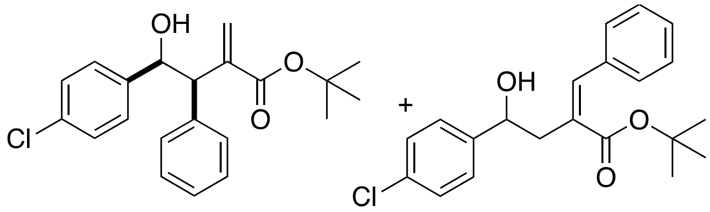

3ae+ 3ae', 81% *dr> 95:5* *rr* = 80:20

^{1}H NMR (400 MHz, $CDCl_3$)

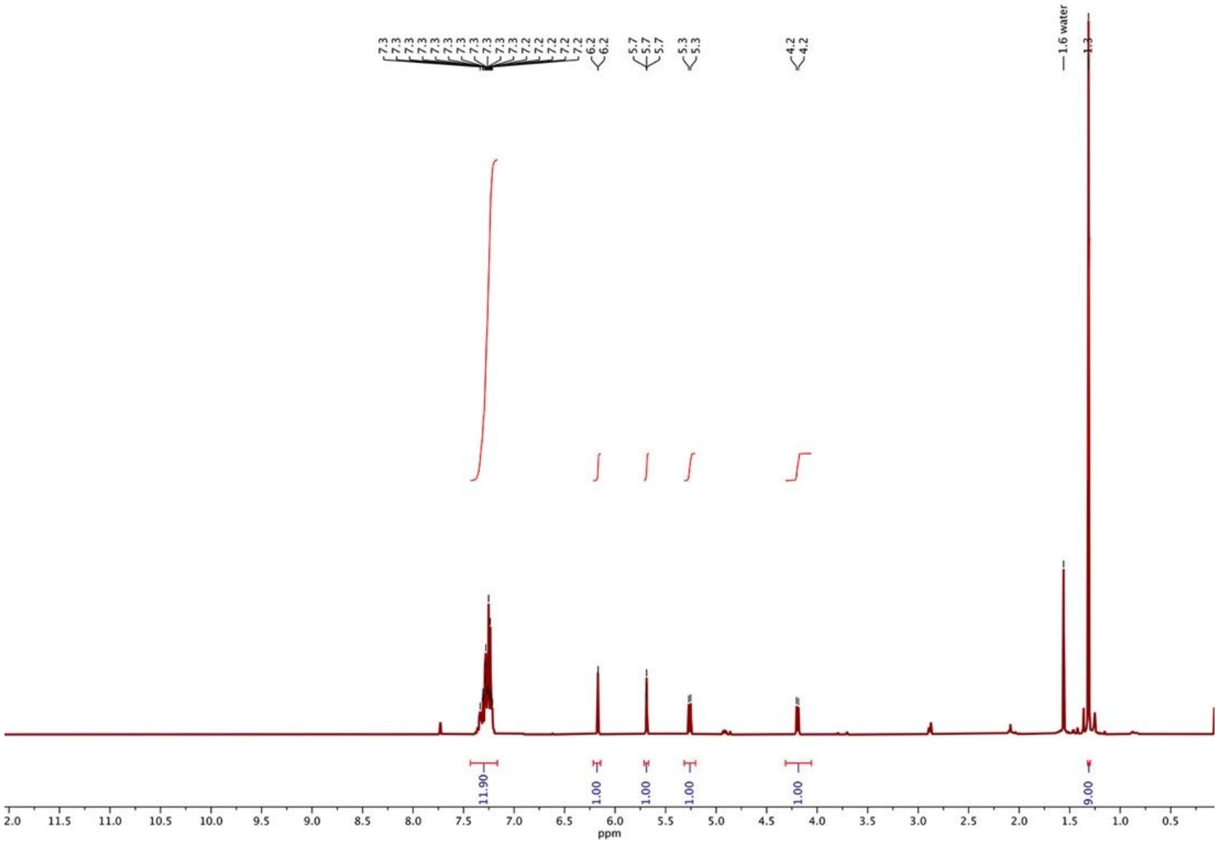

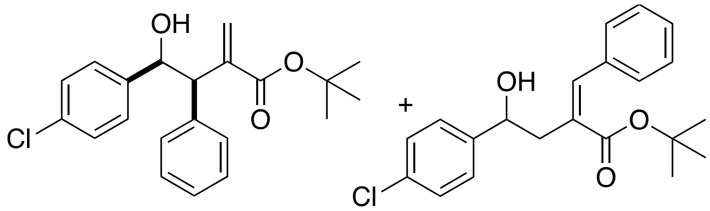

3ae+ 3ae', 81% *dr> 95:5* *rr* = 80:20

^{13}C{^{1}H} NMR (100 MHz, $CDCl_3$)

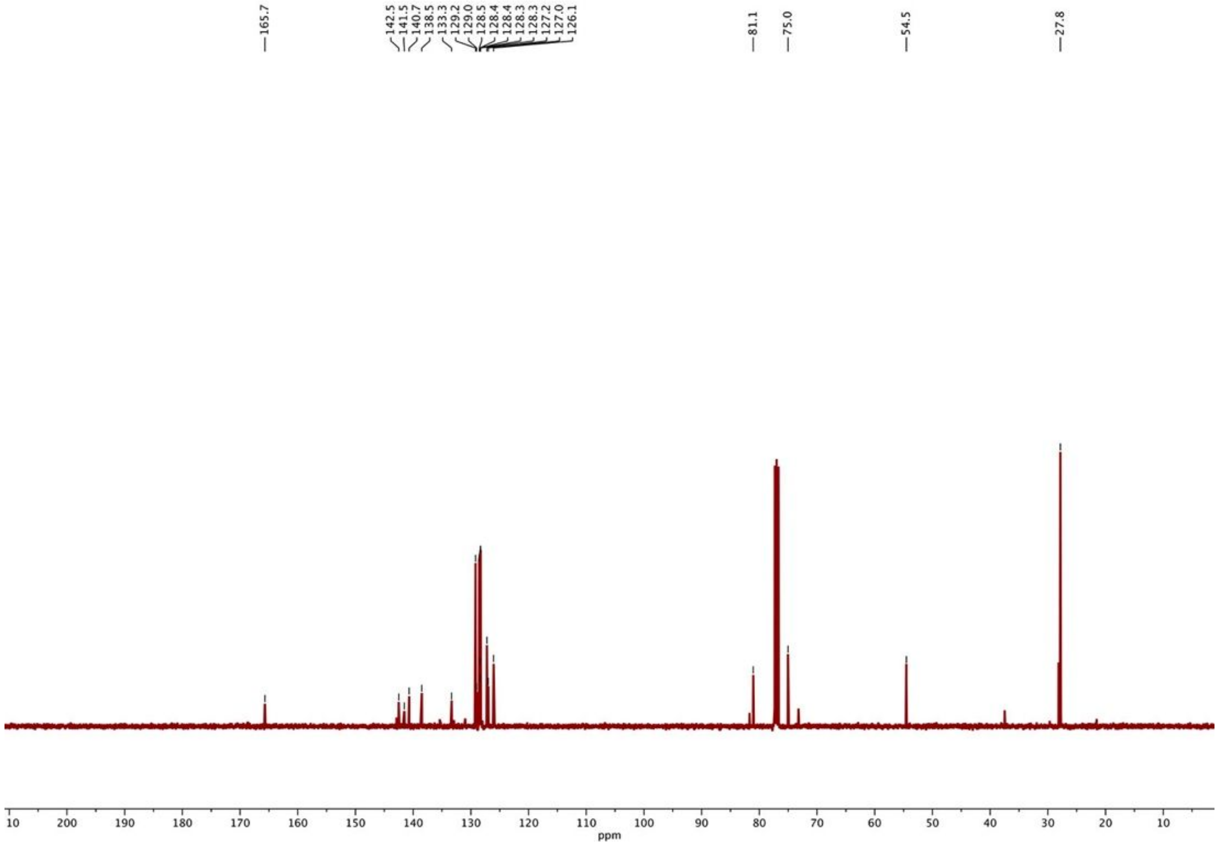

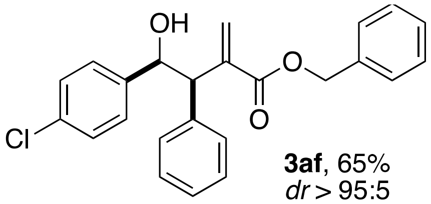

3af, 65%
dr > 95:5

^{1}H NMR (400 MHz, $CDCl_3$)

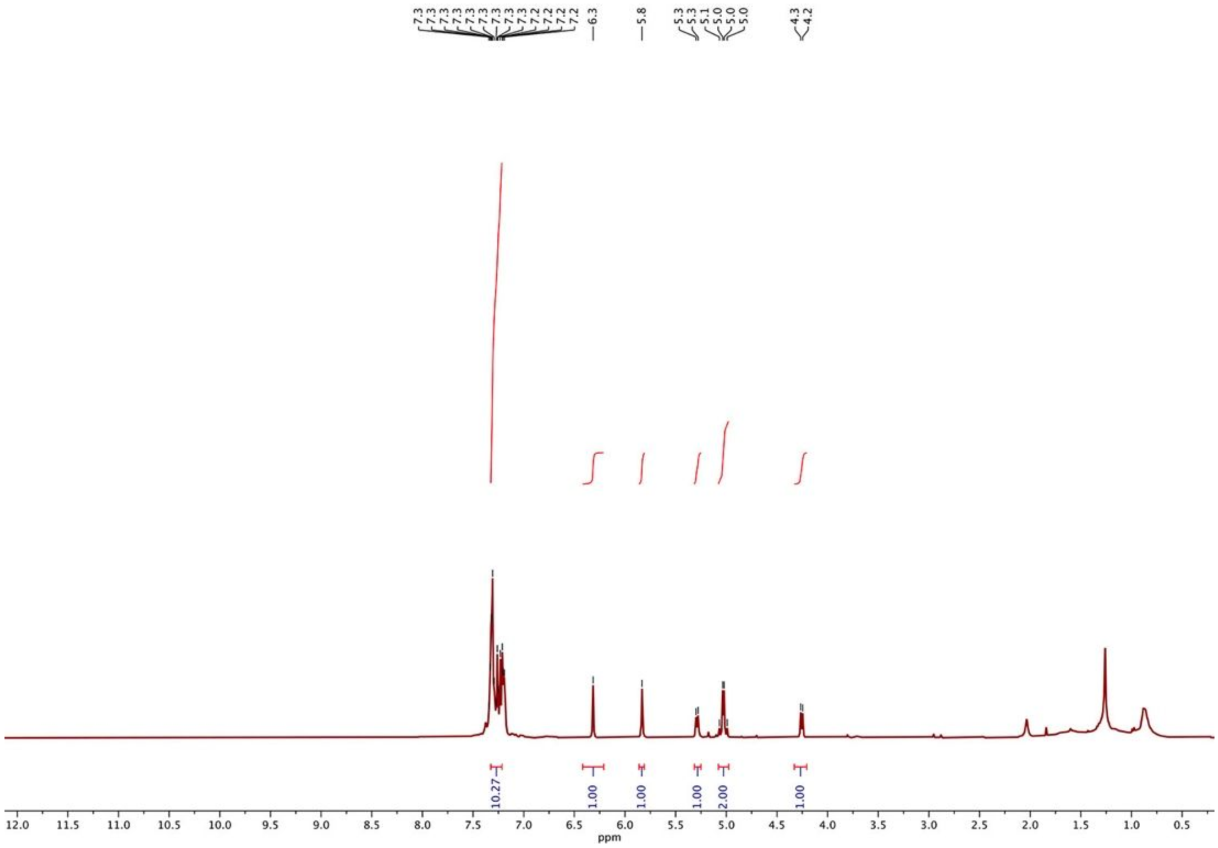

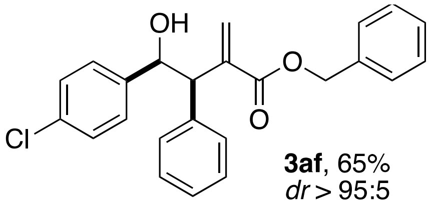

3af, 65%
dr > 95:5

^{13}C{^{1}H} NMR (100 MHz, $CDCl_3$)

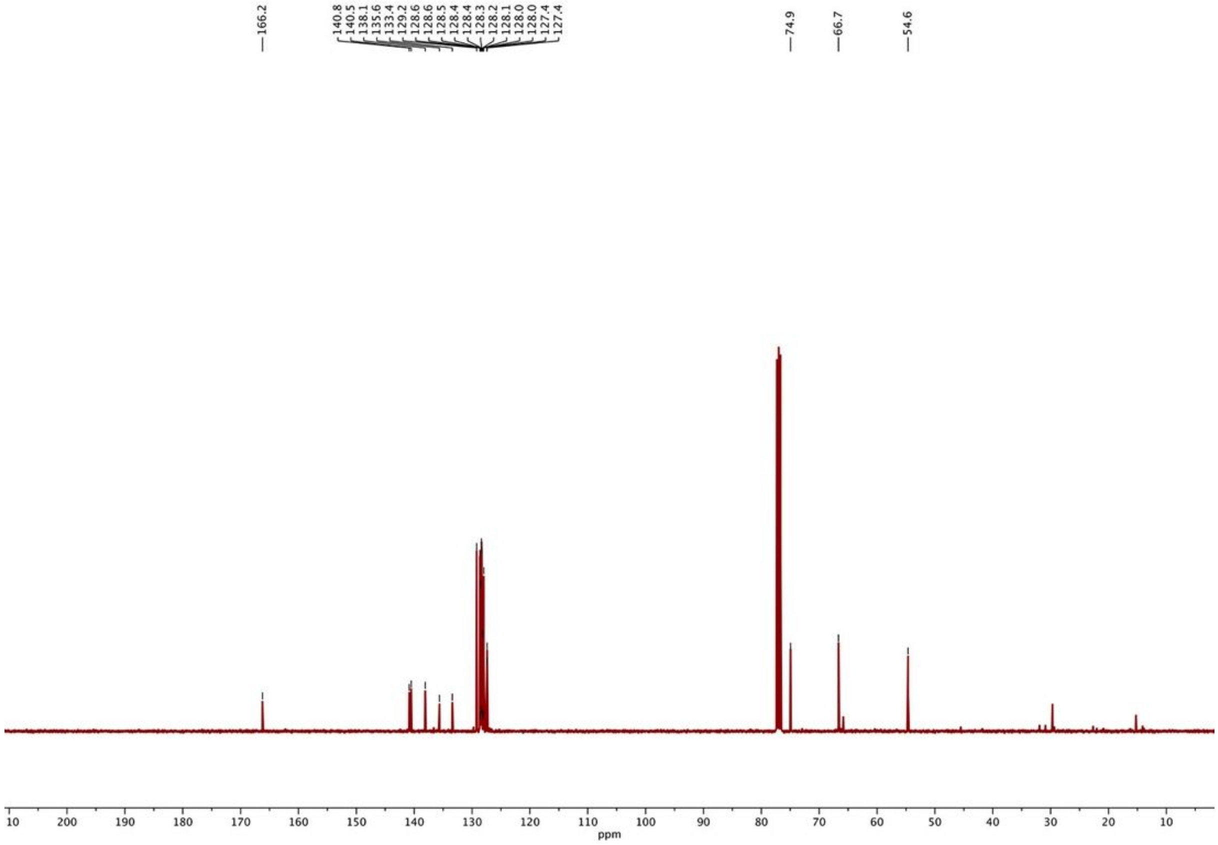

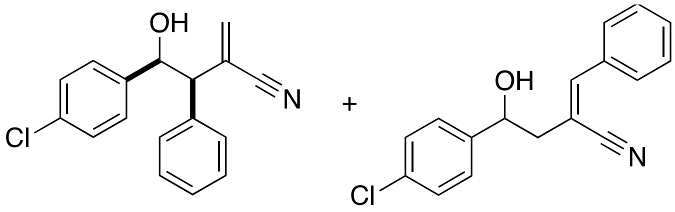

3ah+ 3ah', 67% *dr*= 94:6 *rr* = 65:35

^{1}H NMR (400 MHz, $CDCl_3$)

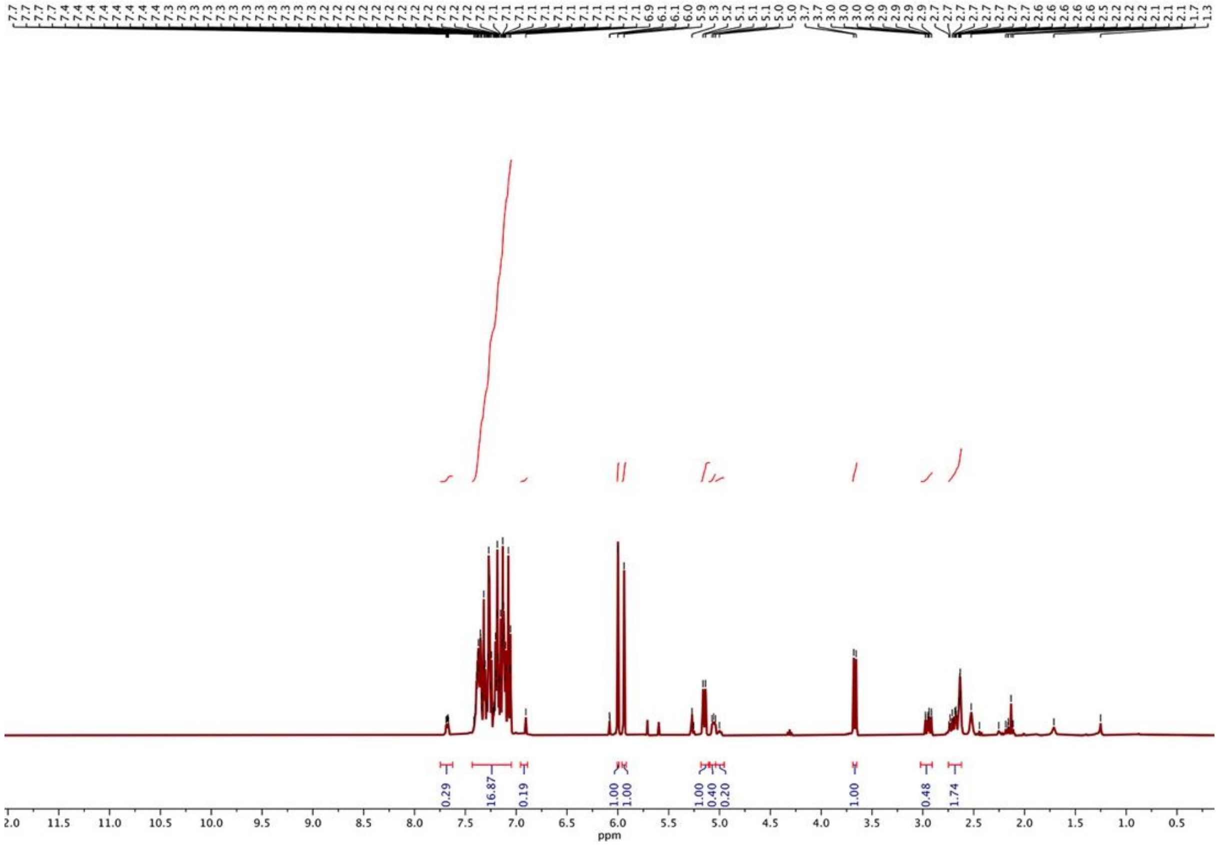

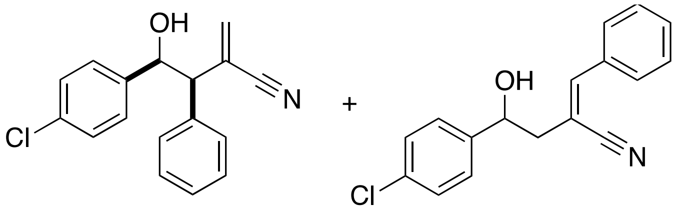

3ah+ 3ah', 67% *dr*= 94:6 *rr* = 65:35

^{13}C{^{1}H} NMR (100 MHz, $CDCl_3$)

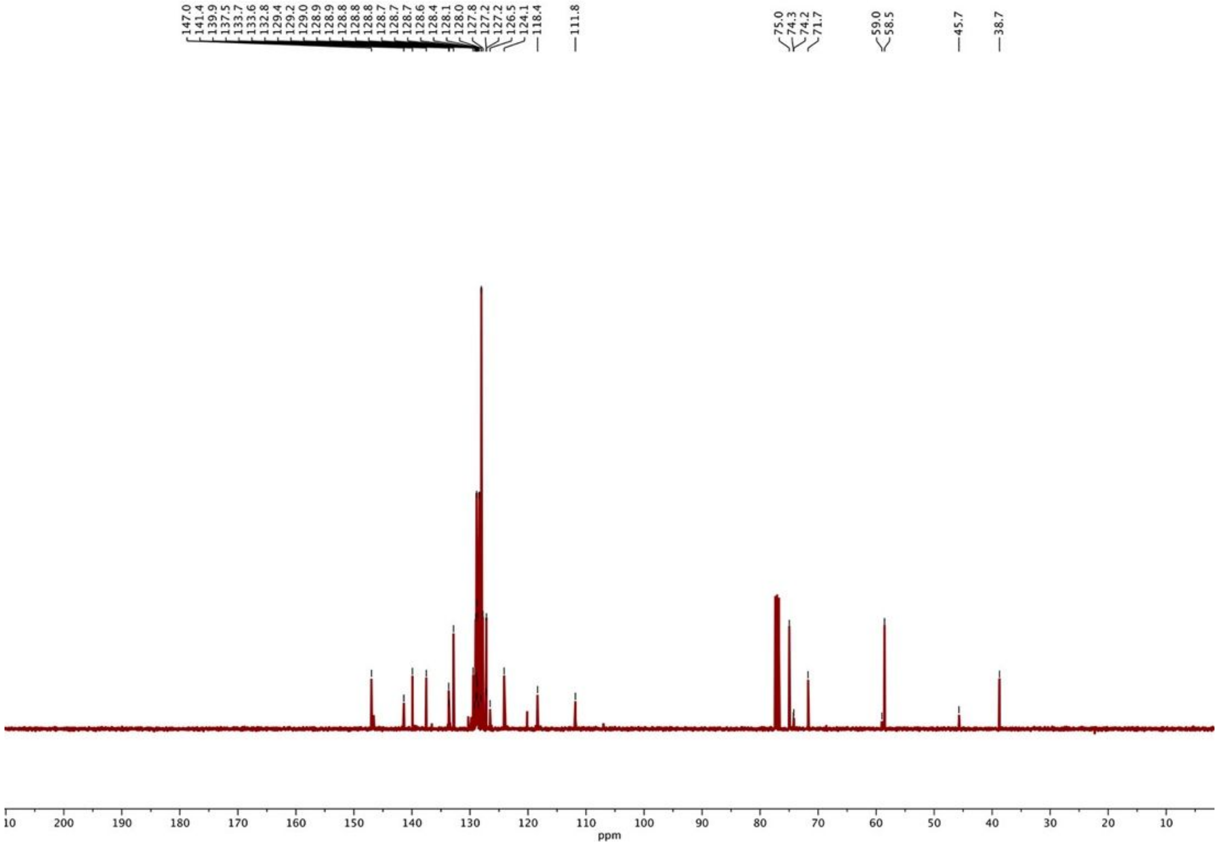

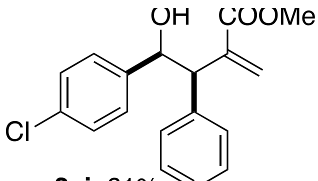

3aj, 61%

^{1}H NMR (400 MHz, $CDCl_3$)

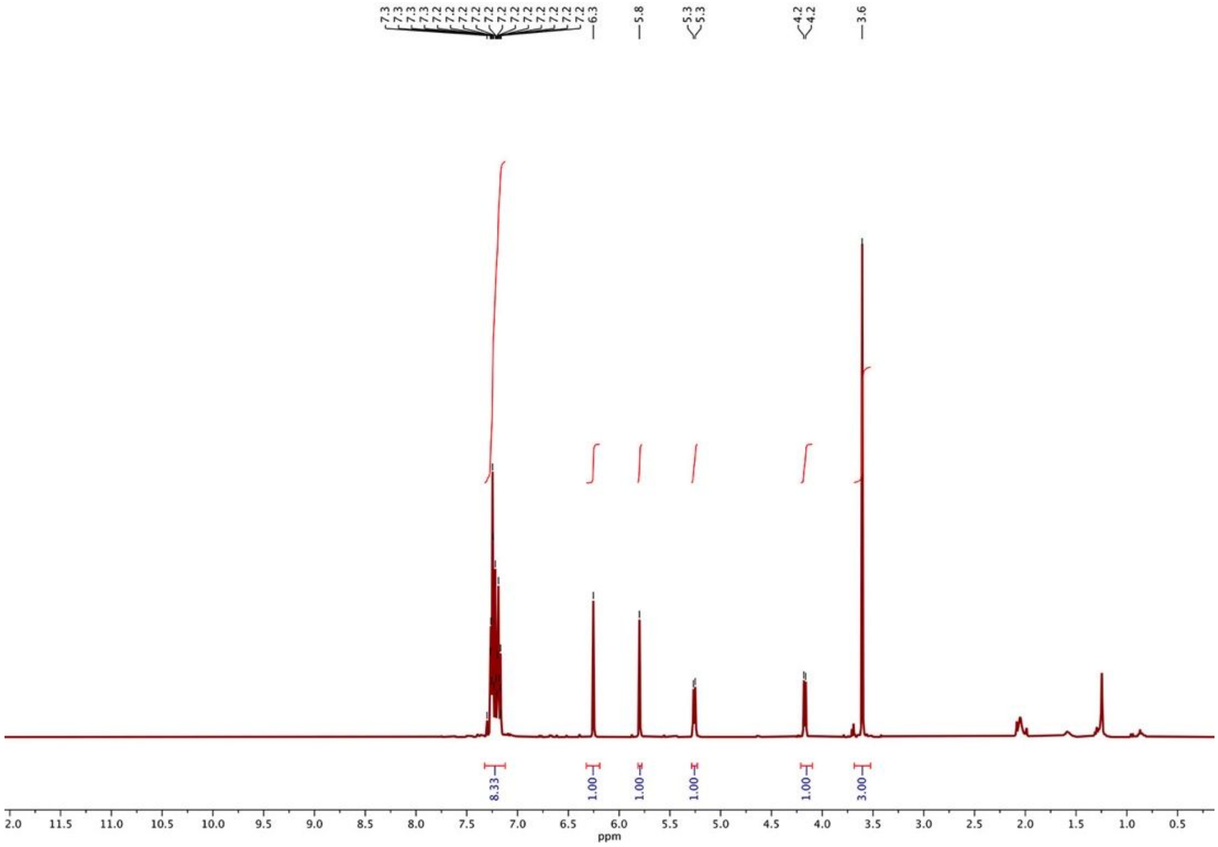

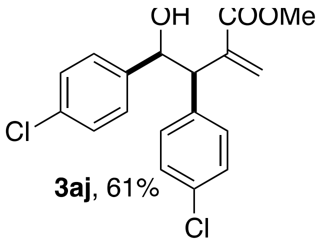

3aj, 61%

$^{13}C\{^1H\}$ NMR (100 MHz, $CDCl_3$)

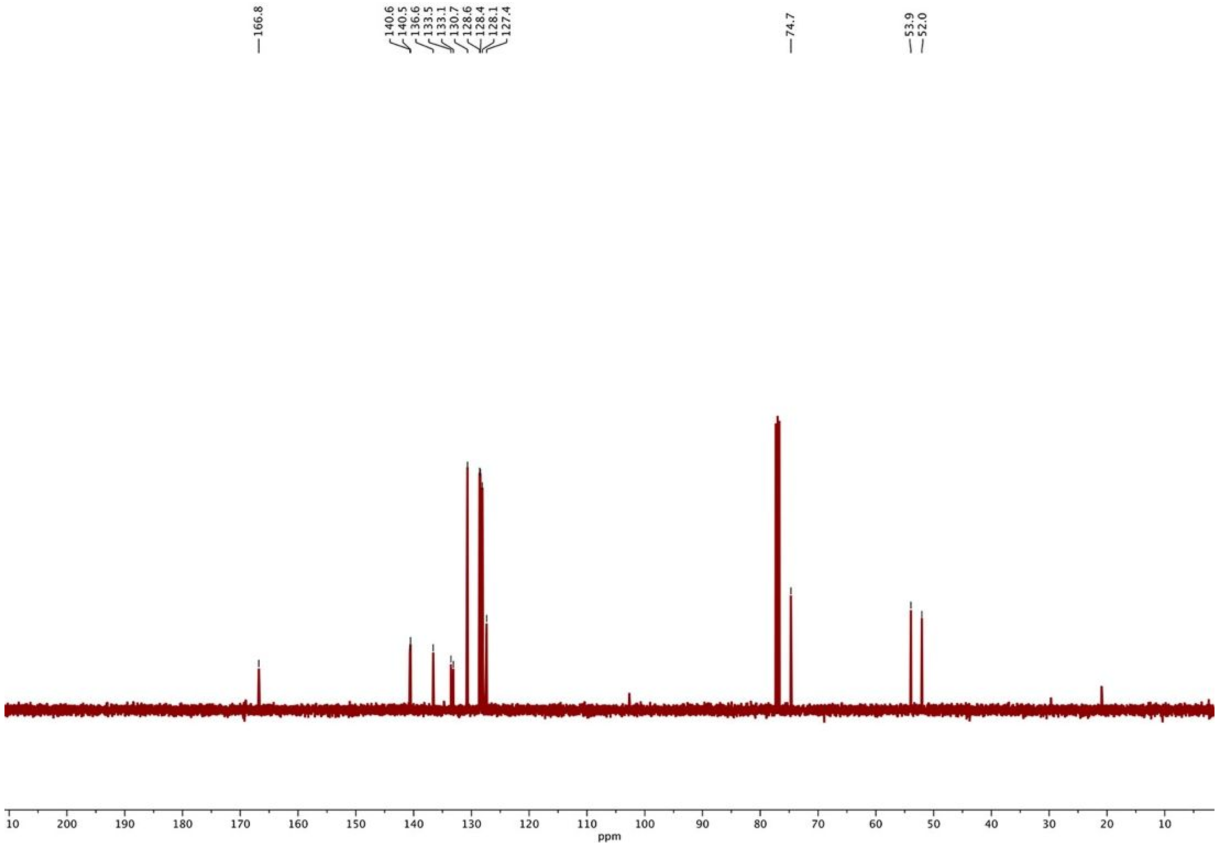

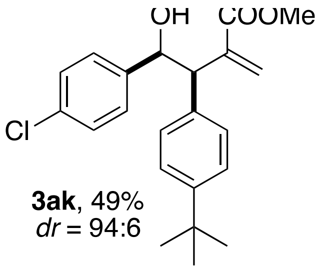

3ak, 49%
dr = 94:6

^{1}H NMR (400 MHz, $CDCl_3$)

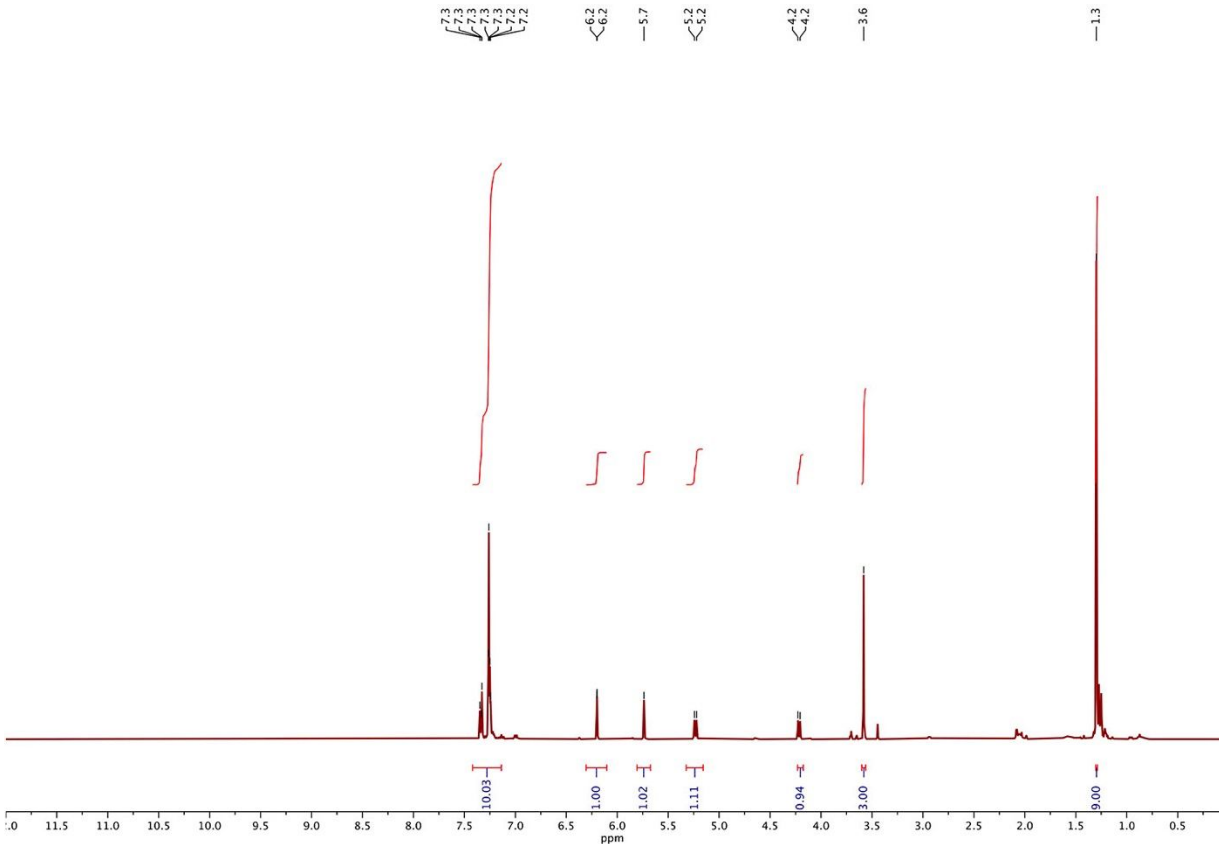

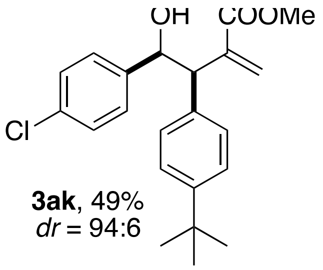

3ak, 49%
dr = 94:6

^{13}C{^{1}H} NMR (100 MHz, $CDCl_3$)

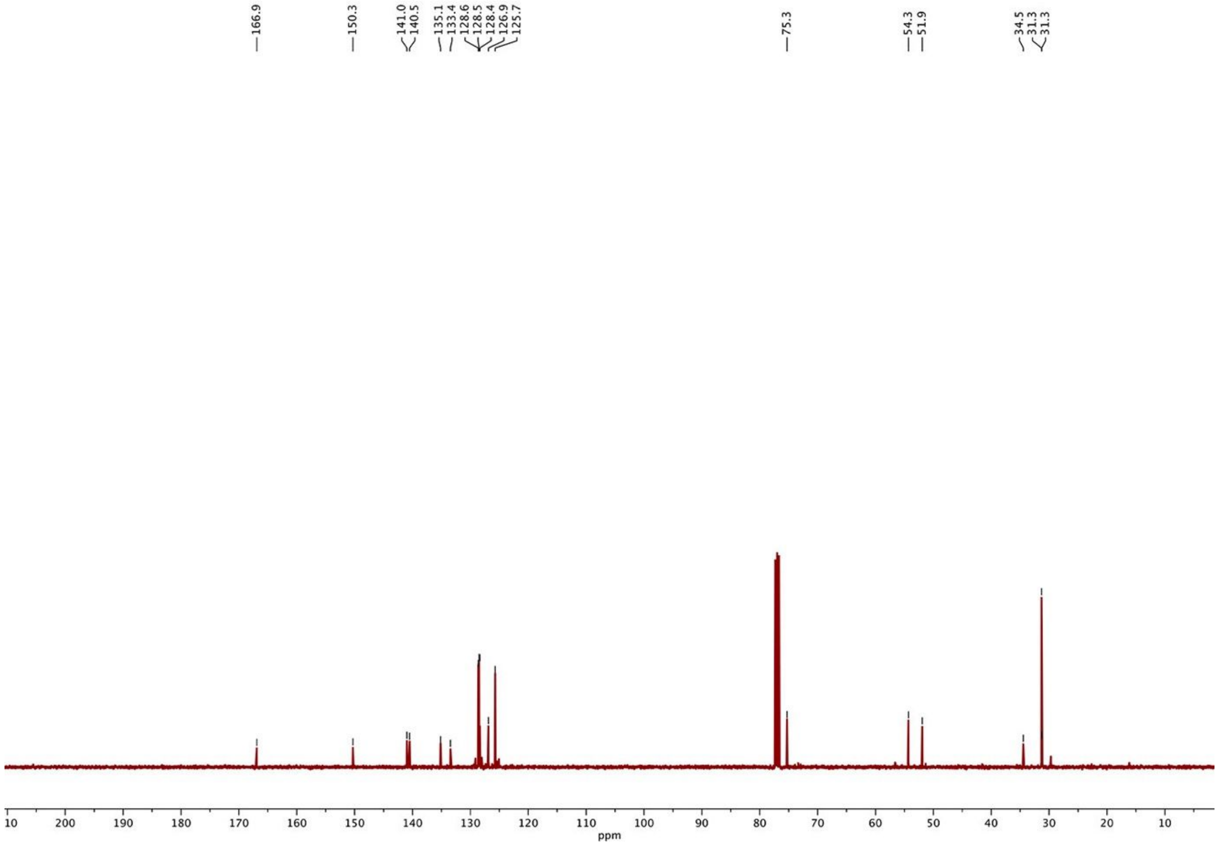

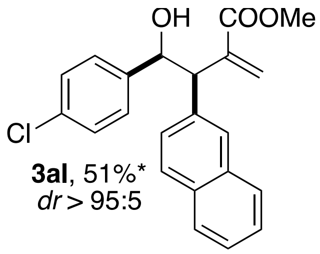

3al, 51%*
dr > 95:5

^{1}H NMR (400 MHz, $CDCl_3$)

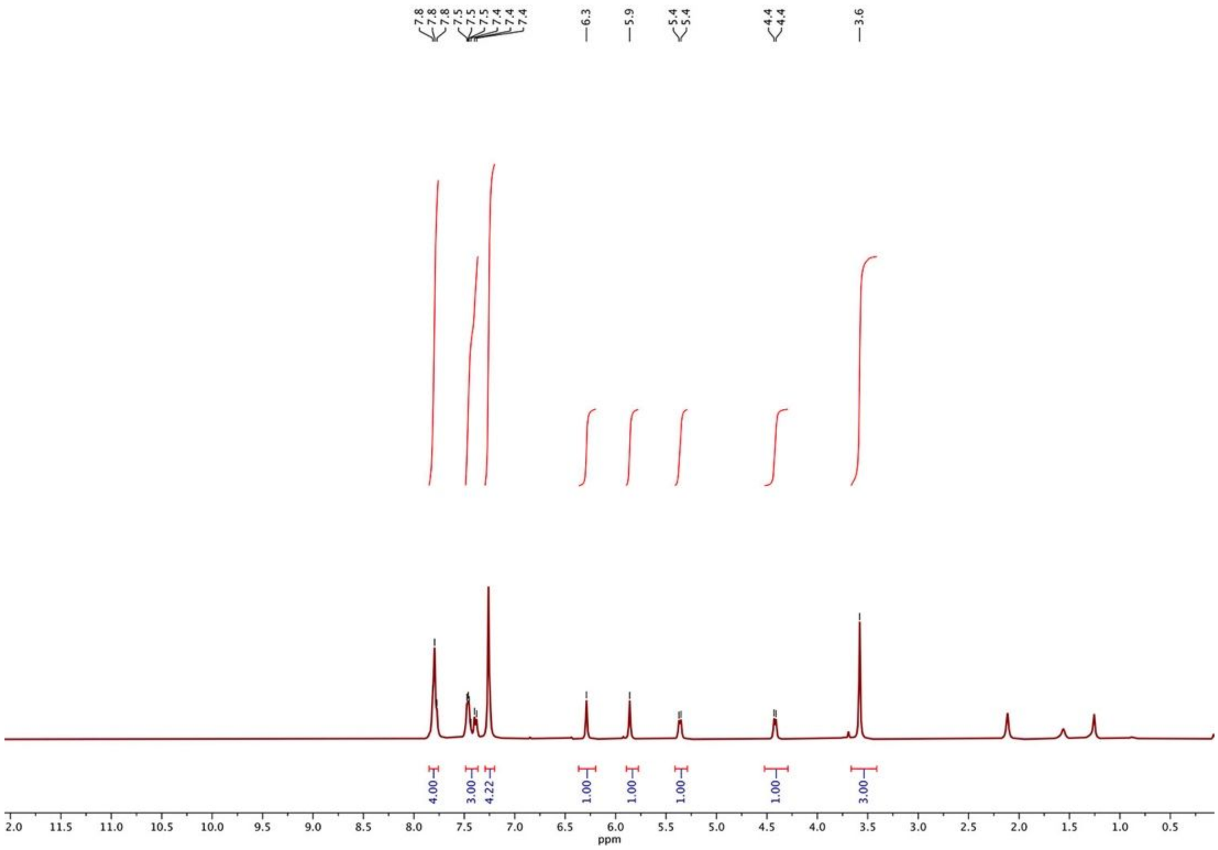

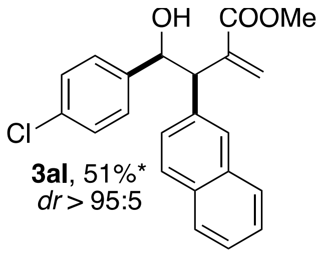

3al, 51%*
dr > 95:5

^{13}C{^{1}H} NMR (100 MHz, $CDCl_3$)

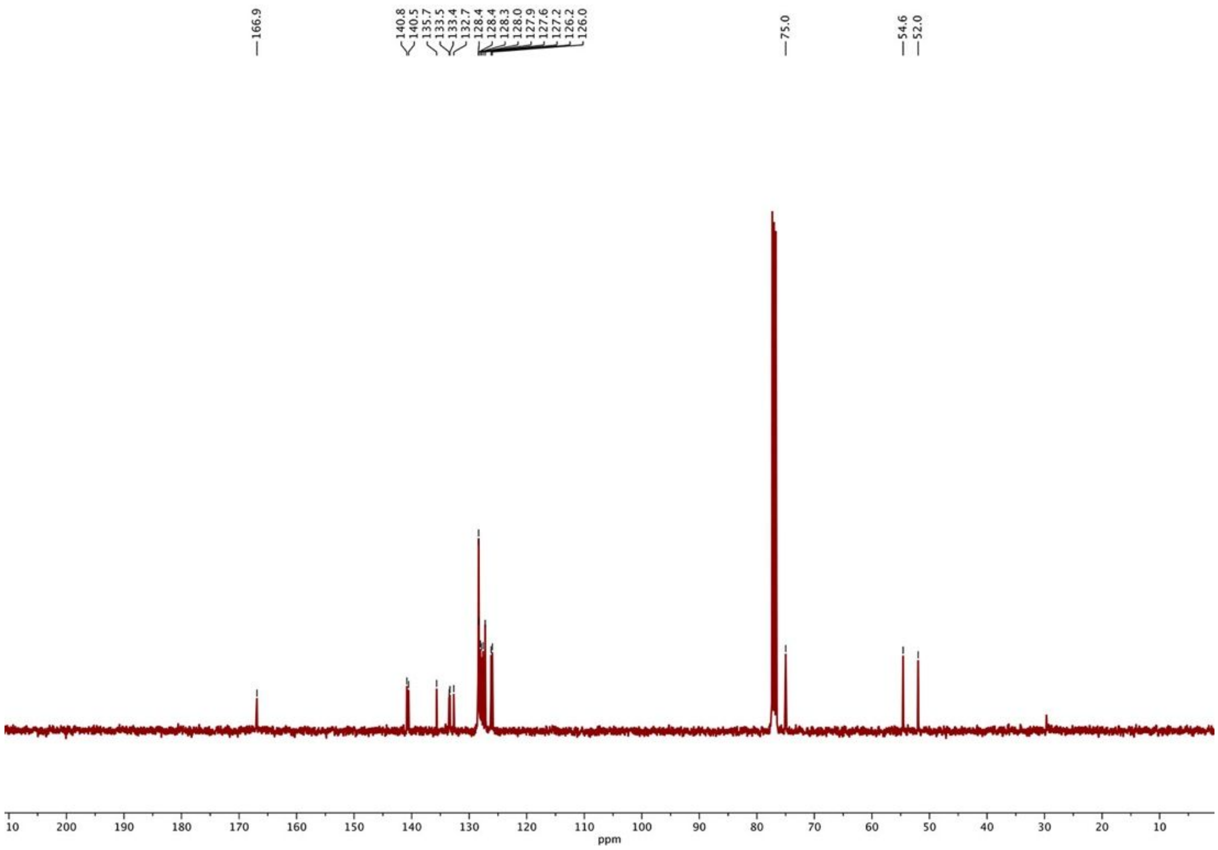

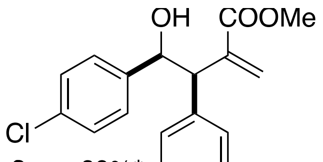

3am, 60%*
dr > 95:5

^{1}H NMR (400 MHz, $CDCl_3$)

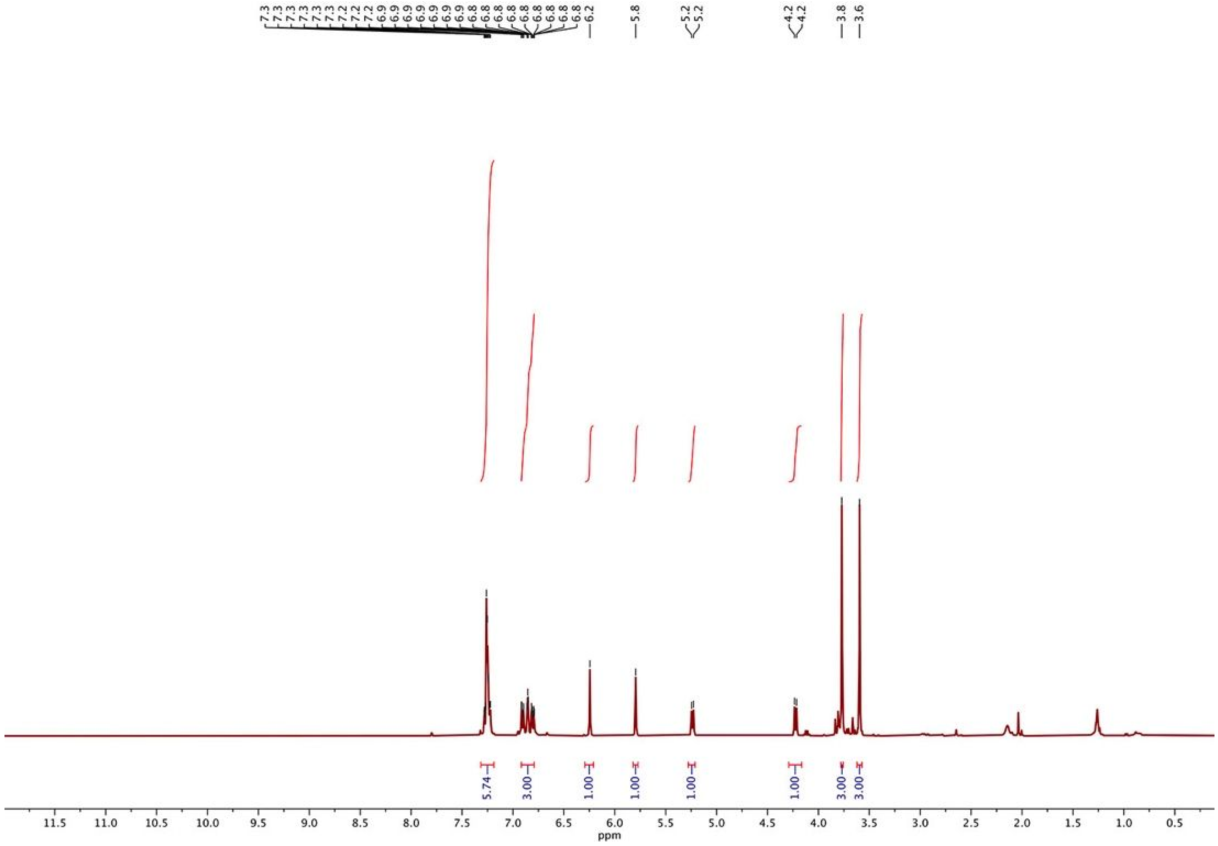

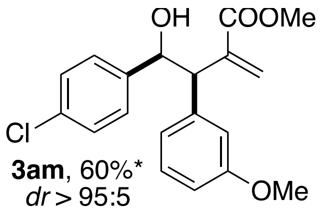

3am, 60%*
dr > 95:5

^{13}C{^{1}H} NMR (100 MHz, $CDCl_3$)

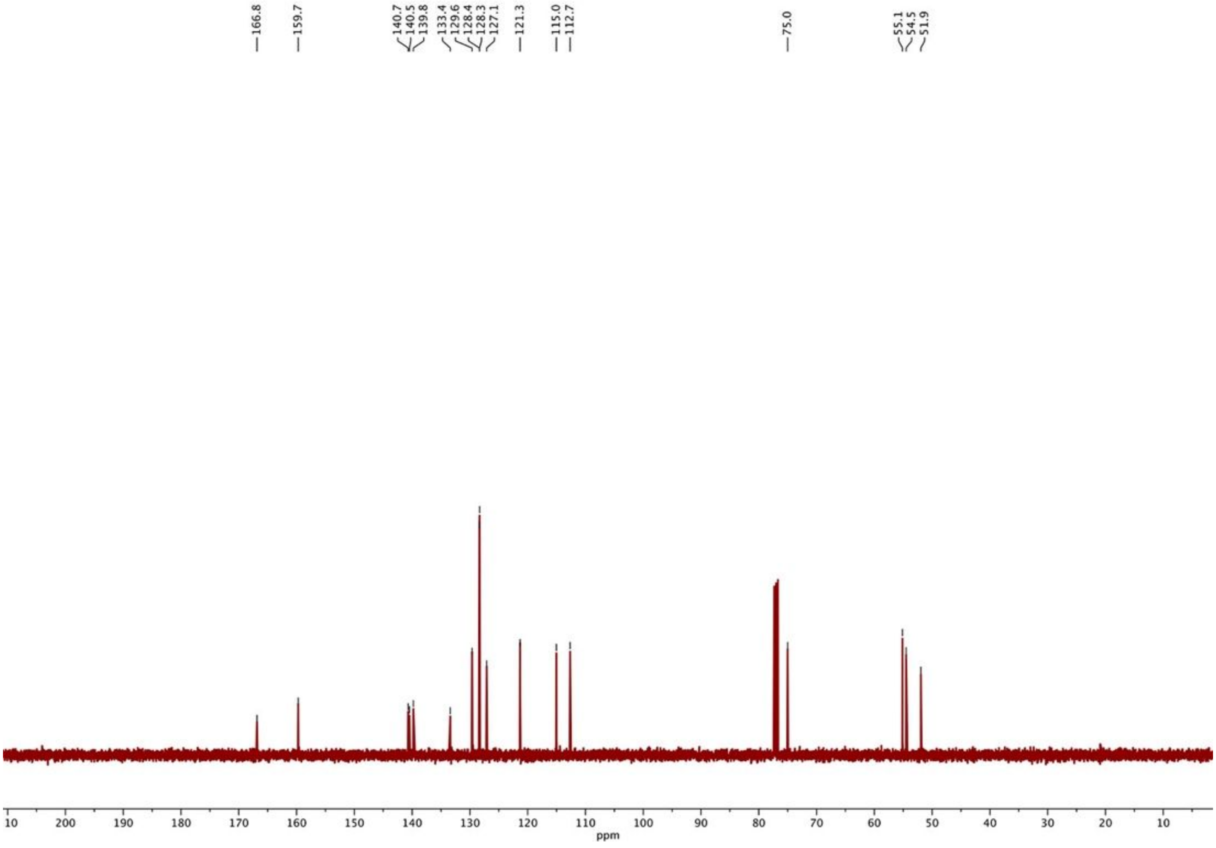

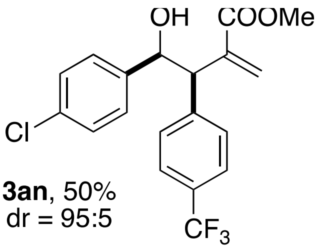

3an, 50%
dr = 95:5

^{1}H NMR (400 MHz, $CDCl_3$)

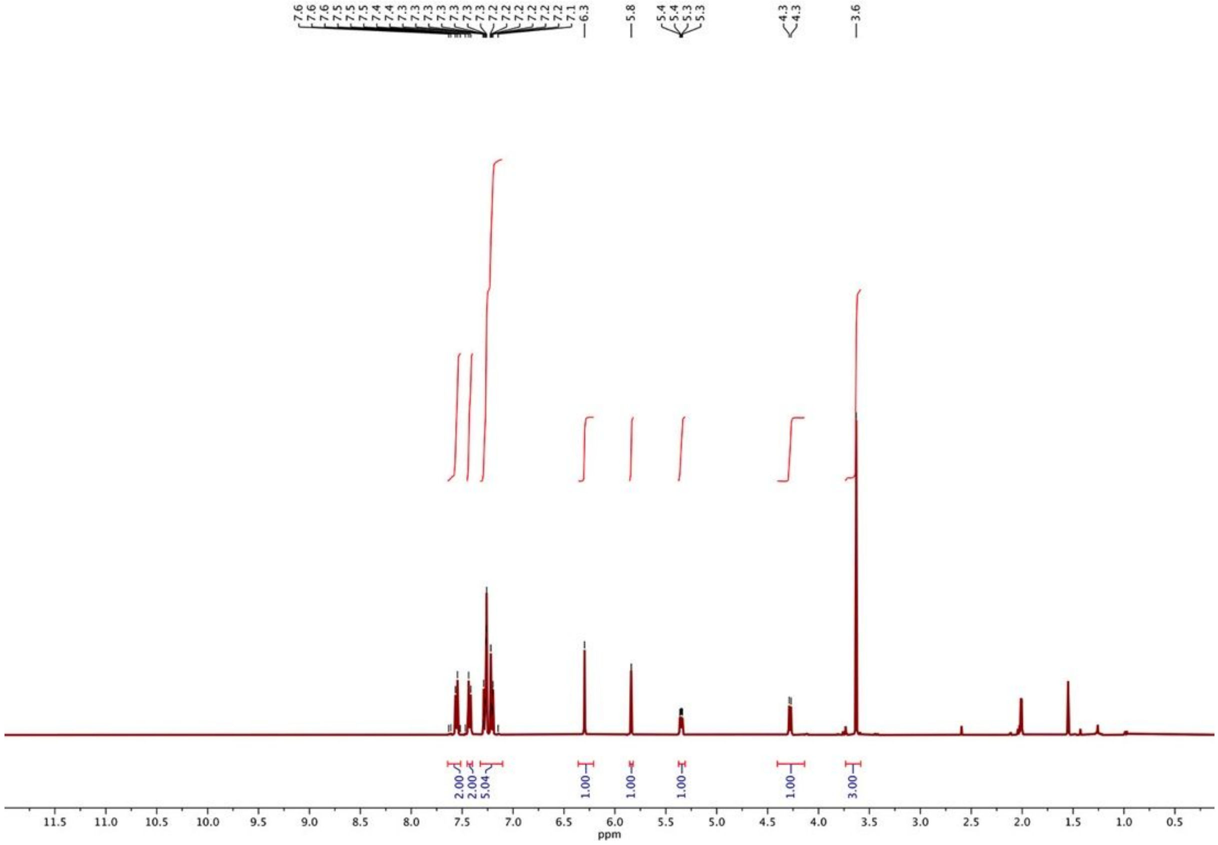

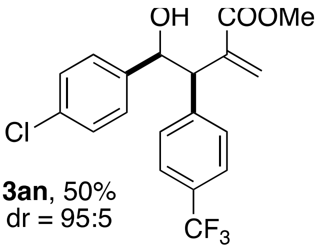

3an, 50%
dr = 95:5

^{13}C{^{1}H} NMR (100 MHz, CDCl$_3$)

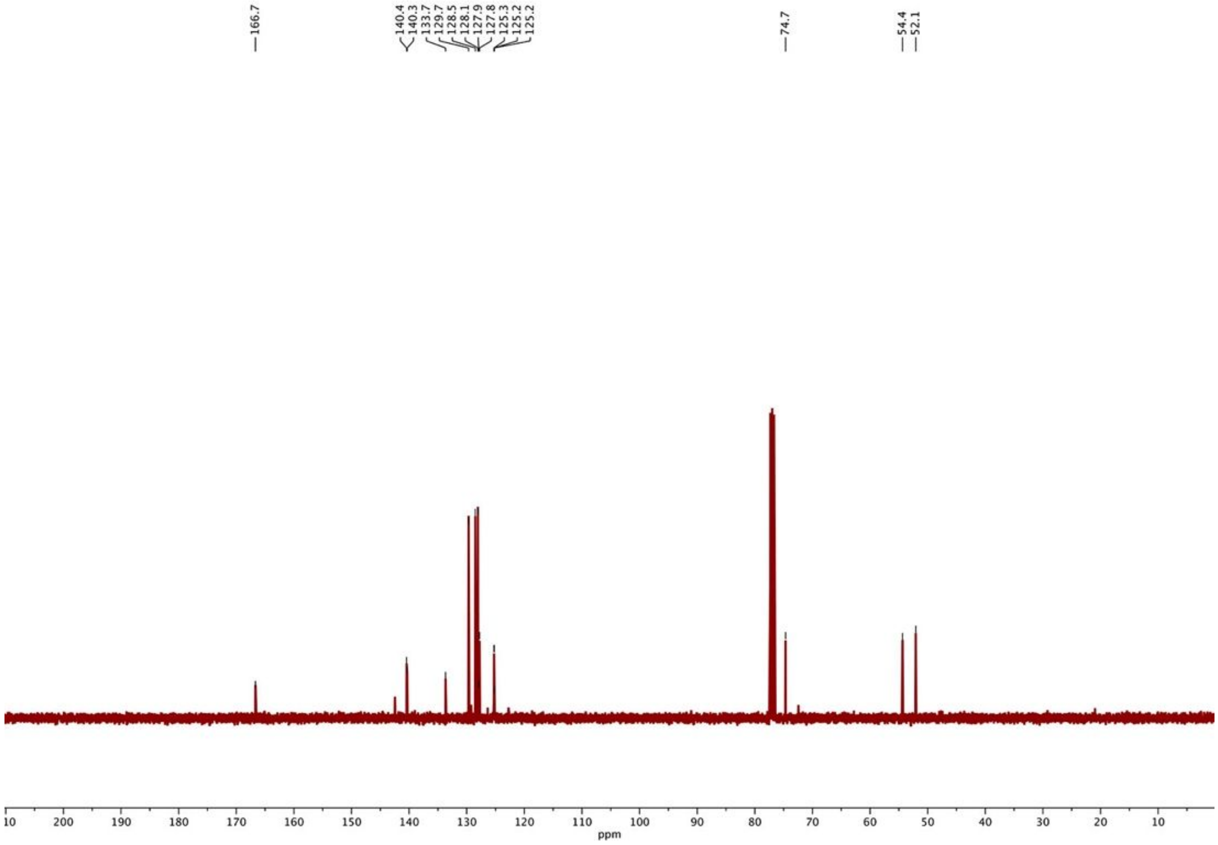

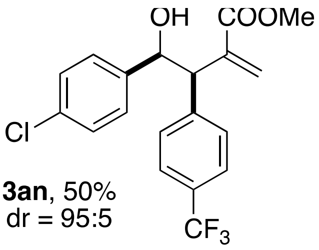

3an, 50%
dr = 95:5

^{19}**F NMR** (377 MHz, $CDCl_3$)

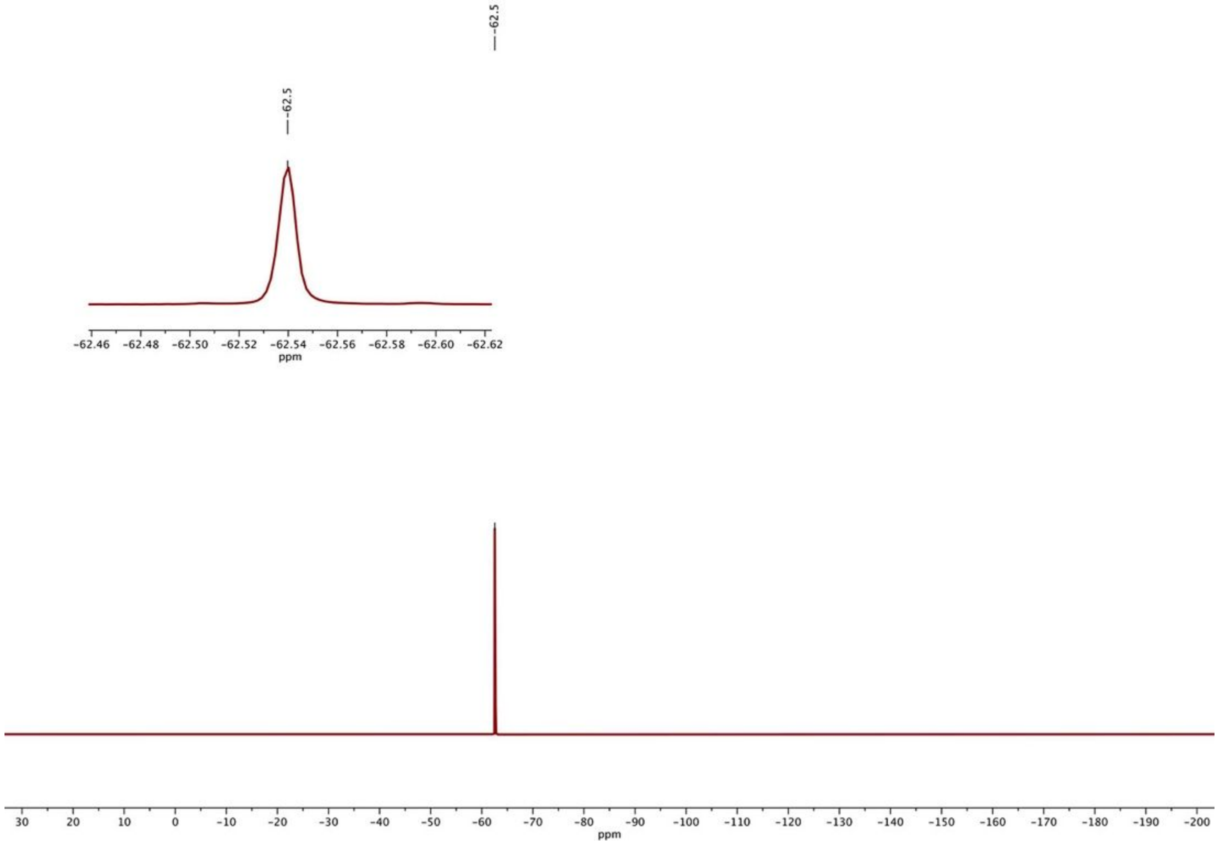

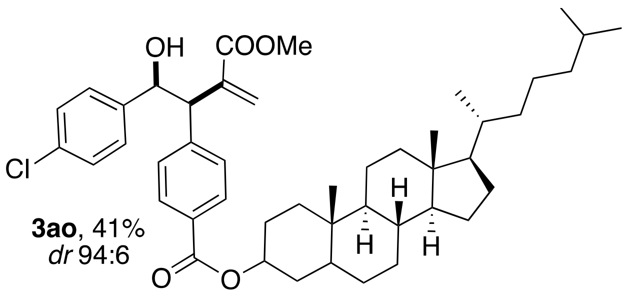

3ao, 41%
dr 94:6

^{1}H NMR (400 MHz, $CDCl_3$)

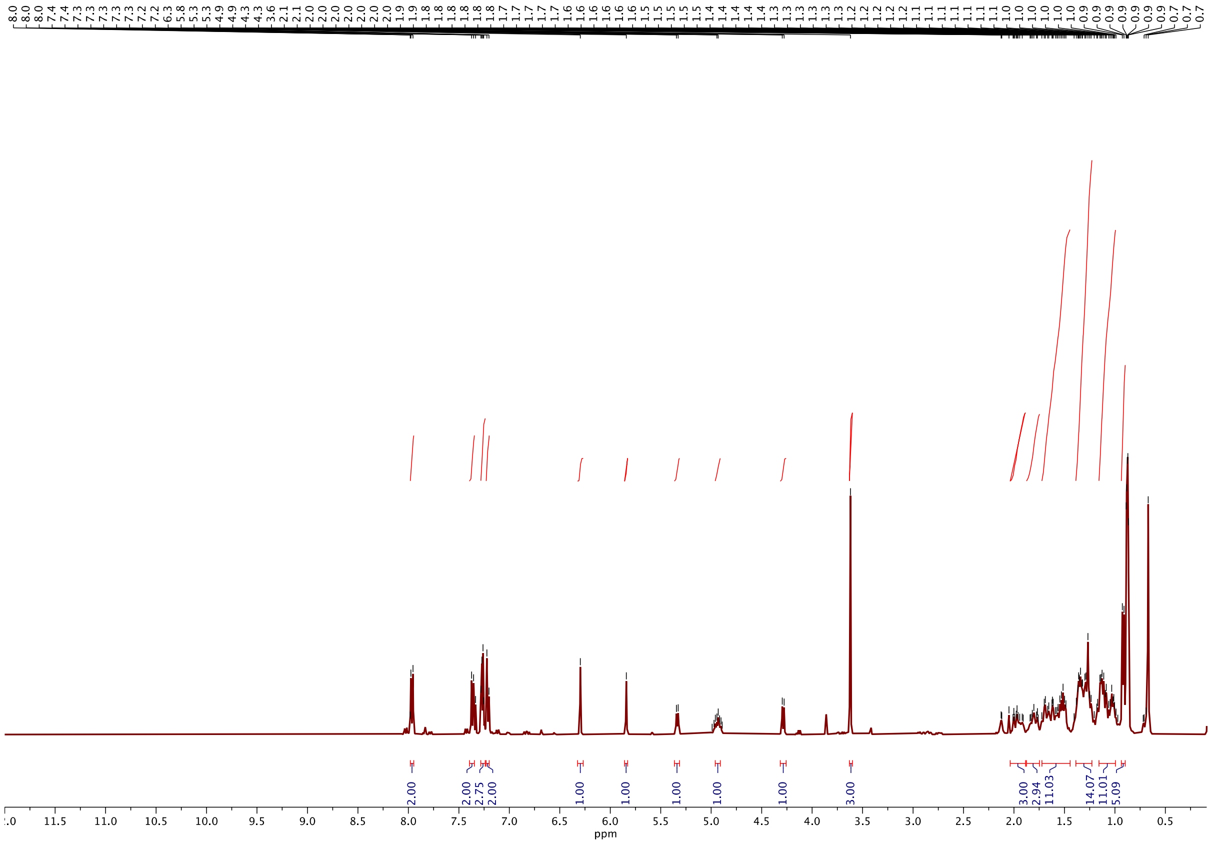

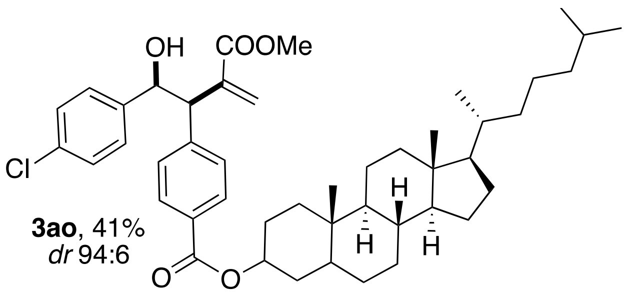

3ao, 41%
dr 94:6

^{13}C{^{1}H} NMR (100 MHz, $CDCl_3$)

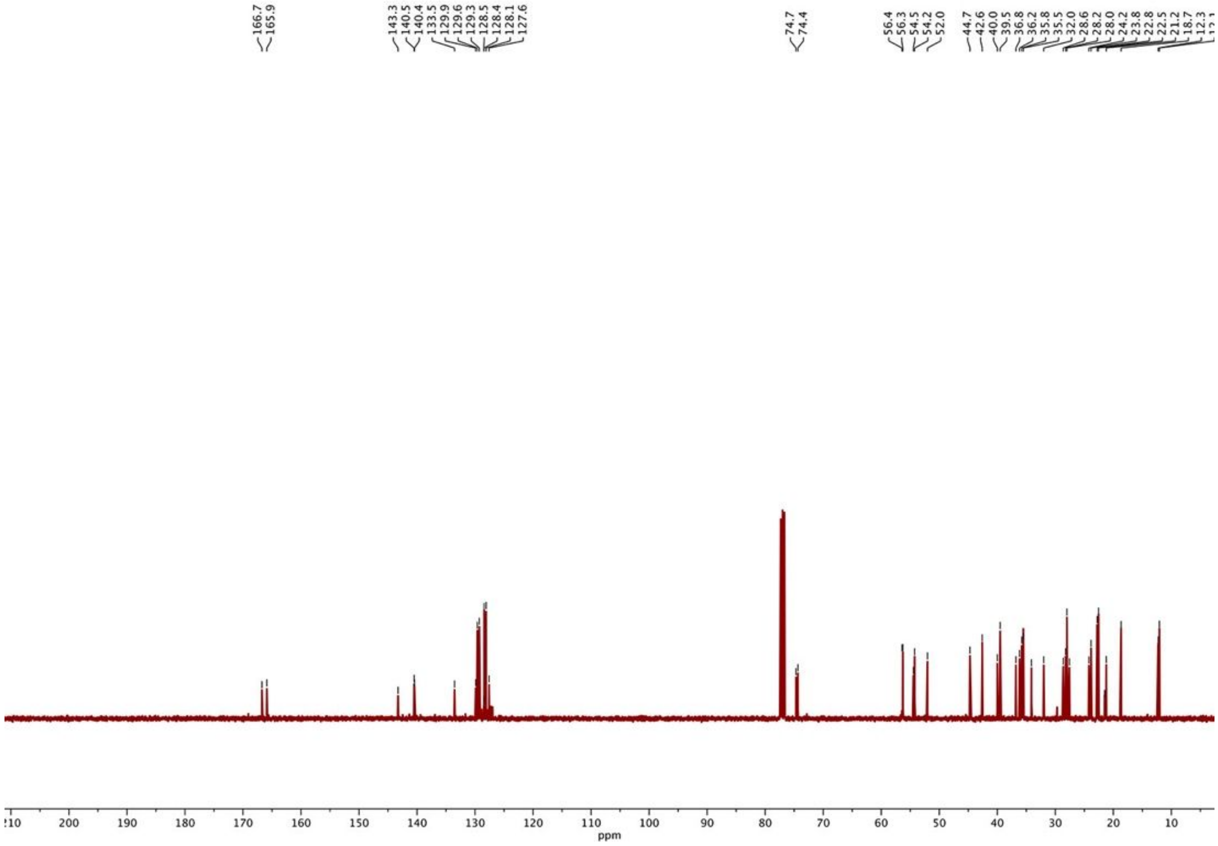

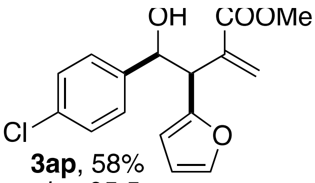

3ap, 58%
dr > 95:5

^{1}H NMR (400 MHz, $CDCl_3$)

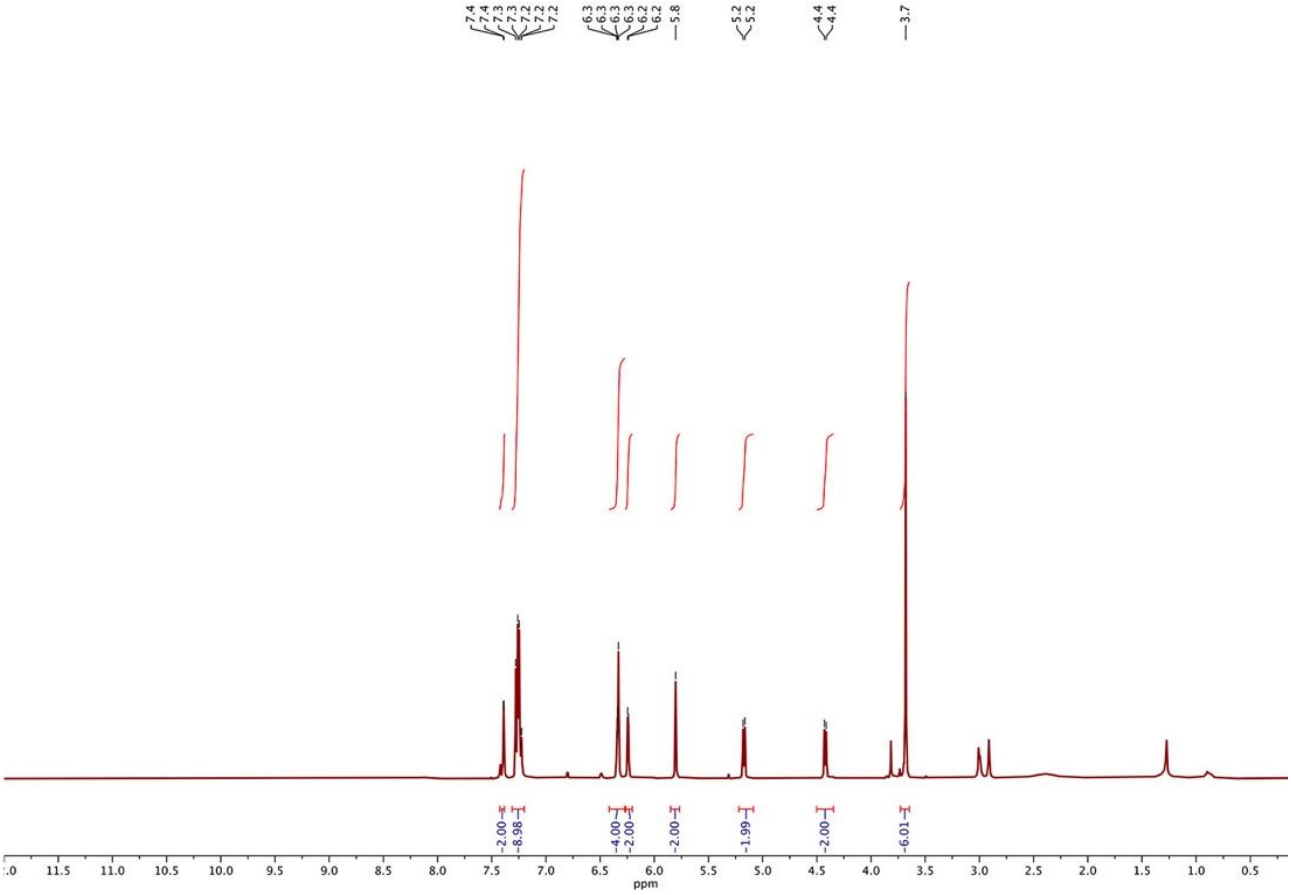

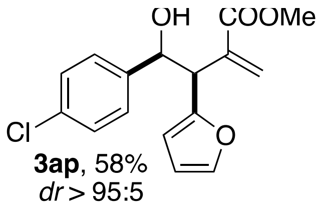

3ap, 58%
dr > 95:5

^{13}C{^{1}H} NMR (100 MHz, $CDCl_3$)

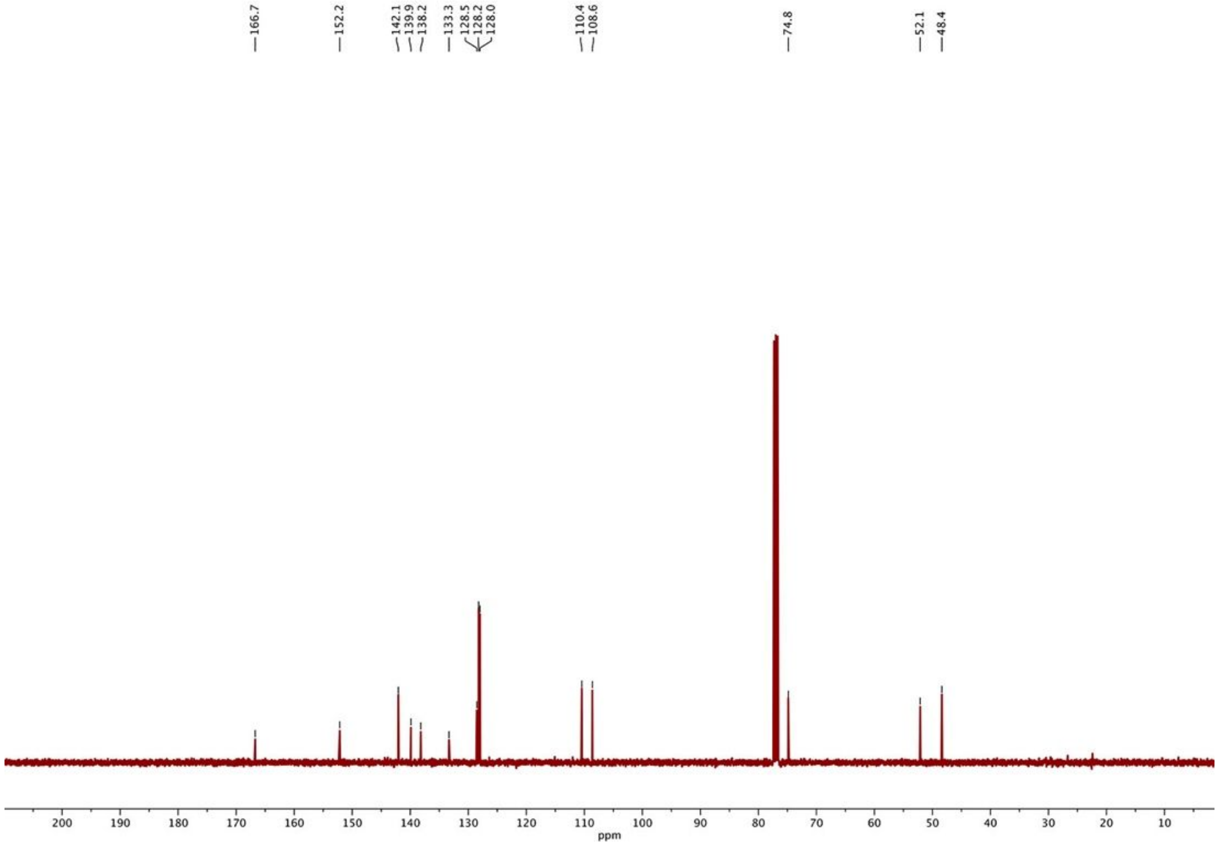

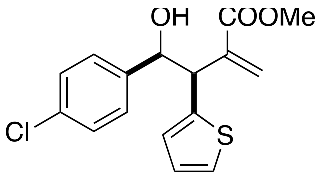

3aq, 77%*
dr > 95:5

^{1}H NMR (400 MHz, $CDCl_3$)

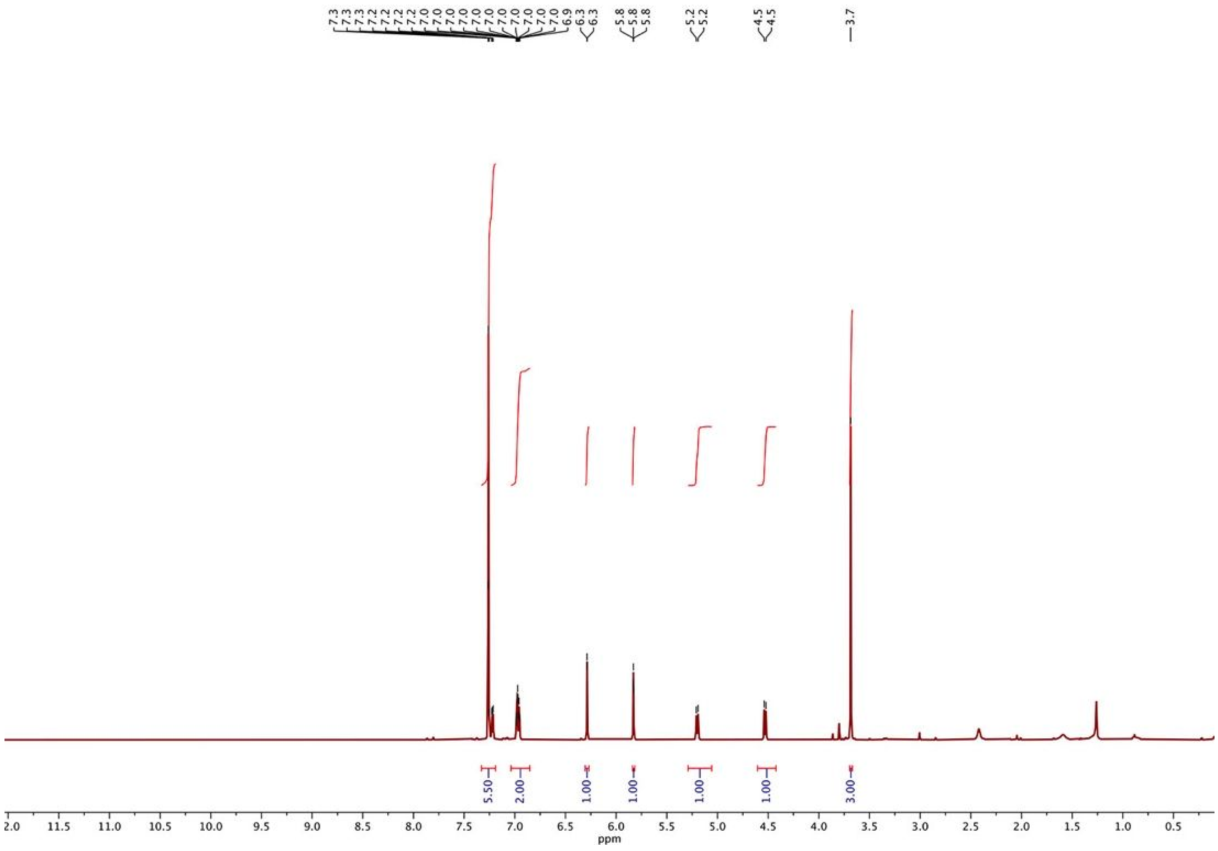

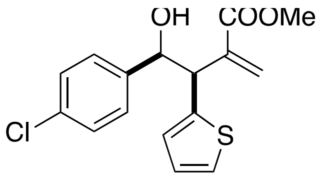

3aq, 77%*
dr > 95:5

^{13}C{^{1}H} NMR (100 MHz, $CDCl_3$)

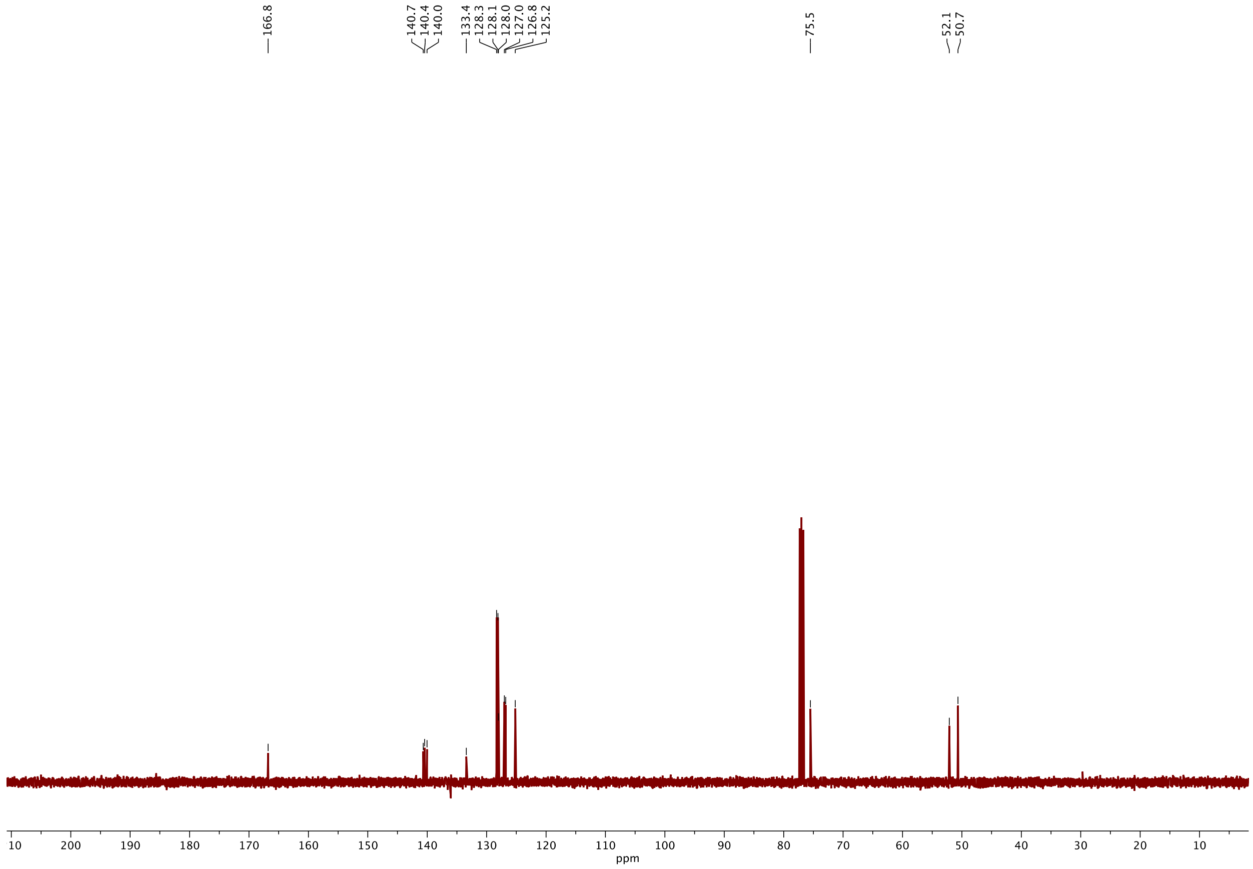

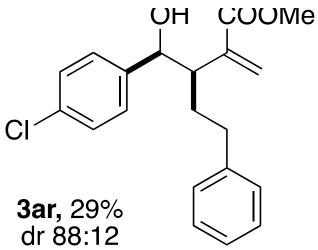

3ar, 29%
dr 88:12

[1]**H NMR** (400 MHz, $CDCl_3$)

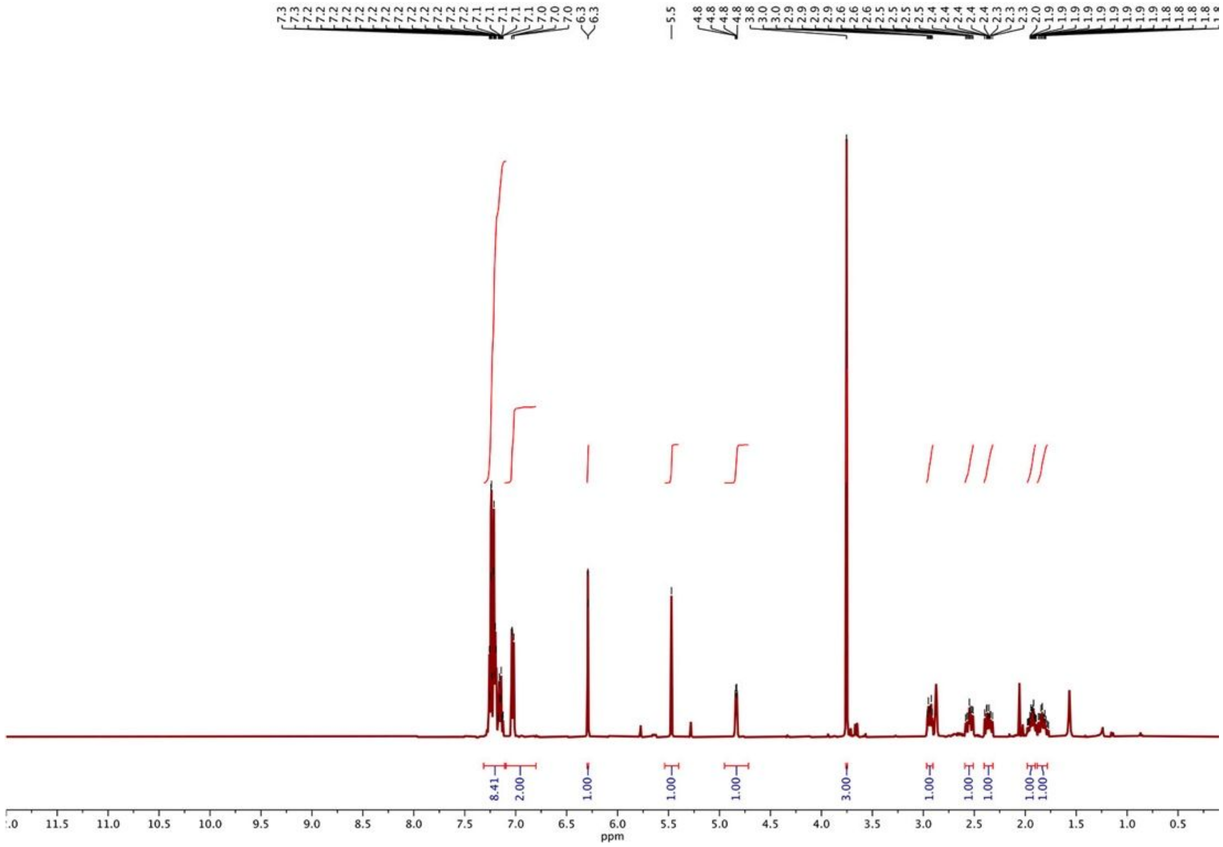

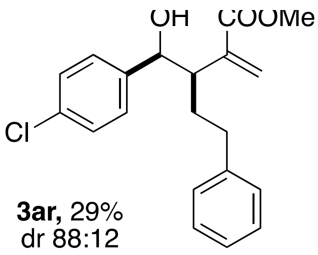

3ar, 29%
dr 88:12

^{13}C{^{1}H} NMR (100 MHz, $CDCl_3$)

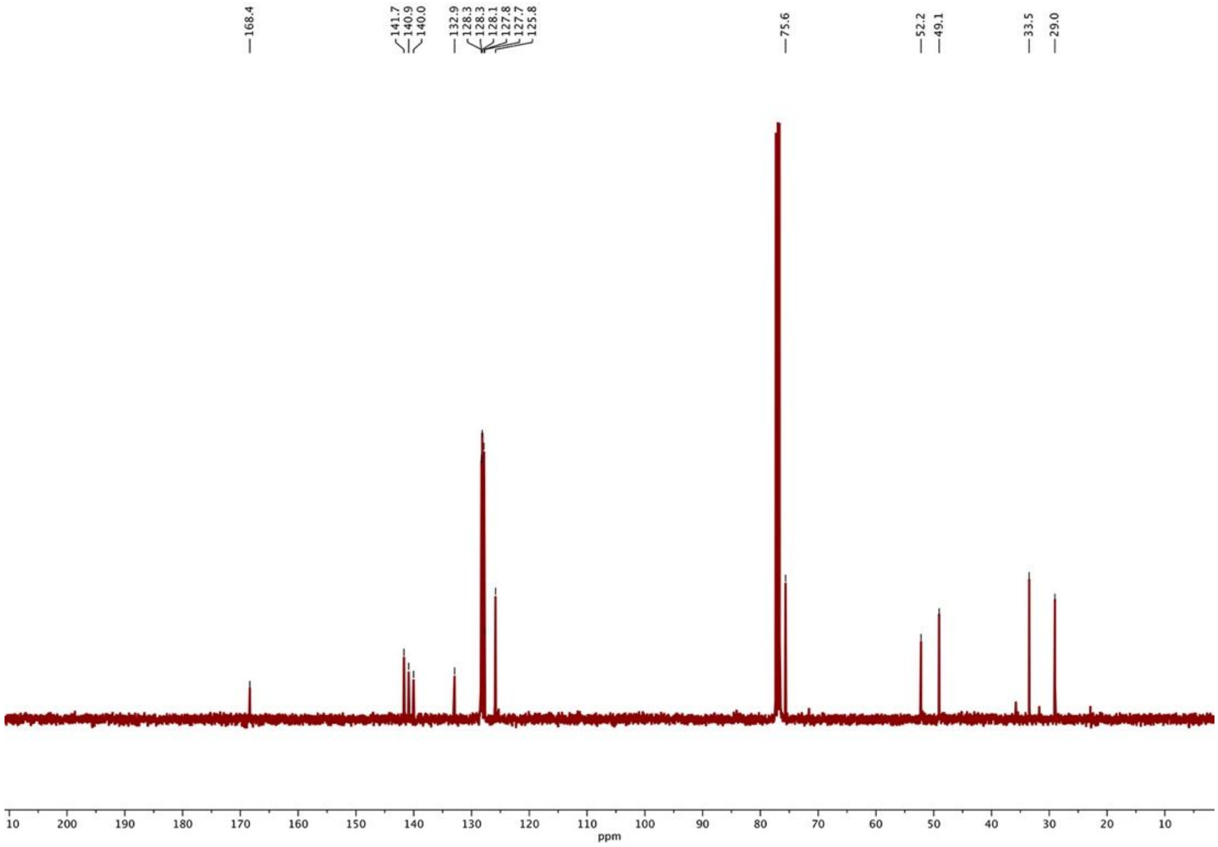

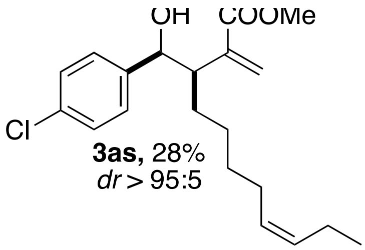

3as, 28%
dr > 95:5

^{1}H NMR (400 MHz, $CDCl_3$)

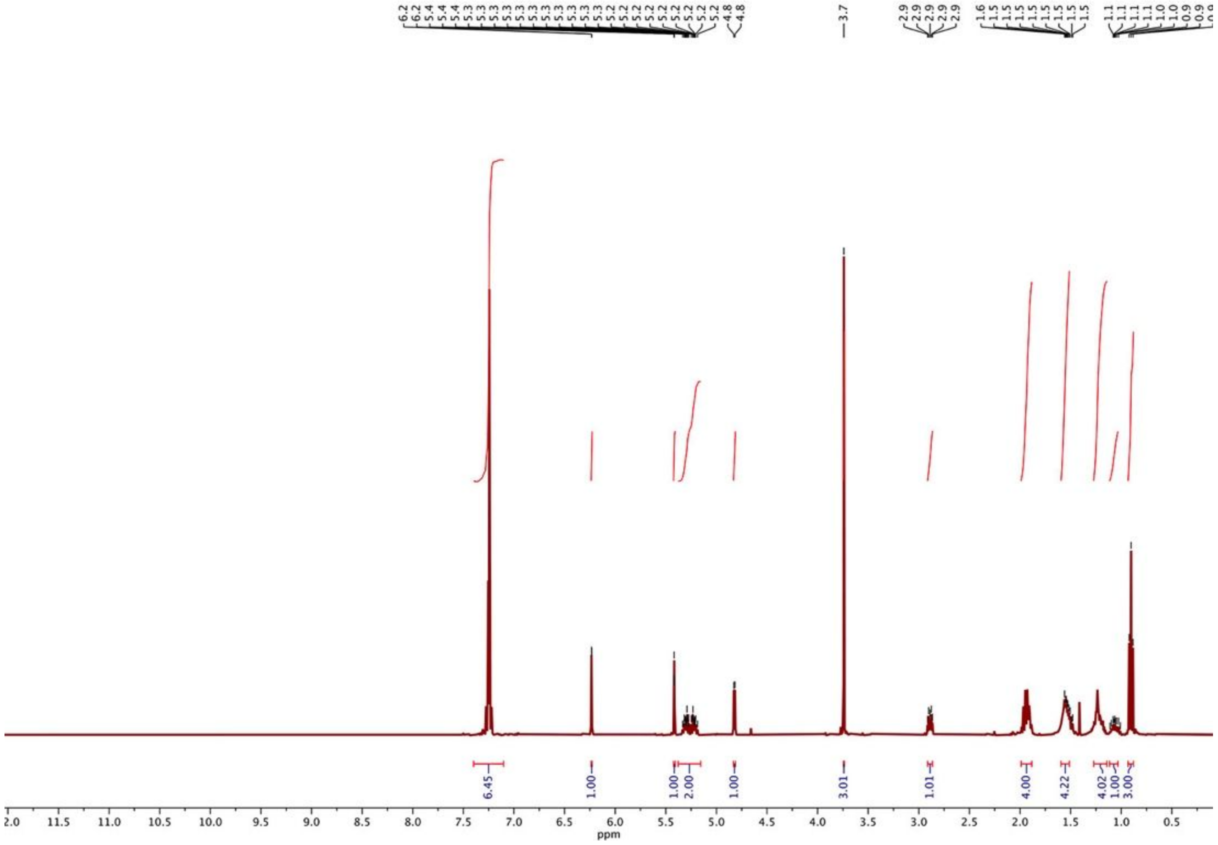

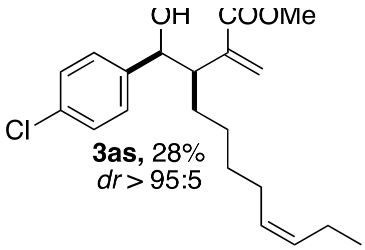

3as, 28%
dr > 95:5

$^{13}C\{^{1}H\}$ NMR (100 MHz, $CDCl_3$)

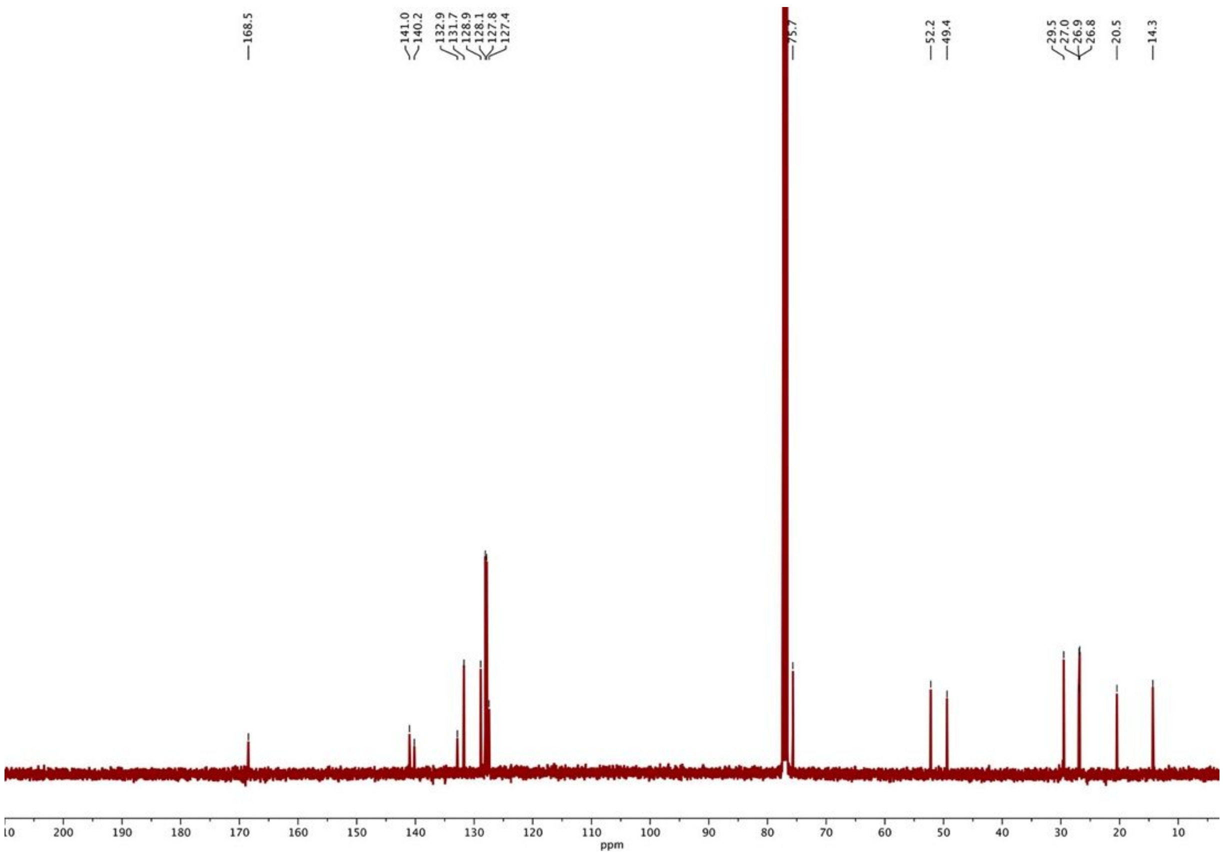

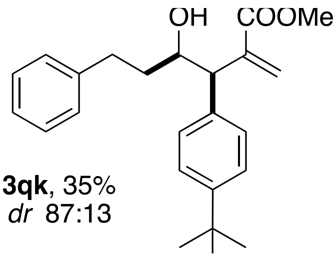

3qk, 35%
dr 87:13

^{1}**H NMR** (400 MHz, $CDCl_3$)

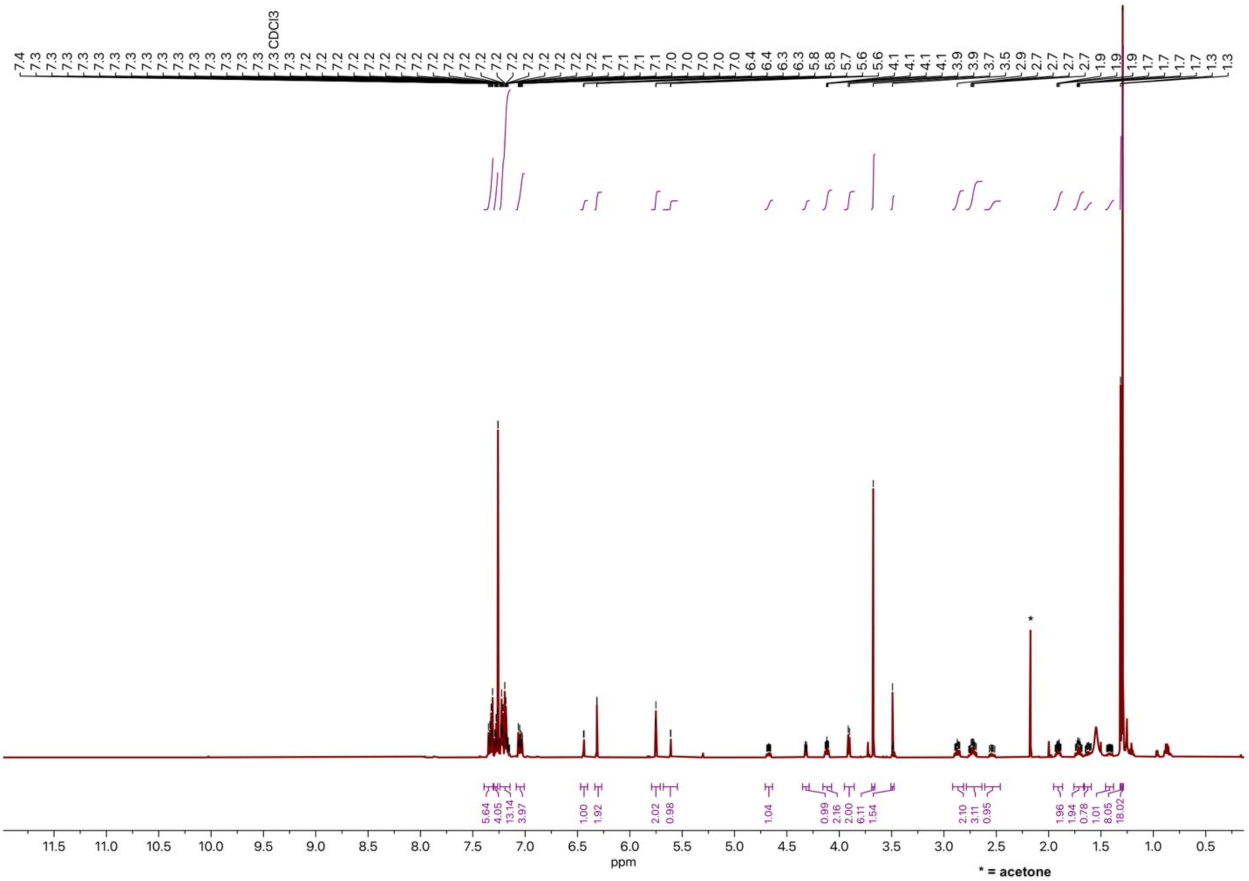

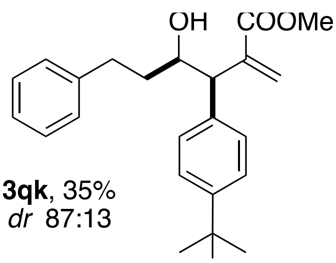

3qk, 35%
dr 87:13

^{13}C{^{1}H} NMR (100 MHz, $CDCl_3$)

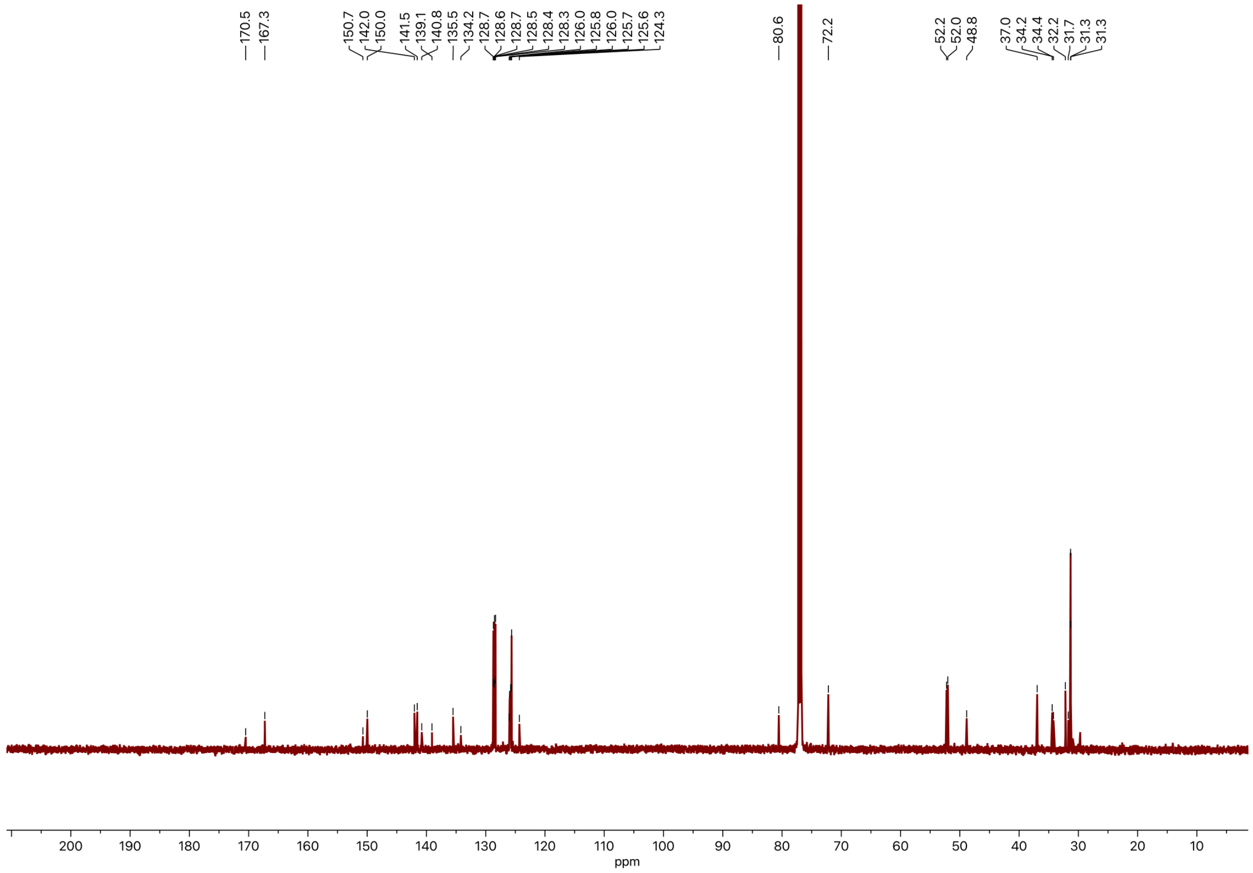

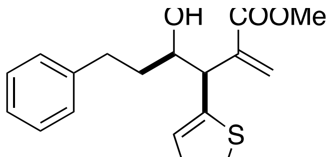

3qq, 24%
dr > 95:5

^{1}H NMR (400 MHz, $CDCl_3$)

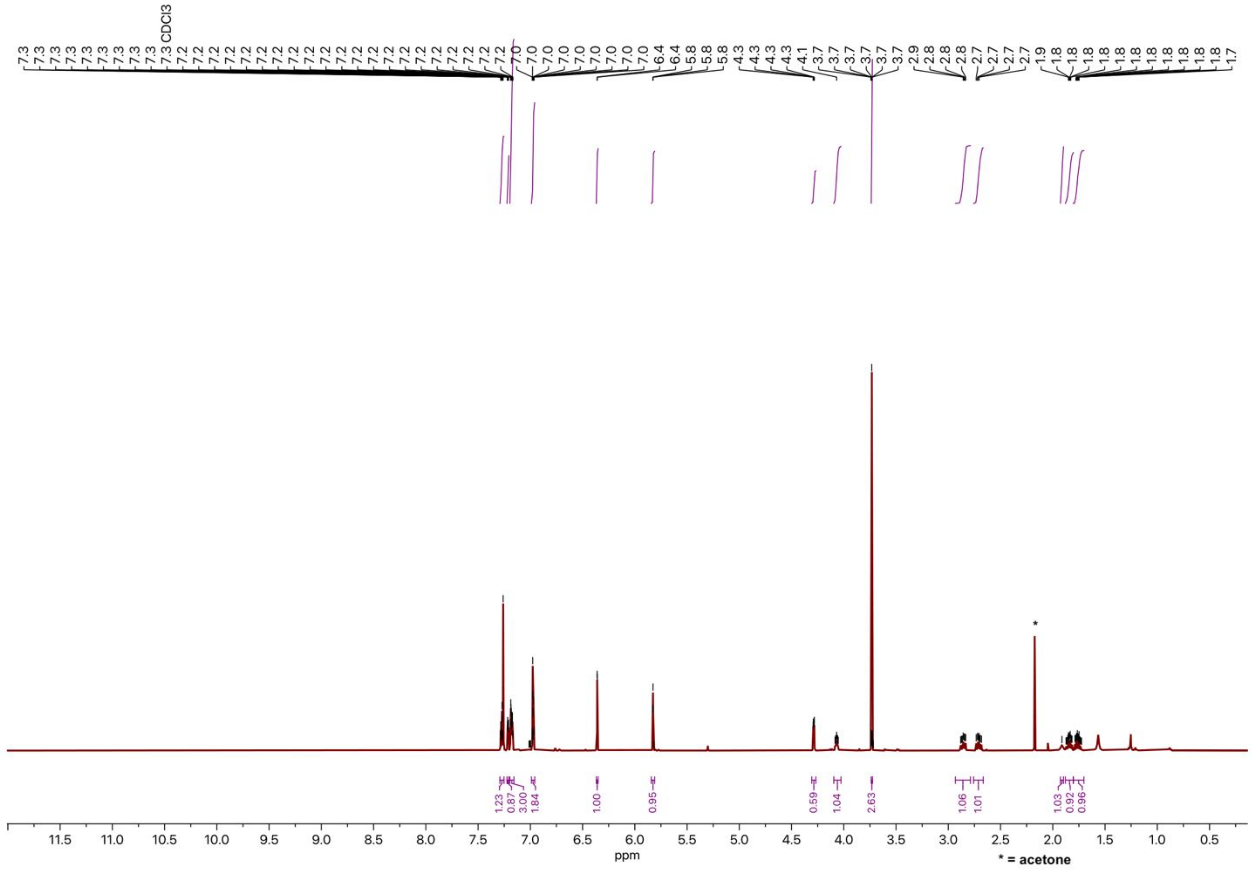

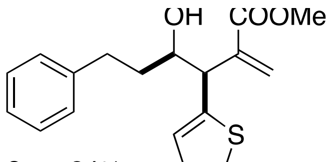

3qq, 24%
dr > 95:5

^{13}C{^{1}H} NMR (100 MHz, $CDCl_3$)

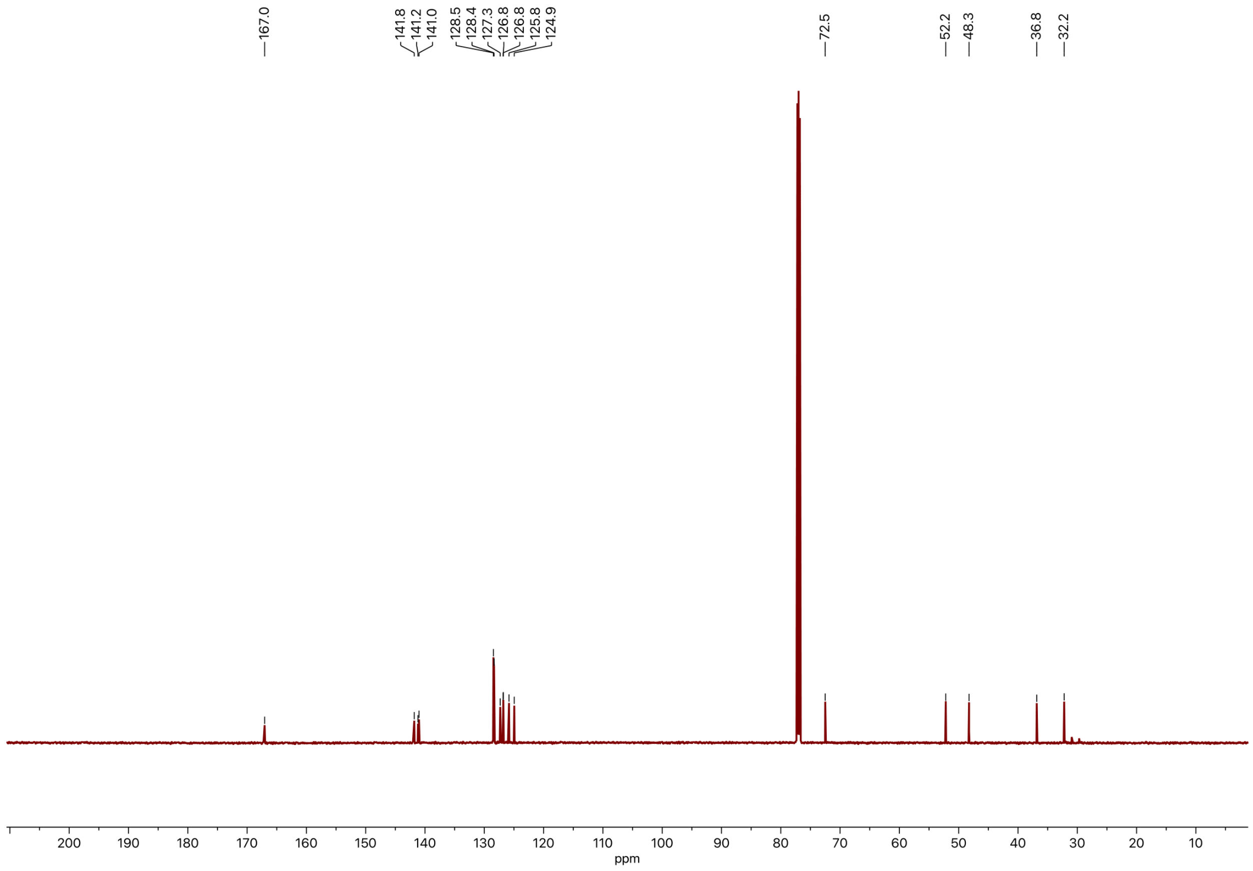

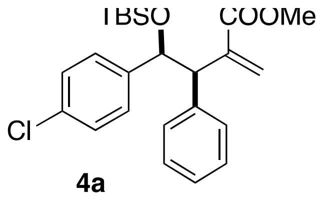

4a

65%, *dr > 20:1*

^{1}H NMR (400 MHz, $CDCl_3$)

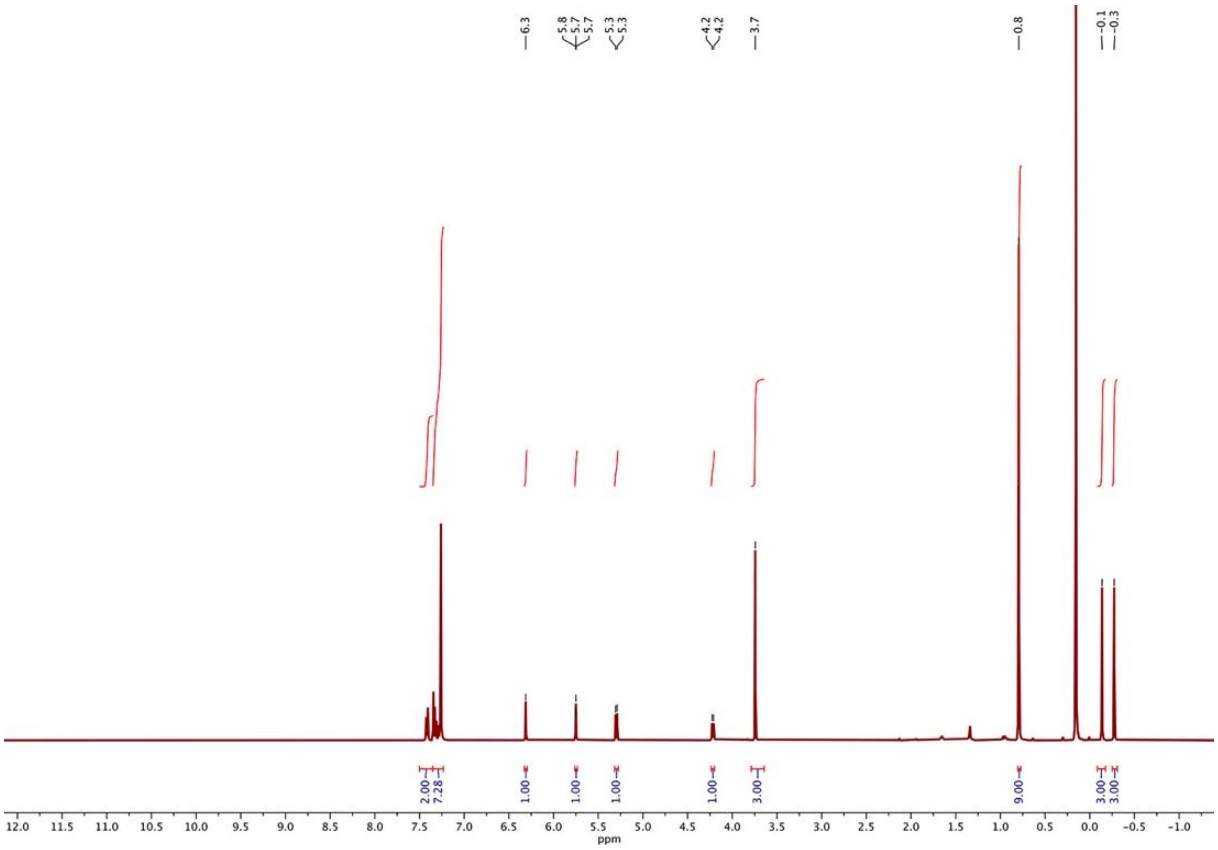

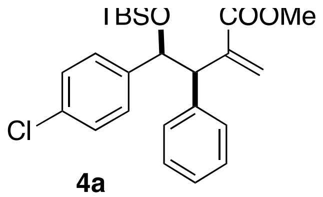

4a
65%, *dr > 20:1*

^{13}C{^{1}H} NMR (100 MHz, $CDCl_3$)

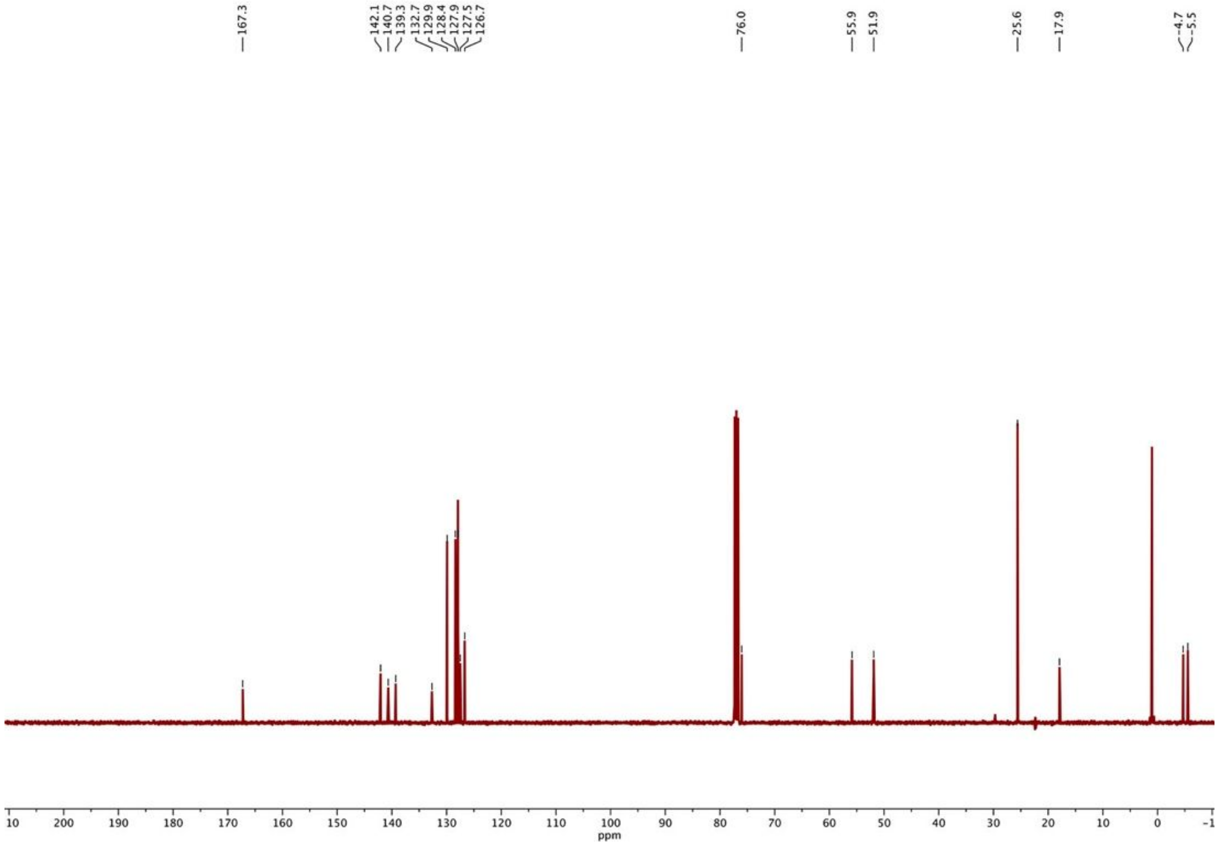

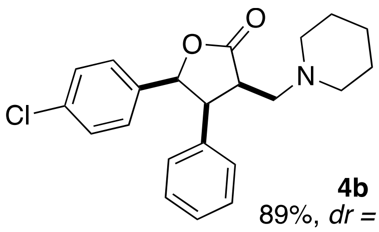

4b
89%, *dr* = *89:11*

^{1}H NMR (400 MHz, $CDCl_3$)

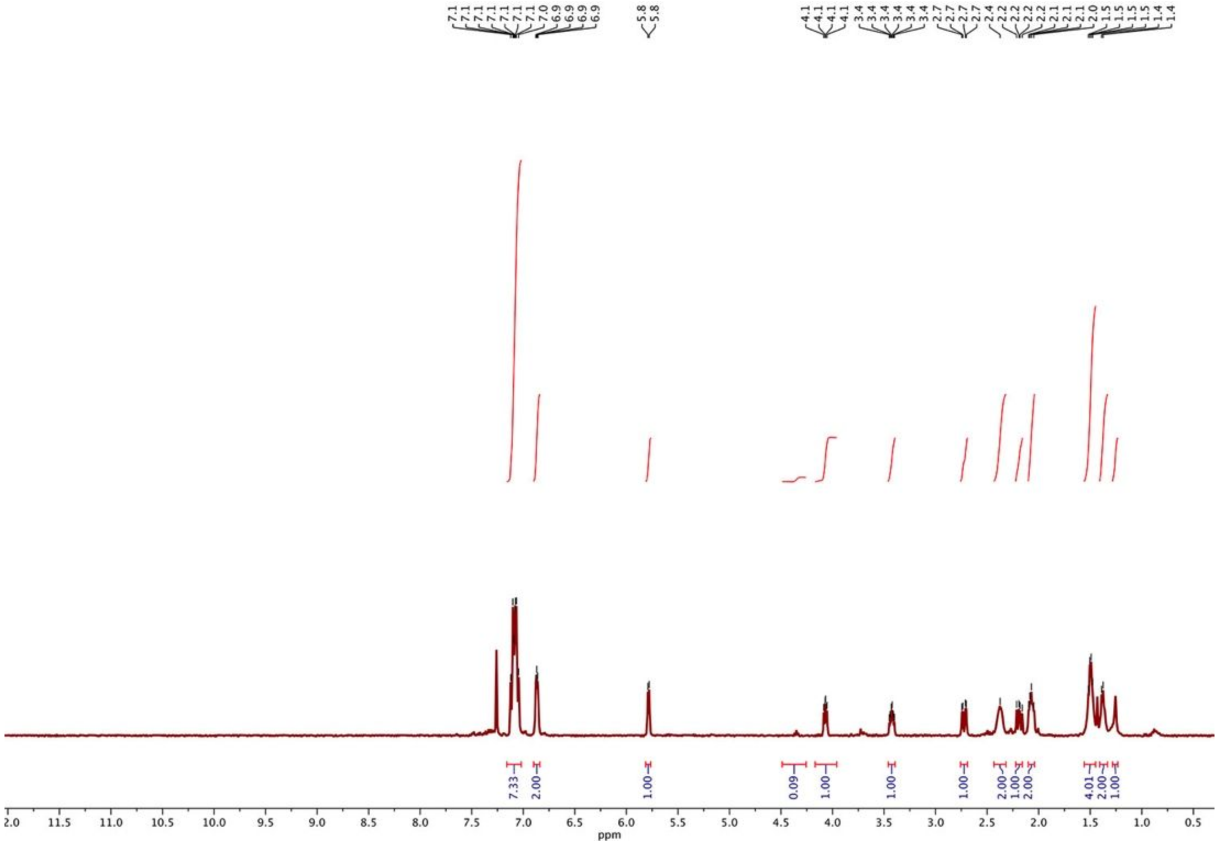

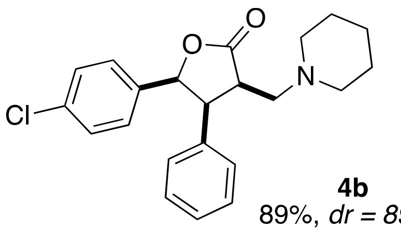

4b
89%, *dr = 89:11*

^{13}C{^{1}H} NMR (100 MHz, $CDCl_3$)

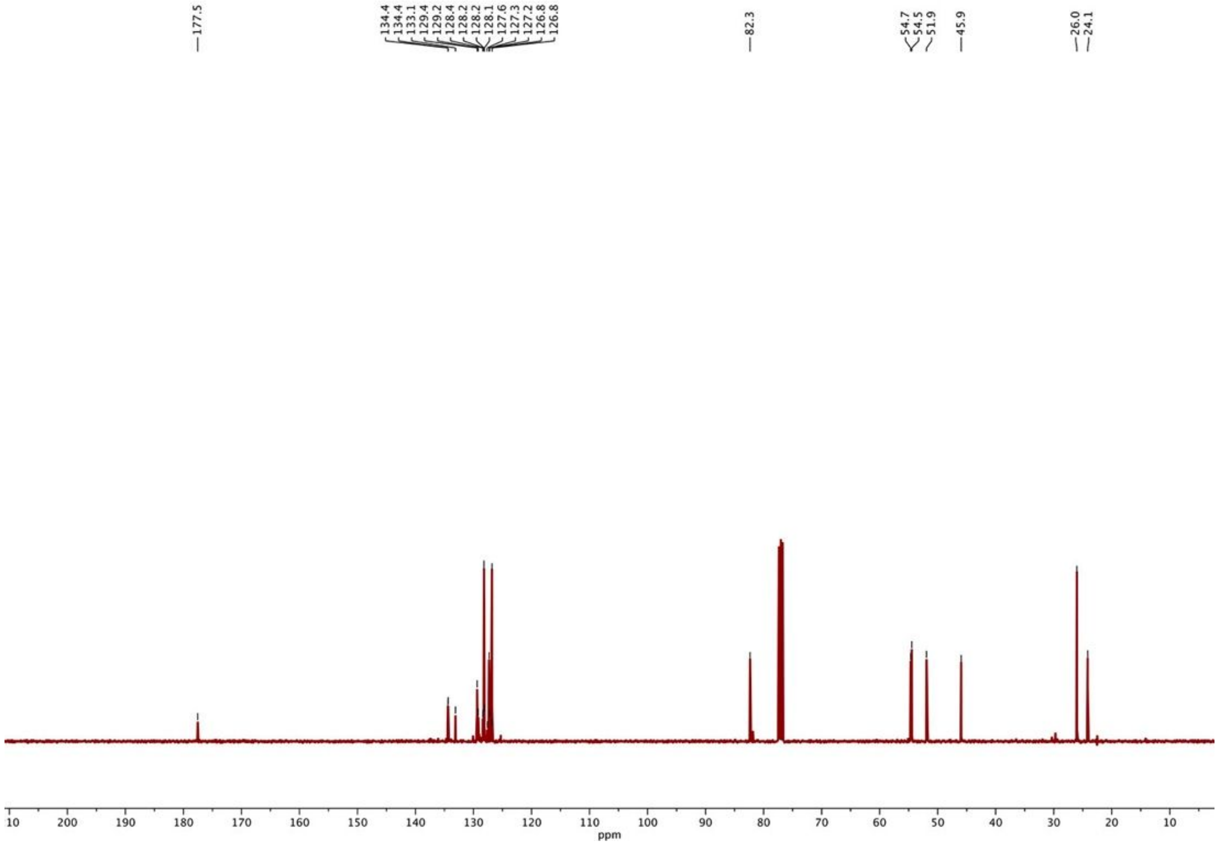

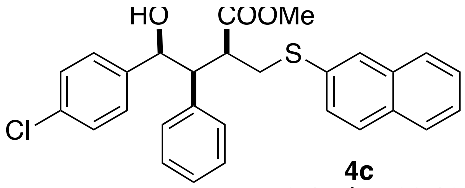

4c
78%, *dr = 88:12*

^{1}H NMR (400 MHz, CDCl$_3$)

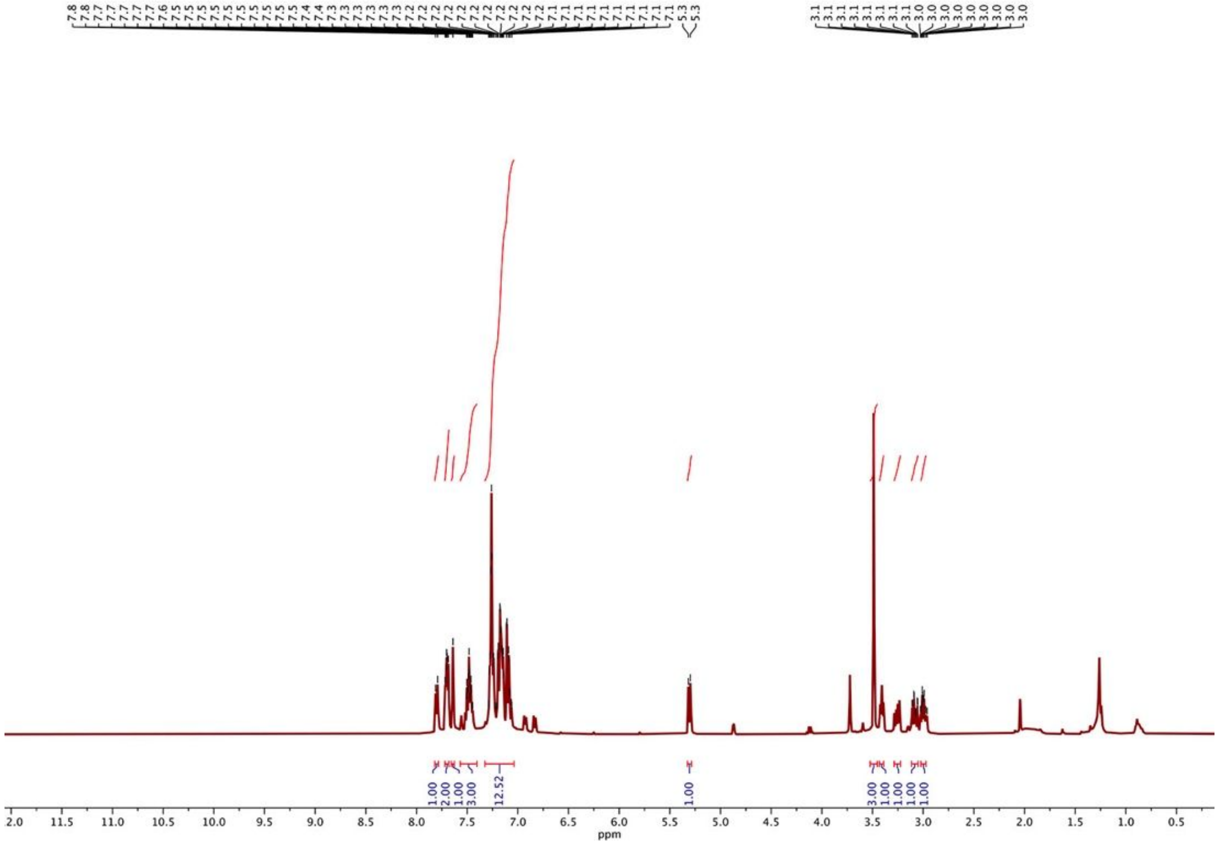

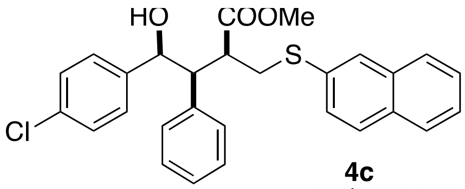

4c
78%, *dr = 88:12*

$^{13}C\{^1H\}$ NMR (100 MHz, $CDCl_3$)

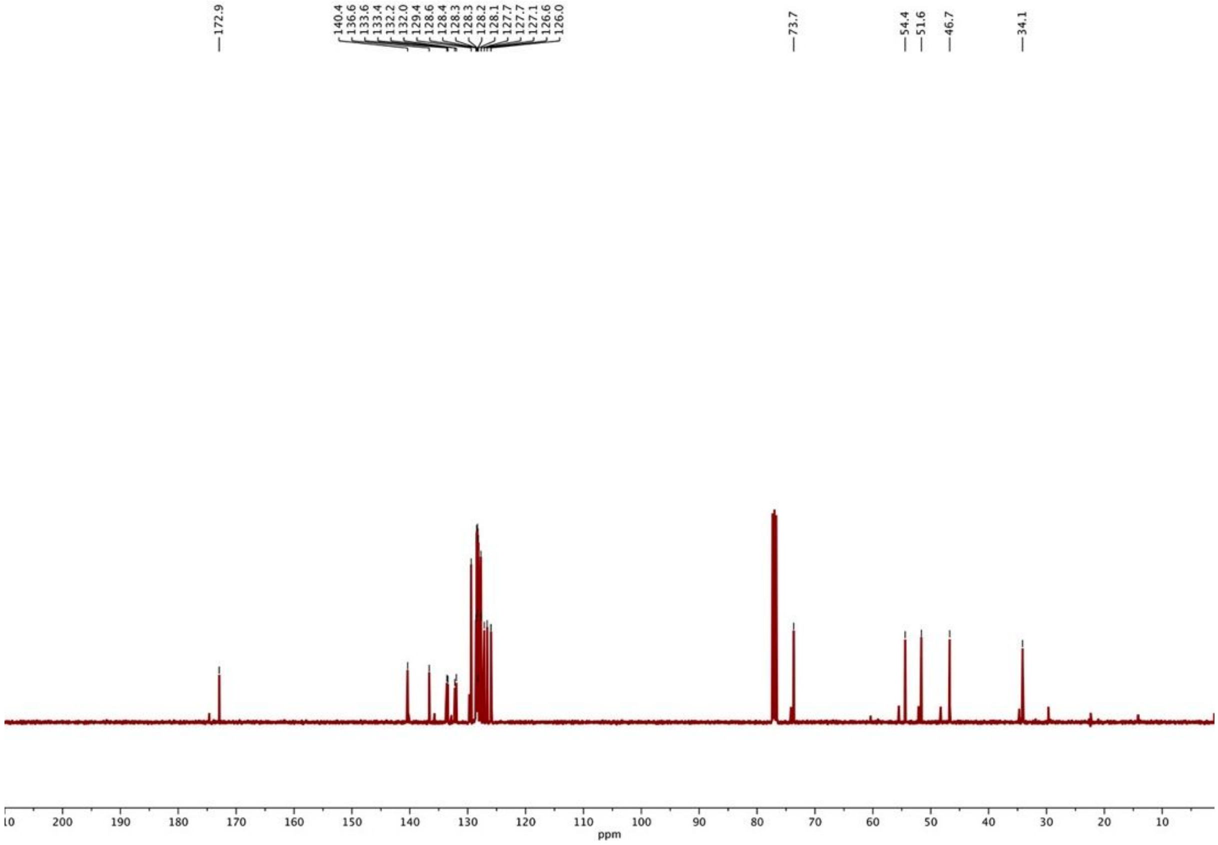

References

1 https://kessil.com/products/science_PR160L.php

2 Speckmeier, E.; Fischer, T. G.; Zeitler, K.; A Toolbox Approach To Construct Broadly Applicable Metal-Free Catalysts for Photoredox Chemistry: Deliberate Tuning of Redox Potentials and Importance of Halogens in Donor–Acceptor Cyanoarenes. *J. Am. Chem. Soc.* **2018,** *140*, 15353-15365.

3 Tatunashvili, E.; Chan, B.; Nashar, P. E.; McErlean, C. S. σ-Bond Initiated Generation of Aryl Radicals from Aryl Diazonium Salts. *Organic & Biomolecular Chemistry* **2020**, *18*, 1812–1819.

4 Huang, H.; Sun, B.; Huang, Y.; Niu, J. Radical Cascade-Triggered Controlled Ring-Opening Polymerization of Macrocyclic Monomers. *Journal of the American Chemical Society* **2018**, *140*, 10402–10406.

5 (a) Ramachandran, P. V.; Nicponski, D. R. Diastereoselective Synthesis of α-(Aminomethyl)-γ-Butyrolactones via a Catalyst-Free Aminolactonization. *Chemical Communications* **2014**, *50*, 15216–15219 and the references therein. (b) Bertuzzi, G.; Ombrosi, G.; Bandini, M. Regio-and Stereoselective Electrochemical Alkylation of Morita–Baylis–Hillman Adducts. *Organic Letters* **2022**, *24* (24), 4354–4359 and the references therein. (c) Batchu, H.; Bhattacharyya, S.; Batra, S. Iodine-Mediated Intramolecular Electrophilic Aromatic Cyclization in Allylamines: A General Route to Synthesis of Quinolines, Pyrazolo[4,3-b]pyridines, and Thieno[3,2-b]pyridines, *Org. Lett.* **2012**, *14*, 6330-6333 and references therein. (d) Juma, W. P.; Chhiba, V.; Brady, D.; Bode, M. L. Enzymatic Kinetic Resolution of Morita-Baylis-Hillman Acetates. *Tetrahedron: Asymmetry* **2017**, *28*, 1169–1174 and the references therein. (e) Ramachandran, P. V.; Pratihar, D.; Biswas, D.; Srivastava, A.; Ram Reddy, M. V. Novel Functionalized Trisubstituted Allylboronates via Hosomi− Miyaura Borylation of Functionalized Allyl Acetates. *Organic Letters* **2004**, *6*, 481–484. (f) Paula, B. R.; Zampieri, D.; Rodrigues, J. A. R.; Moran, P. J. Bioreduction of A-Acetoxymethyl Enones: Proposal for an SN2′ Mechanism Catalyzed by Enereductase. *Advanced Synthesis & Catalysis* **2016**, *358*, 3555–3571. (g) Enevoldsen, M. V.; Overgaard, J.; Pedersen, M. S.; Lindhardt, A. T. Organocatalyzed Decarboxylative Trichloromethylation of Morita–Baylis–Hillman Adducts in Batch and Continuous Flow. *Chemistry–A European Journal* **2018**, *24*, 1204–1208. (h) Nilov, D.; Raecker, R.; Reiser, O. Synthesis of 1, 4-Oxazepin-7-Ones Using Baylis-Hillman Products as Key Intermediates. *Synthesis* **2002**, 2232–2242. (i) Suga, T.; Takada, R.; Shimazu, S.; Sakata, M.; Ukaji, Y. Highly (E)-Selective Trisubstituted Alkene Synthesis by Low-Valent Titanium-Mediated Homolytic Cleavage of Alcohol C–O Bond. *The Journal of Organic Chemistry* **2022**, *87*, 7487–7493. (j) Li, L.; Chen, B.; Ke, Y.; Li, Q.; Zhuang, Y.; Duan, K.; Huang, Y.; Pang, J.; Qiu, L. Highly Efficient Synthesis of Heterocyclic and Alicyclic Β2-Amino Acid Derivatives by Catalytic Asymmetric Hydrogenation. *Chem.– Asian J.* **2013**, *8*, 2167–2174.

6 (a) Dong, S.; Wang, T.; Hu, C.; Chen, X.; Jin, Y.; Wang, Z. Design and Synthesis of 5-Substituted Benzo [d][1, 3] Dioxole Derivatives as Potent Anticonvulsant Agents. *Archiv der Pharmazie* **2017**, *350*, 1600274. (b) Zuckerman, D. S.; Woerpel, K. Diastereoselective Peroxidation of Derivatives of Baylis–Hillman Adducts. *Tetrahedron* **2019**, *75*, 4118–4129. (c) Hewitt, K. A.; Herbert, C. A.; Jarvo, E. R. Synthesis of Vicinal Carbocycles by Intramolecular Nickel-Catalyzed Conjunctive Cross-Electrophile Coupling Reaction. *Org. Lett.* **2022**, *24*, 6093–6098. (d) Huang, H.; Yu, C.; Zhang, Y.; Zhang, Y.; Mariano, P. S.; Wang, W. Chemo-and Regioselective Organo-Photoredox Catalyzed Hydroformylation of Styrenes via a Radical Pathway. *J. Am. Chem. Soc.* **2017**, *139*, 9799–9802. (e) Aoyagi, Y.; Saitoh, Y.; Ueno, T.; Horiguchi, M.; Takeya, K.; Williams, R. M., Lipase TL-Mediated Kinetic Resolution of 5-Benzyloxy-1-tert-butyldimethylsilyloxy-2-pentanol at Low Temperature: Concise Asymmetric Synthesis of Both Enantiomers of a Piperazic Acid Derivative. *J. Org. Chem.* **2003,** *68*, 6899-6904. (f) Koyanagi, T.; Leriche, G.; Onofrei, D.; Holland, G. P.; Mayer, M.; Yang, J., Cyclohexane Rings Reduce Membrane Permeability to Small Ions in Archaea-Inspired Tetraether Lipids. *Angew. Chem. Int. Ed.* **2016,** *55*, 1890-1893. (g) Romano, C.; Fiorito, D.; Mazet, C. Remote Functionalization of α, β-Unsaturated Carbonyls by Multimetallic Sequential Catalysis. *J. Am. Chem. Soc.* **2019**, *141*, 16983–16990. (h) Mason, H. P.; Emslie N. D. Some mechanistic and synthetic aspects of the DABCO catalysed rearrangement of allylic esters. *Tetrahedron*, **1994**, *50* 12001-12008. (i) Sakurada. T.; Takagi K.; Murofushi H. polymerizable compound, curable composition and cured article. JP 7 056 141 B2, 2022.

7 Ko-Hoon, K.; Hyun-Seung, L.; Sung-Hwan, K.; Ka-Young, L.; Eun,L.; Jae-Nyoung, K.; Expedient One-Pot Synthesis of γ-hydroxybutenolides Starting from Baylis-Hillman Adducts: Lactonization, Isomerization, and Aerobic Oxidation of α-Methylene-γ-hydroxyester *Bull. Korean Chem. Soc.* **2009**, *30*, 1012-1020.

[8] Hamann, H. J.; Abutaleb, N. S.; Pal, R.; Seleem, M. N.; Ramachandran, P. V. β,γ-Diaryl α-Methylene-γ-Butyrolactones as Potent Antibacterials against Methicillin-Resistant Staphylococcus Aureus. *Bioorg. Chem.* **2020**, *104*, 104183.

[9] Park, B. R.; Kim, K. H.; Kim, J. N. An Efficient Synthesis of α-Methylene-γ-Butyrolactones from Baylis–Hillman Adducts via an In-Mediated Barbier Reaction and Stereoselective Lactonization under $MeSO_2Cl/Et_3N$ Conditions. *Tetrahedron Letters* **2010**, *51*, 6568–6571.

[10] Ramachandran, P. V.; Helppi, M. A.; Lehmkuhler, A. L.; Marchi, J. M.; Schmidt, C. M.; Yip-Schneider, M. T. Factors Influencing the Cytotoxicity of α-Methylene-γ-Hydroxy Esters against Pancreatic Cancer. *Bioorg. Med. Chem. Lett.* **2015**, *25*, 4270–4273.

[11] Calogero, F.; Potenti, S.; Bassan, E.; Fermi, A.; Gualandi, A.; Monaldi, J.; Dereli, B.; Maity, B.; Cavallo, L.; Ceroni, P.; Cozzi, P. G. Nickel Mediated enantioselective photoredox allylation of aldehydes with visible light. *Angew. Chem. Int. Ed.* **2021**, *61*, e202114981

[12] Frisch, M. J.; Trucks, G. W.; Schlegel, H. B.; Scuseria, G. E.; Robb, M. a.; Cheeseman, J. R.; Scalmani, G.; Barone, V.; Petersson, G. a.; Nakatsuji, H.; Li, X.; Caricato, M.; Marenich, a. V.; Bloino, J.; Janesko, B. G.; Gomperts, R.; Mennucci, B.; Hratchian, H. P.; Ortiz, J. V.; Izmaylov, a. F.; Sonnenberg, J. L.; Williams; Ding, F.; Lipparini, F.; Egidi, F.; Goings, J.; Peng, B.; Petrone, A.; Henderson, T.; Ranasinghe, D.; Zakrzewski, V. G.; Gao, J.; Rega, N.; Zheng, G.; Liang, W.; Hada, M.; Ehara, M.; Toyota, K.; Fukuda, R.; Hasegawa, J.; Ishida, M.; Nakajima, T.; Honda, Y.; Kitao, O.; Nakai, H.; Vreven, T.; Throssell, K.; Montgomery Jr., J. a.; Peralta, J. E.; Ogliaro, F.; Bearpark, M. J.; Heyd, J. J.; Brothers, E. N.; Kudin, K. N.; Staroverov, V. N.; Keith, T. a.; Kobayashi, R.; Normand, J.; Raghavachari, K.; Rendell, a. P.; Burant, J. C.; Iyengar, S. S.; Tomasi, J.; Cossi, M.; Millam, J. M.; Klene, M.; Adamo, C.; Cammi, R.; Ochterski, J. W.; Martin, R. L.; Morokuma, K.; Farkas, O.; Foresman, J. B.; Fox, D. J.*Gaussian 16*, *Revision B.01*, Gaussian, Inc., Wallingford CT, **2016**.

[13] Marenich, A. V; Cramer, C. J.; Truhlar, D. G. Universal Solvation Model Based on Solute Electron Density and on a Continuum Model of the Solvent Defined by the Bulk Dielectric Constant and Atomic Surface Tensions. *J. Phys. Chem. B* **2009**, *113*, 6378-6396.

[14] (a) Perdew, J. P.; Burke, K.; Ernzerhof, M. Generalized Gradient Approximation Made Simple. *Phys. Rev. Lett.* **1996**, *77*, 3865-3868; (b) Perdew, J. P.; Burke, K.; Ernzerhof, M.Generalized Gradient Approximation Made Simple *Phys. Rev. Lett.* **1997**, *78*, 1396.

[15] Grimme, S.; Antony, J.; Ehrlich, S.; Krieg, H. A Consistent and Accurate Ab Initio Parametrization of Density Functional Dispersion Correction (DFT-D) for the 94 Elements H-Pu. *J. Chem. Phys.* **2010**, *132*, 154104.

[16] (a) F. Weigend, R. Ahlrichs, *Phys. Chem. Chem. Phys.* **2005**, *7*, 3297-3305; (b) Weigend, F. Accurate Coulomb-Fitting Basis Sets for H to Rn. Phys. *Phys. Chem. Chem. Phys.* **2006**, *8*, 1057-1065.

[17] Halgren, T. A.; Lipscomb, W. N. The Synchronous-Transit Method for Determining Reaction Pathways and Locating Molecular Transition States. *Chem. Phys. Lett.* **1977**, *49*, 225-232.

[18] (a) Hratchian, H. P.; Schlegel, H. B. Accurate Reaction Paths Using a Hessian Based Predictor–Corrector Integrator. *J. Chem. Phys.* **2004**, *120*, 9918-9924; (b) Fukui, K. The Path of Chemical Reactions - the IRC Approach. *Acc. Chem. Res.* **1981**, *14*, 363-368.

[19] Zhao, Y.; Truhlar, D. G. The M06 Suite of Density Functionals for Main Group Thermochemistry, Thermochemical Kinetics, Noncovalent Interactions, Excited States, and Transition Elements: Two New Functionals and Systematic Testing of Four M06-Class Functionals and 12 Other Functionals. *Theor. Chem. Acc.* **2008**, *120*, 215-241.

[20] (a) Kitaura, K.; Morokuma, K.; A new energy decomposition scheme for molecular interactions within the Hartree-Fock approximation. *Int. J. Quantum Chem.* **1976**, *10*, 325-340; (b) Bickelhaupt, F. M.; Houk, K. N. Analyzing reaction rates with the distortion/interaction-activation strain model. *Angew. Chem. Int. Ed.* **2017**, *56*, 10070-10086.

[21] (a) Marcus, R. A. On the theory of oxidation-reduction reactions involving electron transfer. I. *J. Chem. Phys.* **1956**, *24*, 966-978. (b) Hush, N. S. Adiabatic theory of outer sphere electron-transfer reactions in solution. *Trans. Faraday Soc.* **1961**, *57*, 557-580.
